# Collective Synthesis of Illudalane Sesquiterpenes via Cascade Inverse Electron Demand (4 + 2) Cycloadditions of Thiophene *S*,*S*-Dioxides

**DOI:** 10.1021/jacs.2c03304

**Published:** 2022-05-24

**Authors:** Kun Ho Kenny Park, Nils Frank, Fernanda Duarte, Edward A. Anderson

**Affiliations:** Chemistry Research Laboratory, 12 Mansfield Road, Oxford OX1 3TA, U.K.

## Abstract

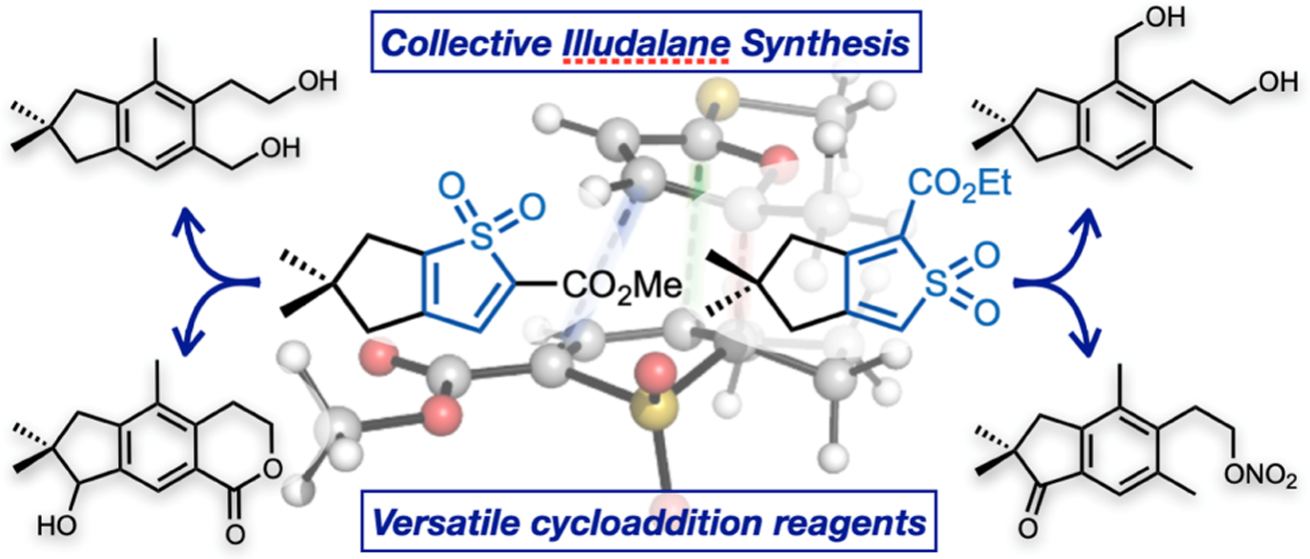

Thiophene *S,S-*dioxides are underutilized tools for the *de novo* construction of benzene rings in organic synthesis. We report a collective synthesis of nine illudalane sesquiterpenes using bicyclic thiophene *S,S-*dioxides as generalized precursors to the indane core of the natural products. Exploiting furans as unusual dienophiles in this inverse electron demand Diels–Alder cascade, this concise and convergent approach enables the synthesis of these targets in as little as five steps. Theoretical studies rationalize the reactivity of thiophene *S,S-*dioxides with both electron-poor and electron-rich dienophiles and reveal reaction pathways involving either nonpolar pericyclic or bifurcating ambimodal cycloadditions. Overall, this work demonstrates the wider potential of thiophene *S,S*-dioxides as convenient and flexible precursors to polysubstituted arenes.

## Introduction

**Figure 1:**
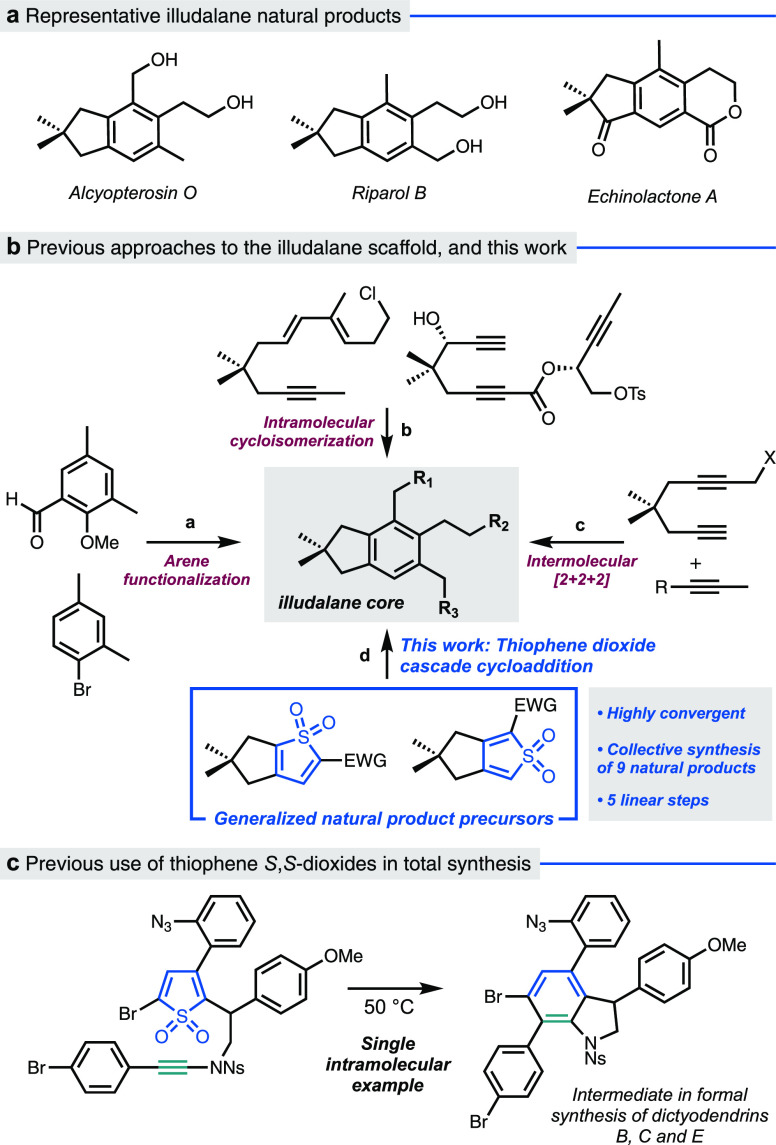
(a) Illudalane natural products. (b) Previous approaches and the strategy employed in this work. (c) Intramolecular thiophene *S*,*S*-dioxide/ynamide cycloaddition in the formal synthesis of dictyodendrins B, C, and E.^8^

Polysubstituted benzene rings are challenging synthetic targets due to the difficulty of regioselective introduction of different functional groups onto the aromatic core. Such motifs are commonly found in natural products such as the illudalane sesquiterpenoids^1^ (Figure 1a), pharmaceuticals,^2^ and organic materials.^3^ In the context of the illudalane natural products, the functionalization of pre-formed arenes offers one approach (Figure 1b, path a), but this can be challenging due to issues of regioselectivity compounded by steric considerations, resulting in lengthy synthetic routes that are specific to a single target.^4^ An alternative strategy involves the *de novo* construction of the benzene ring, simultaneously installing all required substituents; for the illudalanes, this has almost exclusively involved fully intramolecular (path b),^5^ or two-component (path c) metal-catalyzed alkyne cyclizations.^6^

A different approach to benzene synthesis involves the Diels–Alder cycloaddition/retro-cycloaddition of dienes equipped with “leaving groups” such as N_2_, nitriles, and CO_2_.^7^ Single-atom variants such as SO_2_ or CO (i.e., cheletropic extrusions) are also possible but have been surprisingly little used in target-oriented synthesis. In the case of SO_2_ extrusion, the majority of research has exploited the ability of sulfolenes (2,5-dihydrothiophenes) to reveal dienes by the loss of SO_2_, which subsequently engage with a dienophile.^9^ Far less studied are thiophene *S,S-*dioxides: to the best of our knowledge, only a single use of these motifs in natural product synthesis exists in an elegant formal synthesis of dictyodendrins B, C, and E by Kabuki and Yamaguchi, involving intramolecular cycloaddition of an electron-rich ynamide with a thiophene *S,S-*dioxide (Figure 1c).^8,10^ Simple intermolecular cycloadditions with alkynes, alkenes, and furans are known in a methodological context but have mainly employed (poly)halogenated or symmetric thiophenes.^11^

We questioned whether thiophene *S,S-*dioxides could offer an efficient and general entry to the illudalane sesquiterpenoid natural products and specifically if intermolecular cycloaddition cascades of these substrates, which are unprecedented in natural product synthesis, could be employed in this context (Figure 1b, path d). Here, we describe concise syntheses of nine members of the illudalane natural product family, where variation of the orientation of a bicyclic thiophene *S,S-* dioxide (i.e., a 2,3- or 3,4-fused bicyclic framework) brings strategic flexibility in the positioning of the arene substituents. We also report theoretical studies into the nature of thiophene *S,S-*dioxide cycloaddition reactions: studies using both electron-poor and electron-rich dienophiles revealed a balance of reactivity pathways from classical nonpolar Diels–Alder to bifurcating “ambimodal” cycloadditions.

## Results and Discussion

**Scheme 1:**
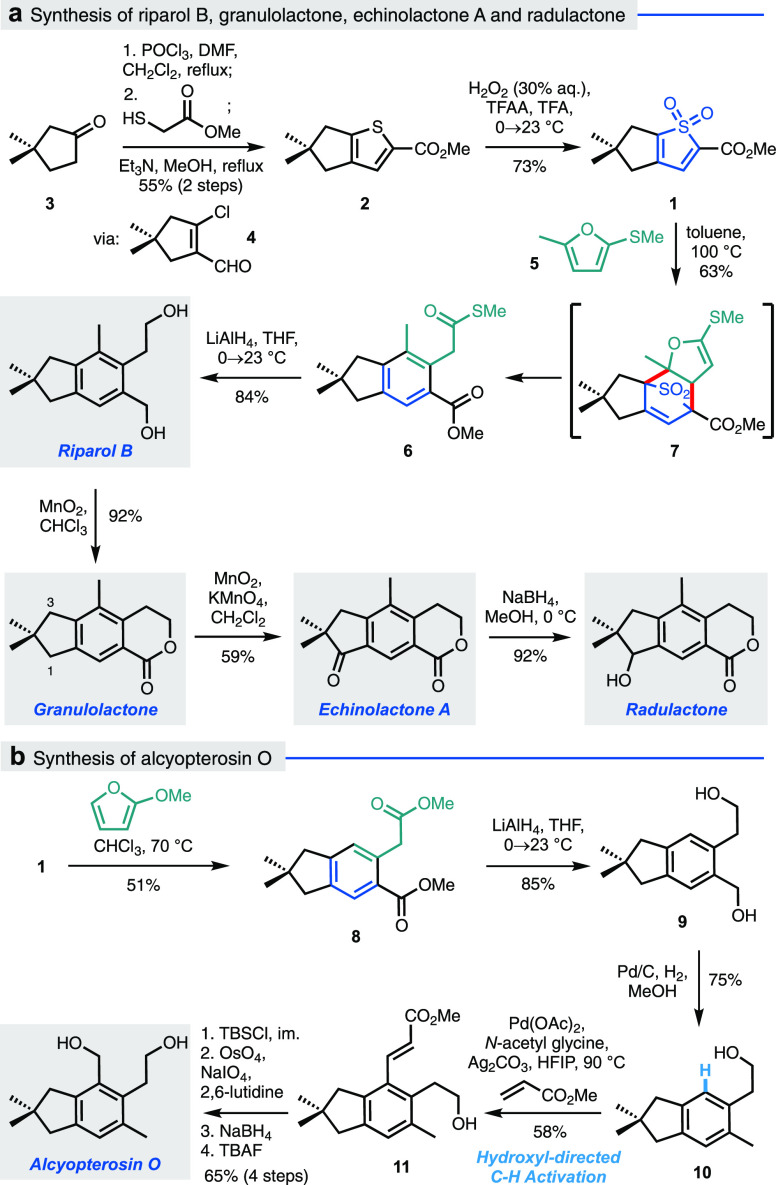
2,3-Fused Bicyclic Thiophene *S,S*-Dioxide 1 as a Precursor to Five Illudalane Natural Products

Our studies commenced with the preparation of the 2,3-fused bicyclic thiophene *S,S-*dioxide **1** (Scheme 1). Thiophene **2** was first constructed using a Fiesselmann synthesis,^12^ whereby the reaction of commercially available 3,3-dimethylcyclopentanone **3** with POCl_3_/DMF led to intermediate β-chloroenal **4**, which was directly reacted with methyl thioglycolate to yield **2**. Oxidation of **2** to the targeted *S,S-*dioxide **1** was initially found to be challenging due to the electron-withdrawing methyl ester, with oxidants such as *m*-CPBA or Oxone proving unsuccessful. However, the use of *in situ*-generated trifluoroacetic peracid, as described by Nenajdenko and co-workers,^13^ afforded **1** in good yield (73%).

With **1** in hand, the key intermolecular cycloaddition/SO_2_ extrusion was investigated. Efforts to engage **1** with various alkynes were unsuccessful, but to our delight, the use of furan **5** as dienophile (at 100 °C in toluene) constructed the illudalane benzenoid core **6** in good yield. This reaction presumably proceeds via the initial formation of the (4 + 2) adduct **7**, which aromatizes via cheletropic extrusion of SO_2_ and ring-opening elimination of the furan; however, the path for the formation of **7** could also conceivably involve a stepwise sequence of 1,6-Michael addition followed by cyclization of an intermediate zwitterion (i.e., the extreme of a concerted asynchronous cycloaddition pathway, *vide infra*). Notwithstanding the reaction pathway, the illudalane core was thus constructed in just four steps from commercial materials.

The presence of the two carbonyl functionalities in **6** facilitates direct access to a number of illudalane natural products. Reduction of **6** with LiAlH_4_ afforded riparol B, the selective benzylic oxidation of which gave granulolactone in high yield. Mindful of the previously reported conversion of granulolactone to echinolactone A by Zhang and co-workers,^6c^ in which the efficiency of indane oxidation was hampered by poor differentiation between C1 and C3 (using CrO_3_/AcOH), more selective conditions were explored. Pleasingly, we found that the use of the heterogeneous co-oxidant system KMnO_4_/MnO_2_^14^ conferred respectable selectivity for oxidation at C1 (3:1), giving echinolactone A in 59% yield; reduction with NaBH_4_ afforded radulactone.^6c^

Thiophene dioxide **1** could also be deployed in the synthesis of alcyopterosin O (Scheme 1b). Subjection of **1** to the cycloaddition cascade with 2-methoxyfuran afforded the bicyclic tetrasubstituted arene **8**; reduction of both esters (**9**) followed by selective hydrogenolysis of the benzylic alcohol gave **10**. To introduce the required hydroxymethyl group onto the benzene ring, the remaining alcohol was then utilized in a hydroxyl-directed *ortho* C–H alkenylation;^15^ pleasingly, this afforded enoate **11** in a 58% yield, which was advanced to the natural product in a further four steps (65%).

**Scheme 2:**
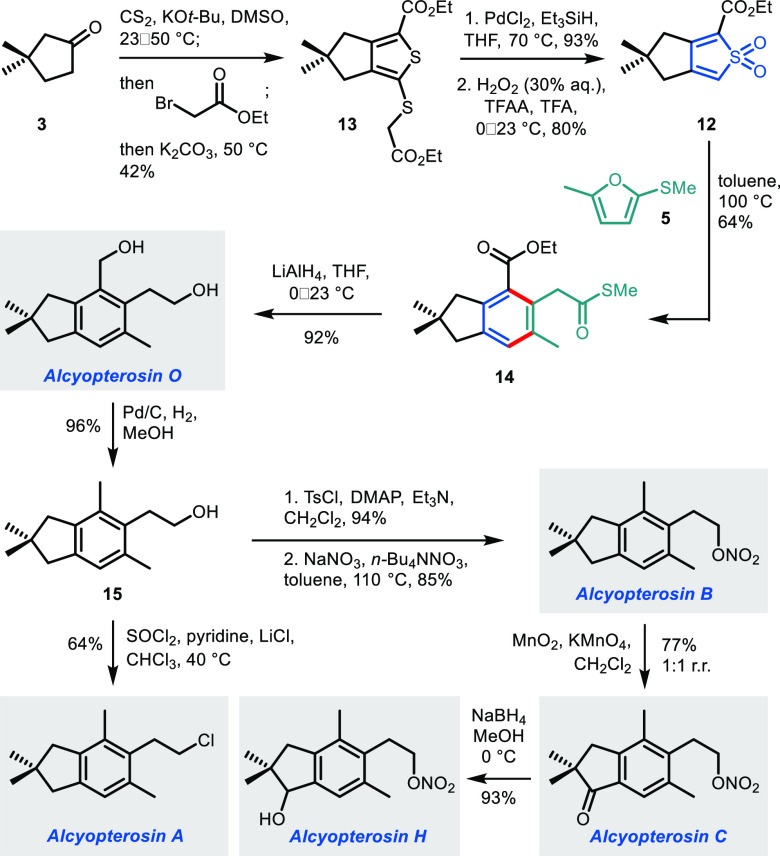
3,4-Fused Bicyclic Thiophene *S,S*-Dioxide 12 as a Precursor to Alcyopterosins O, A, B, C, and H

Although alcyopterosin O could be accessed using this route, we questioned whether an alternative approach could be devised with a lower step count. Comparison of alcyopterosin O with riparol B reveals the structural source of the problem—namely, the transposition of the arene methyl and hydroxymethyl substituents between the two targets, which necessitated multiple post-cycloaddition manipulations. A different approach involves “rotating” the orientation of the thiophene relative to its fused cyclopentane ring to enable the direct introduction of all arene substituents with suitable degrees of oxygenation. Thiophene *S,S*-dioxide **12** (Scheme 2) was therefore targeted, the synthesis of which again commenced with 3,3-dimethylcyclopentanone **3**. Treatment of **3** with carbon disulfide under basic conditions, followed by the addition of ethyl bromoacetate, led to thiophene **13**. Reduction of the exocyclic C–S bond using PdCl_2_/Et_3_SiH, followed by oxidation to the bicyclic thiophene *S,S*-dioxide **12**, proceeded in high yield (74% over two steps). The key (4 + 2) cycloaddition/SO_2_ extrusion step again proceeded smoothly using furan **5**, giving 2,2-dimethylindane core **14** with the methyl group “transposed” to the C6 position. Reduction of the two carboxyl functionalities completed the synthesis of alcyopterosin O in a much improved five steps from **3**.

Once again, this initial natural product served as a gateway to other members of the illudalane family. Hydrogenolysis of the benzylic alcohol (**15**) followed by chlorination gave alcyopterosin A. Alternatively, tosylation of **15** and reaction with NaNO_3_ afforded alcyopterosin B, which could be converted to alcyopterosin C by oxidation with KMnO_4_/MnO_2_, albeit this time with poor regioselectivity (1:1) owing to the lack of an electron-withdrawing group on the arene. Finally, reduction of the indanone gave alcyopterosin H.

**Figure 2:**
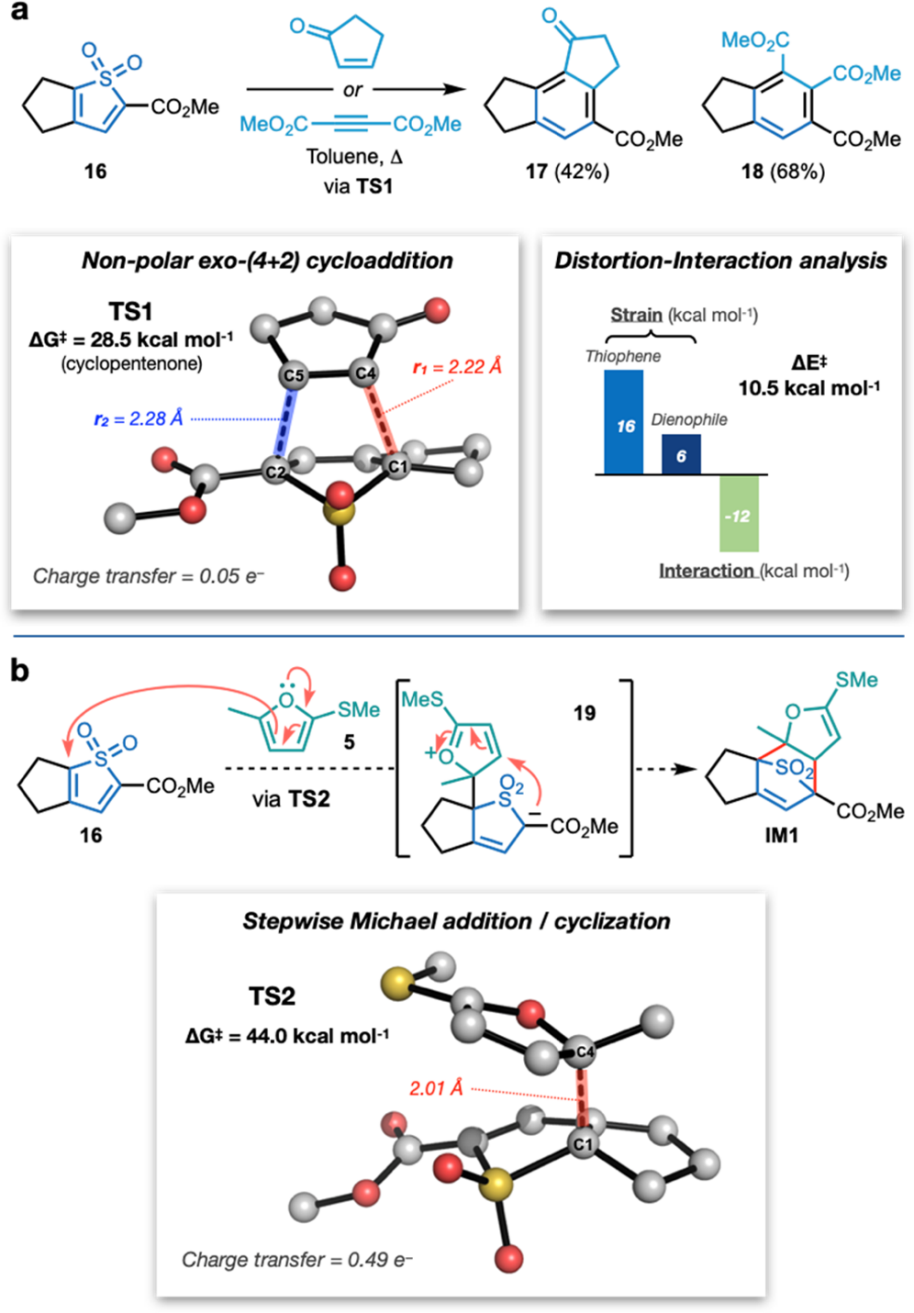
(a) Reaction of thiophene *S,S*-dioxide **16** with electron-deficient dienophiles, calculated **TS1** for nonpolar Diels–Alder reaction of **16** with cyclopentenone, and distortion–interaction analysis. (b) Possible stepwise Michael addition/cyclization mechanism and calculated **TS2** for the reaction of **16** with furan **5**. Calculations at the CPCM(toluene)-DLPNO-CCSD(T)/def2-TZVPP//CPCM(toluene)-M06-2X/def2-TZVPP level of theory at 373.15 K/1 M. Hydrogen atoms are omitted for clarity.

The facile reaction of furans with the thiophene *S,S-*dioxides led us to question the nature of the cycloaddition process. The ability of thiophene *S,S-*dioxides to undergo genuine (4 + 2) cycloadditions was first confirmed by the successful reaction of *S*,*S*-dioxide **16** with cyclopentenone and dimethylacetylene dicarboxylate (DMAD; Figure 2a). Computational examination of these reactions led to the identification of pericyclic transition states (e.g., Figure 2a; **TS1** for reaction with cyclopentenone, Δ*G*^‡^ = 28.5 kcal mol^–1^).

Domingo et al. have classified Diels–Alder (DA) reactions as “nonpolar” or “polar”.^16^ Nonpolar DA reactions are characterized by relatively high activation energies and proceed through highly synchronous pathways in which the transition state (TS) does not involve significant charge transfer between the fragments (CT < 0.1 e^–^). Polar DA reactions exhibit lower activation energies and are typified by asynchronous bond formation with significant charge transfer between the two fragments at the TS (CT > 0.1 e^–^). The reactions of **16** with the electron-deficient dienophiles cyclopentenone or DMAD can thus be interpreted as nonpolar DA reactions due to limited charge transfer (Figure 2a and Table S6, 0.05 e^–^ and 0.03 e^–^, respectively) and only slight asynchronicity (*r*_2_ – *r*_1_ = 0.05 and 0.08 Å, respectively).^17^ Distortion–interaction analysis^18^ of **TS1** revealed a modest distortion of the dienophile compared to that of the diene and an interaction energy of 12 kcal mol^–1^.

Attempts to identify a pericyclic (4 + 2) TS for the reaction of **16** with furan **5** were unsuccessful; however, this reaction could also proceed by a 1,6-Michael addition/cyclization via zwitterion **19** (Figure 2b). A transition state was found for this Michael addition (**TS2**), but displayed a significant activation barrier of 44.0 kcal mol^–1^. The magnitude of this barrier appears to be inconsistent with the observed reactivity.

**Figure 3:**
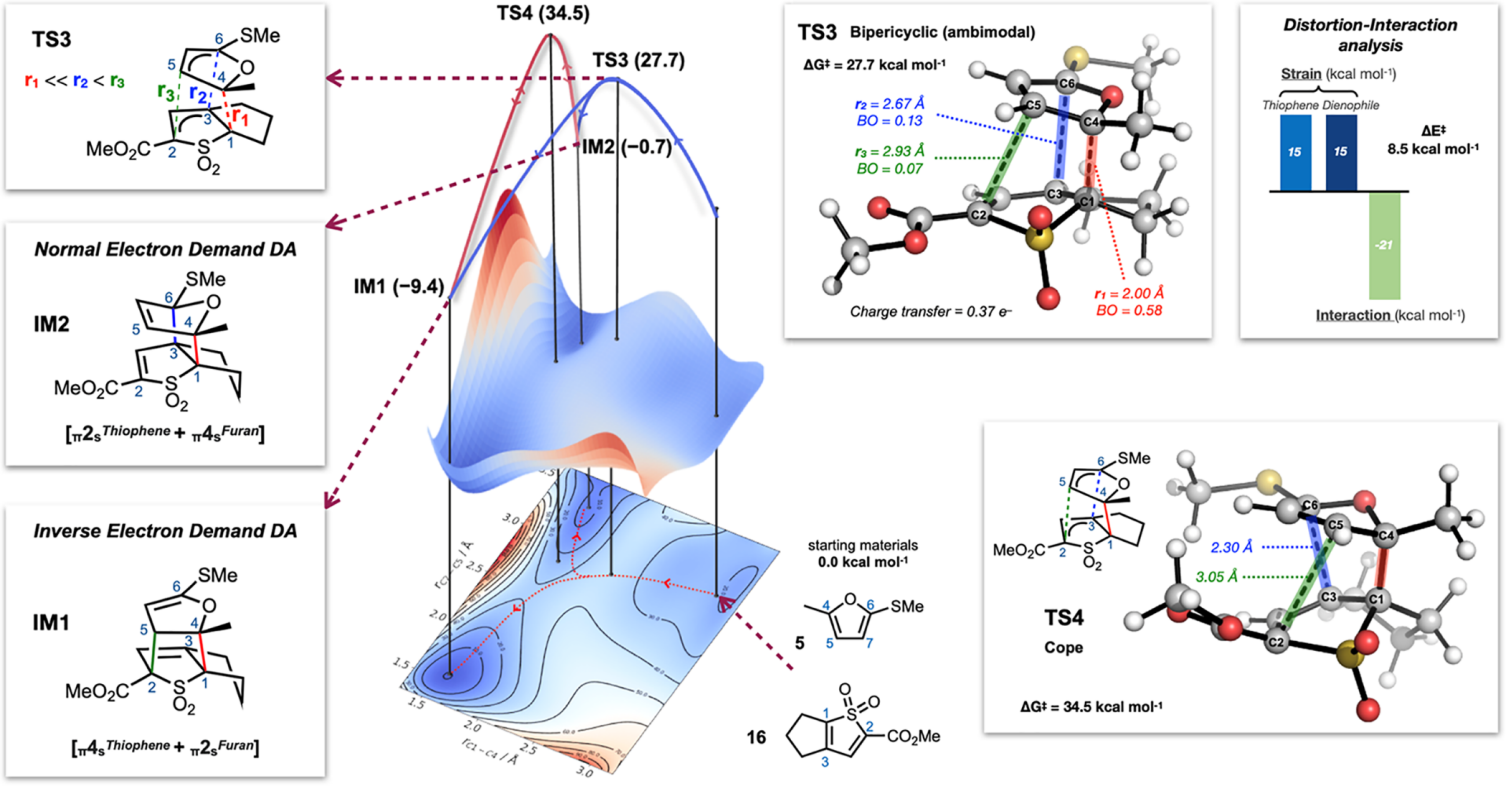
More O’Ferrall–Jencks plot for the reaction between **16** and **5** as a function of the forming C1–C4 (red), C2–C5 (green) bond distances calculated at the CPCM(toluene)-M06-2X/def2-SVP level of theory. Minima and TSs were further optimized at the CPCM(toluene)-DLPNO-CCSD(T)/def2-TZVPP//CPCM(toluene)-M06-2X/def2-TZVPP level of theory at 373.15 K/1 M. Gibbs free energies are reported in kcal mol^–1^ relative to the starting materials.

The concerted cycloaddition pathway was then explored in greater depth by constructing a More O’Ferrall–Jencks plot^19^ for the reaction of **16** with **5** as a function of the forming C1–C4 and C2–C5 bonds (Figure 3). This revealed a third possibility: a highly asynchronous, ambimodal pathway that proceeds via **TS3** (Δ*G*^‡^ = 27.7 kcal mol^–1^). This transition state features partial bond formation between the diene and dienophile at three distinct positions—one advanced (*r*_1_, C1–C4, 2.00 Å) and two less advanced (*r*_2_, C3–C6, 2.67 Å; and *r*_3_, C2–C5, 2.93 Å), with significant charge transfer observed (CT = 0.37 e^–^)—and is favored over the Michael addition pathway (**TS2**) by 16 kcal mol^–1^. Characteristic of **TS3** is a bifurcation of the potential energy surface via a valley ridge inflection into two distinct intermediates: **IM1** (the “expected” inverse electron demand cycloadduct, which is then consumed by the cheletropic extrusion of SO_2_) and **IM2** (a normal electron demand cycloadduct with the furan acting as the 4π component).^20^ These intermediates are formally connected via a Cope rearrangement (**TS4**; Figure 3).

Such cycloaddition transition states were first envisioned by Woodward and Katz during their investigations on the dimerization of cyclopentadiene and were named “two-stage-one-step” processes.^21^ Caramella later revisited this phenomenon computationally and clarified the bifurcating nature of the transition state, as distinct from the Cope rearrangement pathway that connects the products.^22^ A number of other cycloadditions have since been reported that proceed through “bipericyclic” ambimodal pathways, in which two distinct products are formed from the bifurcation point.^23^

It is notable that the less-advanced bond formations in **TS3** (C3–C6, 2.67 Å and C2–C5, 2.93 Å) are highly asymmetric (Δ*r* = 0.26 Å). This suggests a product distribution favoring **IM2** over **IM1**,^24^ and analysis using Goodman’s *ValleyRidge* algorithm showed that **IM2** is indeed expected as the dynamic product.^25^ However, as this process is reversible (Δ*G*_rev_^0^ = 0.7 kcal mol^–1^, Δ*G*_rev_^‡^ = 28.4 kcal mol^–1^), the “required” cycloadduct **IM1** can be formed by recrossing from **IM2** via the starting materials.^24^ While the abovementioned Cope rearrangement pathway could also operate, the barrier is too high to contribute significantly to the rearrangement (**TS4**, 35.2 kcal mol^–1^). Following this conversion of **IM2** to **IM1**, the exergonic extrusion of SO_2_ to form the corresponding diene product was calculated to proceed with a barrier of 16.2 kcal mol^–1^ (Δ*G*^0^ = −15.1 kcal mol^–1^; see Table S4).^26^

**Figure 4:**
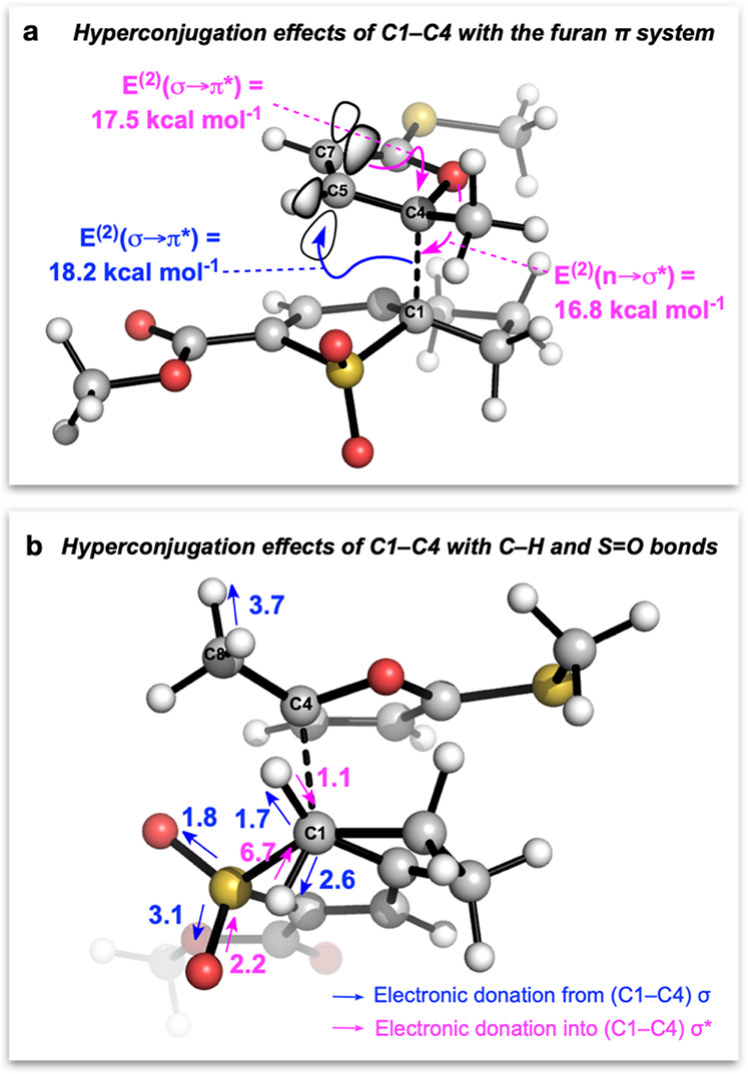
NBO second-order perturbation energy E^(2)^ analysis of **TS3** (Table S3). (a) Major contributions to stabilization and (b) minor hyperconjugation effects. Electron donation is from (blue) or into (pink) the developing C1–C4 bond. Calculations at the CPCM(toluene)-M06-2X/def2-TZVPP//CPCM (toluene)-M06-2X/def2-SVP level of theory at 373.15 K/1 M; all values are in kcal mol^–1^.

Distortion–interaction analysis revealed a higher distortion of the dienophile in **TS3** compared to the pericyclic transition state **TS1** (15 kcal mol^–1^ vs 6 kcal mol^–1^), which is offset by a significant increase in interaction energy (−21 kcal mol^–1^ vs −12 kcal mol^–1^ for **TS1**). This additional stabilization can be explained by electron donation from the developing C1–C4 σ bond into the C5–C7 π* orbital of the furan (Figure 4a; as evidenced by the NBO analysis, 18.2 kcal mol^–1^), accompanied by the expected donation into the developing C1–C4 σ* orbital from the furan π system (17.5 kcal mol^–1^) and oxygen lone pair (16.8 kcal mol^–1^). Several smaller C–H and S=O reciprocal hyperconjugation effects with the developing C1–C4 bond further stabilize **TS3** (Figure 4b). NBO population analysis also illustrates the asynchronicity of the process, with the forming C1–C4 bond (*r*_1_) being significantly populated (1.71 e^–^), while minimal population is observed for C3–C6 (*r*_2_) and C2–C5 (*r*_3_).^26^

**Figure 5:**
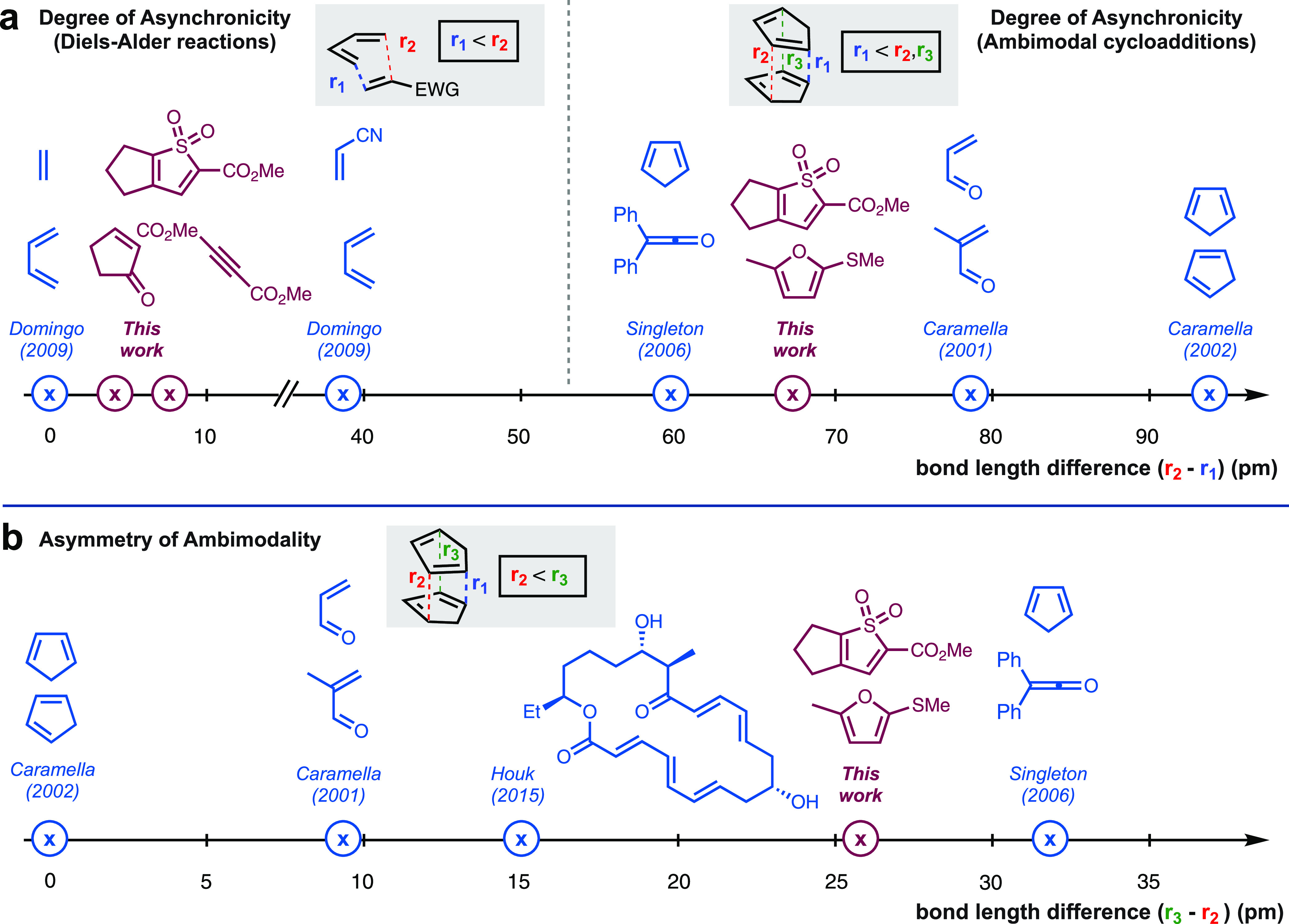
Context of thiophene *S,S*-dioxide cycloadditions in ambimodal processes (geometries taken from refs (16, 34)). (a) Degree of asynchronicity of Diels–Alder cycloadditions and degree of ambimodality of bifurcating cycloadditions, with respect to the difference in forming bond lengths *r*_1_ and *r*_2_. (b) Asynchronicity of ambimodality with respect to the difference in forming bond lengths *r*_2_ and *r*_3_. *r*_1_ is the advanced bond formation. *r*_1_ ≪ *r*_2_ and *r*_3_, and *r*_2_ ≤ *r*_3_.

The reactions of **16** with electron-deficient dienophiles are typical of nonpolar cycloadditions (Figure 5a, left, and **TS1**). Comparison of **TS3** with other ambimodal transition states^27^ reveals that it is moderately asynchronous with respect to *r*_1_ and *r*_2_ (Figure 5a, right) and highly asynchronous with respect to *r*_2_ and *r*_3_ (Figure 5b). These characteristics are similar to the reaction of diphenylketene with cyclopentadiene reported by Singleton,^23e^ with the “asymmetry of ambimodality” (*r*_3_ – *r*_2_; Figure 5b) lying toward the upper end of such transition states characterized to date. Indeed, comparison with other ambimodal processes appears to suggest a qualitative inverse correlation between the “degree” and “asymmetry” of ambimodality, with the dimerization of cyclopentadiene reported by Caramella^22a^ lying at the opposite end of this scale. It is interesting to consider whether these characteristics are critical or coincidental in enabling the reaction of **16** with an (aromatic) furan.

## Conclusions

In summary, thiophene *S,S-*dioxides can undergo (4 + 2) cycloadditions via several reactivity channels. In the context of the illudalane natural products, reactions with furans proceed via ambimodal transition states that bifurcate reversibly to discrete cycloadducts, while electron-deficient dienophiles react through classical nonpolar pericyclic pathways. This multifaceted intermolecular reactivity profile enables the synthesis of the illudalanes in as little as five steps and underlines the utility of thiophene *S,S-*dioxides as powerful synthetic tools for the concise assembly of polysubstituted aromatic rings.

## Computational Methods

All calculations were carried out using the ORCA suite of programs (version 4.2.1).^28^ Geometries were initially obtained via autodE using standard settings, with GFN2-XTB for conformational sampling and PBE-D3BJ/def2-SVP for geometry optimization.^29^ Dispersion corrections were considered using Grimme’s D3 empirical method with Becke–Johnson damping (D3BJ).^30^ The obtained geometries were then optimized at the CPCM(toluene)-M06-2X/def2-TZVPP level of theory. To support the computational validity of the M06-2X functional used herein, **TS3** was also optimized at the CPCM(toluene)-SCS-MP2/def2-TZVPP level of theory. Significant differences in the asynchronicity of the ambimodal **TS3** were found between two other DFT functionals and SCS-MP2; M06-2X was therefore used as it showed satisfactory agreement. A final CPCM(toluene)-DLPNO-CCSD(T)/def2-TZVPP single point calculation was performed on each structure to obtain reliable electronic energies (see the Supporting Information).^31^

Vibrational frequencies were computed at the optimization level of theory to confirm whether the structures correspond to minima or transition states. Grimme’s quasiRRHO approach was used to calculate free energies at 373.15 K.^32^ A standard state correction from 1 atm to 1 M was applied by adding *RT* ln(1/24.5) = 2.37 kcal mol^–1^ (*T* = 373.15 K) to the calculated free energy of each species. For calculating thermodynamic data, the python-script *OTherm.py* was used, with ω_0_ = 100 cm^–1^ replacing harmonic oscillators with free rotors below *ω*_0_.^32,33^

The 2D surface (More O’Ferrall–Jencks plot) in Figure 3 was generated in the space defined by the forming C1–C4 and C2–C5 bond distances using a grid of 0.1 Å. The area around stationary points **TS3** and **TS4** was sampled with a smaller grid size of 0.05 Å. These points were interpolated using the cubic spline function scipy.interp2d with matplotlib.py.

## Supporting Information Available

Experimental procedures and characterization data for novel compounds and computational details (PDF)
Coordinates (ZIP)
