# Supplementary material for: Collective Synthesis of Illudalane Sesquiterpenes via Cascade Inverse Electron Demand (4 + 2) Cycloadditions of Thiophene S,S-Dioxides
Source: J Am Chem Soc. 2022 May 24;144(22):10017–24. doi: 10.1021/jacs.2c03304 (PMC9185749; doi:10.1021/jacs.2c03304)
Supplement: Supplementary file 1 — ja2c03304_si_001.pdf [file ja2c03304_si_001.pdf]

Collective synthesis of illudalane sesquiterpenes *via* cascade inverse electron demand (4+2) cycloadditions of thiophene *S,S*-dioxides

Kun Ho (Kenny) Park, Nils Frank, Fernanda Duarte and Edward A. Anderson**

Chemistry Research Laboratory, 12 Mansfield Road, Oxford, OX1 3TA, UK

Supporting Information

1. General Experimental Considerations S2
2. Experimental Procedures and Physical Data S3
3. Copies of ^{1}H and ^{13}C NMR Spectra S26
4. Computational details S54
5. References S95

1. General Experimental Considerations

Solvents. Dichloromethane, tetrahydrofuran, *N,N*-dimethylformamide, and toluene were dried by passing through an activated alumina column under argon in a solvent dispenser. All other reagents were used as received. Brine refers to a saturated aqueous solution of NaCl. $NaHCO_3$, NH_4Cl and $Na_2S_2O_3$ solutions refer to saturated aqueous solutions. HCl was also used as an aqueous solution at the specified molarity.

Reactions. All reactions were carried out under argon or nitrogen unless otherwise stated. Oven-dried glassware was used for reactions requiring anhydrous conditions.

Chromatography. Thin-layer chromatography was performed on Merck aluminium-backed DC 60 F254 0.2 mm precoated plates, which were visualized with UV fluorescence and staining with potassium(VII) manganate or vanillin. Flash column chromatography was performed on MN Kieselgel 60M (particle size 40-63 μm), with the solvent system used in parentheses.

Infrared Spectroscopy. Infrared spectra were recorder on a Bruker Tensor 27 Fourier transform spectrometer, as a thin film on a diamond ATR module.

NMR Spectroscopy. ^{1}H NMR spectra were recorded at 400 or 500 MHz on Bruker AVIII HD 400 and AVII 500 spectrometers. ^{13}C NMR spectra were recorded at 101 MHz or 126 MHz on Bruker AVIII HD 400 and AVII 500 spectrometers. Chemical shifts (δ H and δ C) are expressed in parts per million (ppm), referenced to the residual solvent peak of $CDCl_3$. Coupling constants (*J*) are reported to the nearest 0.1 Hz. Spectra are assigned based on chemical shift, coupling constants, COSY, HSQC and HMBC data and / or comparison with similar compounds. Splitting patterns are described using the following abbreviations: s (singlet), d (doublet), t (triplet), q (quartet), quin. (quintet), sept. (septet).

Mass spectrometry. Low-resolution mass spectra (m/z) were performed on a Micromass LCT Premier Open Access. High resolution mass spectra were recorded under ESI or EI conditions on a Bruker MicroTOF.

Heating. For reactions that require heating, an oil bath was employed. The temperature was monitored via a temperature probe plugged into the stirrer plate.

2. Experimental Procedures and Physical Data

Methyl 5,5-dimethyl-5,6-dihydro-4H-cyclopenta[b]thiophene-2-carboxylate, 2

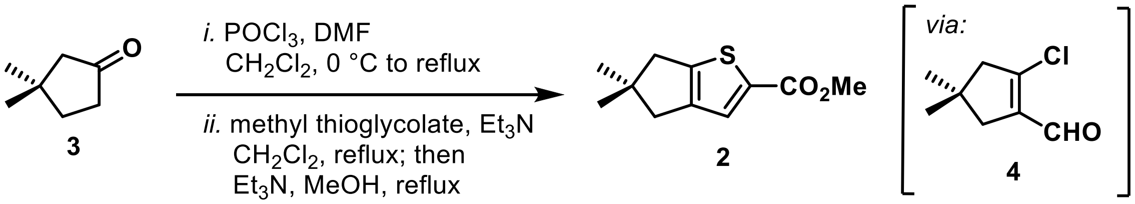

According to a modified literature procedure.[1] (i) To a stirred solution of anhydrous DMF (1.52 mL, 19.6 mmol, 1.6 equiv.) at 0 °C was added phosphorus oxychloride (1.34 mL, 14.3 mmol, 1.2 equiv.) dropwise. The resulting mixture was warmed to room temperature and stirred for 1 h before a solution of 3,3-dimethylcyclopentanone **3** (1.38 g, 12.3 mmol, 1.0 equiv.) in CH_2Cl_2 (6.0 mL) was added. The resulting mixture was warmed to reflux and stirred for 4 h before it was cooled to room temperature and poured into mixture of ice and NaOAc (3.00 g, 36.6 mmol, 3.0 equiv.). The layers were separated and the aqueous layer was extracted with CH_2Cl_2 (3 × 15 mL). The combined organic layers were washed with water (50 mL), brine (50 mL), dried (Na_2SO_4) and concentrated under reduced pressure to afford aldehyde **4** as a dark orange oil, which was used directly in the subsequent step without further purification. **4**: R_f = 0.70 (silica gel, pentane:Et_2O 4:1).

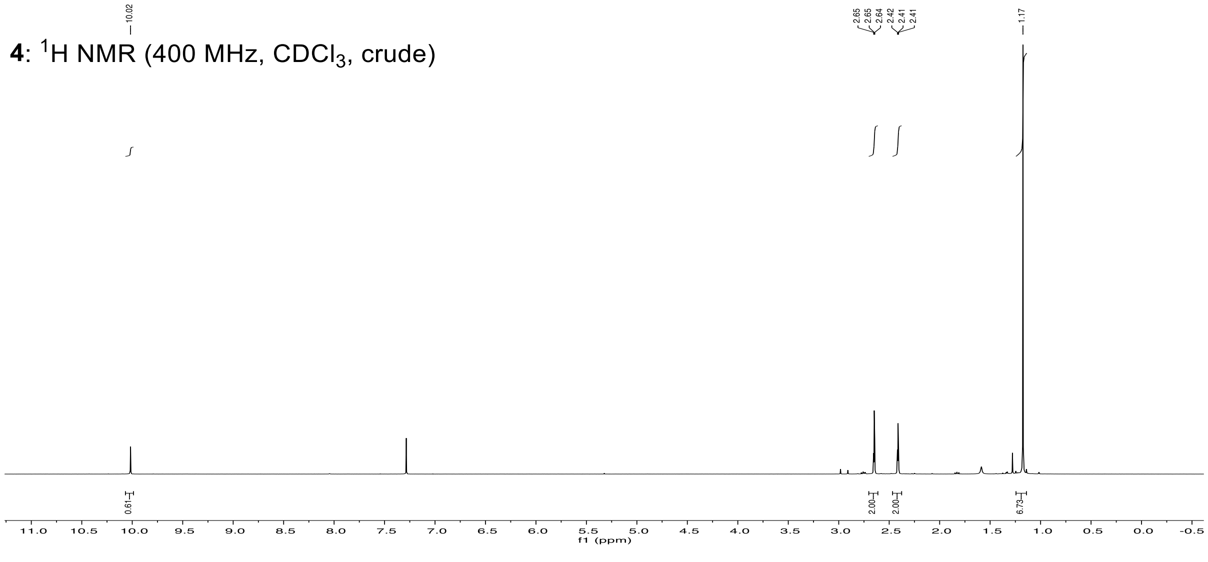

(ii) To a stirred solution of crude aldehyde **4** (1.34 g, 8.45 mmol, 1.0 equiv.) in CH_2Cl_2 (5.0 mL) at room temperature was added methyl thioglycolate (0.80 mL, 8.95 mmol, 1.1 equiv.) and Et_3N (3.60 mL, 25.8 mmol, 3.1 equiv.). The mixture was warmed to reflux and stirred for 16 h before it was cooled to room temperature and concentrated under reduced pressure. The residue was taken up in MeOH (5.0 mL), and Et_3N (3.60 mL, 25.8 mmol, 3.1 equiv.) was added. The resulting mixture was warmed to reflux and stirred for 16 h before it was cooled to room temperature and concentrated under reduced pressure. Flash column chromatography (silica gel, pentane:Et_2O 20:1→6:1) afforded the title compound **2** (1.42 g, 6.75 mmol,

55% over two steps) as an orange waxy solid. **2**: R_f = 0.60 (silica gel, pentane:Et_2O 4:1); IR (film) ν_{max} 2953, 1708, 1439, 1281, 752 cm^{-1}; ^1H NMR (400 MHz, $CDCl_3$): δ 7.47 (s, 1H), 3.82 (s, 3H), 2.70 (s, 2H), 2.54 (s, 2H), 1.21 (s, 6H); ^{13}C NMR (101 MHz, $CDCl_3$): δ 162.8, 149.4, 145.5, 134.8, 129.3, 51.7, 45.8, 44.3, 43.3, 29.6 (2C); HRMS calcd. For $C_{11}H_{15}O_2S^+$ $[M + H]^+$ 211.0787, found 211.0788.

Methyl 5,5-dimethyl-5,6-dihydro-4H-cyclopenta[b]thiophene-2-carboxylate 1,1-dioxide, 1

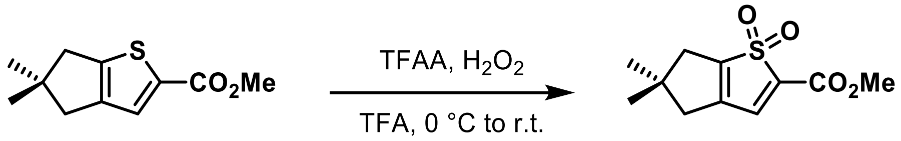

2 1

According to a modified literature procedure.[2] To a stirred solution of trifluoroacetic anhydride (4.80 mL, 34.5 mmol, 9.9 equiv.) and trifluoroacetic acid (2.0 mL) at 0 °C was added H_2O_2 (1.40 mL, 13.7 mmol, 3.9 equiv., aq. 30%) dropwise. The resulting mixture was warmed to room temperature and stirred for 15 min. To the resulting mixture at 0 °C was added a solution of **2** (0.73 g, 3.47 mmol, 1.0 equiv.) in TFA (5.0 mL) dropwise. The resulting mixture was warmed to room temperature and stirred for 14 h before it was concentrated under reduced pressure. Flash column chromatography (silica gel, pentane:EtOAc 10:1→1:1) afforded the title compound **1** (0.61 g, 2.52 mmol, 73%) as a white waxy solid. **1**: R_f = 0.43 (silica gel, pentane:EtOAc 4:1); IR (film) ν_{max} 2958, 1722, 1566, 1435, 1314, 752 cm^{-1}; ^1H NMR (400 MHz, $CDCl_3$): δ 7.41 (s, 1H), 3.87 (s, 3H), 2.53 (t, J = 2.5 Hz, 2H), 2.43 (t, J = 2.9 Hz, 2H), 1.21 (s, 6H); ^{13}C NMR (101 MHz, $CDCl_3$): δ 158.2, 144.8, 143.6, 138.4, 135.5, 52.8, 44.6, 43.9, 40.9, 29.5 (2C); HRMS calcd. For $C_{11}H_{15}O_4S^+$ $[M + H]^+$ 243.0686, found 243.0688.

Methyl 2,2,7-trimethyl-6-(2-(methylthio)-2-oxoethyl)-2,3-dihydro-1H-indene-5-carboxylate, 6

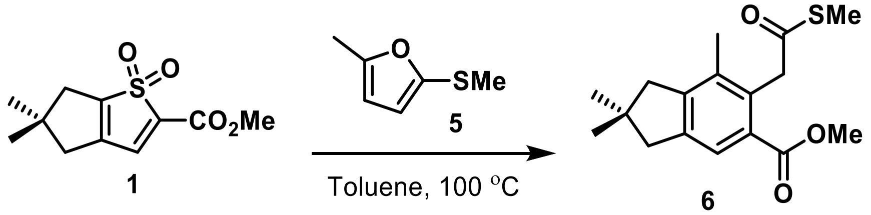

1 6

To a stirred solution of **1** (0.30 g, 1.24 mmol, 1.0 equiv.) in toluene (4.0 mL) at room temperature was added 2-methyl-5-(methylthio)furan **5** (0.60 mL, 4.98 mmol, 4.0 equiv.). The resulting mixture was warmed to 100 °C and stirred for 20 h before it was cooled to room temperature and concentrated under reduced pressure. Flash column chromatography (silica gel, pentane:Et_2O 20:1→8:1) afforded the title compound **6** (0.24 g, 0.78 mmol, 63%) as a yellow oil. **6**: R_f = 0.48 (silica gel, pentane:Et_2O 5:1); IR (film) ν_{max} 2952, 1716, 1694, 1433, 1301, 999 cm^{-1}; ^1H NMR (400 MHz, $CDCl_3$): δ 7.63 (s, 1H), 4.32 (s, 2H), 3.85 (s, 3H), 2.76 (s, 2H), 2.72 (s, 2H), 2.27 (s, 3H), 2.20 (s, 3H), 1.15 (s, 6H); ^{13}C NMR (101 MHz, $CDCl_3$): δ 198.0, 168.4, 147.7, 142.4, 134.9, 131.3, 129.0, 124.5, 51.9, 47.7, 47.5, 44.5, 39.6, 28.9 (2C), 16.3, 11.6; HRMS calcd. For $C_{17}H_{22}O_3SNa^+$ $[M + Na]^+$ 329.1182, found 329.1182.

Riparol B

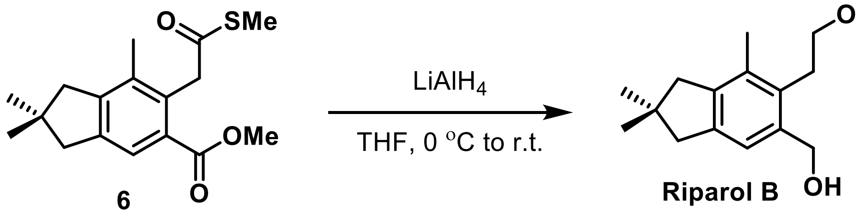

To a stirred solution of **6** (0.31 g, 1.01 mmol, 1.0 equiv.) in THF (5.0 mL) at 0 °C was added $LiAlH_4$ (0.16 g, 4.22 mmol, 4.2 equiv.). The resulting mixture was warmed to room temperature and stirred for 2 h before it was cooled to 0 °C and quenched with sodium potassium tartrate (5 mL, sat. aq.), diluted with EtOAc (10 mL) and stirred vigorously for 1 h. The layers were separated and the aqueous layer was extracted with EtOAc (3 × 10 mL), the combined organic layer was washed with water (30 mL), brine (30 mL), dried (Na_2SO_4) and concentrated under reduced pressure. Flash column chromatography (silica gel, pentane:EtOAc 1:2) afforded **riparol B** (0.20 g, 0.85 mmol, 84%) as a white solid. **riparol B**: R_f = 0.35 (silica gel, pentane:EtOAc 1:3); m.p.: 114–116 °C; IR (film) ν_{max} 3310, 2952, 2924, 1464, 1437, 1379, 1364, 1040 cm^{-1}; ^1H NMR (400 MHz, $CDCl_3$): δ 7.01 (s, 1H), 4.61 (s, 2H), 3.86 (t, J = 6.1 Hz, 2H), 3.02 (t, J = 6.0 Hz, 2H), 2.72 (s, 2H), 2.68 (s, 2H), 2.20 (s, 3H), 1.15 (s, 6H); ^{13}C NMR (101 MHz, $CDCl_3$): δ 143.4, 141.5, 137.9, 133.3, 133.1, 124.0, 64.2, 61.6, 47.9, 47.3, 39.3, 31.7, 29.2 (2C), 16.0; HRMS calcd. For $C_{15}H_{22}O_2Na^+$ $[M + Na]^+$ 257.1512, found 257.1522.

^1H and ^{13}C NMR ($CDCl_3$, ppm) comparison for riparol B

Zhang[5] (^1H, 400 MHz, ppm)	This work (^1H, 400 MHz, ppm)	Zhang[5] (^{13}C, 100 MHz, ppm)	This work (^{13}C, 101 MHz, ppm)
6.99 (s, 1H)	7.01 (s, 1H)	143.4	143.4
4.58 (s, 2H)	4.61 (s, 2H)	141.5	141.5
3.82 (t, J = 5.9 Hz, 2H)	3.86 (t, J = 6.1 Hz, 2H)	137.8	137.9
3.40 (s, 2H)	3.02 (t, J = 6.0 Hz, 2H)	133.4	133.3
2.99 (t, J = 5.9 Hz, 2H)	2.72 (s, 2H)	133.0	133.1
2.72 (s, 2H)	2.68 (s, 2H)	124.0	124.0
2.67 (s, 2H)	2.20 (s, 3H)	64.2	64.2
2.19 (s, 3H)	1.15 (s, 6H)	61.5	61.6
1.15 (s, 3H)		47.8	47.9
1.15 (s, 3H)		47.3	47.3
		39.3	39.3
		31.6	31.7
		29.2	29.2
		29.2	29.2
		16.0	16.0

Granulolactone

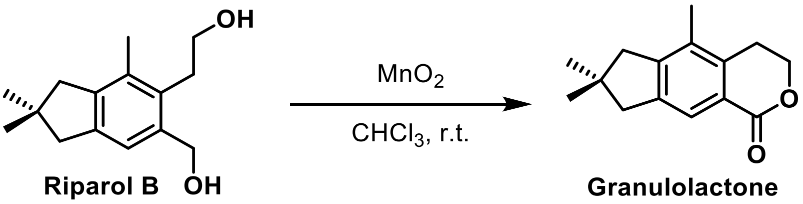

According to a modified literature procedure.[3] To a stirred solution of **riparol B** (25.6 mg, 0.11 mmol, 1.0 equiv.) in $CHCl_3$ (1.5 mL) at room temperature was added activated MnO_2 (0.25 g, 2.88 mmol, 26 equiv.) and stirred for 14 h before it was filtered through Celite,® eluted with CH_2Cl_2 (3 × 3 mL) and concentrated under reduced pressure. Flash column chromatography (silica gel, pentane:EtOAc 6:1) afforded **granulolactone** (23.1 mg, 0.10 mmol, 92%) as a white solid. **Granulolactone**: R_f = 0.55 (silica gel, pentane:EtOAc 3:1); m.p.: 118–120 °C; IR (film) ν_{max} 2952, 2927, 1718, 1610, 1466, 1447, 1395, 1334, 1286, 1187, 1159, 1088, 786 cm^{-1}; ^{1}H NMR (400 MHz, $CDCl_3$): δ 7.79 (s, 1H), 4.48 (t, J = 6.0 Hz, 2H), 2.94 (t, J = 6.0 Hz, 2H), 2.76 (s, 2H), 2.71 (s, 2H), 2.18 (s, 3H), 1.16 (s, 6H); ^{13}C NMR (101 MHz, $CDCl_3$): δ 166.2, 149.3, 142.4, 136.2, 130.7, 124.1, 123.5, 66.7, 47.5, 47.2, 39.7, 28.9 (2C), 25.1, 15.3; HRMS calcd. For $C_{15}H_{18}O_2Na^+$ $[M + Na]^+$ 253.1199, found 253.1199.

^{1}H and ^{13}C NMR ($CDCl_3$, ppm) comparison for granulolactone

Zhang[5] (^{1}H, 400 MHz, ppm)	This work (^{1}H, 400 MHz, ppm)	Zhang[5] (^{13}C, 100 MHz, ppm)	This work (^{13}C, 101 MHz, ppm)
7.79 (s, 1H)	7.79 (s, 1H)	166.2	166.2
4.49 (t, J = 6.0 Hz, 2H)	4.48 (t, J = 6.0 Hz, 2H)	149.3	149.3
2.94 (t, J = 6.0 Hz, 2H)	2.94 (t, J = 6.0 Hz, 2H)	142.4	142.4
2.77 (s, 2H)	2.76 (s, 2H)	136.1	136.2
2.72 (s, 2H)	2.71 (s, 2H)	130.7	130.7
2.18 (s, 3H)	2.18 (s, 3H)	124.1	124.1
1.16 (s, 3H)	1.16 (s, 6H)	123.5	123.5
1.16 (s, 3H)		66.7	66.7
		47.5	47.5
		47.2	47.2
		39.6	39.7
		28.8	28.9
		28.8	28.9
		25.1	25.1
		15.2	15.3

Echinolactone A + S1

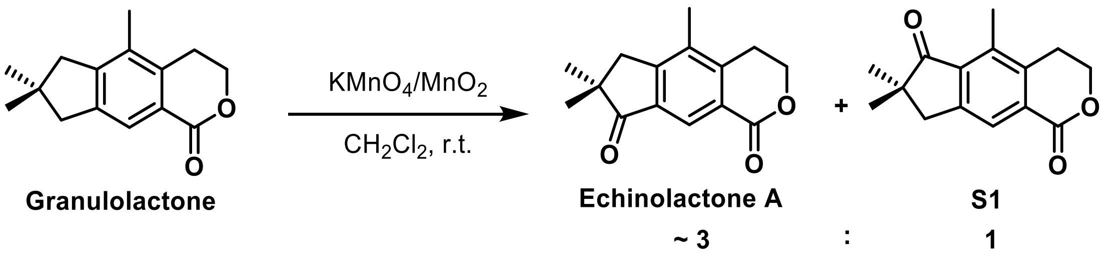

According to a modified literature procedure.[4] To a stirred solution of **granulolactone** (38.0 mg, 0.16 mmol) in CH_2Cl_2 (1.5 mL) at room temperature was added $KMnO_4/MnO_2$ (0.10 g; the oxidant was prepared by grinding potassium permanganate and activated manganese dioxide [1:3 ratio by mass] in a mortar until a homogeneous powder was obtained).[4] The resulting mixture was stirred for 48 h before additional oxidant (0.10 g) was added. The resulting mixture was furthered stirred for 72 h before it was filtered through Celite,® eluted with CH_2Cl_2 (3 × 3 mL) and concentrated under reduced pressure. Flash column chromatography (silica gel, pentane:EtOAc 12:1→3:1) afforded **echinolactone A** (23.7 mg, 97 µmol, 71% brsm, 59%) as a white solid, **S1** (7.4 mg, 30 µmol, 22% brsm, 18%) as a white sticky solid and recovered **granulolactone** (6.5 mg, 28 µmol).

Echinolactone A: R_f = 0.23 (silica gel, pentane:EtOAc 1:1); m.p.: 114–116 °C; IR (film) ν_{max} 2927, 1721, 1605, 1465, 1392, 1291, 1265, 1211, 1173, 1090, 790 cm^{-1}; ^1H NMR (400 MHz, $CDCl_3$): δ 8.42 (s, 1H), 4.54 (t, J = 6.0 Hz, 2H), 3.08 (t, J = 6.0 Hz, 2H), 2.96 (s, 2H), 2.30 (s, 3H), 1.26 (s, 6H); ^{13}C NMR (101 MHz, $CDCl_3$): δ 210.0, 164.8, 155.7, 143.8, 134.4, 132.9, 125.5, 125.1, 66.2, 45.6, 42.5, 25.8, 25.3 (2C), 14.2; HRMS calcd. For $C_{15}H_{17}O_3^+$ $[M + H]^+$ 245.1172, found 245.1173.

S1: R_f = 0.48 (silica gel, pentane:EtOAc 1:1); IR (film) ν_{max} 2921, 1709, 1606, 1452, 1350, 1296, 1260, 1197, 1157, 1090, 768 cm^{-1}; ^1H NMR (500 MHz, $CDCl_3$): δ 8.04 (s, 1H), 4.55 (t, J = 6.0 Hz, 2H), 3.04 (t, J = 6.0 Hz, 2H), 2.98 (s, 2H), 2.65 (s, 3H), 1.23 (s, 6H); ^{13}C NMR (126 MHz, $CDCl_3$): δ 212.3, 165.1, 150.8, 137.6, 136.6, 136.1, 129.7, 126.1, 66.8, 46.2, 41.9, 25.4 (2C), 24.5, 13.2; HRMS calcd. For $C_{15}H_{17}O_3^+$ $[M + H]^+$ 245.1172, found 245.1174.

^{1}H and ^{13}C NMR ($CDCl_3$, ppm) comparison for echinolactone A

Zhang[5] (^{1}H, 400 MHz, ppm)	This work (^{1}H, 400 MHz, ppm)	Zhang[5] (^{13}C, 100 MHz, ppm)	This work (^{13}C, 101 MHz, ppm)
8.41 (s, 1H)	8.42 (s, 1H)	210.1	210.0
4.53 (t, *J* = 5.9 Hz, 2H)	4.54 (t, *J* = 6.0 Hz, 2H)	164.8	164.8
3.08 (t, *J* = 5.9 Hz, 2H)	3.08 (t, *J* = 6.0 Hz, 2H)	155.8	155.7
2.96 (s, 2H)	2.96 (s, 2H)	143.9	143.8
2.30 (s, 3H)	2.30 (s, 3H)	134.3	134.4
1.25 (s, 3H)	1.26 (s, 6H)	133.1	132.9
1.25 (s, 3H)		125.4	125.5
		124.9	125.1
		66.3	66.2
		45.6	45.6
		42.5	42.5
		25.8	25.8
		25.3	25.3
		25.3	25.3
		14.2	14.2

Radulactone

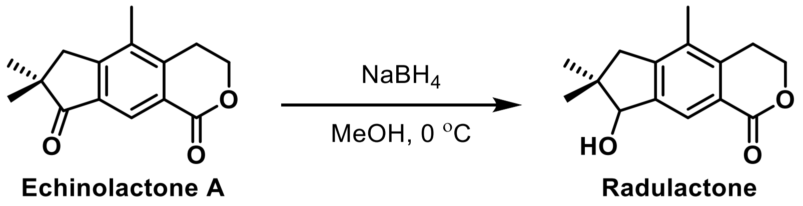

According to a modified literature procedure.[5] To a stirred solution of **echinolactone A** (17.0 mg, 70 μmol, 1.0 equiv.) in MeOH (1.5 mL) at 0 °C was added $NaBH_4$ (10.0 mg, 0.26 mmol, 3.7 equiv.). The resulting mixture was stirred for 1 h before it was diluted with brine (3 mL) and CH_2Cl_2 (3 mL). The layers were separated and the aqueous layer was extracted with CH_2Cl_2 (3 × 3 mL), the combined organic layer was washed with water (10 mL), brine (10 mL), dried (Na_2SO_4) and concentrated under reduced pressure. Flash column chromatography (silica gel, pentane:EtOAc 3:1) afforded **radulactone** (15.8 mg, 64 μmol, 92%) as a clear semi-solid. **Radulactone**: R_f = 0.30 (silica gel, pentane:EtOAc 1:1); IR (film) ν_{max} 3300, 2950, 2920, 1714, 1611, 1468, 1293, 1204, 1170, 1087, 788 cm^{-1}; ^1H NMR (500 MHz, $CDCl_3$): δ 8.00 (s, 1H), 4.70 (s, 1H), 4.50 (t, J = 6.0 Hz, 2H), 2.97 (t, J = 6.0 Hz, 2H), 2.81 (d, J = 16.3 Hz, 1H), 2.63 (d, J = 16.3 Hz, 1H), 2.20 (s, 3H), 1.18 (s, 3H), 1.06 (s, 3H); ^{13}C NMR (126 MHz, $CDCl_3$): δ 165.8, 147.4, 143.7, 138.2, 131.3, 124.2 (2C), 83.0, 66.6, 44.2, 44.2, 26.8, 25.2, 21.5, 15.0; HRMS calcd. For $C_{15}H_{18}O_3Na^+$ $[M + Na]^+$ 269.1148, found 269.1149.

^1H and ^{13}C NMR ($CDCl_3$, ppm) comparison for radulactone

Zhang[5] (^1H, 400 MHz, ppm)	This work (^1H, 500 MHz, ppm)	Zhang[5] (^{13}C, 100 MHz, ppm)	This work (^{13}C, 126 MHz, ppm)
7.99 (s, 1H)	8.00 (s, 1H)	165.9	165.8
4.69 (s, 1H)	4.70 (s, 1H)	147.4	147.4
4.48 (t, J = 5.9 Hz, 2H)	4.50 (t, J = 6.0 Hz, 2H)	143.8	143.7
2.96 (t, J = 5.9 Hz, 2H)	2.97 (t, J = 6.0 Hz, 2H)	138.1	138.2
2.79 (d, J = 16.3 Hz, 1H)	2.81 (d, J = 16.3 Hz, 1H)	131.2	131.3
2.61 (d, J = 16.3 Hz, 1H)	2.63 (d, J = 16.3 Hz, 1H)	124.2	124.2
2.19 (s, 3H)	2.20 (s, 3H)	124.1	124.2
2.13 (brs, 1H)	1.18 (s, 3H)	82.9	83.0
1.17 (s, 3H)	1.06 (s, 3H)	66.6	66.6
1.05 (s, 3H)		44.2	44.2
		44.1	44.2
		26.8	26.8
		25.2	25.2
		21.5	21.5
		14.9	15.0

2-(6-(Hydroxymethyl)-2,2-dimethyl-2,3-dihydro-1H-inden-5-yl)ethan-1-ol, 9

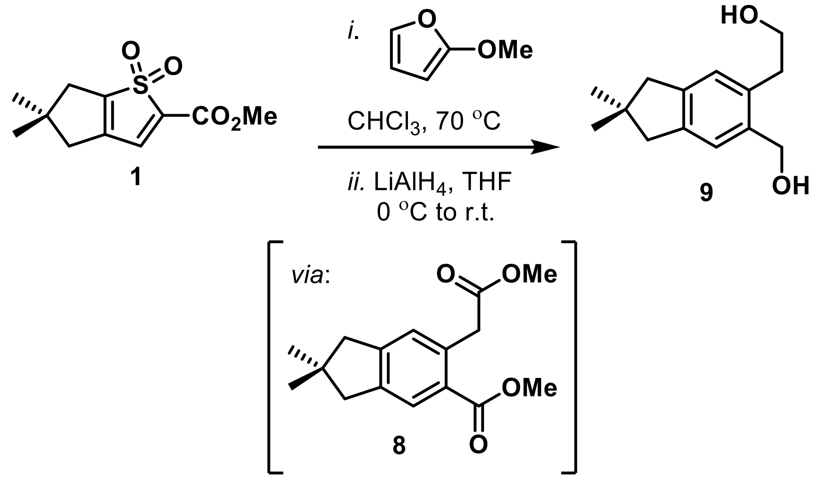

(i) To a stirred solution of **1** (94.8 mg, 0.39 mmol, 1.0 equiv.) in $CHCl_3$ (3.0 mL) at room temperature was added 2-methoxyfuran (0.14 mL, 1.52 mmol, 3.9 equiv.). The resulting mixture was warmed to 70 °C and stirred for 20 h before it was cooled to room temperature and concentrated under reduced pressure. The resulting residue was filtered through silica gel (pentane:Et_2O 12:1) which afforded compound **8** (55.0 mg, 0.20 mmol, 51%) as a yellow oil.

(ii) To a stirred solution of **8** (53.2 mg, 0.19 mmol, 1.0 equiv.) in THF (3.0 mL) at 0 °C was added $LiAlH_4$ (28.8 mg, 0.76 mmol, 4.0 equiv.). The resulting mixture was warmed to room temperature and stirred for 2 h before it was cooled to 0 °C and quenched with sodium potassium tartrate (5 mL, sat. aq.), diluted with EtOAc (5 mL) and stirred vigorously for 1 h. The layers were separated and the aqueous layer was extracted with EtOAc (3 × 8 mL), the combined organic layer was washed with water (15 mL), brine (15 mL), dried (Na_2SO_4) and concentrated under reduced pressure. Flash column chromatography (silica gel, pentane:EtOAc 1:2) afforded the title compound **9** (36.0 mg, 0.16 mmol, 85%) as a yellow sticky solid.

9: R_f = 0.33 (silica gel, pentane:EtOAc 1:3); IR (film) ν_{max} 3309, 2981, 2924, 1463, 1434, 1382, 1365, 1066 cm^{-1}; ^1H NMR (400 MHz, $CDCl_3$): δ 7.10 (s, 1H), 7.02 (s, 1H), 4.56 (s, 2H), 3.83 (t, J = 5.8 Hz, 2H), 2.88 (t, J = 5.9 Hz, 2H), 2.69 (s, 3H), 2.68 (s, 3H), 1.14 (s, 6H); ^{13}C NMR (101 MHz, $CDCl_3$): δ 144.2, 142.1, 137.1, 135.9, 126.3, 126.2, 63.6, 63.3, 47.5, 47.3, 40.2, 35.0, 28.8 (2C); HRMS calcd. For $C_{14}H_{20}O_2Na^+$ $[M + Na]^+$ 243.1356, found 243.1357.

Methyl (E)-3-(5-(2-hydroxyethyl)-2,2,6-trimethyl-2,3-dihydro-1H-inden-4-yl)acrylate 11

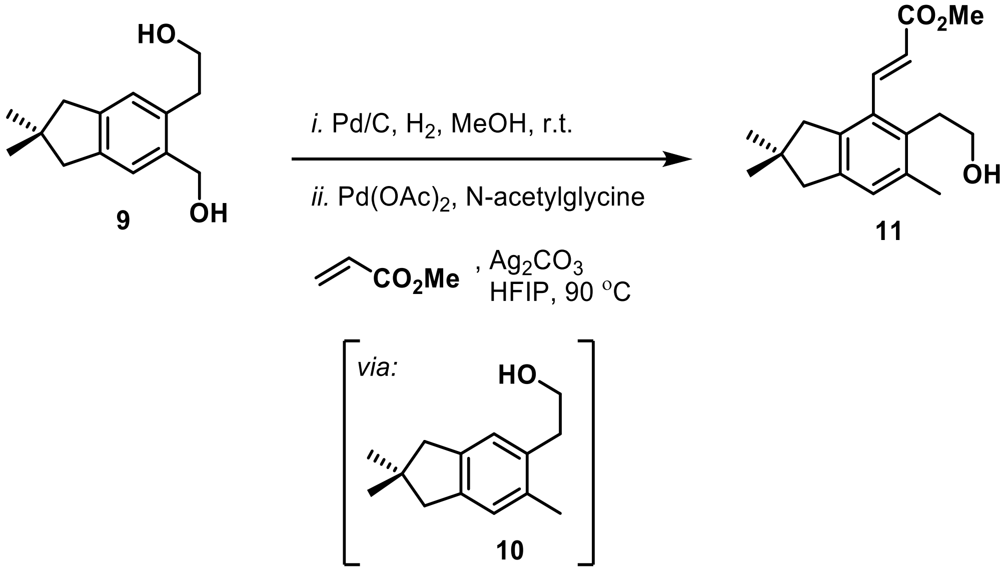

(i) To a stirred solution of **9** (36.0 mg, 0.16 mmol, 1.0 equiv.) in MeOH (2.0 mL) at room temperature was added Pd/C (10% wt/wt, 17.4 mg, 16 μmol, 0.1 equiv.). The resulting mixture was evacuated and filled with hydrogen (3 ×) and stirred under an atmosphere of H_2 (balloon) for 14 h. The resulting mixture was filtered through Celite®, eluted with CH_2Cl_2 (3 × 2 mL) and concentrated under reduced pressure. Flash column chromatography (silica gel, pentane:EtOAc 4:1) afforded the title compound **10** (25.0 mg, 0.12 mmol, 75%) as a clear oil.

(ii) *According to a modified literature procedure.*[6] To a stirred solution of **10** (25.0 mg, 0.12 mmol, 1.0 equiv.) in HFIP (1.2 mL) at room temperature was added Ag_2CO_3 (50.6 mg, 0.18 mmol, 1.5 equiv.), N-acetylglycine (2.9 mg, 25 μmol, 0.2 equiv.), methyl acrylate (22 μL, 0.24 mmol, 2.0 equiv.) and $Pd(OAc)_2$ (2.7 mg, 12 μmol, 0.1 equiv.). The resulting mixture was warmed to 90 °C and stirred for 36 h before it was cooled to room temperature and concentrated under reduced pressure. Flash column chromatography (silica gel, pentane:EtOAc 3:1) afforded the title compound **11** (20.5 mg, 71 μmol, 58%) as a clear oil.

11: R_f = 0.59 (silica gel, pentane:EtOAc 1:2); IR (film) ν_{max} 2956, 2940, 1716, 1624, 1460, 1437, 1310, 1167, 1044 cm^{-1}; ^1H NMR (400 MHz, $CDCl_3$): δ 8.00 (d, J = 16.1 Hz, 1H), 7.02 (s, 1H), 6.11 (d, J = 16.2 Hz, 1H), 3.82 (s, 3H), 3.73 (t, J = 7.4 Hz, 2H), 3.02 (t, J = 7.4 Hz, 2H), 2.78 (s, 2H), 2.69 (s, 2H), 2.35 (s, 3H), 1.12 (s, 6H); ^{13}C NMR (101 MHz, $CDCl_3$): δ 167.5, 143.4, 142.7, 140.5, 135.4, 133.3, 131.4, 127.9, 122.5, 62.4, 51.7, 48.8, 47.4, 40.0, 33.0, 28.7 (2C), 20.3; HRMS calcd. For $C_{18}H_{24}O_3Na^+$ $[M + Na]^+$ 311.1618, found 311.1618.

5-(2-((tert-butyldimethylsilyl)oxy)ethyl)-2,2,6-trimethyl-2,3-dihydro-1H-indene-4-carbaldehyde S3

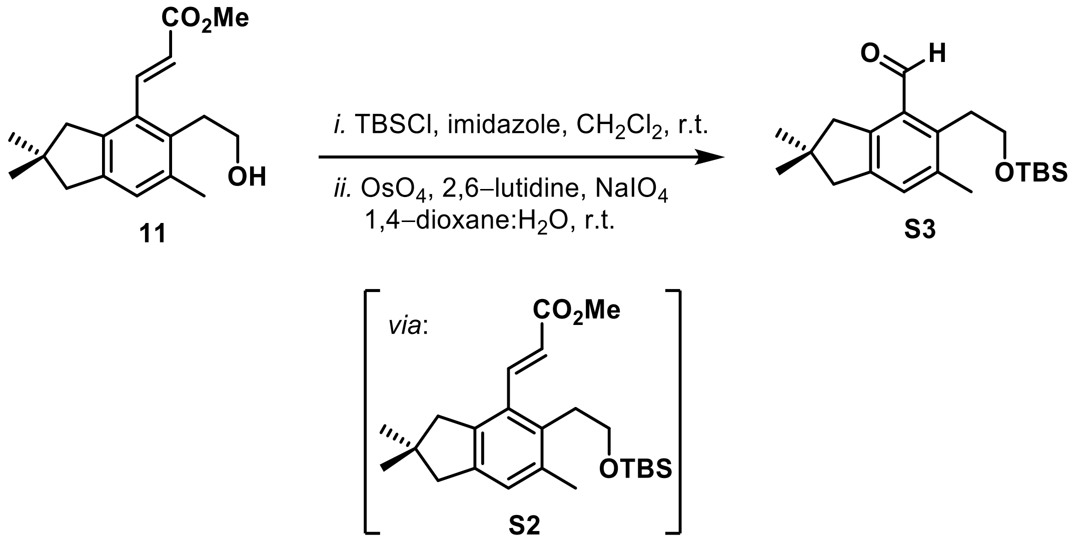

(i) To a stirred solution of **11** (20.0 mg, 69 μmol, 1.0 equiv.) in CH_2Cl_2 (1.0 mL) at room temperature was added TBSCl (12.6 mg, 84 μmol, 1.2 equiv.) and imidazole (7.0 mg, 0.10 mmol, 1.5 equiv.). The resulting mixture was stirred for 24 h before it was diluted with H_2O (2 mL) and CH_2Cl_2 (2 mL). The layers were separated and the aqueous layer was extracted with CH_2Cl_2 (3 × 3 mL), the combined organic layer was washed with water (10 mL), brine (10 mL), dried (Na_2SO_4) and concentrated under reduced pressure. Flash column chromatography (silica gel, pentane: Et_2O 3:1) afforded the title compound **S2** (25.8 mg, 64 μmol, 92%) as a clear oil.

(ii) To a stirred solution of **S2** (25.8 mg, 64 μmol, 1.0 equiv.) in 1,4-dioxane/H_2O (3:1, 4.0 mL) at room temperature were added 2,6-lutidine (15 μL, 0.13 mmol, 2.0 equiv.), osmium tetroxide (4 wt.% in MeOH, 50 μL, 7 μmol, 0.1 equiv.) and sodium periodate (27.4 mg, 0.13 mmol, 2.0 equiv.). The resulting mixture was stirred for 60 h before it was quenched with sodium thiosulfate (3 mL, sat. aq.). The layers were separated and the aqueous layer was extracted with Et_2O (3 × 3 mL), the combined organic layer was washed with brine (15 mL), dried over anhydrous Na_2SO_4 and concentrated under reduced pressure. The resulting residue was purified by flash column chromatography (silica gel, pentane:Et_2O 30:1) afforded the title compound **S3** (19.1 mg, 55 μmol, 86 %) as a clear oil. **S3**: R_f = 0.57 (silica gel, pentane: Et_2O 16:1); IR (film) ν_{max} 2956, 2940, 1727, 1624, 1474, 1419, 1192, 1096, 710 cm^{-1}; ^1H NMR (500 MHz, $CDCl_3$): δ 10.50 (s, 1H), 7.20 (s, 1H), 3.78 (t, J = 7.0 Hz, 2H), 3.24 (t, J = 7.0 Hz, 2H), 3.04 (s, 2H), 2.66 (s, 2H), 2.36 (s, 3H), 1.14 (s, 6H), 0.83 (s, 9H), -0.06 (s, 6H); ^{13}C NMR (101 MHz, $CDCl_3$): δ 193.5, 144.4, 143.1, 137.6, 135.9, 132.0, 131.3, 63.6, 47.7, 46.7, 40.0, 31.1, 28.9 (2C), 25.9 (3C), 19.9, 18.3, -5.5 (2C); HRMS calcd. For $C_{21}H_{34}O_2SiNa^+$ $[M + Na]^+$ 369.2220, found 369.2221.

Alcyopterosin O

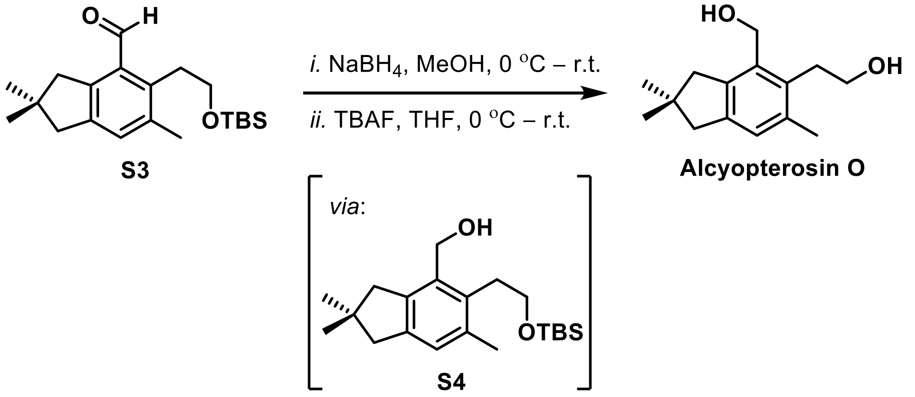

(i) To a stirred solution of **S3** (19.1 mg, 55 µmol, 1.0 equiv.) in MeOH (1.0 mL) at 0 °C was added $NaBH_4$ (10.0 mg, 0.26 mmol, 4.7 equiv.). The resulting mixture was stirred for 1 h before it was concentrated under reduced pressure. The mixture was diluted with brine (2 mL) and CH_2Cl_2 (2 mL). The layers were separated and the aqueous layer was extracted with CH_2Cl_2 (3 × 3 mL), the combined organic layer was washed with water (10 mL), brine (10 mL), dried (Na_2SO_4), concentrated under reduced pressure and filtered through short pad of silica and eluded with pentane: Et_2O 2:1 to afford alcohol **S4** (18.1 mg, 52 µmol, 94%) as a clear oil. **S4**: R_f = 0.28 (silica gel, pentane:Et_2O 4:1).

S4: ^{1}H NMR (400 MHz, $CDCl_3$, filtered through silica)

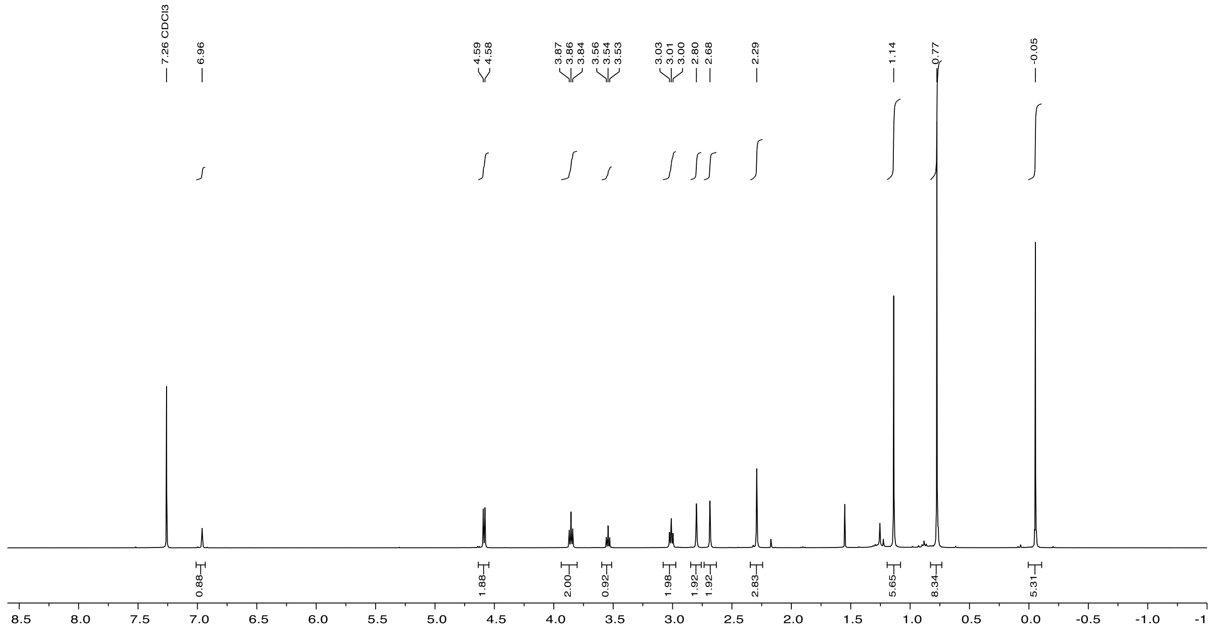

(ii) To a stirred solution of **S4** (18.1 mg, 52 µmol, 1.0 equiv.) in THF (1.0 mL) at 0 °C was added TBAF (1M in THF, 0.15 mL, 0.15 mmol, 2.9 equiv.). The resulting mixture was stirred for 1 h before it was diluted with H_2O (1 mL) and EtOAc (1 mL). The layers were separated and the aqueous layer was extracted with EtOAc (3 × 2 mL), the combined organic layer was washed with water (8 mL), brine (8 mL), dried (Na_2SO_4) and concentrated under reduced pressure. Flash column chromatography (silica gel, pentane:EtOAc 2:1→1:5) afforded **alcyopterosin O** (10.7 mg, 46 µmol, 88%) as a white semi-solid. Characterization data match that reported from the alternative sulfone pathway shown below.

Ethyl 3-((2-ethoxy-2-oxoethyl)thio)-5,5-dimethyl-5,6-dihydro-4H cyclopenta[c]thiophene-1-carboxylate, 13

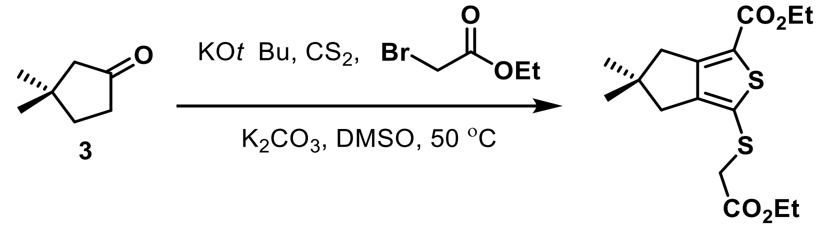

3 13

According to a modified literature procedure.[7] To a stirred solution of potassium tert-butoxide (1.74 g, 15.5 mmol, 2.1 equiv.) in DMSO (20.0 mL) at room temperature was added 3,3-dimethylcyclopentanone **3** (0.83 g, 7.40 mmol, 1.0 equiv.) dropwise. The resulting mixture was stirred for 0.5 h before CS_2 (0.47 mL, 7.78 mmol, 1.1 equiv.) was added. The resulting mixture was warmed to 50 °C and stirred for 1 h before it was cooled to room temperature and ethyl bromoacetate (1.68 mL, 15.2 mmol, 2.1 equiv.) was added. The resulting mixture was stirred for 14 h before K_2CO_3 (1.03 g, 7.45 mmol, 1.0 equiv.) was added. The resulting mixture was warmed to 50 °C and stirred for 3 h before it was cooled to room temperature and diluted with water (20 mL). The layers were separated and the aqueous layer was extracted with EtOAc (3 × 15 mL), the combined organic layer was washed with water (40 mL), brine (2 × 40 mL), dried (Na_2SO_4) and concentrated under reduced pressure. Flash column chromatography (silica gel, pentane:Et_2O 12:1→5:1) afforded the title compound **13** (1.06 g, 3.10 mmol, 42%) as a yellow semi-solid.

13: R_f = 0.35 (silica gel, pentane: Et_2O 5:1); IR (film) ν_{max} 2955, 1736, 1705, 1477, 1368, 1276, 1115, 760 cm^{-1}; ^{1}H NMR (400 MHz, $CDCl_3$): δ 4.27 (q, J = 7.1 Hz, 2H), 4.16 (q, J = 7.1 Hz, 2H), 3.51 (s, 2H), 2.74 (s, 2H), 2.49 (s, 2H), 1.32 (t, J = 7.1 Hz, 3H), 1.24 (t, J = 7.1 Hz, 3H), 1.15 (s, 6H); ^{13}C NMR (101 MHz, $CDCl_3$): δ 168.8, 161.9, 155.0, 153.5, 129.3, 125.6, 61.6, 60.8, 46.3, 44.6, 42.2, 39.3, 29.1 (2C), 14.3, 14.0; HRMS calcd. For $C_{16}H_{23}O_4^+$ $[M + H]^+$ 343.1032, found 343.1031.

Ethyl 5,5-dimethyl-5,6-dihydro-4H-cyclopenta[c]thiophene-1-carboxylate S5

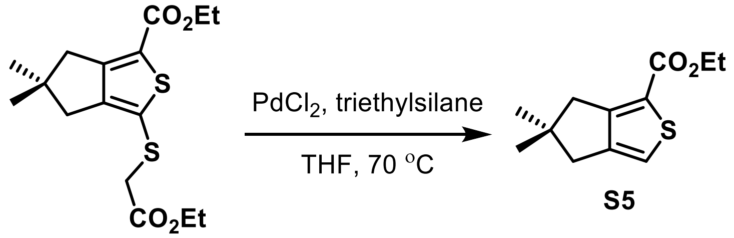

13

According to a modified literature procedure.[7] To a stirred solution of **13** (0.18 g, 0.53 mmol, 1.0 equiv.) in THF (2.0 mL) at room temperature was added $PdCl_2$ (9.5 mg, 54 μmol, 0.1 equiv.) followed by triethylsilane (0.17 mL, 1.06 mmol, 2.0 equiv.) dropwise. The resulting mixture was warmed to 70 °C and stirred for 16 h before it was cooled to room temperature and filtered through Celite®, eluted with CH_2Cl_2 (3 × 3 mL) and concentrated under reduced pressure. Flash column chromatography (silica gel, pentane:Et_2O 12:1→8:1) afforded the title compound **S5** (0.11 g, 0.49 mmol, 93%) as a clear oil. **S5**: R_f = 0.67 (silica gel, pentane: Et_2O 5:1); IR (film) ν_{max} 2921, 1718, 1264, 1125, 1103, 726 cm^{-1}; ^{1}H NMR (400 MHz, $CDCl_3$): δ 6.95 (s, 1H), 4.30 (q, *J* = 7.1 Hz, 2H), 2.75 (s, 2H), 2.49 (s, 2H), 1.35 (t, *J* = 7.1 Hz, 3H), 1.17 (s, 6H); ^{13}C NMR (101 MHz, $CDCl_3$): δ 162.8, 155.5, 148.6, 123.1, 121.3, 60.6, 46.6, 43.9, 42.5, 29.1 (2C), 14.4; HRMS calcd. For $C_{12}H_{17}O_2S^+$ $[M + H]^+$ 225.0944, found 225.0944.

Ethyl 5,5-dimethyl-5,6-dihydro-4H-cyclopenta[c]thiophene-1-carboxylate 2,2-dioxide, 12

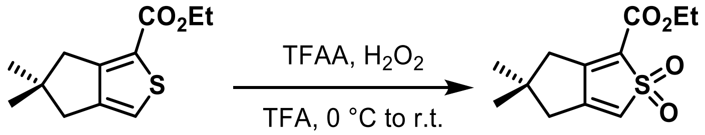

S5 12

According to a modified literature procedure.[2] To a stirred solution of TFAA (0.68 mL, 4.89 mmol, 10 equiv.) and TFA (0.5 mL) at 0 °C was added H_2O_2 (0.19 mL, 1.86 mmol, 3.8 equiv., aq. 30%) dropwise. The resulting mixture was warmed to room temperature and stirred for 15 min. To the resulting mixture at 0 °C was added a solution of **S5** (0.11 g, 0.49 mmol, 1.0 equiv.) in TFA (1.0 mL) dropwise. The resulting mixture was warmed to room temperature and stirred for 14 h before it was concentrated under reduced pressure. Flash column chromatography (silica gel, pentane:EtOAc 6:1) afforded the title compound **12** (0.10 g, 0.39 mmol, 80%) as a white waxy solid. **12**: R_f = 0.43 (silica gel, pentane:EtOAc 4:1); IR (film) ν_{max} 2958, 1711, 1605, 1369, 1288, 1154, 1117, 1096, 1027, 790 cm^{-1}; ^{1}H NMR (400 MHz, $CDCl_3$): δ 6.36 (t, *J* = 2.3 Hz, 1H), 4.32 (q, *J* = 7.1 Hz, 2H), 2.76 (s, 2H), 2.47 (d, *J* = 2.3 Hz, 2H), 1.34 (t, *J* = 7.1 Hz, 3H), 1.11 (s, 6H); ^{13}C NMR (101 MHz, $CDCl_3$): δ 159.5, 158.2, 147.4, 125.3, 123.6, 61.8, 43.6, 41.9, 41.2, 28.0 (2C), 14.0; HRMS calcd. For $C_{12}H_{17}O_4S^+$ $[M + H]^+$ 257.0842, found 257.0842.

Ethyl 2,2,6-trimethyl-5-(2-(methylthio)-2-oxoethyl)-2,3-dihydro-1H-indene-4-carboxylate, 14

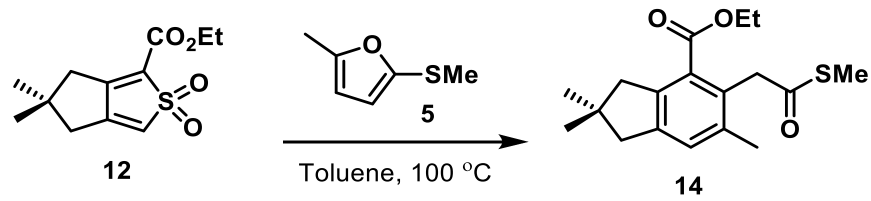

To a stirred solution of **12** (99.0 mg, 0.39 mmol, 1.0 equiv.) in toluene (2.0 mL) at room temperature was added 2-methyl-5-(methylthio)furan **5** (0.19 mL, 1.58 mmol, 4.1 equiv.). The resulting mixture was warmed to 100 °C and stirred for 36 h before it was cooled to room temperature and concentrated under reduced pressure. Flash column chromatography (silica gel, pentane:Et_2O 15:1→9:1) afforded the title compound **14** (79.0 mg, 0.25 mmol, 64%) as a yellow oil. **14**: R_f = 0.68 (silica gel, pentane:EtOAc 10:1); IR (film) ν_{max} 2953, 1714, 1700, 1463, 1300, 1030 cm^{-1}; ^1H NMR (400 MHz, $CDCl_3$): δ 7.11 (s, 1H), 4.35 (q, *J* = 7.1 Hz, 2H), 4.09 (s, 2H), 2.84 (s, 2H), 2.69 (s, 2H), 2.29 (s, 3H), 2.26 (s, 3H), 1.36 (t, *J* = 7.1 Hz, 3H), 1.13 (s, 6H); ^{13}C NMR (101 MHz, $CDCl_3$): δ 197.8, 168.7, 143.8, 141.4, 136.4, 129.9, 129.2, 128.6, 60.9, 48.1, 47.3, 44.7, 39.7, 28.8 (2C), 20.3, 14.2, 11.7; HRMS calcd. For $C_{18}H_{24}O_3SNa^+$ $[M + Na]^+$ 343.1338, found 343.1336.

Alcyopterosin O

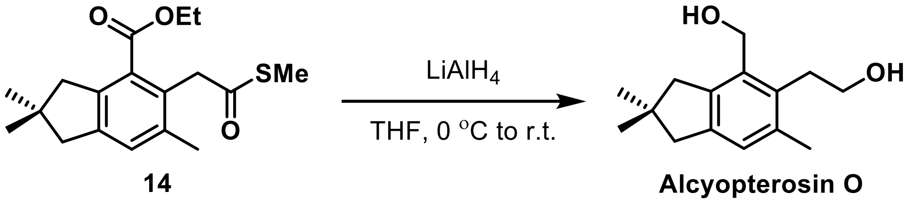

To a stirred solution of **14** (79.0 mg, 0.25 mmol, 1.0 equiv.) in THF (2.0 mL) at 0 °C was added $LiAlH_4$ (37.4 mg, 0.99 mmol, 4.0 equiv.). The resulting mixture was warmed to room temperature and stirred for 2 h before it was cooled to 0 °C and quenched with sodium potassium tartrate (3 mL, sat. aq.), diluted with EtOAc (10 mL) and stirred vigorously for 1 h. The layers were separated and the aqueous layer was extracted with EtOAc (3 × 8 mL), the combined organic layer was washed with water (20 mL), brine (20 mL), dried (Na_2SO_4) and concentrated under reduced pressure. Flash column chromatography (silica gel, pentane:EtOAc 2:1→1:5) afforded **alcyopterosin O** (53.0 mg, 0.23 mmol, 92%) as a white semi-solid. **Alcyopterosin O**: R_f = 0.32 (silica gel, pentane:EtOAc 1:3); IR (film) ν_{max} 3293, 2952, 2924, 1463, 1434, 1381, 1364, 1028 cm^{-1}; ^1H NMR (400 MHz, $CDCl_3$): δ 6.98 (s, 1H), 4.56 (s, 2H), 4.03 (s, 2H), 3.76 (t, *J* = 5.8 Hz, 2H), 2.95 (t, *J* = 5.8 Hz, 2H), 2.80 (s, 2H), 2.69 (s, 2H), 2.29 (s, 3H), 1.16 (s, 6H); ^{13}C NMR (101 MHz, $CDCl_3$): δ 141.7, 141.1, 135.4, 134.8, 133.8, 126.9, 61.1, 59.4, 47.8, 46.4, 39.4, 31.5, 29.1 (2C), 20.2; HRMS calcd. For $C_{15}H_{22}O_2Na^+$ $[M + Na]^+$ 257.1512, found 257.1511.

^{1}H and ^{13}C NMR ($CDCl_3$, ppm) comparison for alcyopterosin O

Dudley[10] (^{1}H, 400 MHz, ppm)	This work (^{1}H, 400 MHz, ppm)	Dudley[10] (^{13}C, 100 MHz, ppm)	This work (^{13}C, 101 MHz, ppm)
6.98 (s, 1H)	6.98 (s, 1H)	141.9	141.7
4.57 (s, 2H)	4.56 (s, 2H)	141.2	141.1
3.80 (t, *J* = 5.8 Hz, 2H)	4.03 (s, 2H)	135.6	135.4
2.97 (t, *J* = 5.8 Hz, 2H)	3.76 (t, *J* = 5.8 Hz, 2H)	134.8	134.8
2.79 (s, 2H)	2.95 (t, *J* = 5.8 Hz, 2H)	133.7	133.8
2.69 (s, 2H)	2.80 (s, 2H)	126.9	126.9
2.28 (s, 3H)	2.69 (s, 2H)	61.3	61.1
1.14 (s, 6H)	2.29 (s, 3H)	59.6	59.4
	1.16 (s, 6H)	47.8	47.8
		46.4	46.4
		39.5	39.4
		31.5	31.5
		29.1	29.1 (2C)
		20.2	20.2

2-(2,2,4,6-tetramethyl-2,3-dihydro-1H-inden-5-yl)ethan-1-ol, 15

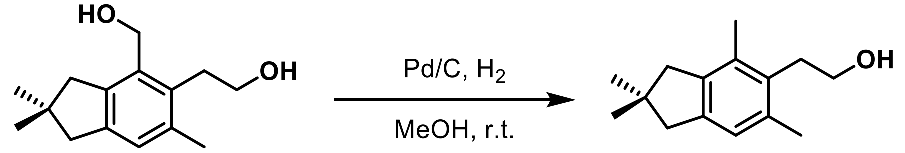

Alcyopterosin O **15**

To a stirred solution of **alcyopterosin O** (39.0 mg, 0.17 mmol, 1.0 equiv.) in MeOH (1.0 mL) at room temperature was added Pd/C (10% wt/wt, 17.7 mg, 17 μmol, 0.1 equiv.). The resulting mixture was evacuated and filled with hydrogen (3 ×) and stirred under an atmosphere of H_2 (balloon) for 3 h. The resulting mixture was filtered through Celite®, eluted with CH_2Cl_2 (3 × 3 mL) and concentrated under reduced pressure. Flash column chromatography (silica gel, pentane:EtOAc 3:1→1:3) afforded the title compound **15** (35.0 mg, 0.16 mmol, 96%) as a white amorphous solid. **15**: R_f = 0.56 (silica gel, pentane:EtOAc 1:3); IR (film) ν_{max} 3334, 2952, 2921, 1464, 1364, 1041, 861 cm^{-1}; ^{1}H NMR (400 MHz, $CDCl_3$): δ 6.85 (s, 1H), 3.75 (t, *J* = 7.5 Hz, 2H), 2.95 (t, *J* = 7.5 Hz, 2H), 2.69 (s, 2H), 2.65 (s, 2H), 2.32 (s, 3H), 2.22 (s, 3H), 1.47 (br s, 1H), 1.15 (s, 6H); ^{13}C NMR (101 MHz, $CDCl_3$): δ 141.2, 140.7, 134.8, 132.9, 131.8, 124.2, 62.0, 47.9, 47.2, 39.2, 32.8, 29.3 (2C), 20.4, 16.1; HRMS calcd. For $C_{15}H_{22}ONa^{+}$ $[M + Na]^{+}$ 241.1563, found 241.1564.

Alcyopterosin A

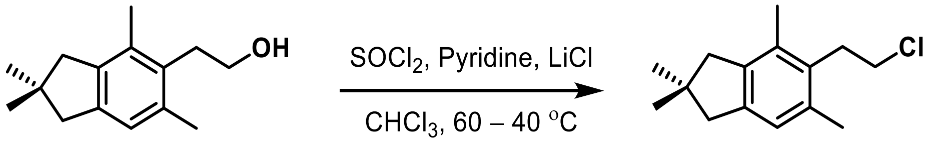

15 → Alcyopterosin A

According to a modified literature procedure.[8] To a stirred solution of **15** (14.6 mg, 67 μmol, 1.0 equiv.) in pyridine (0.5 M in $CHCl_3$, 0.29 mL, 0.15 mmol, 2.2 equiv.) at 60 °C was added $SOCl_2$ (0.5 M in $CHCl_3$, 0.19 mL, 95 μmol, 1.4 equiv.) dropwise. The resulting mixture was cooled to 40 °C and stirred for 15 min. To the mixture at 40 °C was added LiCl (6.1 mg, 0.14 mmol, 2.1 equiv.) and stirred for 14 h. The resulting mixture was cooled to room temperature, diluted with brine (2 mL) and stirred vigorously for 0.5 h. The layers were separated and the aqueous layer was extracted with pentane (5 × 3 mL), the combined organic layer was washed with $NaHCO_3$ (10 mL, sat. aq.), dried (Na_2SO_4) and concentrated. Flash chromatography (silica gel, pentane:Et_2O 1:0→20:1) afforded **alcyopterosin A** (10.2 mg, 43 μmol, 64%) as a clear semi-solid. **Alcyopterosin A**: R_f = 0.43 (silica gel, pentane:EtOAc 1:0); IR (film) ν_{max} 2953, 2924, 2866, 2836, 1464, 1381, 1364, 1320, 1246 cm^{-1}; ^1H NMR (400 MHz, $CDCl_3$): δ 6.86 (s, 1H), 3.58-3.49 (m, 2H), 3.17-3.07 (m, 2H), 2.69 (s, 2H), 2.65 (s, 2H), 2.32 (s, 3H), 2.21 (s, 3H), 1.15 (s, 6H); ^{13}C NMR (101 MHz, $CDCl_3$): δ 141.8, 140.8, 134.6, 132.6, 131.9, 124.3, 47.9, 47.1, 42.5, 39.3, 33.5, 29.3, 20.1, 15.9; HRMS calcd. For $C_{15}H_{21}Cl^+$ $[M]^+$ 236.1326, found 236.1332.

^1H and ^{13}C NMR ($CDCl_3$, ppm) comparison for alcyopterosin A

Dudley[11] (^1H, 400 MHz, ppm)	This work (^1H, 400 MHz, ppm)	Dudley[11] (^{13}C, 100 MHz, ppm)	This work (^{13}C, 101 MHz, ppm)
6.86 (s, 1H)	6.86 (s, 1H)	141.9	141.8
3.53 (t, *J* = 8.6 Hz, 2H)	3.58-3.49 (m, 2H)	140.9	140.8
3.11 (t, *J* = 8.6Hz, 2H)	3.17-3.07 (m, 2H)	134.6	134.6
2.69 (s, 2H)	2.69 (s, 2H)	132.6	132.6
2.65 (s, 2H)	2.65 (s, 2H)	132.0	131.9
2.31 (s, 3H)	2.32 (s, 3H)	124.3	124.3
2.21 (s, 3H)	2.21 (s, 3H)	47.9	47.9
1.14 (s, 6H)	1.15 (s, 6H)	47.1	47.1
		42.5	42.5
		39.3	39.3
		33.4	33.5
		29.3	29.3
		20.1	20.1
		16.2	15.9

2-(2,2,4,6-Tetramethyl-2,3-dihydro-1H-inden-5-yl)ethyl 4-methylbenzenesulfonate, S6

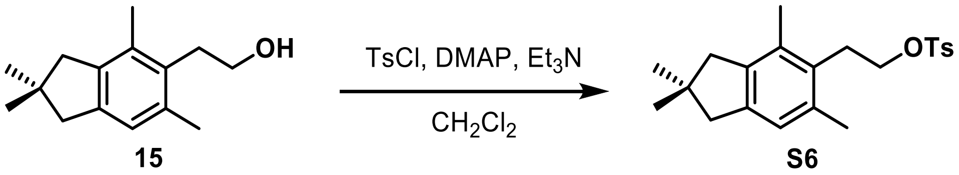

To a stirred solution of **15** (38.0 mg, 0.17 mmol, 1.0 equiv.) in CH_2Cl_2 (2.0 mL) at room temperature was added Et_3N (73 µL, 0.52 mmol, 3.1 equiv.), DMAP (2.1 mg, 17.2 µmol, 0.1 equiv.) and TsCl (49.8 mg, 0.26 mmol, 1.5 equiv.). The resulting mixture was stirred for 14 h before it was diluted with water (3 mL). The layers were separated and the aqueous layer was extracted with CH_2Cl_2 (3 × 3 mL). The combined organic layers were washed with $NaHCO_3$ (10 mL, sat. aq.), dried (Na_2SO_4) and concentrated. Flash chromatography (silica gel, pentane:Et_2O 1:0→20:1) afforded the title compound **S6** (61.2 mg, 0.16 mmol, 94%) as a tan sticky solid. **S6**: R_f = 0.48 (silica gel, pentane:Et_2O 5:1); IR (film) ν_{max} 2955, 2931, 2866, 2836, 1465, 1362, 1188, 1176, 1097, 958, 813 cm^{-1}; ^1H NMR (400 MHz, $CDCl_3$): δ 7.76 (d, J = 8.4 Hz, 2H), 7.32 (d, J = 7.9 Hz, 2H), 6.82 (s, 1H), 4.12-3.97 (m, 2H), 3.08-2.99 (m, 2H), 2.69 (s, 2H), 2.62 (s, 2H), 2.45 (s, 3H), 2.21 (s, 3H), 2.11 (s, 3H), 1.15 (s, 6H); ^{13}C NMR (101 MHz, $CDCl_3$): δ 144.6, 141.8, 140.7, 134.7, 133.2, 132.8, 129.7 (2C), 129.4, 127.8 (2C), 124.3, 68.6, 47.9, 47.0, 39.2, 29.2, 29.2 (2C), 21.6, 20.0, 15.8; HRMS calcd. For $C_{22}H_{28}O_3SNa^+$ $[M + Na]^+$ 395.1651, found 395.1650.

Alcyopterosin B

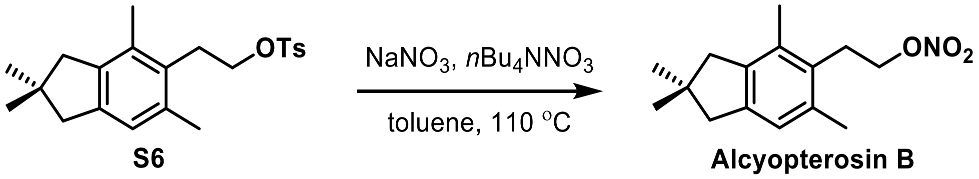

According to a modified literature procedure.[9] To a stirred solution of **S6** (17.5 mg, 47 µmol, 1.0 equiv.) in toluene (1.5 mL) at room temperature was added $NaNO_3$ (35.9 mg, 0.42 mmol, 8.9 equiv.) and $n$$Bu_4NNO_3$ (64.4 mg, 0.21 mmol, 4.5 equiv.). The resulting mixture was warmed to 110 °C and stirred for 5 h before it was cooled to room temperature, filtered through Celite®, eluted with CH_2Cl_2 (3 × 3 mL) and concentrated under reduced pressure. Flash column chromatography (silica gel, pentane:Et_2O 1:0→20:1) afforded **alcyopterosin B** (10.5 mg, 40 µmol, 85%) as a clear oil. **Alcyopterosin B**: R_f = 0.78 (silica gel, pentane:Et_2O 18:1); IR (film) ν_{max} 2954, 2923, 2866, 2836, 1634, 1465, 1277, 858 cm^{-1}; ^1H NMR (400 MHz, $CDCl_3$): δ 6.91 (s, 1H), 4.58 – 4.45 (m, 2H), 3.16 – 3.04 (m, 2H), 2.73 (s, 2H), 2.69 (s, 2H), 2.35 (s, 3H), 2.25 (s, 3H), 1.18 (s, 6H); ^{13}C NMR (101 MHz, $CDCl_3$): δ 142.1, 140.9, 134.8, 132.9, 129.3, 124.5, 71.5, 47.9, 47.1, 39.3, 29.2 (2C), 27.1, 20.1, 15.9.

^{1}H and ^{13}C NMR ($CDCl_3$, ppm) comparison for alcyopterosin B

Isolation[12] (^{1}H, 200 MHz, ppm)	This work (^{1}H, 400 MHz, ppm)	Isolation[12] (^{13}C, 50 MHz, ppm)	This work (^{13}C, 101 MHz, ppm)
6.87 (s, 1H)	6.91 (s, 1H)	142.1	142.1
4.48 (t, J = 8 Hz, 2H)	4.58 – 4.45 (m, 2H)	140.9	140.9
3.06 (t, J = 8 Hz, 2H)	3.16 – 3.04 (m, 2H)	134.8	134.8
2.69 (s, 2H)	2.73 (s, 2H)	132.8	132.9
2.65 (s, 2H)	2.69 (s, 2H)	129.3	129.3
2.32 (s, 3H)	2.35 (s, 3H)	124.5	124.5
2.21 (s, 3H)	2.25 (s, 3H)	71.5	71.5
1.15 (s, 3H)	1.18 (s, 6H)	47.4	47.9
1.15 (s, 3H)		47.1	47.1
		39.3	39.3
		29.2	29.2
		29.2	29.2
		27.1	27.1
		20.1	20.1
		15.9	15.9

Alcyopterosin C + S7

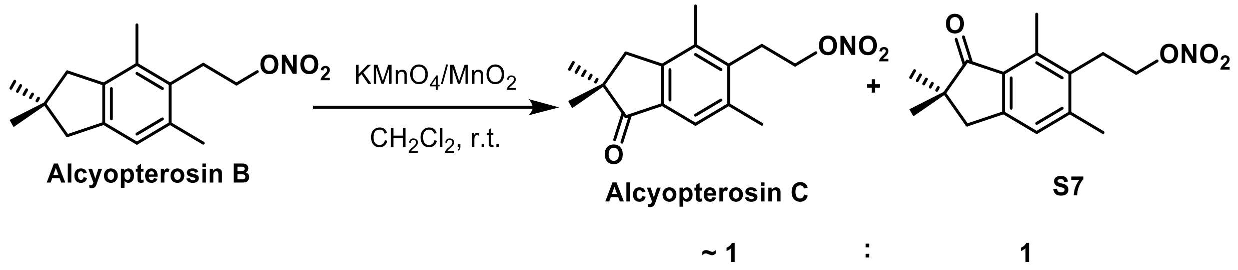

According to a modified literature procedure.[4] To a stirred solution of **7** (25.0 mg, 95 μmol) in CH_2Cl_2 (1.5 mL) at room temperature was added $KMnO_4/MnO_2$ (0.10 g; the oxidant was prepared by grinding potassium permanganate and active manganese dioxide [1:3 ratio] in a mortar until a homogeneous powder was obtained).[4] The resulting mixture was stirred for 48 h before additional oxidant (0.10 g) was added. The resulting mixture was furthered stirred for 48 h before it was filtered through Celite,® eluted with CH_2Cl_2 (3 × 3 mL) and concentrated under reduced pressure. Flash column chromatography (silica gel, pentane:Et_2O 12:1→3:1) afforded **alcyopterosin C** (10.5 mg, 23 μmol, 46% brsm, 40%) s a white sticky solid, **S7** (9.8 mg, 22 μmol, 43% brsm, 37%) as a white sticky solid and recovered alcyopterosin B **7** (3.5 mg, 11 μmol).

Alcyopterosin C: R_f = 0.49 (silica gel, pentane:Et_2O 5:1); IR (film) ν_{max} 2964, 2926, 2868, 1710, 1632, 1464, 1279, 858 cm^{-1}; ^1H NMR (500 MHz, $CDCl_3$): δ 7.47 (s, 1H), 4.59 – 4.49 (m, 2H), 3.24 – 3.15 (m, 2H), 2.87 (s, 2H), 2.42 (s, 3H), 2.33 (s, 3H), 1.23 (s, 6H); ^{13}C NMR (126 MHz, $CDCl_3$): δ 211.5, 149.7, 139.4, 136.8, 134.6, 134.0, 123.5, 70.6, 45.5, 42.3, 27.8, 25.4 (2C), 20.3, 14.8; HRMS calcd. For $C_{15}H_{20}O_4N^+$ $[M + H]^+$ 278.1387, found 278.1389.

S7: R_f = 0.68 (silica gel, pentane:Et_2O 5:1); IR (film) ν_{max} 2962, 2926, 2863, 1702, 1634, 1465, 1278, 858 cm^{-1}; ^1H NMR (500 MHz, $CDCl_3$): δ 7.11 (s, 1H), 4.57 – 4.44 (m, 2H), 3.21 – 3.10 (m, 2H), 2.87 (s, 2H), 2.69 (s, 3H), 2.44 (s, 3H), 1.20 (s, 6H); ^{13}C NMR (126 MHz, $CDCl_3$): δ 211.9, 152.0, 144.1, 138.5, 132.2, 131.3, 126.2, 70.9, 45.6, 41.7, 26.3, 25.5 (2C), 21.1, 13.6; HRMS calcd. For $C_{15}H_{20}O_4N^+$ $[M + H]^+$ 278.1387, found 278.1388.

^1H and ^{13}C NMR ($CDCl_3$, ppm) comparison for alcyopterosin C

Witulski[9] (^1H, 400 MHz, ppm)	This work (^1H, 500 MHz, ppm)	Witulski[12] (^{13}C, 100 MHz, ppm)	This work (^{13}C, 126 MHz, ppm)
7.47 (s, 1H)	7.47 (s, 1H)	211.6	211.5
4.54 (t, J = 7.6 Hz, 2H)	4.59 – 4.49 (m, 2H)	149.9	149.7
3.19 (t, J = 7.6 Hz, 2H)	3.24 – 3.15 (m, 2H)	139.6	139.4
2.88 (s, 2H)	2.87 (s, 2H)	137.0	136.8
2.42 (s, 3H)	2.42 (s, 3H)	134.8	134.6
2.33 (s, 3H)	2.33 (s, 3H)	134.2	134.0
1.23 (s, 6H)	1.23 (s, 6H)	123.7	123.5
		70.8	70.6
		45.7	45.5
		42.5	42.3
		27.9	27.8
		25.6	25.4 (2C)
		20.4	20.3
		15.0	14.8

Alcyopterosin H

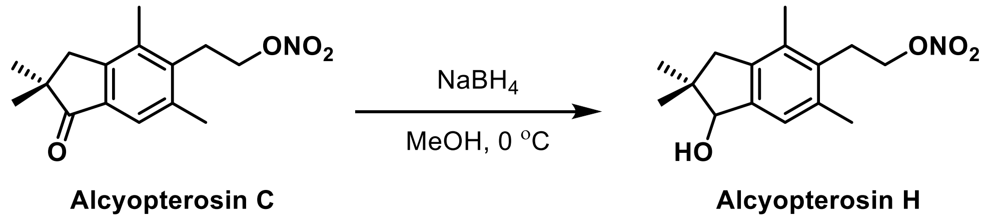

To a stirred solution of **alcyopterosin C** (15.0 mg, 54 μmol, 1.0 equiv.) in MeOH (1.0 mL) at 0 °C was added $NaBH_4$ (10.0 mg, 0.26 mmol, 4.8 equiv.). The resulting mixture was stirred for 1 h before it was diluted with brine (2 mL) and CH_2Cl_2 (2 mL). The layers were separated and the aqueous layer was extracted with CH_2Cl_2 (3 × 3 mL), the combined organic layer was washed with water (10 mL), brine (10 mL), dried (Na_2SO_4) and concentrated under reduced pressure. Flash column chromatography (silica gel, pentane:EtOAc 4:1) afforded **alcyopterosin H** (14.1 mg, 50 μmol, 93%) as a clear sticky solid. **Alcyopterosin H**: R_f = 0.18 (silica gel, pentane:Et_2O 4:1); IR (film) ν_{max} 3339, 2958, 2926, 1631, 1466, 1277, 859 cm^{-1}; ^1H NMR (500 MHz, $CDCl_3$): δ 7.08 (s, 1H), 4.63 (d, *J* = 7.6 Hz, 1H), 4.53 – 4.43 (m, 2H), 3.14 – 3.05 (m, 2H), 2.72 (d, *J* = 15.6 Hz, 1H), 2.57 (d, *J* = 15.6 Hz, 1H), 2.36 (s, 3H), 2.23 (s, 3H), 1.56 (d, *J* = 7.7 Hz, 1H), 1.17 (s, 3H), 1.05 (s, 3H); ^{13}C NMR (126 MHz, $CDCl_3$): δ 143.1, 139.3, 135.6, 133.2, 131.7, 124.2, 83.8, 71.3, 44.2, 43.9, 27.2, 27.1, 21.7, 20.2, 15.6; HRMS calcd. For $C_{15}H_{21}NO_4Na^+$ $[M + Na]^+$ 302.1363, found 302.1363.

^1H and ^{13}C NMR ($CDCl_3$, ppm) comparison for alcyopterosin H

Isolation[12] (^1H, 200 MHz, ppm)	This work (^1H, 500 MHz, ppm)	Isolation[12] (^{13}C, 50 MHz, ppm)	This work (^{13}C, 126 MHz, ppm)
7.07 (s, 1H)	7.08 (s, 1H)	143.1	143.1
4.63 (s, 1H)	4.63 (d, *J* = 7.6 Hz, 1H)	139.2	139.3
4.49 (t, *J* = 8, 2H)	4.53 – 4.43 (m, 2H)	135.5	135.6
3.09 (t, *J* = 8, 2H)	3.14 – 3.05 (m, 2H)	133.2	133.2
2.72 (d, *J* = 15.0)	2.72 (d, *J* = 15.6 Hz, 1H)	131.7	131.7
2.56 (d, *J* = 15.0)	2.57 (d, *J* = 15.6 Hz, 1H)	124.2	124.2
2.36 (s, 3H)	2.36 (s, 3H)	83.7	83.8
2.23 (s, 3H)	2.23 (s, 3H)	71.3	71.3
1.17 (s, 3H)	1.56 (d, *J* = 7.7 Hz, 1H)	44.2	44.2
1.05 (s, 3H)	1.17 (s, 3H)	43.8	43.9
	1.05 (s, 3H)	27.2	27.2
		27.1	27.1
		21.8	21.7
		20.2	20.2
		15.6	15.6

Methyl 5,6-dihydro-4H-cyclopenta[b]thiophene-2-carboxylate, S9

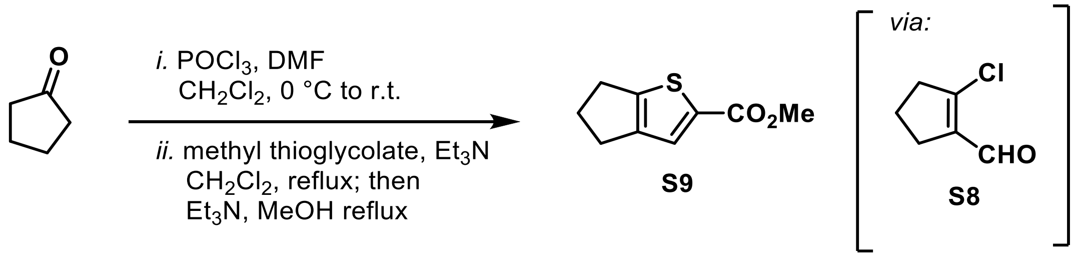

According to a modified literature procedure.[1] (i) To a stirred solution of anhydrous DMF (0.29 mL, 3.75 mmol, 1.6 equiv.) at 0 °C was added phosphorus oxychloride (0.26 mL, 2.78 mmol, 1.2 equiv.) dropwise. The resulting mixture was warmed to room temperature and stirred for 1 h before a solution cyclopentanone (0.20 g, 2.38 mmol, 1.0 equiv.) in CH_2Cl_2 (1.0 mL) was added. The resulting mixture was warmed to reflux and stirred for 4 h before it was cooled to room temperature and poured into mixture of ice and NaOAc (0.60 g, 7.31 mmol, 3.1 equiv.). The layers were separated and the aqueous layer was extracted with CH_2Cl_2 (3 × 5 mL), the combined organic layer was washed with water (10 mL), brine (10 mL), dried (Na_2SO_4) and concentrated under reduced pressure to afford aldehyde **S8** as a dark orange oil, which was used directly in the subsequent step without further purification. **S8**: R_f = 0.62 (silica gel, pentane:Et_2O 5:1).

(ii) To a stirred solution of crude aldehyde **S8** (0.20 g, 1.53 mmol, 1.0 equiv.) in CH_2Cl_2 (3.0 mL) at room temperature was added methyl thioglycolate (0.14 mL, 1.57 mmol, 1.0 equiv.) and Et_3N (0.85 mL, 6.10 mmol, 4.0 equiv.). The resulting mixture was warmed to reflux and stirred for 16 h before it was cooled to room temperature and concentrated under reduced pressure. To the resulting residue in MeOH (3.0 mL) at room temperature was added Et_3N (0.85 mL, 6.10 mmol, 4.0 equiv.). The resulting mixture was warmed to reflux and stirred for 16 h before it was cooled to room temperature and concentrated under reduced pressure. Flash column chromatography (silica gel, pentane:Et_2O 20:1→6:1) afforded the title compound **S9** (0.21 g, 1.15 mmol, 48% over two steps) as a white sticky solid. **S8**: R_f = 0.59 (silica gel, pentane:Et_2O 5:1); IR (film) ν_{max} 2950, 1707, 1447, 1404, 1274, 752 cm^{-1}; ^1H NMR (500 MHz, $CDCl_3$): δ 7.50 (s, 1H), 3.84 (s, 3H), 2.90 (t, J = 7.3 Hz, 2H), 2.73 (t, J = 7.3 Hz, 2H), 2.43 (p, J = 7.3 Hz, 2H); ^{13}C NMR (126 MHz, $CDCl_3$): δ 162.9, 151.0, 147.0, 135.3, 128.7, 51.8, 29.3, 29.1, 27.9; HRMS calcd. For $C_9H_{11}O_2S^+$ $[M + H]^+$ 183.0474, found 183.0477.

Methyl 5,6-dihydro-4H-cyclopenta[b]thiophene-2-carboxylate 1,1-dioxide, 16

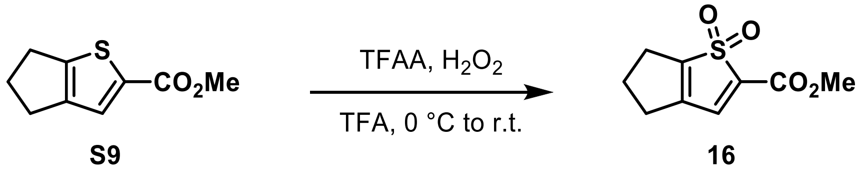

According to a modified literature procedure.[2] To a stirred solution of trifluoroacetic anhydride (0.46 mL, 3.31 mmol, 10 equiv.) and trifluoroacetic acid (0.5 mL) at 0 °C was added H_2O_2 (0.13 mL, 1.27 mmol, 3.8 equiv., aq. 30%) dropwise. The resulting mixture was warmed to room temperature and stirred for 15 min. To the resulting mixture at 0 °C was added a solution of **S9** (60.0 mg, 0.33 mmol, 1.0 equiv.) in TFA (1.0 mL) dropwise. The resulting mixture was warmed to room temperature and stirred for 14 h before it was concentrated under reduced pressure. Flash column chromatography (silica gel, pentane:EtOAc 10:1→1:1) afforded the title compound **16** (52.0 mg, 0.24 mmol, 74%) as a white waxy solid. **16**: R_f = 0.37 (silica gel, pentane:EtOAc 1:1); IR (film) ν_{max} 2958, 1721, 1566, 1437, 1310, 752 cm^{-1}; ^{1}H NMR (400 MHz, $CDCl_3$): δ 7.46 (s, 1H), 3.90 (s, 3H), 2.82-2.71 (m, 2H), 2.69-2.60 (m, 2H), 2.49-2.39 (m, 2H); ^{13}C NMR (101 MHz, $CDCl_3$): δ 158.3, 146.6, 146.0, 139.0, 135.1, 52.9, 28.9, 26.5 (2C); HRMS calcd. For $C_9H_{11}O_4S^+$ $[M + H]^+$ 215.0373, found 215.0373.

Methyl 1-oxo-1,2,3,6,7,8-hexahydro-as-indacene-4-carboxylate 17

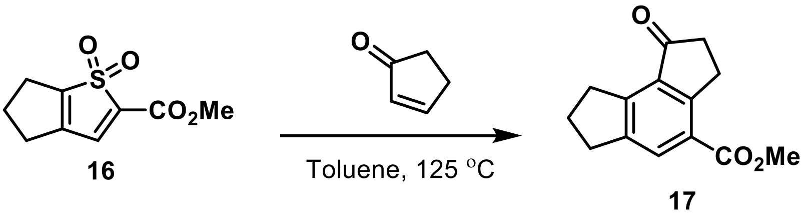

To a stirred solution of **16** (18.0 mg, 84 μmol, 1.0 equiv.) in toluene (0.5 mL) at room temperature was added 2-cyclopenten-1-one (25.0 mg, 0.30 mmol, 3.6 equiv.). The resulting mixture was warmed to 125 °C and stirred for 20 h before it was cooled to room temperature and concentrated under reduced pressure. Flash column chromatography (silica gel, pentane:Et_2O 12:1→6:1) afforded the title compound **17** (8.0 mg, 35 μmol, 42%) as a yellow semi-solid. **17**: R_f = 0.51 (silica gel, pentane:EtOAc 3:1); IR (film) ν_{max} 2956, 2918, 2849, 1710, 1706, 1580, 1436, 1292, 1274, 1193, 1041 cm^{-1}; ^{1}H NMR (500 MHz, $CDCl_3$): δ 8.10 (s, 1H), 3.93 (s, 3H), 3.52 – 3.39 (m, 2H), 3.28 (t, J = 7.5 Hz, 2H), 2.94 (t, J = 7.5 Hz, 2H), 2.73 – 2.64 (m, 2H), 2.17 (p, J = 7.6 Hz, 2H); ^{13}C NMR (126 MHz, $CDCl_3$): δ 207.5, 166.7, 155.7, 148.7, 144.9, 133.9, 131.6, 125.9, 51.9, 36.5, 31.5, 31.4, 27.2, 25.4; HRMS calcd. For $C_{14}H_{15}O_3^+$ $[M + H]^+$ 231.1016, found 231.1017.

Trimethyl 2,3-dihydro-1H-indene-4,5,6-tricarboxylate 18

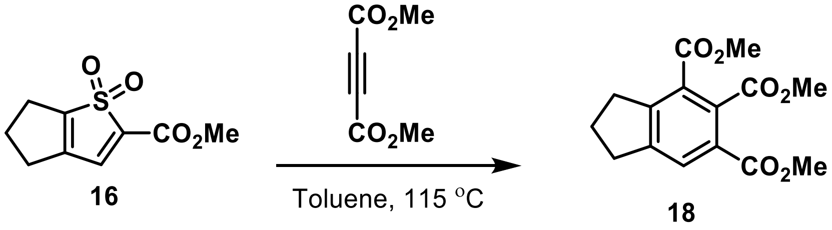

To a stirred solution of **16** (12.4 mg, 58 μmol, 1.0 equiv.) in toluene (0.5 mL) at room temperature was added dimethyl acetylenedicarboxylate (24.6 mg, 0.17 mmol, 2.9 equiv.). The resulting mixture was warmed to 115 °C and stirred for 40 h before it was cooled to room temperature and concentrated under reduced pressure. Flash column chromatography (silica gel, pentane:Et_2O 9:1→6:1) afforded the title compound **18** (11.5 mg, 39 μmol, 68%) as a yellow oil. **18**: R_f = 0.38 (silica gel, pentane:EtOAc 3:1); IR (film) ν_{max} 2953, 1729, 1438, 1344, 1274, 1237, 1147, 806 cm^{-1}; ^1H NMR (400 MHz, $CDCl_3$): δ 7.92 (s, 1H), 3.92 (s, 3H), 3.87 (s, 6H), 3.17 (t, J = 7.6 Hz, 2H), 2.97 (t, J = 7.6 Hz, 2H), 2.11 (p, J = 7.6 Hz, 2H); ^{13}C NMR (101 MHz, $CDCl_3$): δ 169.0, 166.5, 165.9, 150.4, 147.2, 134.4, 128.6, 127.5, 126.3, 52.7, 52.5, 52.3, 33.9, 32.5, 24.8; HRMS calcd. For $C_{15}H_{16}O_6Na^+$ $[M + Na]^+$ 315.0839, found 315.0840.

Methyl 7-methyl-6-(2-(methylthio)-2-oxoethyl)-2,3-dihydro-1H-indene-5-carboxylate, S10

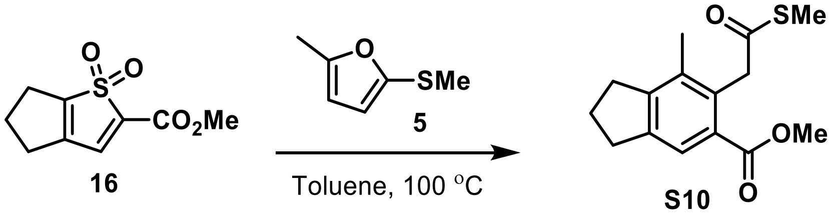

To a stirred solution of **16** (16.5 mg, 77 μmol, 1.0 equiv.) in toluene (0.4 mL) at room temperature was added 2-methyl-5-(methylthio)furan **13** (39.5 mg, 0.31 mmol, 4.0 equiv.). The resulting mixture was warmed to 100 °C and stirred for 20 h before it was cooled to room temperature and concentrated under reduced pressure. Flash column chromatography (silica gel, pentane:Et_2O 12:1→9:1) afforded the title compound **S10** (14.4 mg, 52 μmol, 67%) as a yellow oil. **S10**: R_f = 0.85 (silica gel, pentane:EtOAc 3:1); IR (film) ν_{max} 2949, 1715, 1694, 1435, 1313, 1007 cm^{-1}; ^1H NMR (400 MHz, $CDCl_3$): δ 7.69 (s, 1H), 4.34 (s, 2H), 3.86 (s, 3H), 2.94 (dt, J = 18.1, 7.3 Hz, 4H), 2.27 (s, 3H), 2.24 (s, 3H), 2.10 (p, J = 7.6 Hz, 2H); ^{13}C NMR (101 MHz, $CDCl_3$): δ 198.1, 168.5, 148.5, 143.1, 134.7, 131.3, 129.0, 124.2, 52.0, 44.6, 33.0, 32.7, 24.6, 16.5, 11.7; HRMS calcd. For $C_{15}H_{18}O_3SNa^+$ $[M + Na]^+$ 301.0869, found 301.0869.

3. Copies of ^1H and ^{13}C NMR Spectra

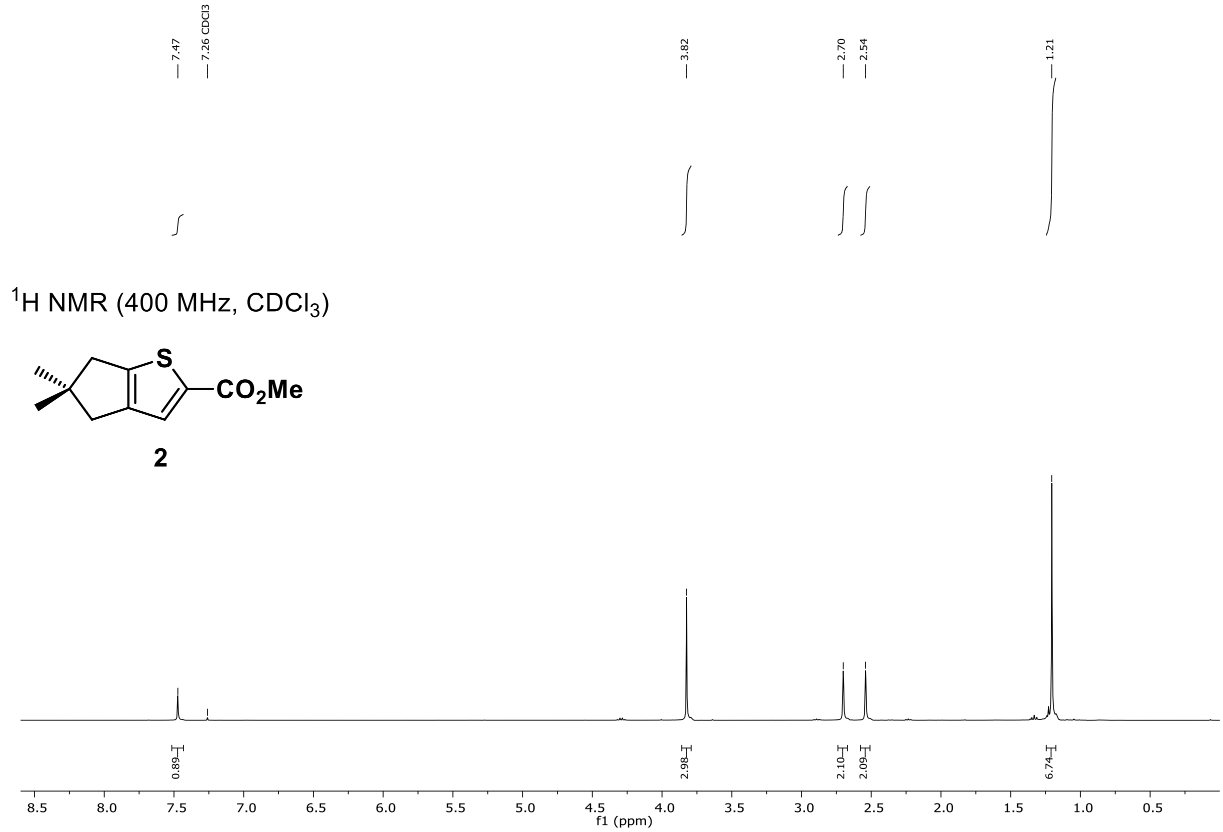

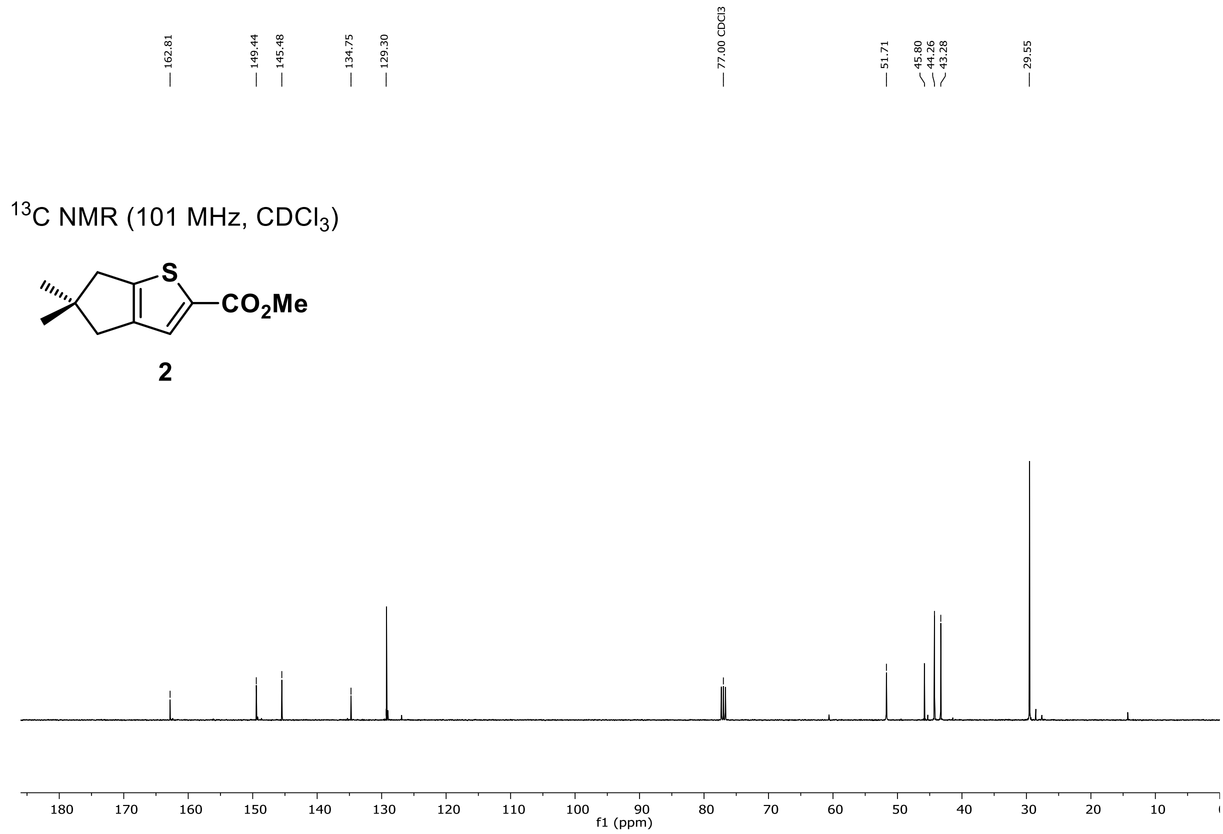

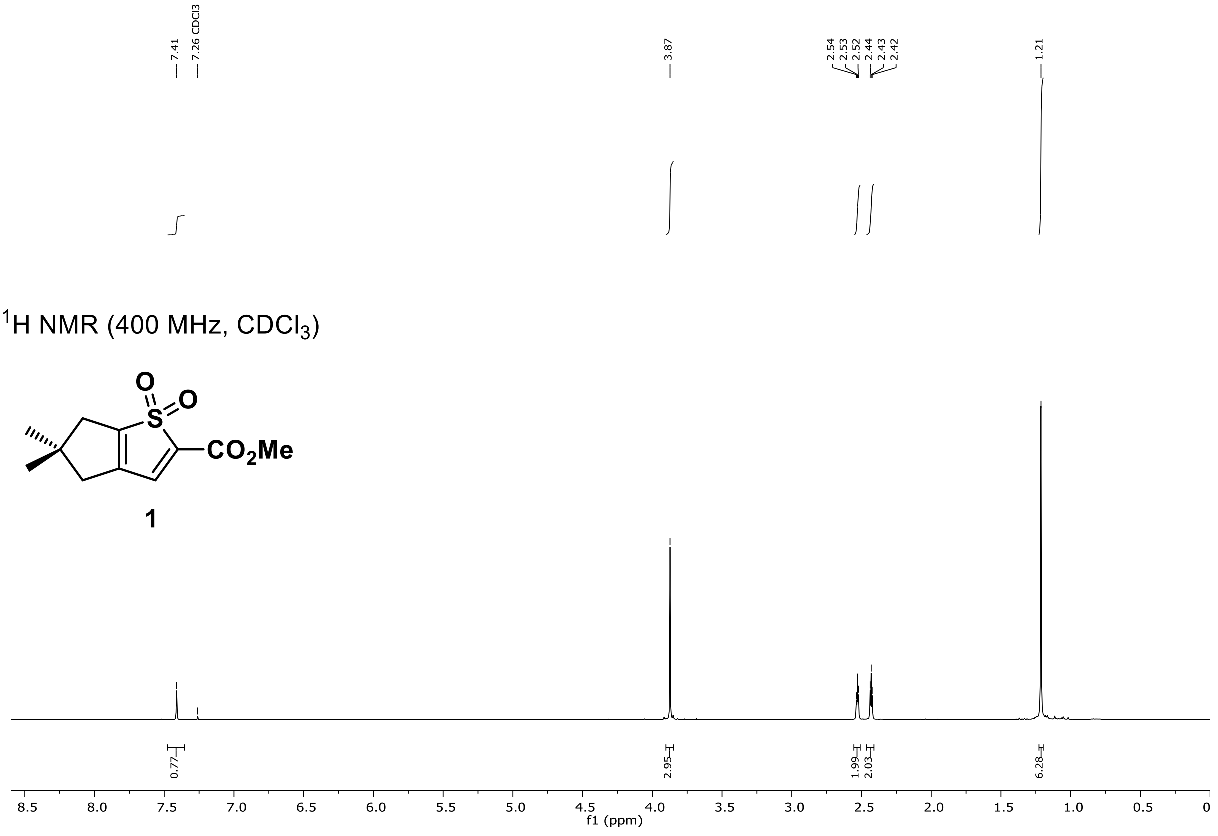
7.41
7.26 CDCl3
3.87
2.54
2.53
2.52
2.44
2.43
2.42
1.21
1H NMR (400 MHz, CDCl3)
O
S
O
CO2Me
1
0.77
2.95
1.99
2.03
6.28
8.5 8.0 7.5 7.0 6.5 6.0 5.5 5.0 4.5 4.0 3.5 3.0 2.5 2.0 1.5 1.0 0.5 0
f1 (ppm)

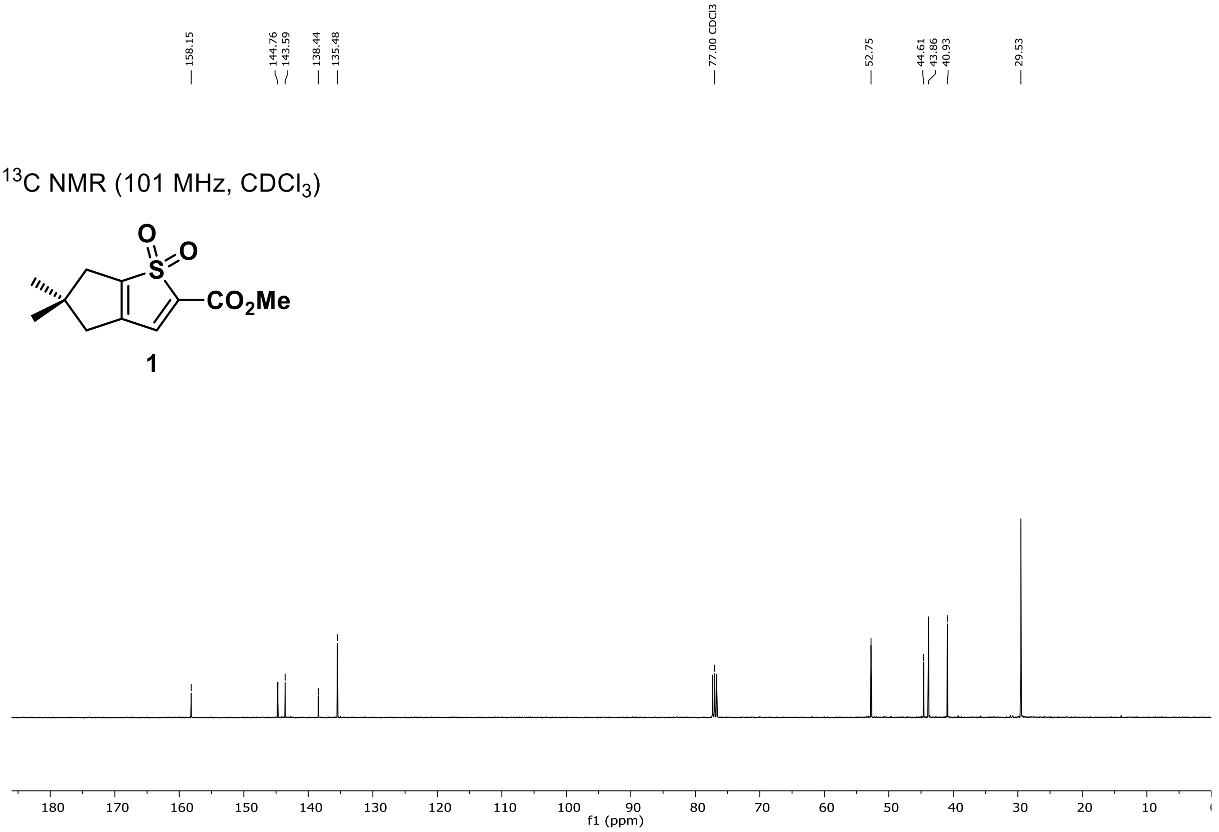
158.15
144.76
143.59
138.44
135.48
77.00 CDCl3
52.75
44.61
43.86
40.93
29.53
13C NMR (101 MHz, CDCl3)
O
S
O
CO2Me
1
180 170 160 150 140 130 120 110 100 90 80 70 60 50 40 30 20 10
f1 (ppm)

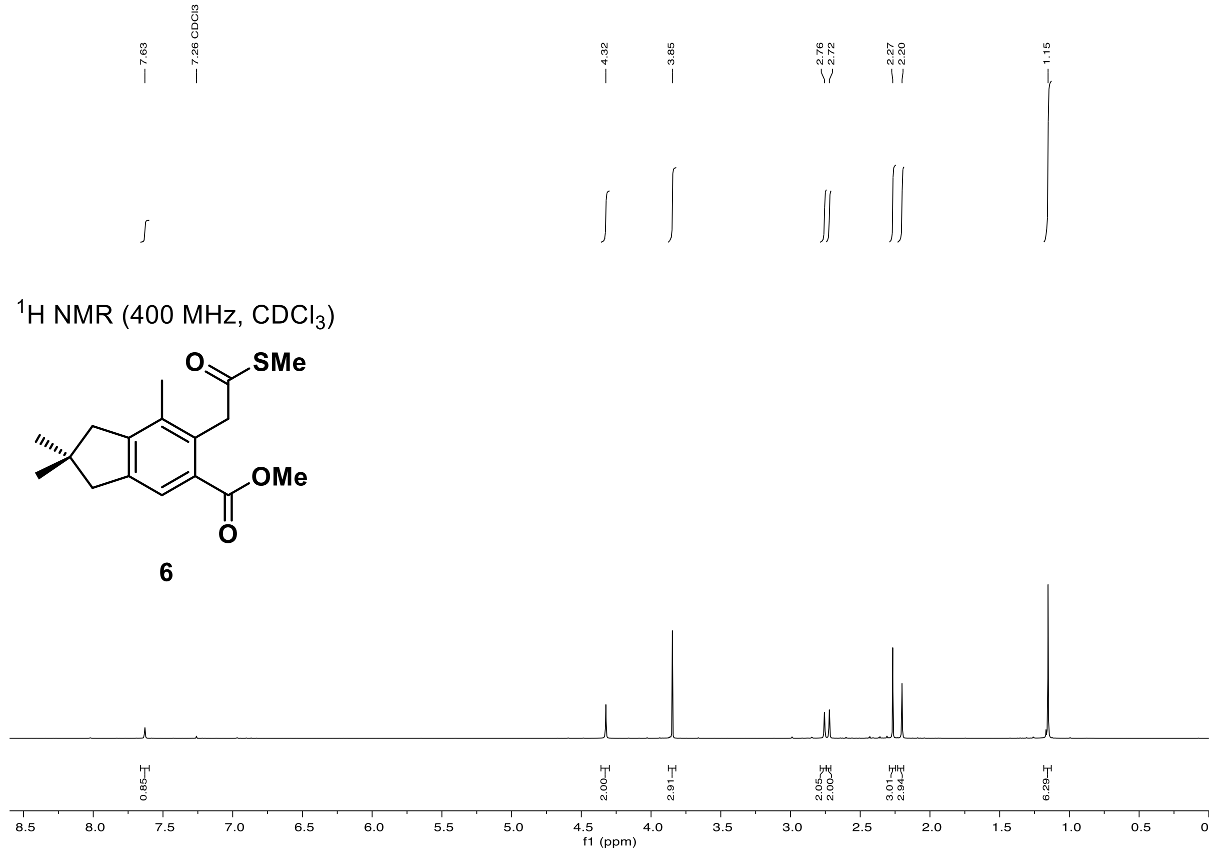
7.63
7.26 CDCl3
4.32
3.85
2.76
2.72
2.27
2.20
1.15
1H NMR (400 MHz, CDCl3)
O
SMe
OMe
O
6
0.85
2.00
2.91
2.05
2.00
3.01
2.94
6.29
8.5 8.0 7.5 7.0 6.5 6.0 5.5 5.0 4.5 4.0 3.5 3.0 2.5 2.0 1.5 1.0 0.5 0
f1 (ppm)

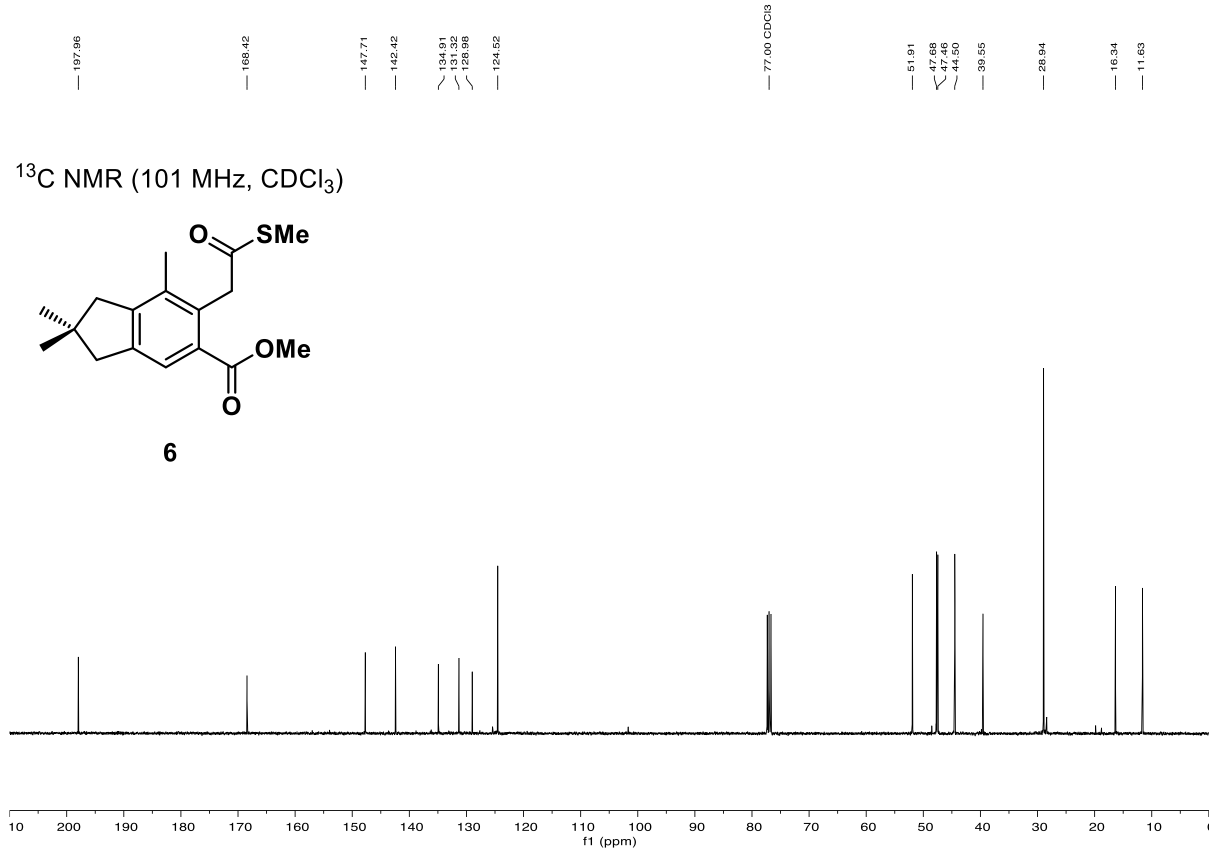
197.96
168.42
147.71
142.42
134.91
131.32
128.98
124.52
77.00 CDCl3
51.91
47.68
47.46
44.50
39.55
28.94
16.34
11.63
13C NMR (101 MHz, CDCl3)
O
SMe
OMe
O
6
200 190 180 170 160 150 140 130 120 110 100 90 80 70 60 50 40 30 20 10
f1 (ppm)

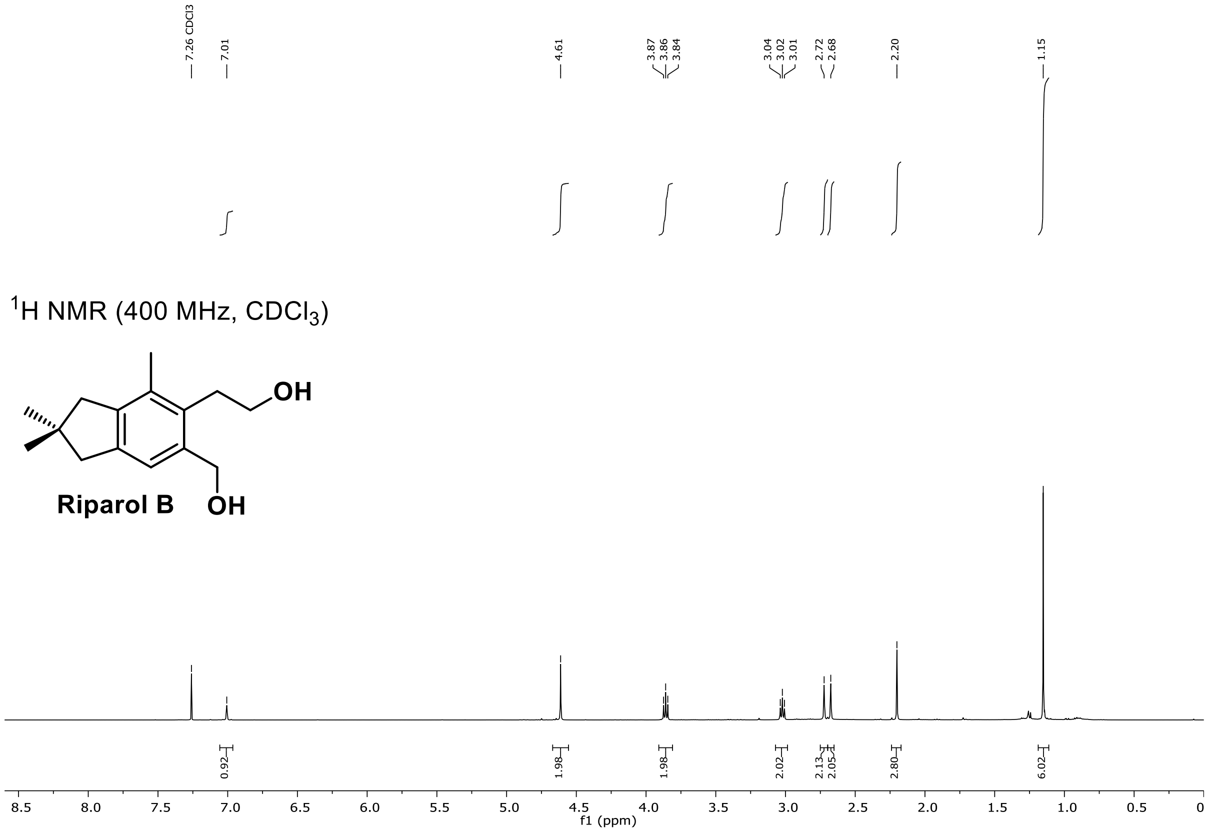
7.26 CDCl3
7.01
4.61
3.87
3.86
3.84
3.04
3.02
3.01
2.72
2.68
2.20
1.15
^{1}H NMR (400 MHz, $CDCl_3$)
OH
Riparol B
OH
0.92
1.98
1.98
2.02
2.13
2.05
2.80
6.02
f1 (ppm)

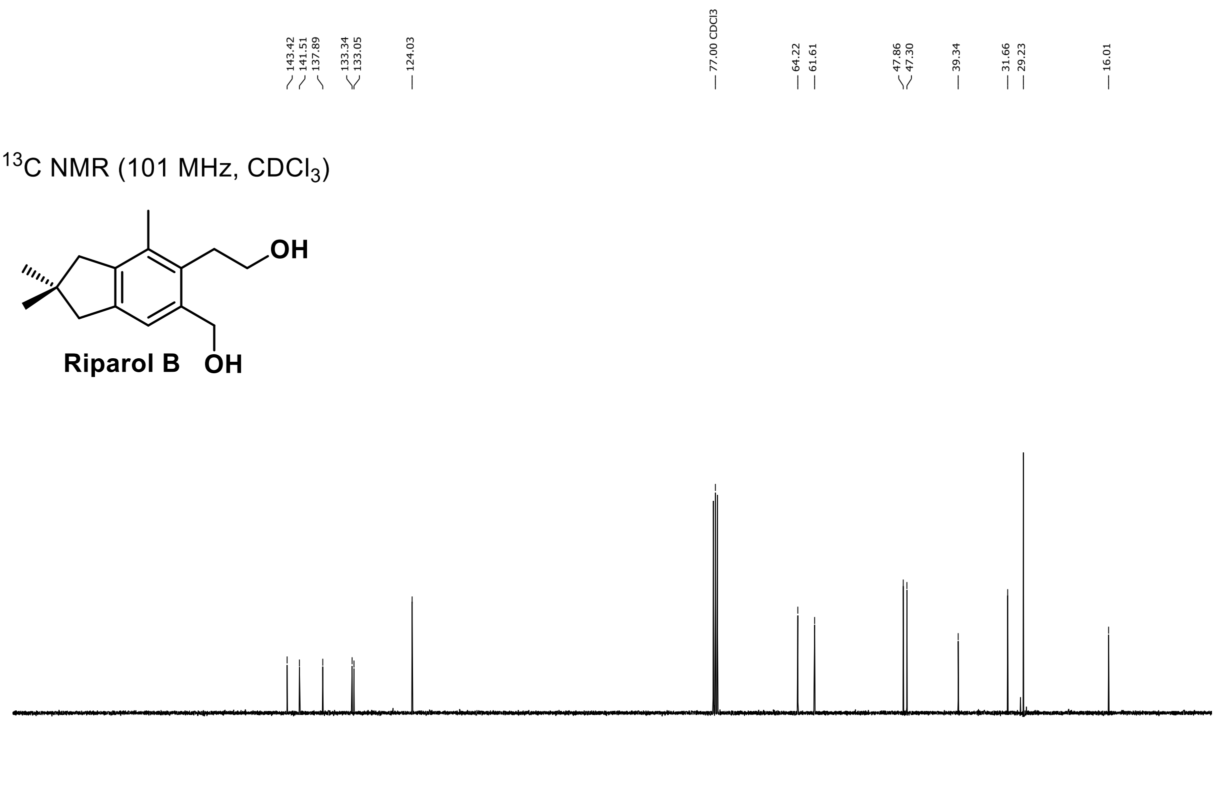
143.42
141.51
137.89
133.34
133.05
124.03
77.00 CDCl3
64.22
61.61
47.86
47.30
39.34
31.66
29.23
16.01
^{13}C NMR (101 MHz, $CDCl_3$)
OH
Riparol B
OH
f1 (ppm)

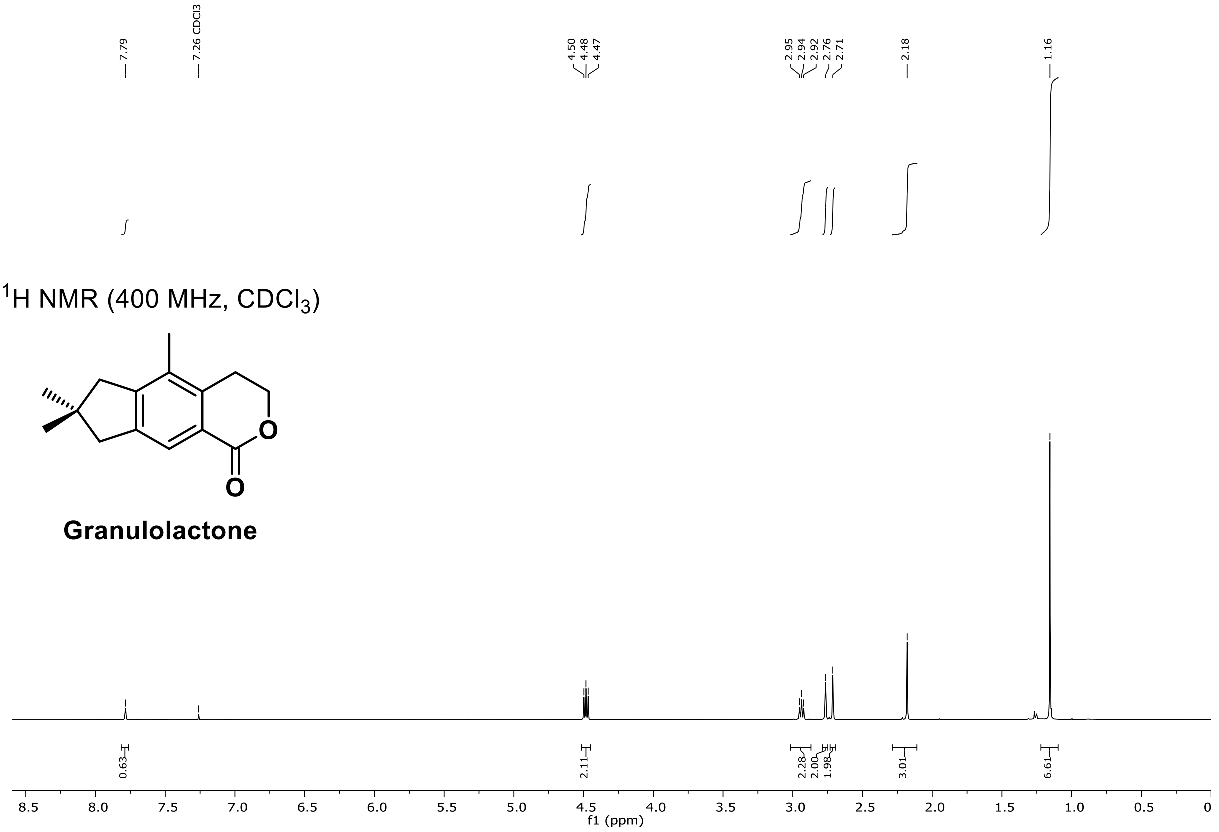
7.79
7.26 CDCl3
4.50
4.48
4.47
2.95
2.94
2.92
2.76
2.71
2.18
1.16
^{1}H NMR (400 MHz, $CDCl_3$)
O
O
Granulolactone
0.63
2.11
2.28
2.00
1.98
3.01
6.61
f1 (ppm)

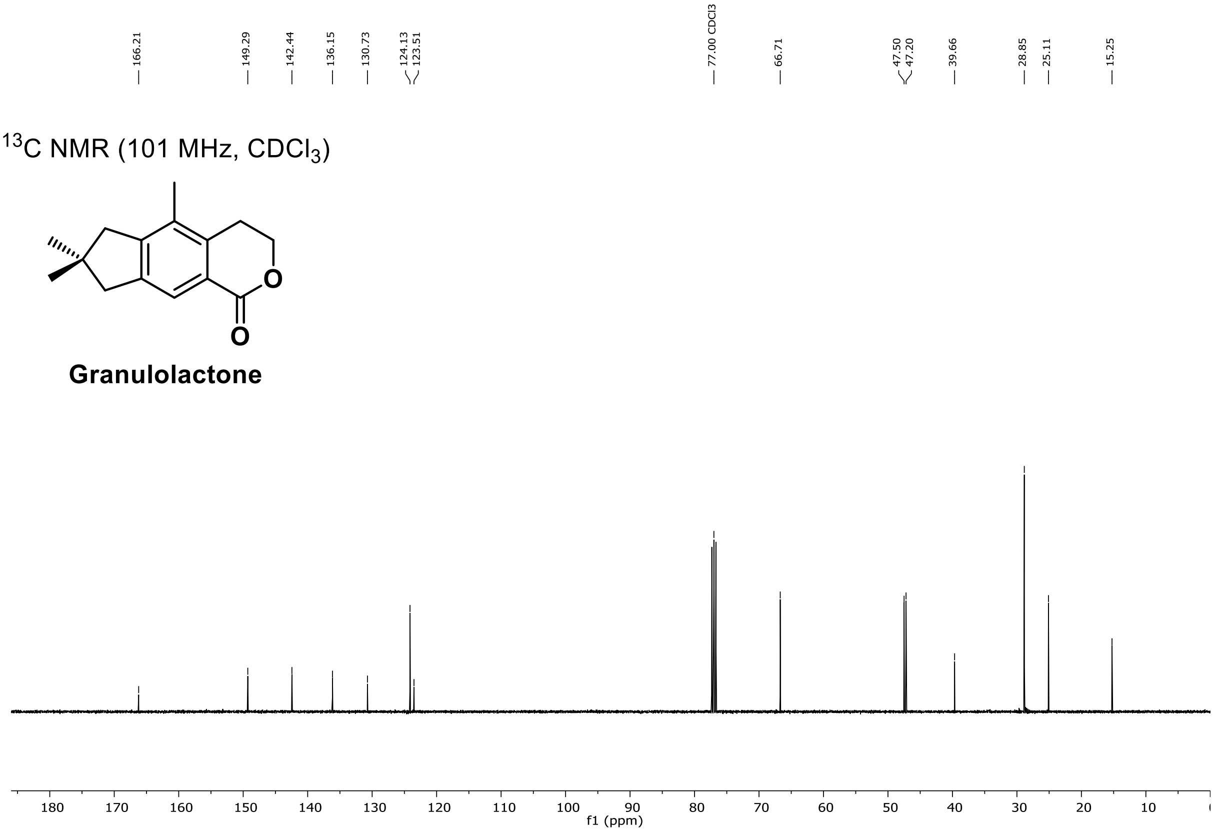
166.21
149.29
142.44
136.15
130.73
124.13
123.51
77.00 CDCl3
66.71
47.50
47.20
39.66
28.85
25.11
15.25
^{13}C NMR (101 MHz, $CDCl_3$)
O
O
Granulolactone
f1 (ppm)

^{1}H NMR (400 MHz, $CDCl_3$)

Echinolactone A

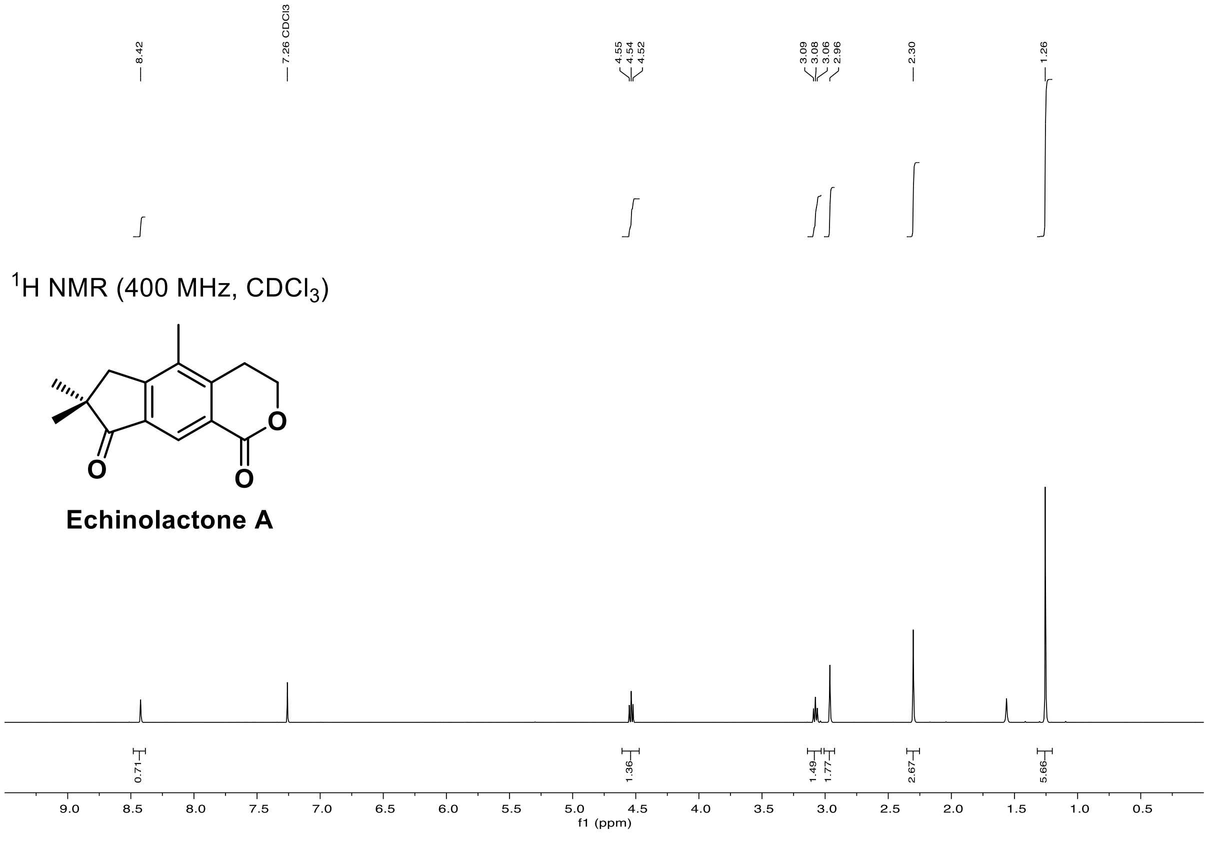

^{13}C NMR (101 MHz, $CDCl_3$)

Echinolactone A

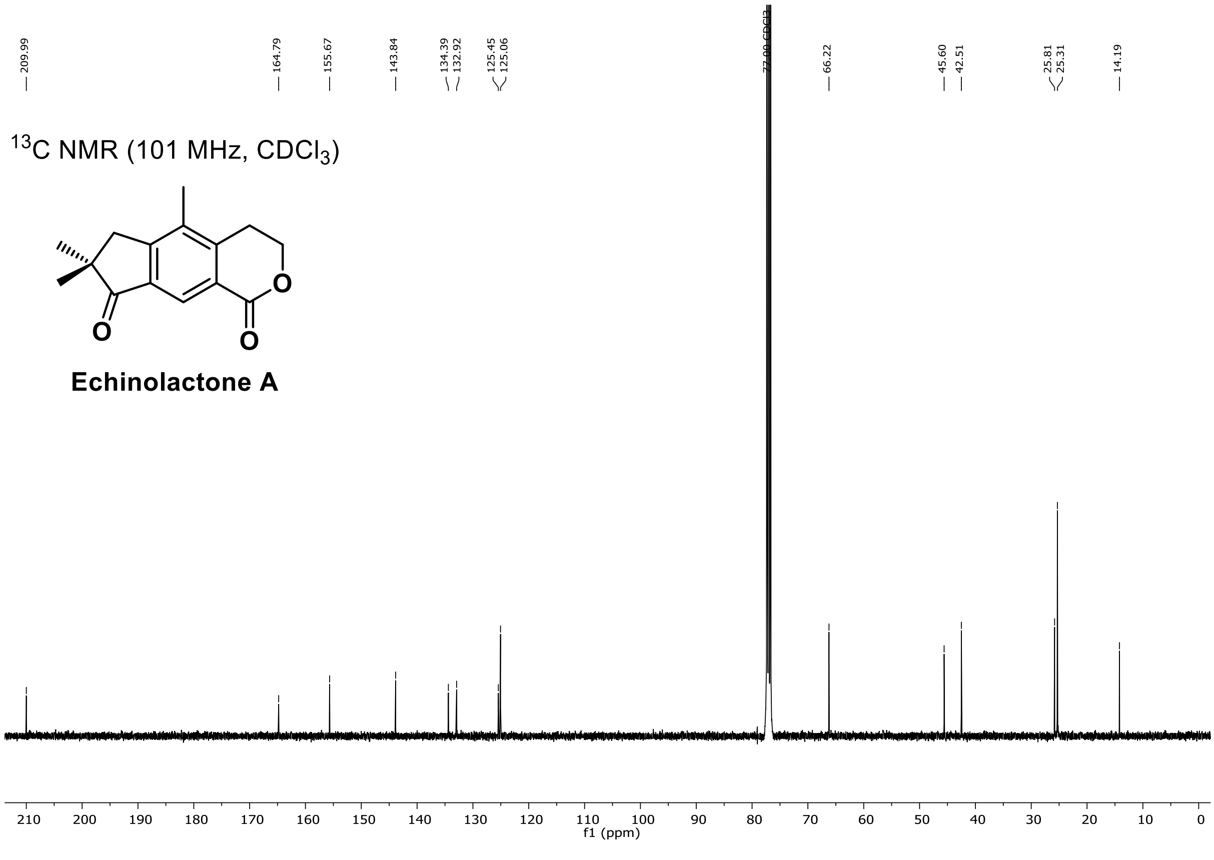

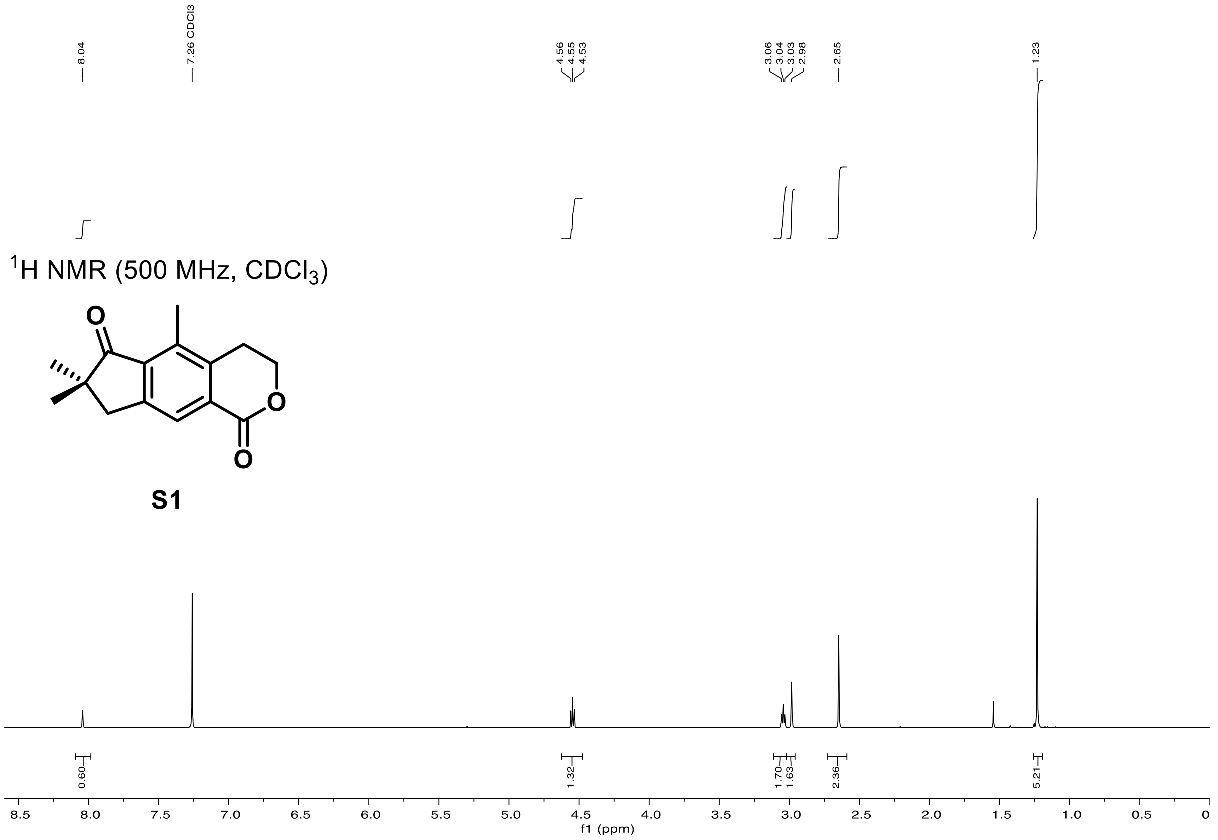
8.04
7.26 CDCl3
4.56
4.55
4.53
3.06
3.04
3.03
2.98
2.65
1.23
^{1}H NMR (500 MHz, $CDCl_3$)
S1
0.60
1.32
1.70
1.63
2.36
5.21
8.5 8.0 7.5 7.0 6.5 6.0 5.5 5.0 4.5 4.0 3.5 3.0 2.5 2.0 1.5 1.0 0.5 0
f1 (ppm)

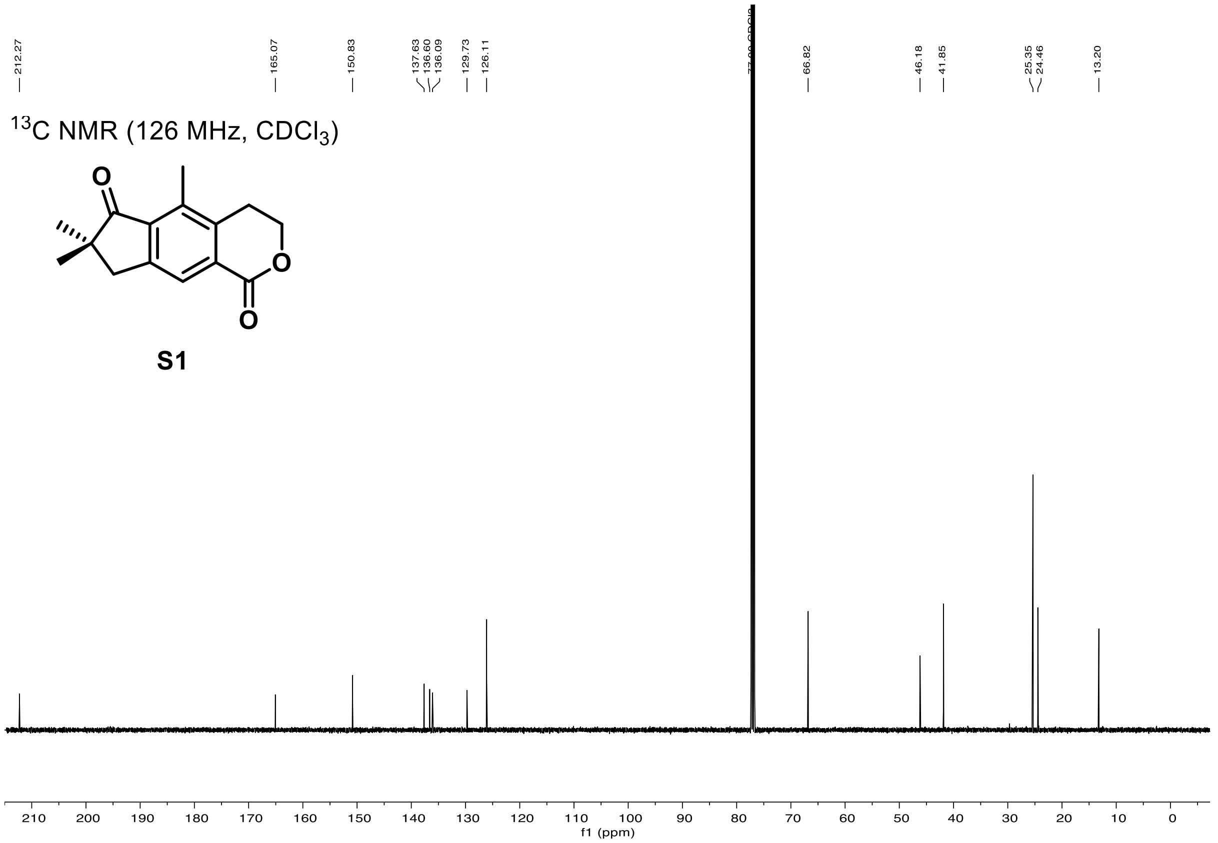
212.27
165.07
150.83
137.63
136.60
136.09
129.73
126.11
66.82
46.18
41.85
25.35
24.46
13.20
^{13}C NMR (126 MHz, $CDCl_3$)
S1
210 200 190 180 170 160 150 140 130 120 110 100 90 80 70 60 50 40 30 20 10 0
f1 (ppm)

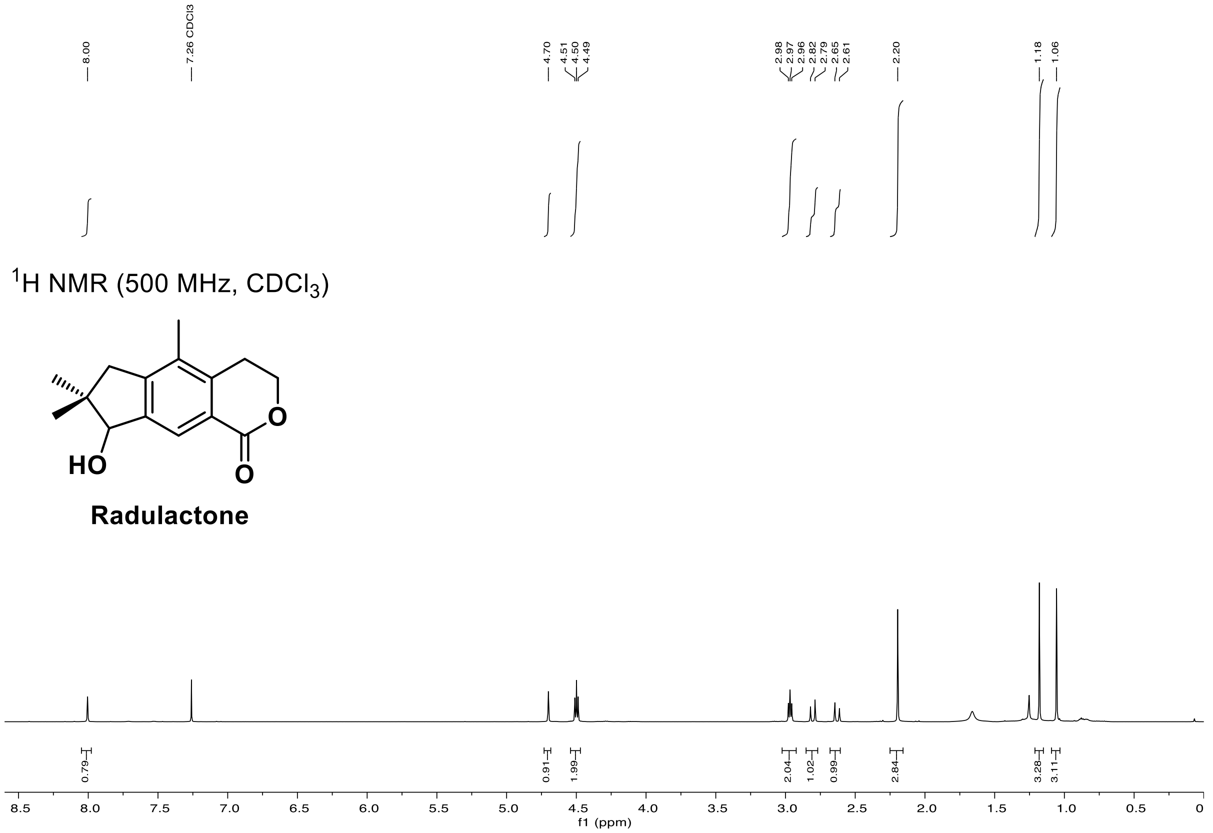

8.00
7.26 CDCl3
4.70
4.51
4.50
4.49
2.98
2.97
2.96
2.82
2.79
2.65
2.61
2.20
1.18
1.06
^{1}H NMR (500 MHz, $CDCl_3$)
HO
O
O
Radulactone
0.79
0.91
1.99
2.04
1.02
0.99
2.84
3.28
3.11
f1 (ppm)

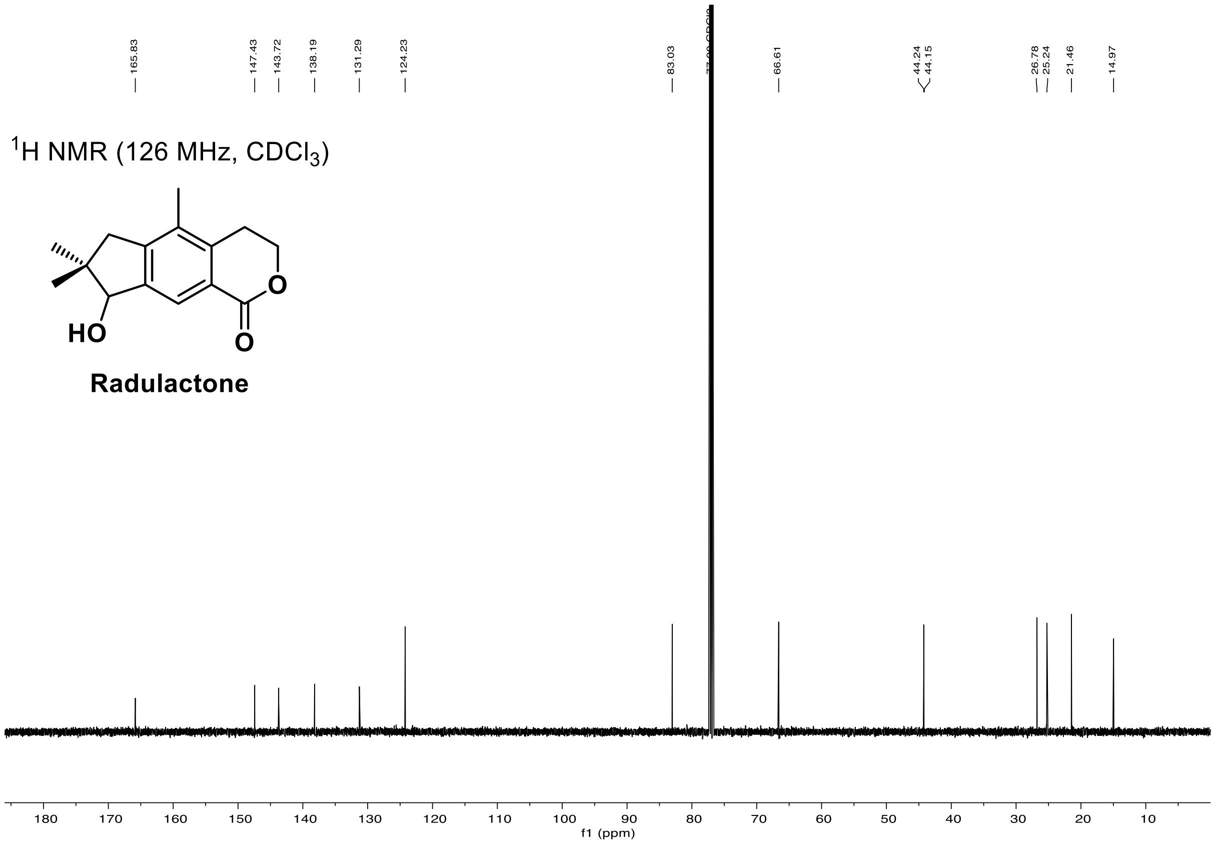

165.83
147.43
143.72
138.19
131.29
124.23
83.03
66.61
44.24
44.15
26.78
25.24
21.46
14.97
^{1}H NMR (126 MHz, $CDCl_3$)
HO
O
O
Radulactone
f1 (ppm)

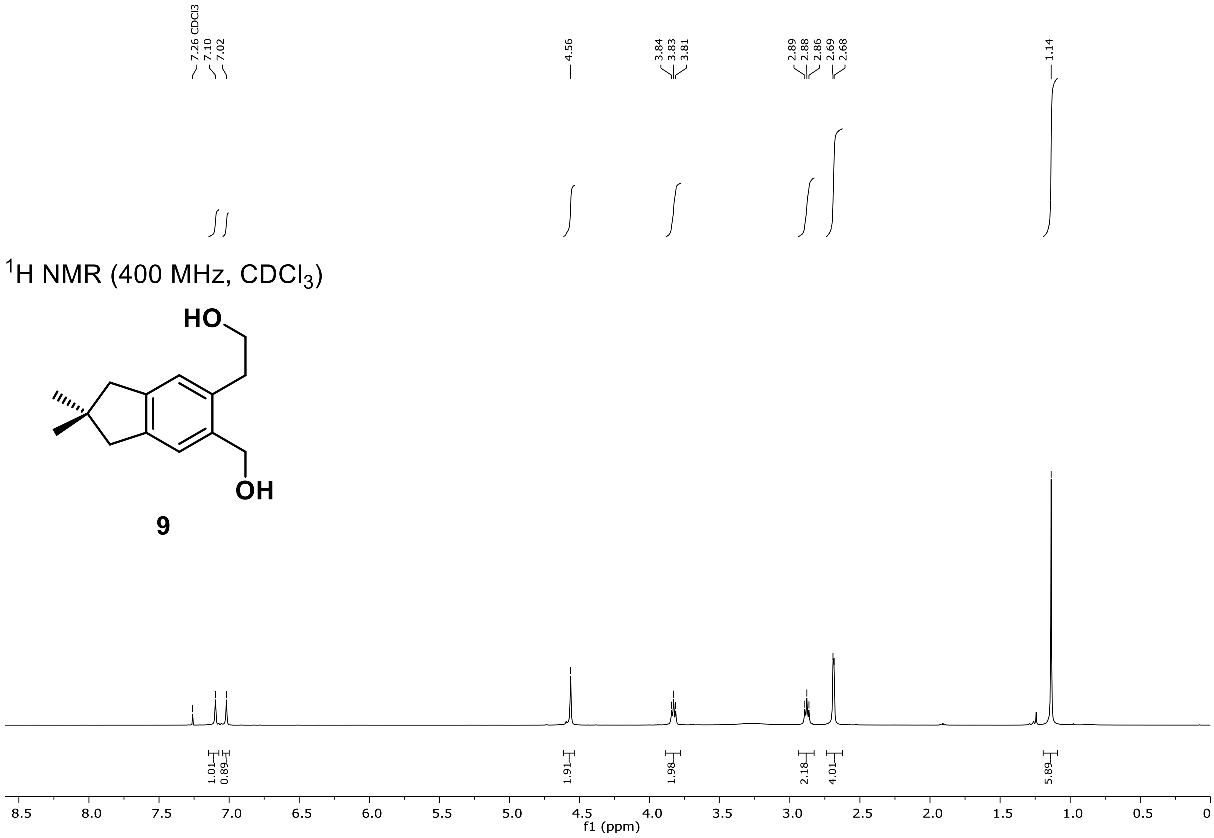

^{1}H NMR (400 MHz, CDCl$_3$)
HO
OH
9
7.26 CDCl3
7.10
7.02
4.56
3.84
3.83
3.81
2.89
2.88
2.86
2.69
2.68
1.14
1.01
0.89
1.91
1.98
2.18
4.01
5.89
f1 (ppm)

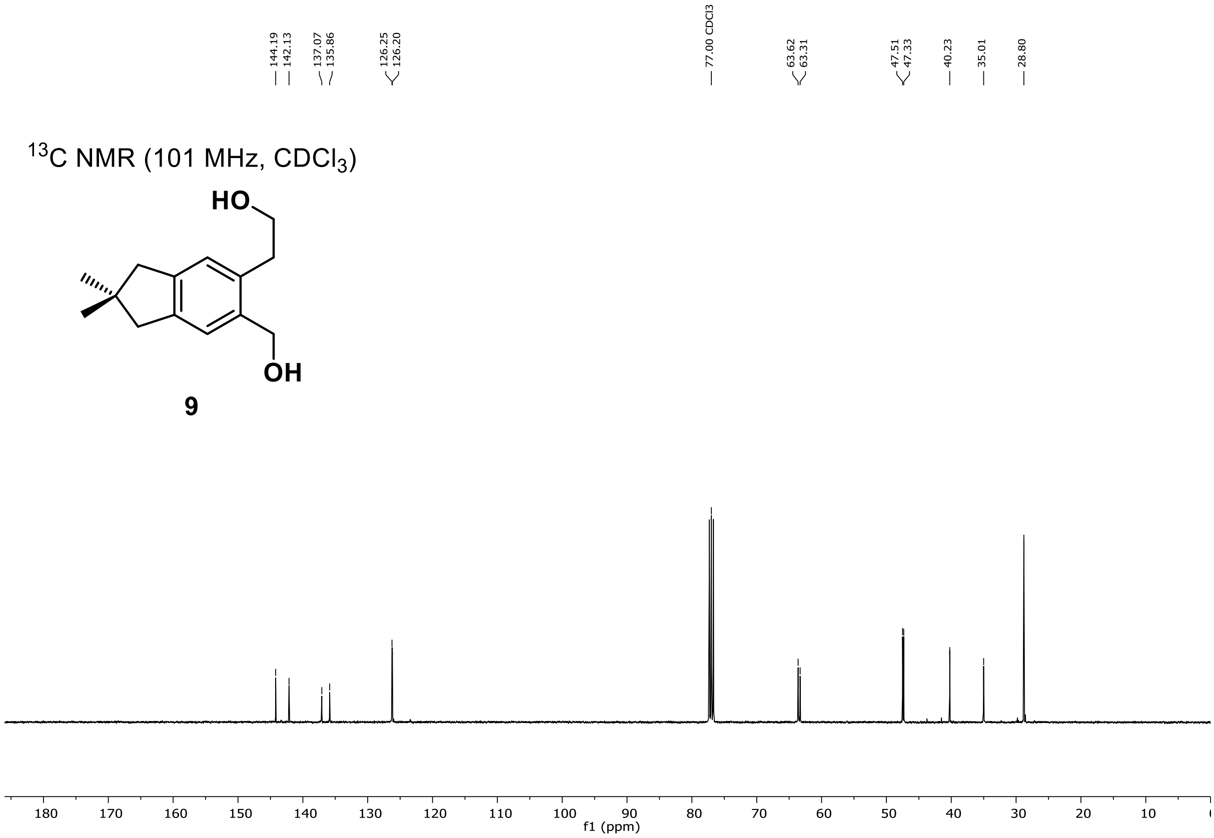

^{13}C NMR (101 MHz, CDCl$_3$)
HO
OH
9
144.19
142.13
137.07
135.86
126.25
126.20
77.00 CDCl3
63.62
63.31
47.51
47.33
40.23
35.01
28.80
f1 (ppm)

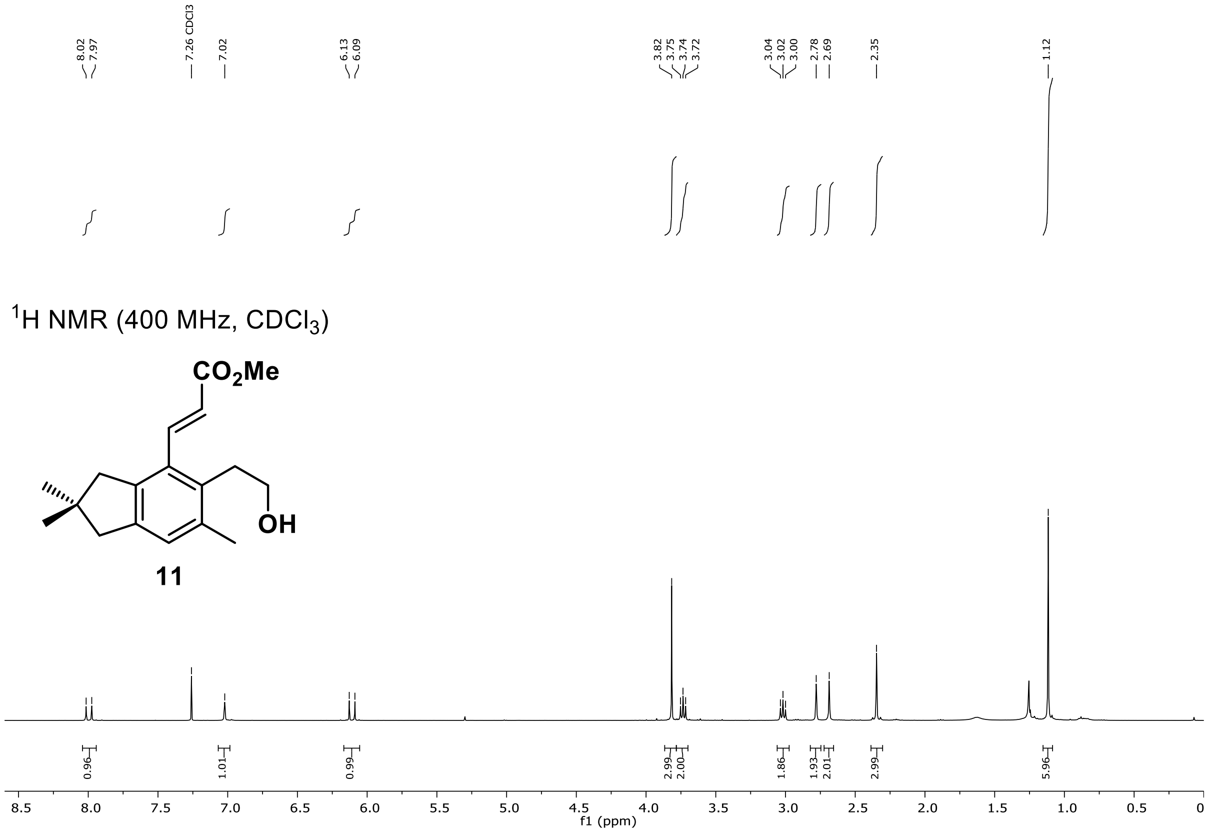

8.02
7.97
7.26 CDCl3
7.02
6.13
6.09
3.82
3.75
3.74
3.72
3.04
3.02
3.00
2.78
2.69
2.35
1.12
^{1}H NMR (400 MHz, $CDCl_3$)
CO_2Me
OH
11
0.96
1.01
0.99
2.99
2.00
1.86
1.93
2.01
2.99
5.96
8.5 8.0 7.5 7.0 6.5 6.0 5.5 5.0 4.5 4.0 3.5 3.0 2.5 2.0 1.5 1.0 0.5 0
f1 (ppm)

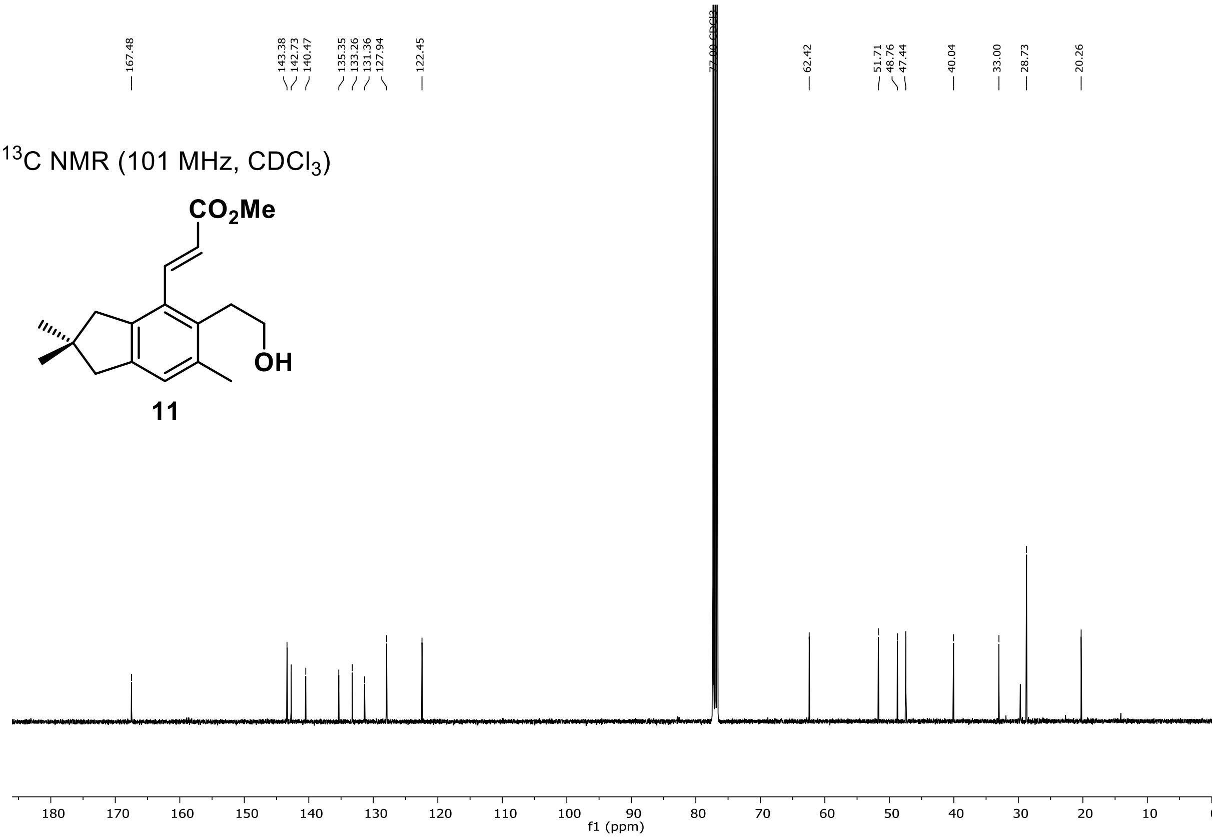

167.48
143.38
142.73
140.47
135.35
133.26
131.36
127.94
122.45
77.00 CDCl3
62.42
51.71
48.76
47.44
40.04
33.00
28.73
20.26
^{13}C NMR (101 MHz, $CDCl_3$)
CO_2Me
OH
11
180 170 160 150 140 130 120 110 100 90 80 70 60 50 40 30 20 10
f1 (ppm)

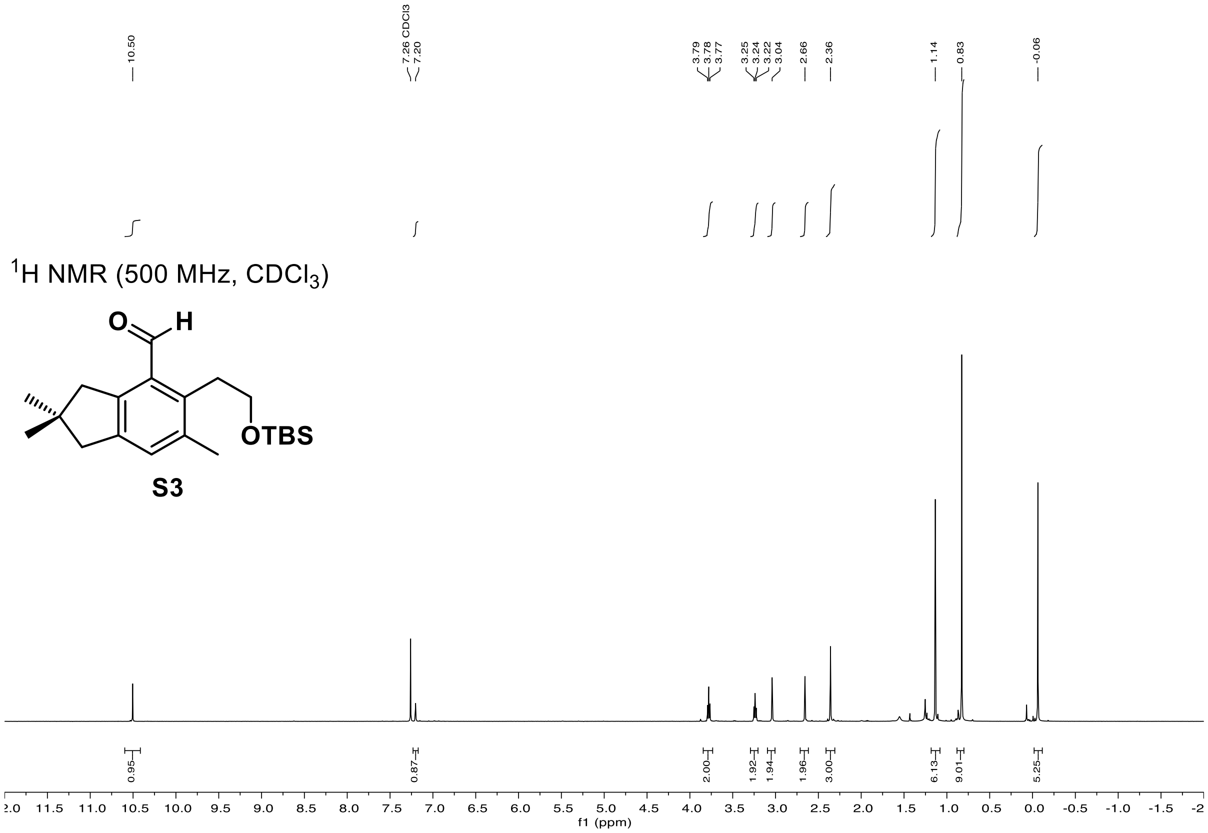

10.50
7.26 CDCl3
7.20
3.79
3.78
3.77
3.25
3.24
3.22
3.04
2.66
2.36
1.14
0.83
-0.06
^{1}H NMR (500 MHz, CDCl$_3$)
O
H
OTBS
S3
0.95
0.87
2.00
1.92
1.94
1.96
3.00
6.13
9.01
5.25
f1 (ppm)

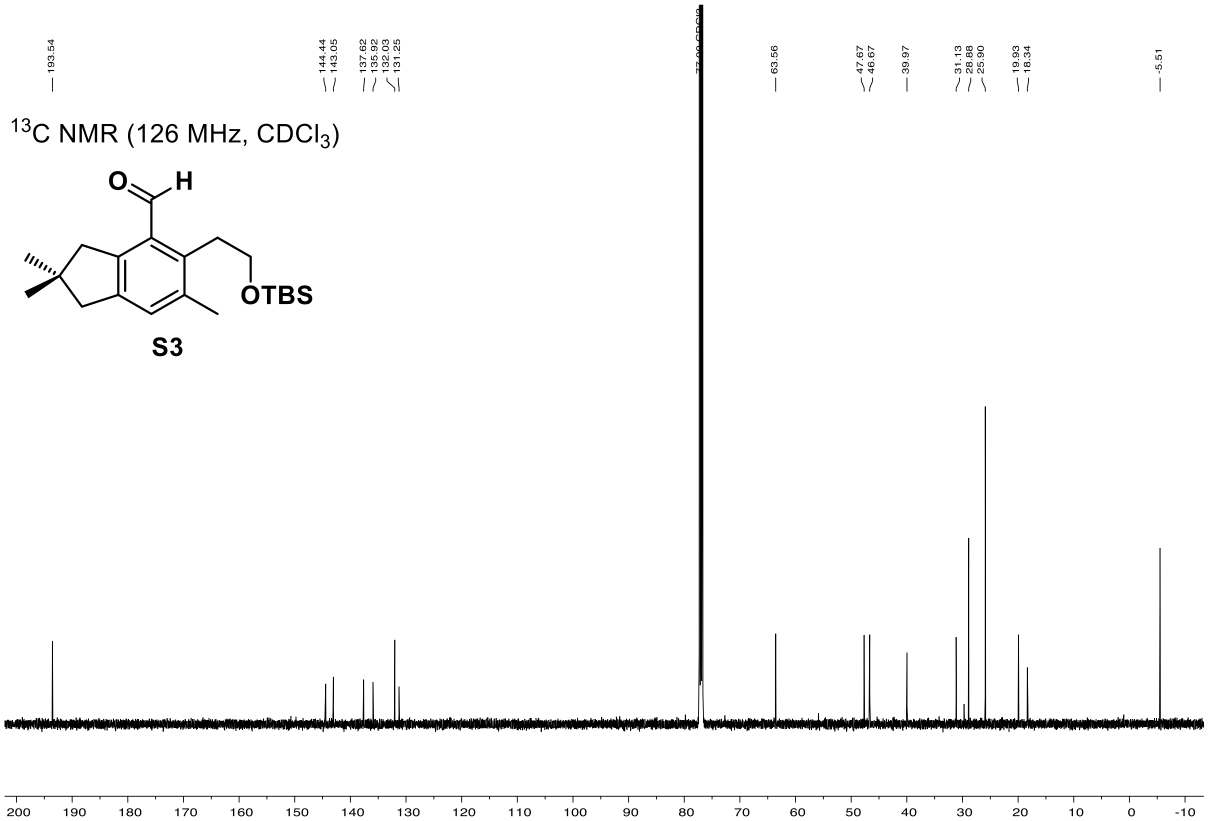

193.54
144.44
143.05
137.62
135.92
132.03
131.25
63.56
47.67
46.67
39.97
31.13
28.88
25.90
19.93
18.34
-5.51
^{13}C NMR (126 MHz, CDCl$_3$)
O
H
OTBS
S3
f1 (ppm)

^1H NMR (400 MHz, $CDCl_3$)

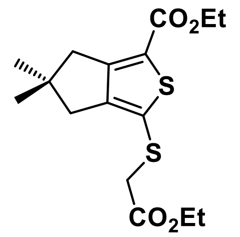

13

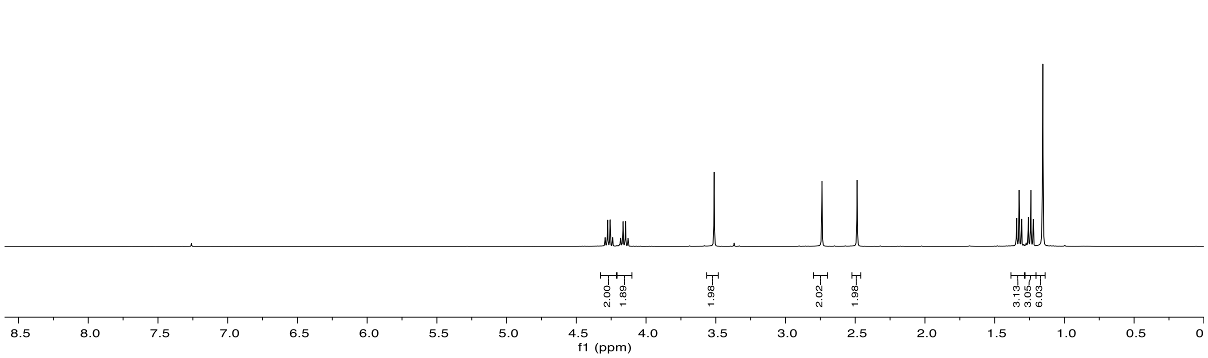

^{13}C NMR (101 MHz, $CDCl_3$)

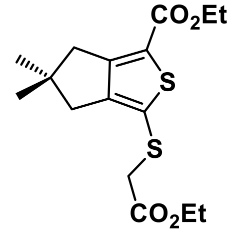

13

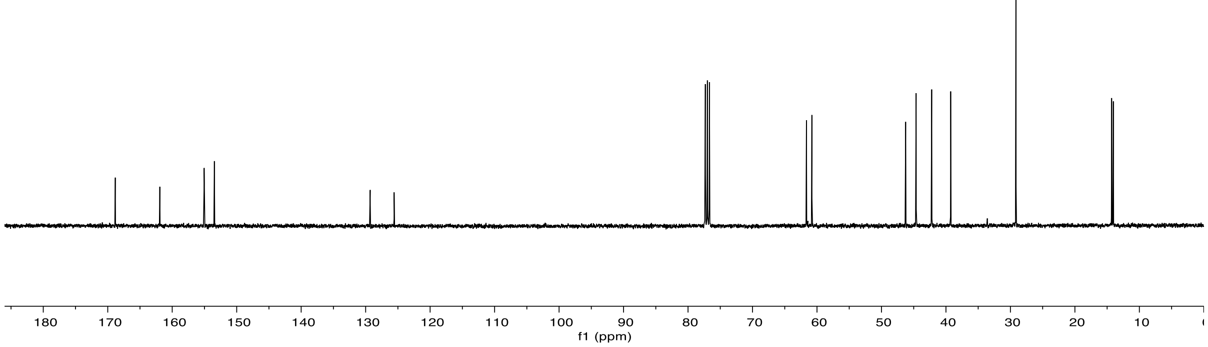

^{1}H NMR (400 MHz, $CDCl_3$)

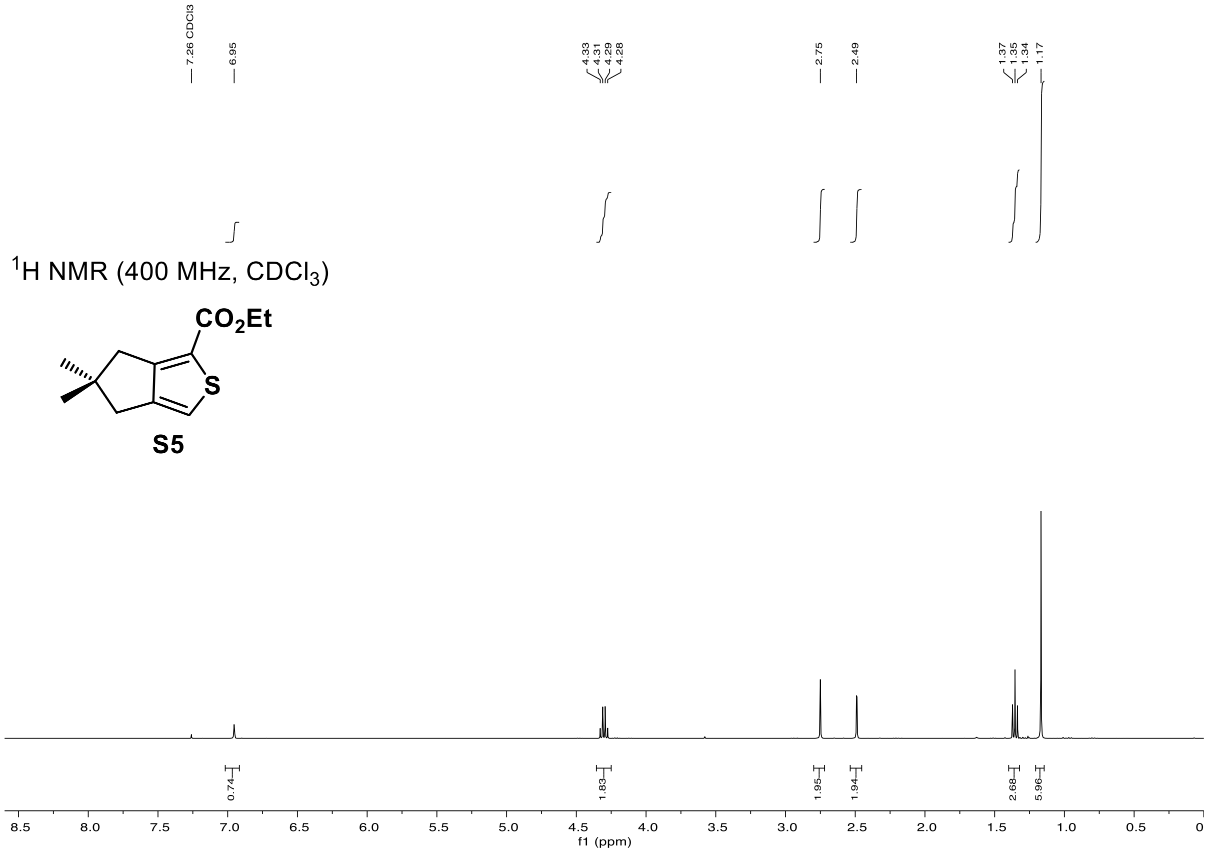

^{13}C NMR (101 MHz, $CDCl_3$)

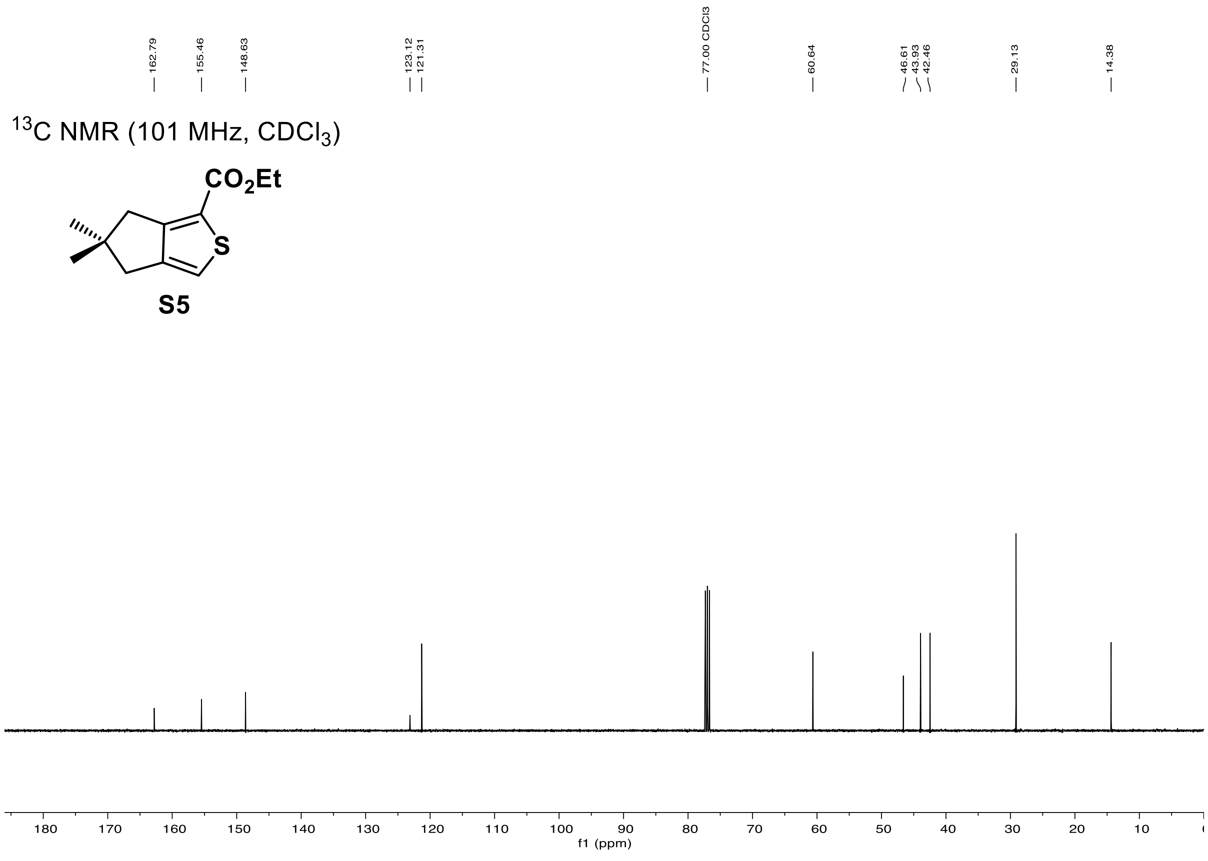

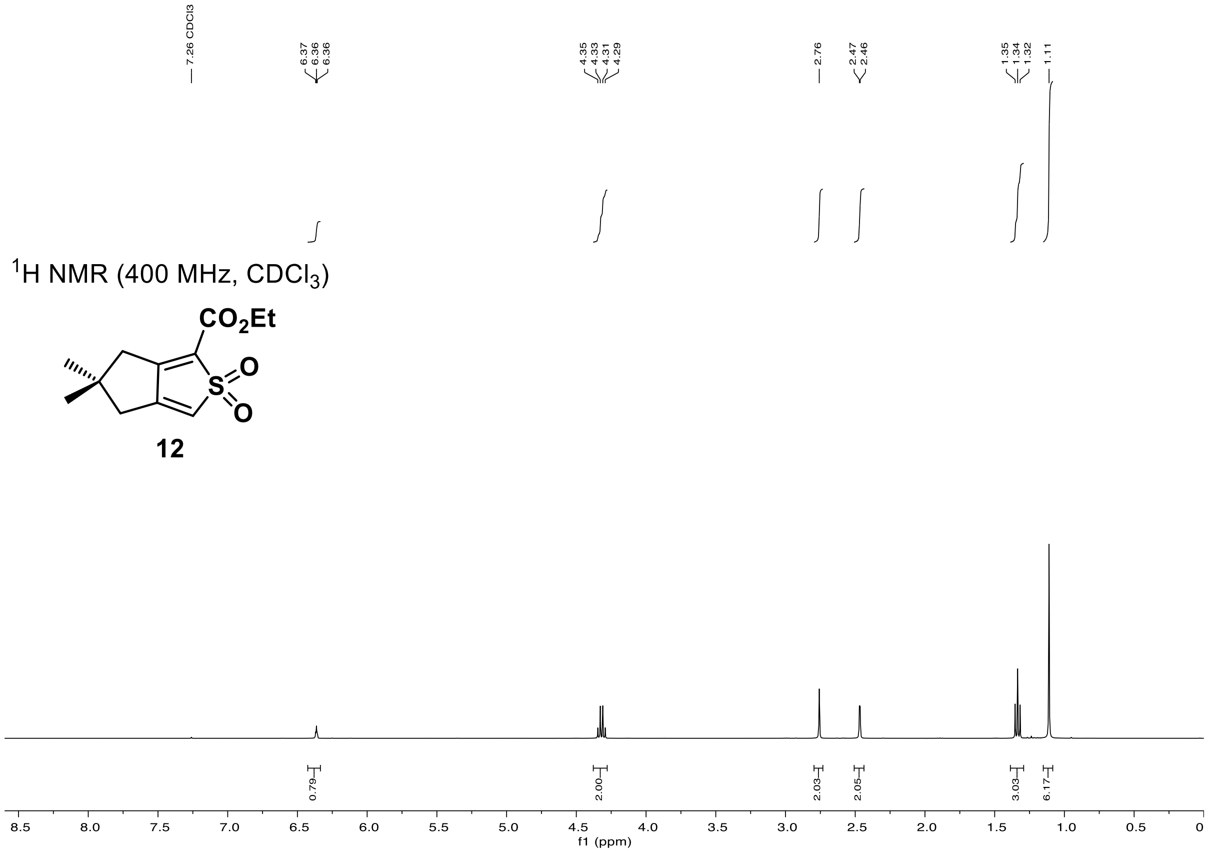

7.26 CDCl3
6.37
6.36
6.36
4.35
4.33
4.31
4.29
2.76
2.47
2.46
1.35
1.34
1.32
1.11
^{1}H NMR (400 MHz, CDCl$_3$)
CO$_2$Et
S
O
O
12
0.79
2.00
2.03
2.05
3.03
6.17
8.5 8.0 7.5 7.0 6.5 6.0 5.5 5.0 4.5 4.0 3.5 3.0 2.5 2.0 1.5 1.0 0.5 0
f1 (ppm)

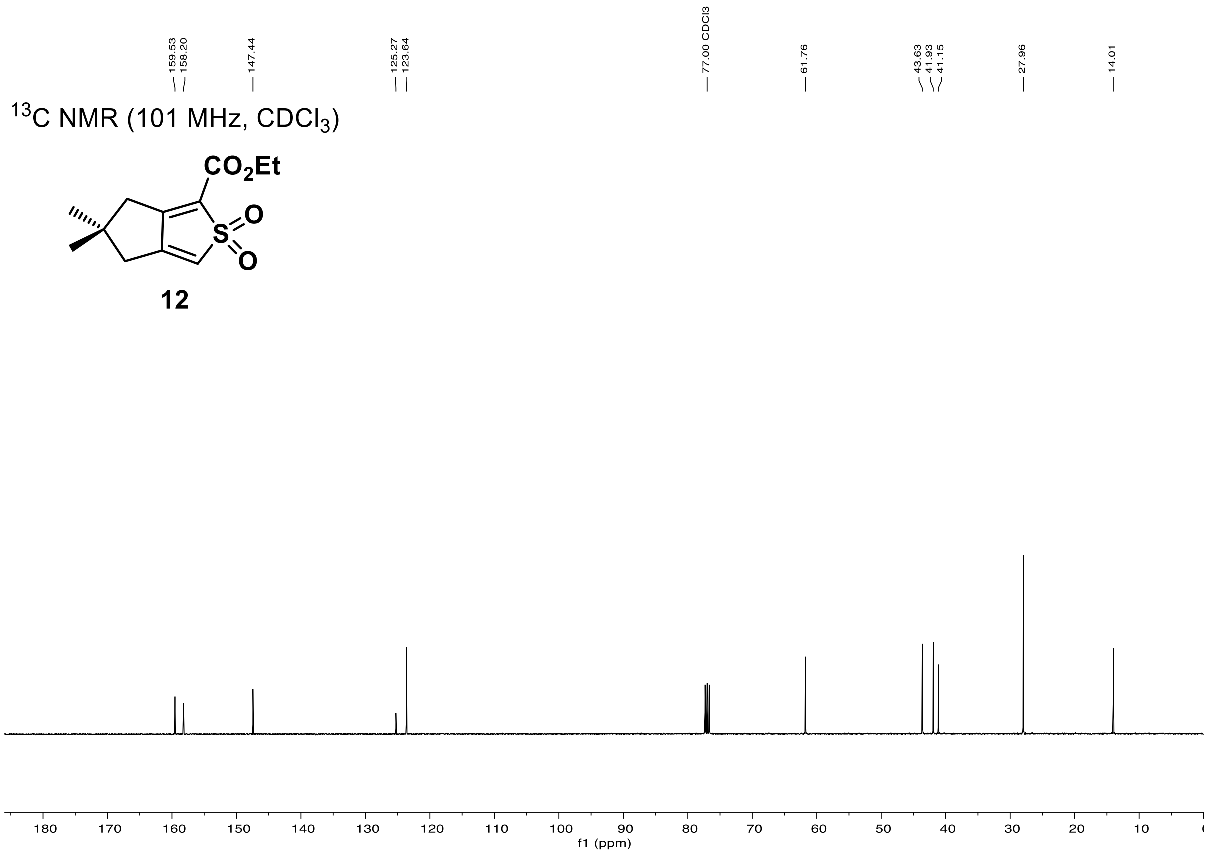

159.53
158.20
147.44
125.27
123.64
77.00 CDCl3
61.76
43.63
41.93
41.15
27.96
14.01
^{13}C NMR (101 MHz, CDCl$_3$)
CO$_2$Et
S
O
O
12
180 170 160 150 140 130 120 110 100 90 80 70 60 50 40 30 20 10
f1 (ppm)

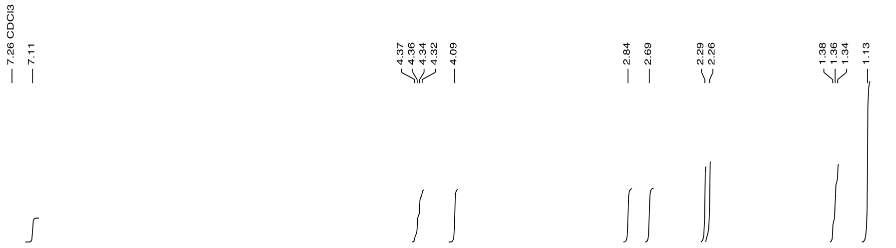

^{1}H NMR (400 MHz, $CDCl_3$)

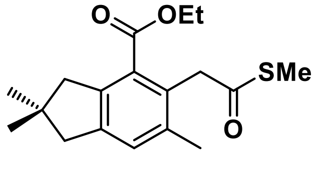

14

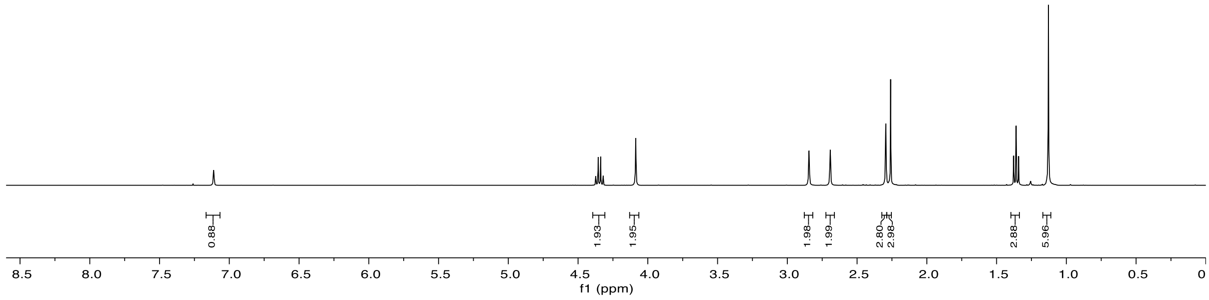

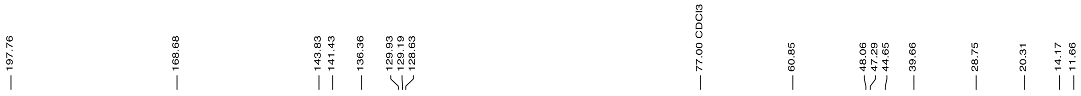

^{13}C NMR (101 MHz, $CDCl_3$)

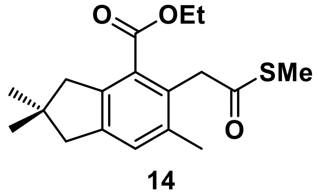

14

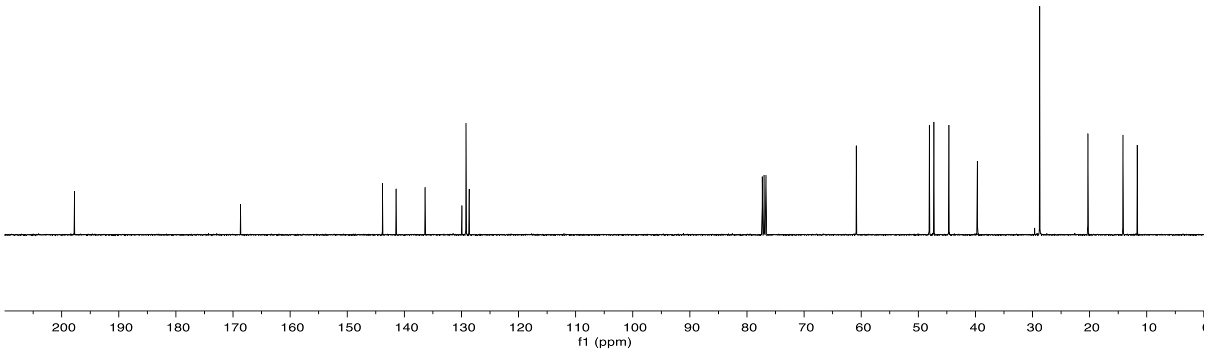

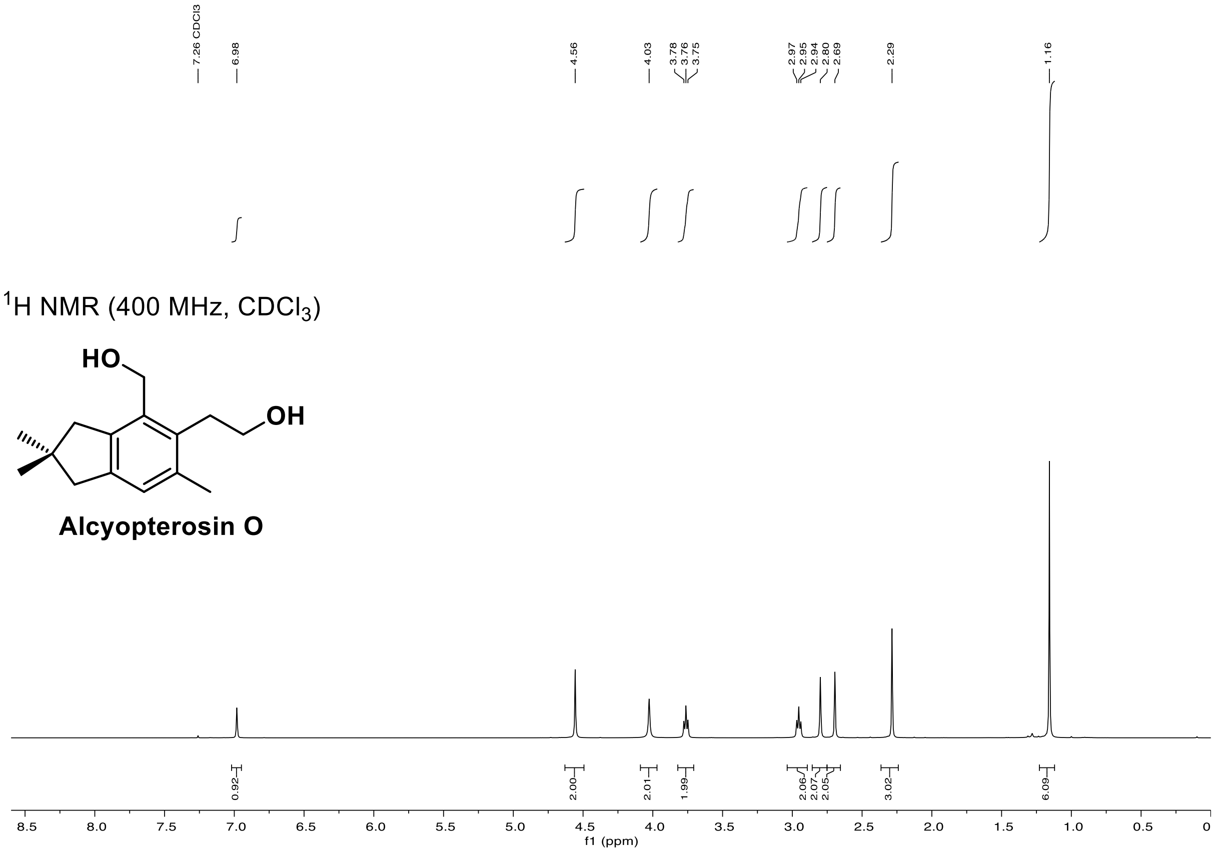

^{1}H NMR (400 MHz, $CDCl_3$)

Alcyopterosin O

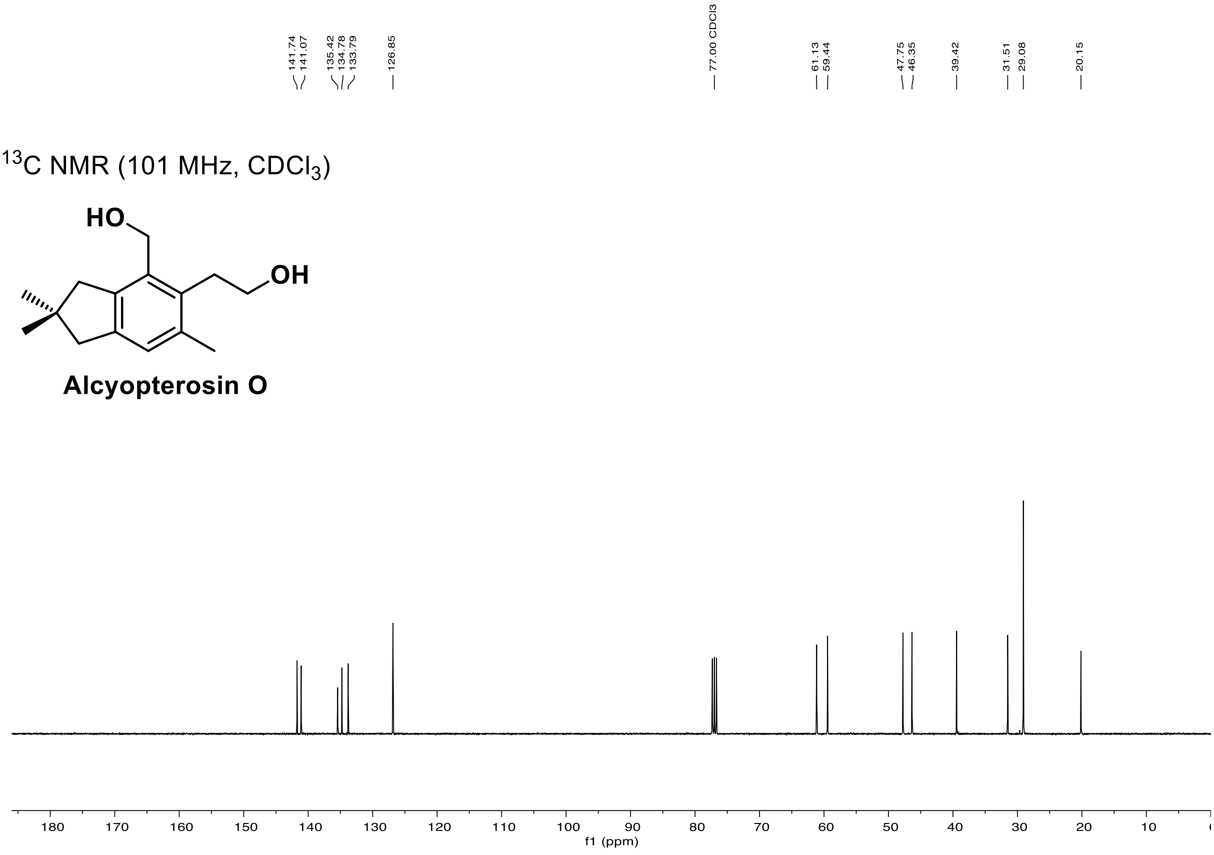

^{13}C NMR (101 MHz, $CDCl_3$)

Alcyopterosin O

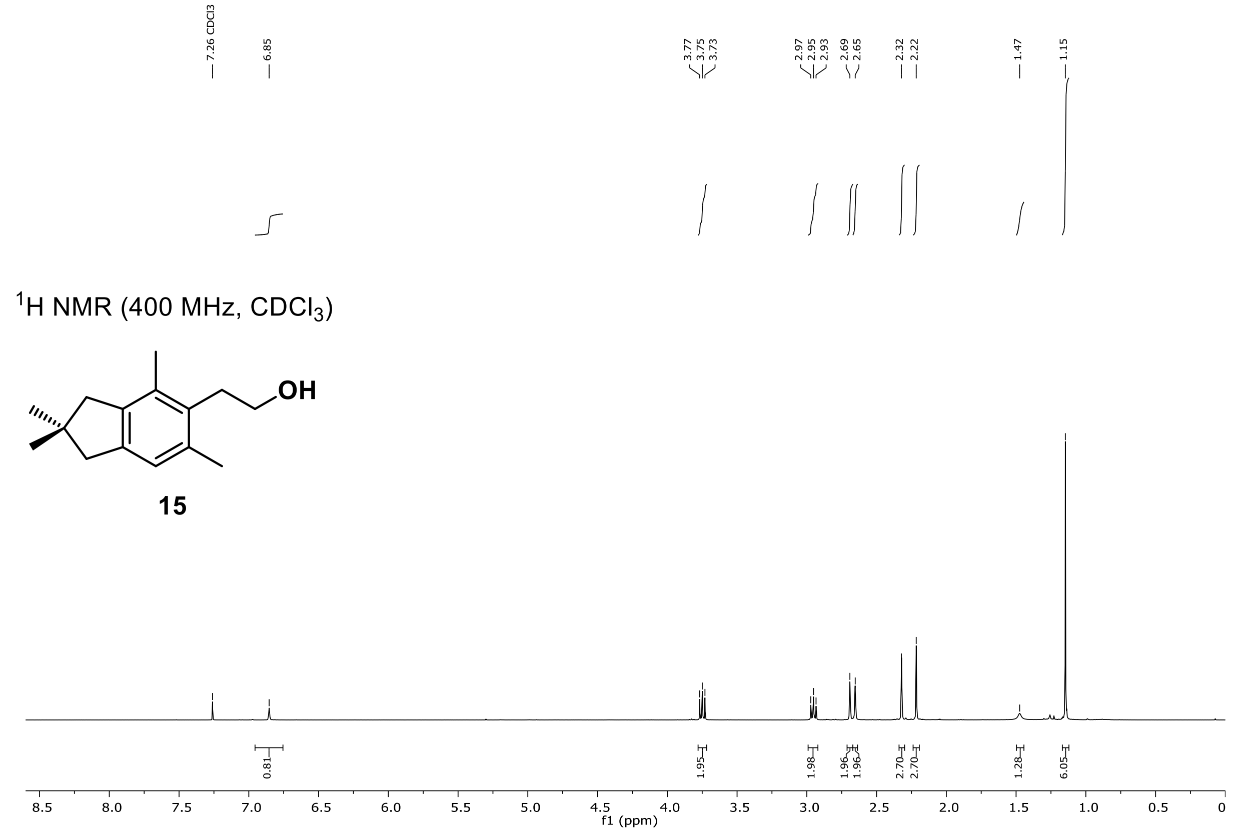
7.26 CDCl3
6.85
3.77
3.75
3.73
2.97
2.95
2.93
2.69
2.65
2.32
2.22
1.47
1.15
1H NMR (400 MHz, CDCl3)
OH
15
0.81
1.95
1.98
1.96
1.96
2.70
2.70
1.28
6.05
8.5 8.0 7.5 7.0 6.5 6.0 5.5 5.0 4.5 4.0 3.5 3.0 2.5 2.0 1.5 1.0 0.5 0
f1 (ppm)

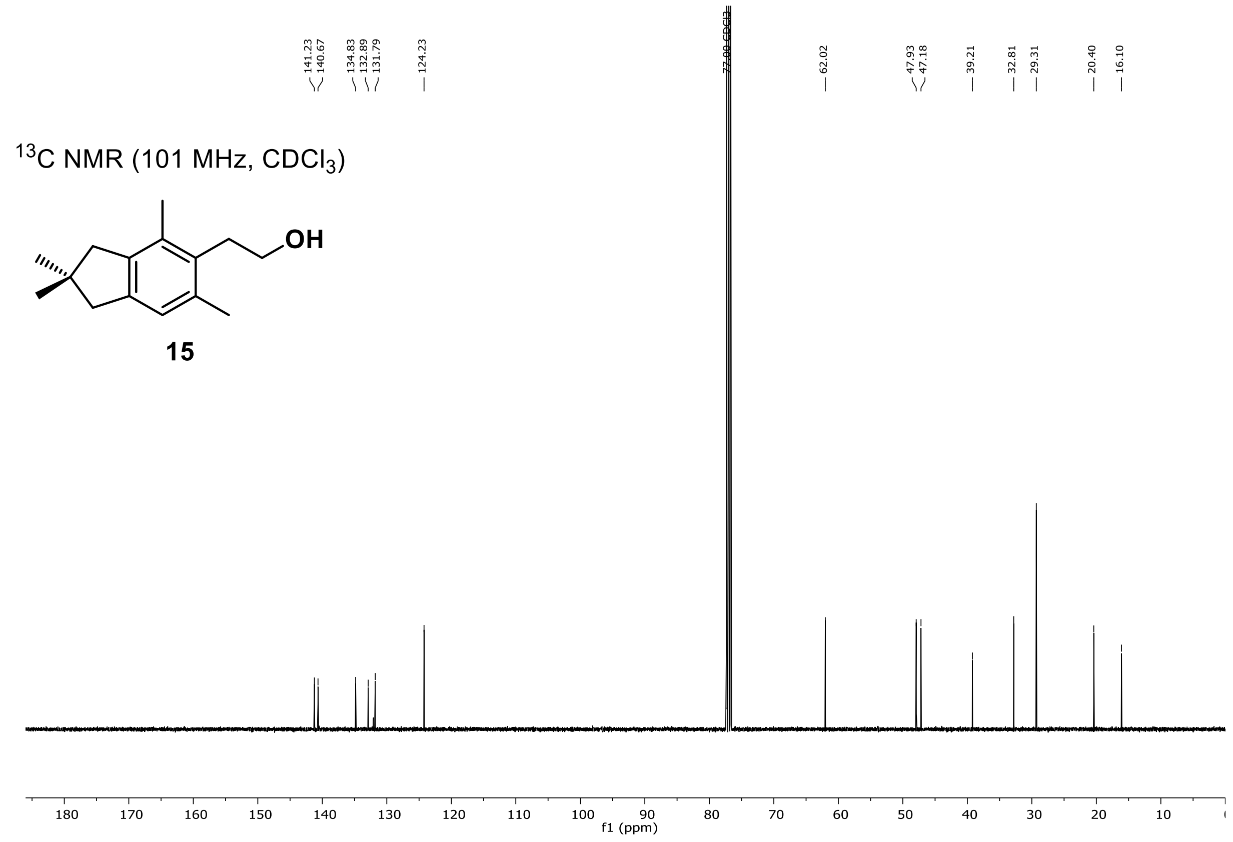
141.23
140.67
134.83
132.89
131.79
124.23
77.00 CDCl3
62.02
47.93
47.18
39.21
32.81
29.31
20.40
16.10
13C NMR (101 MHz, CDCl3)
OH
15
180 170 160 150 140 130 120 110 100 90 80 70 60 50 40 30 20 10
f1 (ppm)

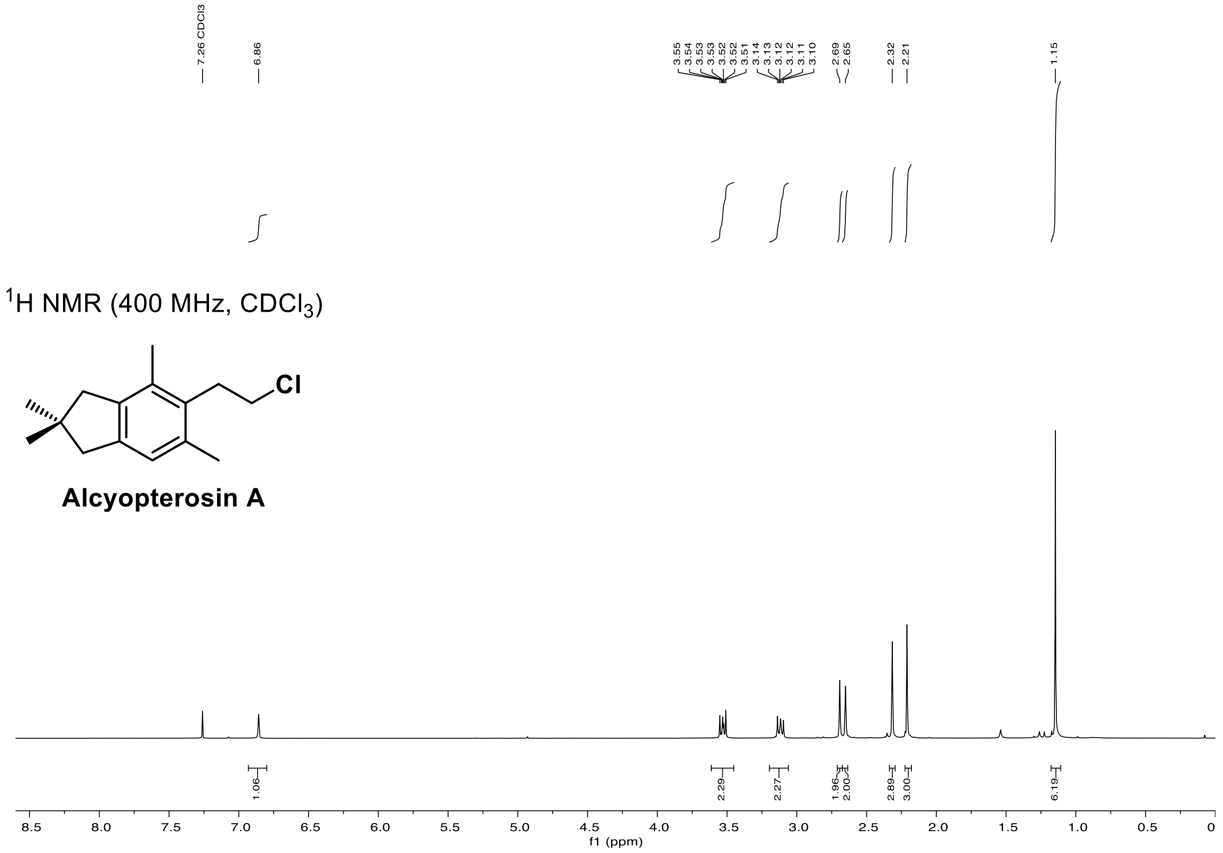

7.26 CDCl3
6.86
3.55
3.54
3.53
3.53
3.52
3.52
3.51
3.14
3.13
3.12
3.12
3.11
3.10
2.69
2.65
2.32
2.21
1.15
^{1}H NMR (400 MHz, $CDCl_3$)
Cl
Alcyopterosin A
1.06
2.29
2.27
1.96
2.00
2.89
3.00
6.19
f1 (ppm)

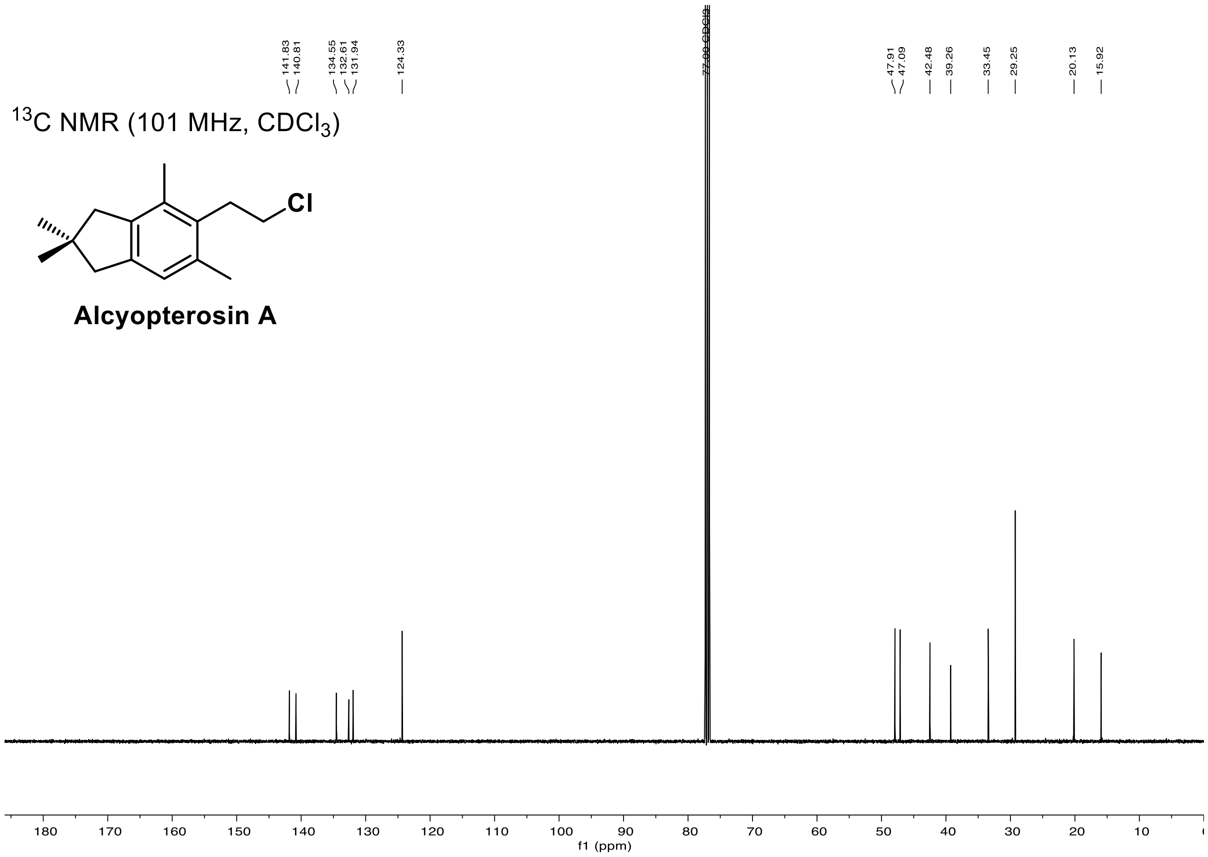

141.83
140.81
134.55
132.61
131.94
124.33
77.00 CDCl3
47.91
47.09
42.48
39.26
33.45
29.25
20.13
15.92
^{13}C NMR (101 MHz, $CDCl_3$)
Cl
Alcyopterosin A
f1 (ppm)

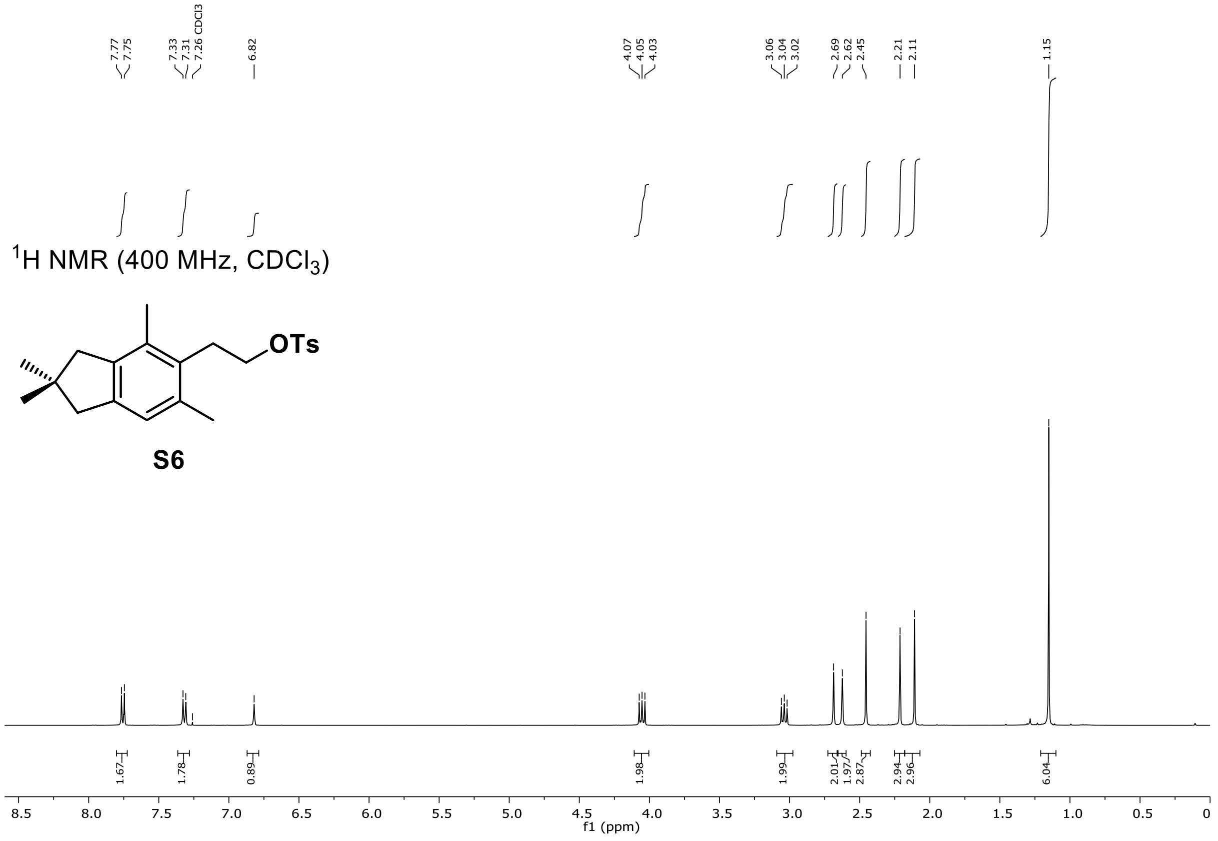
7.77
7.75
7.33
7.31
7.26 CDCl3
6.82
4.07
4.05
4.03
3.06
3.04
3.02
2.69
2.62
2.45
2.21
2.11
1.15
^{1}H NMR (400 MHz, $CDCl_3$)
OTs
S6
1.67
1.78
0.89
1.98
1.99
2.01
1.97
2.87
2.94
2.96
6.04
f1 (ppm)

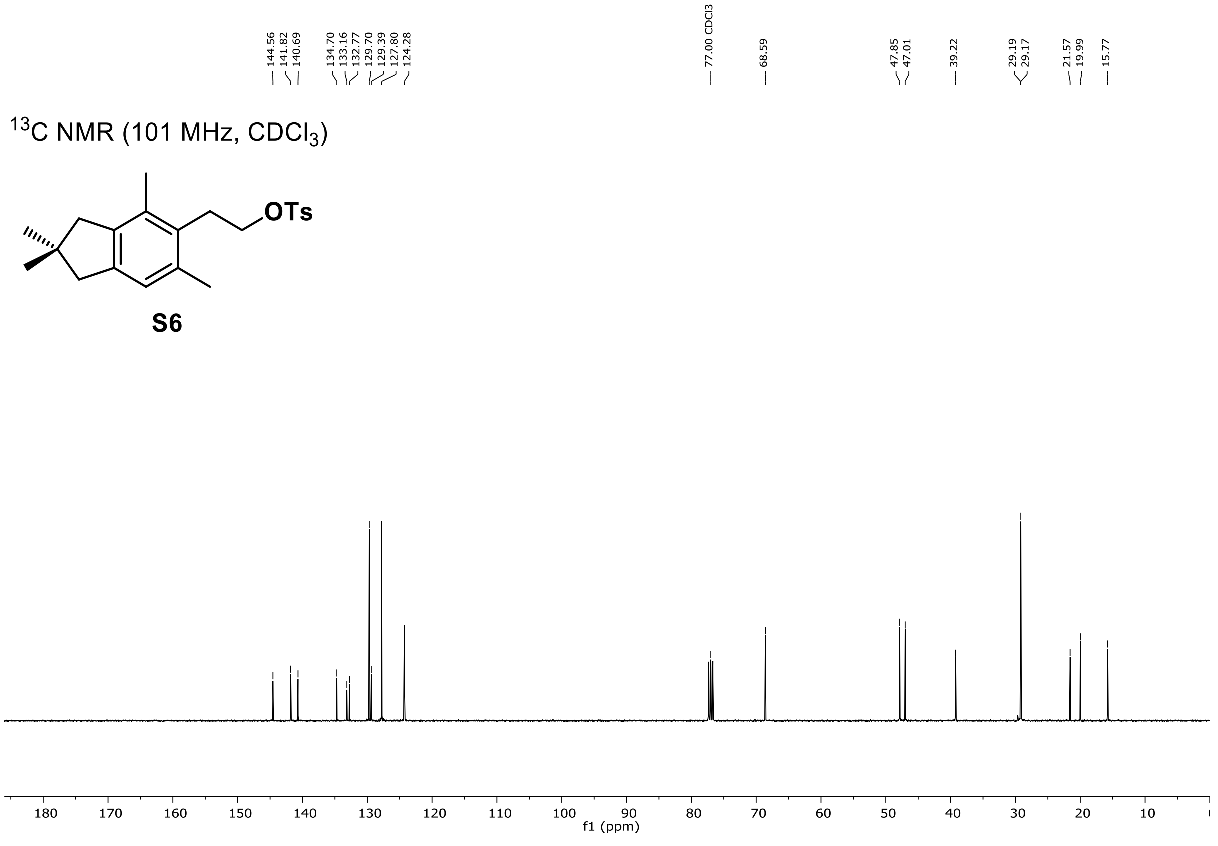
144.56
141.82
140.69
134.70
133.16
132.77
129.70
129.39
127.80
124.28
77.00 CDCl3
68.59
47.85
47.01
39.22
29.19
29.17
21.57
19.99
15.77
^{13}C NMR (101 MHz, $CDCl_3$)
OTs
S6
f1 (ppm)

^{1}H NMR (400 MHz, $CDCl_3$)

Alcyopterosin B

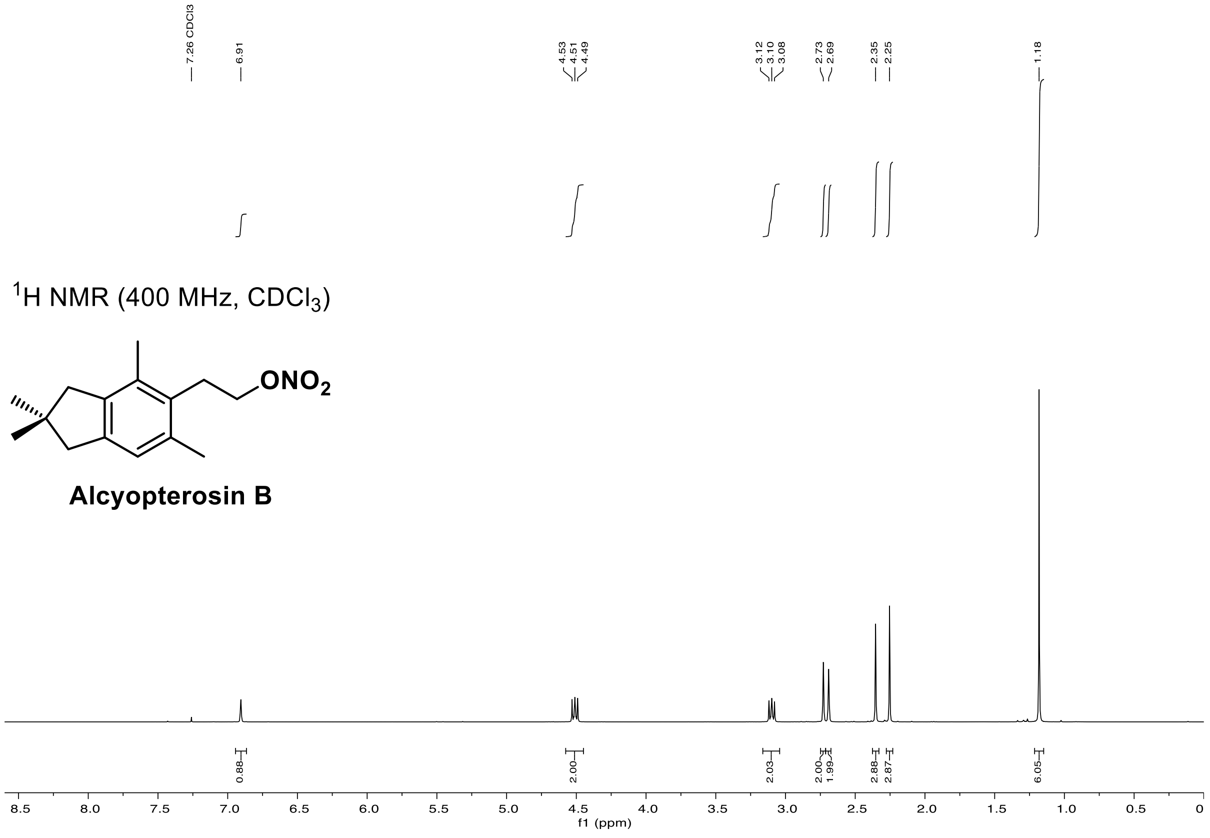

^{13}C NMR (101 MHz, $CDCl_3$)

Alcyopterosin B

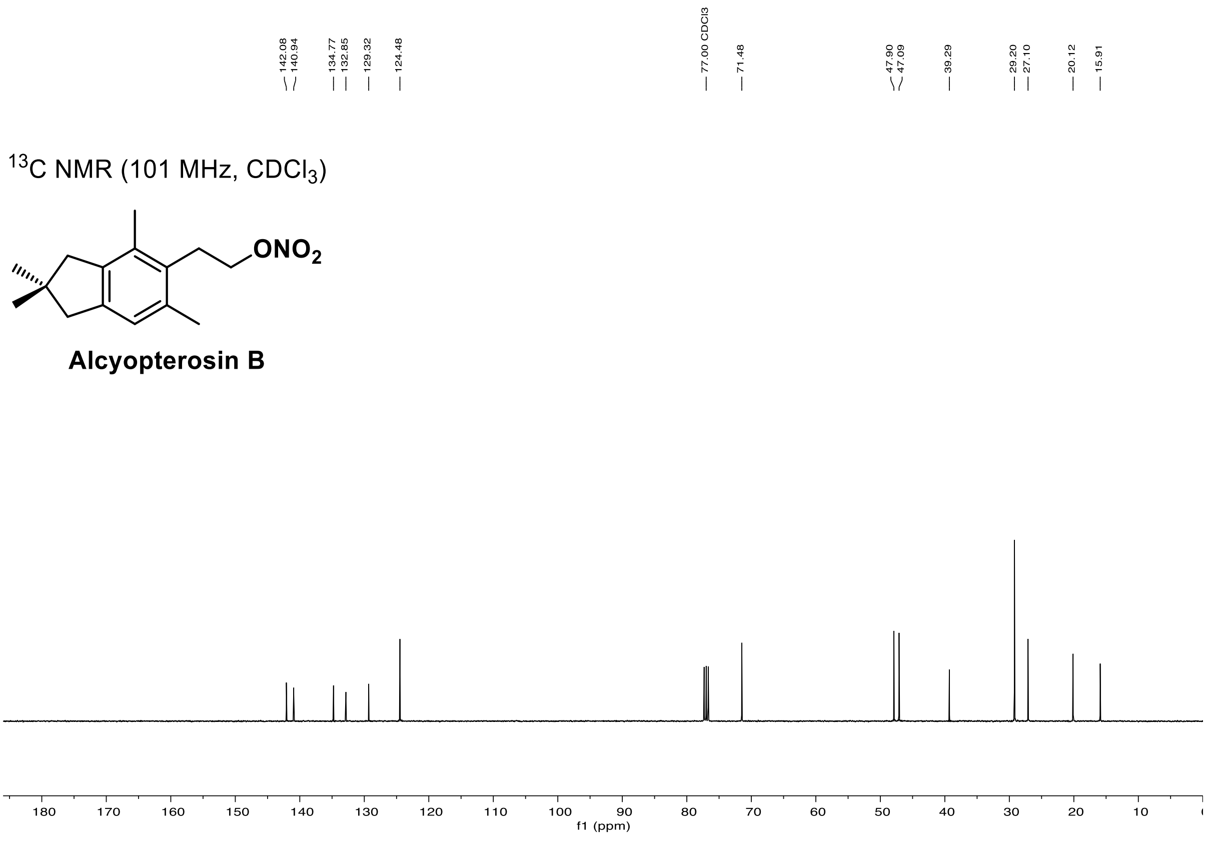

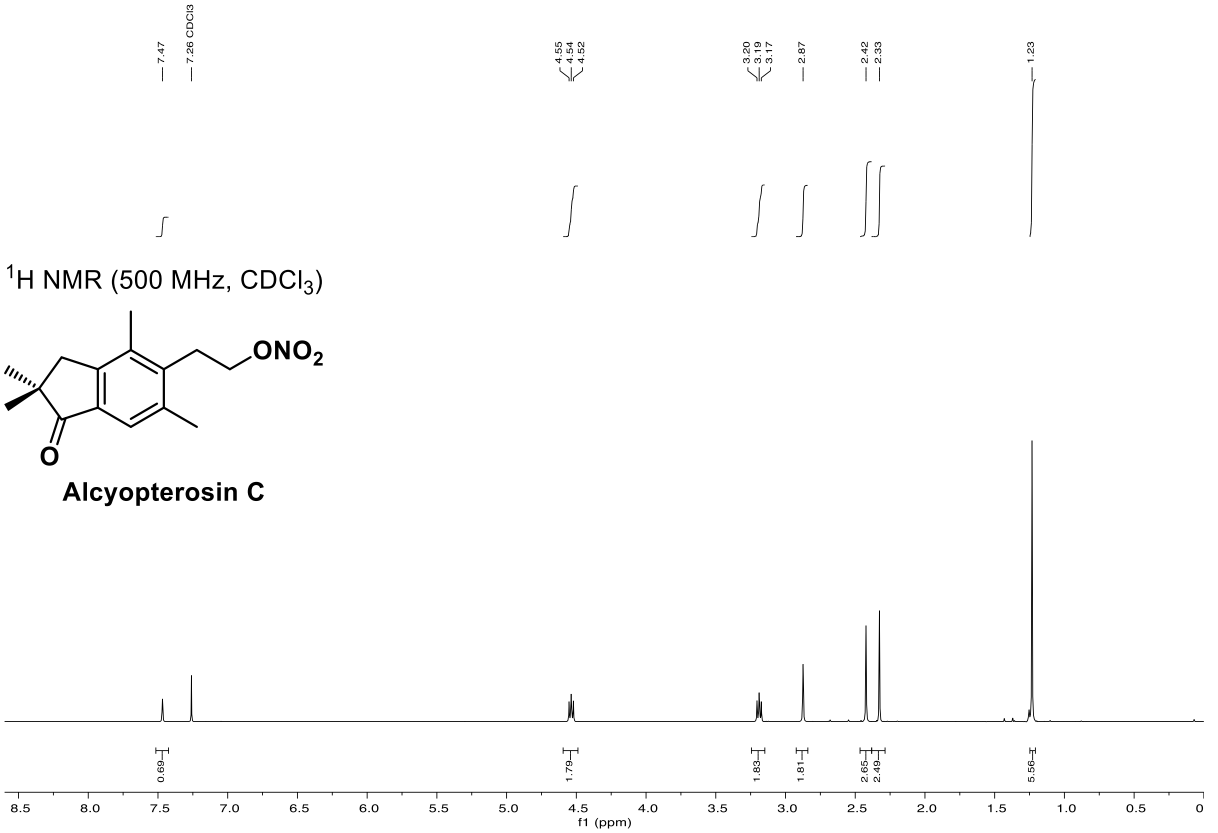

7.47
7.26 CDCl3
4.55
4.54
4.52
3.20
3.19
3.17
2.87
2.42
2.33
1.23
1H NMR (500 MHz, CDCl3)
ONO2
O
Alcyopterosin C
0.69
1.79
1.83
1.81
2.65
2.49
5.56
8.5 8.0 7.5 7.0 6.5 6.0 5.5 5.0 4.5 4.0 3.5 3.0 2.5 2.0 1.5 1.0 0.5 0
f1 (ppm)

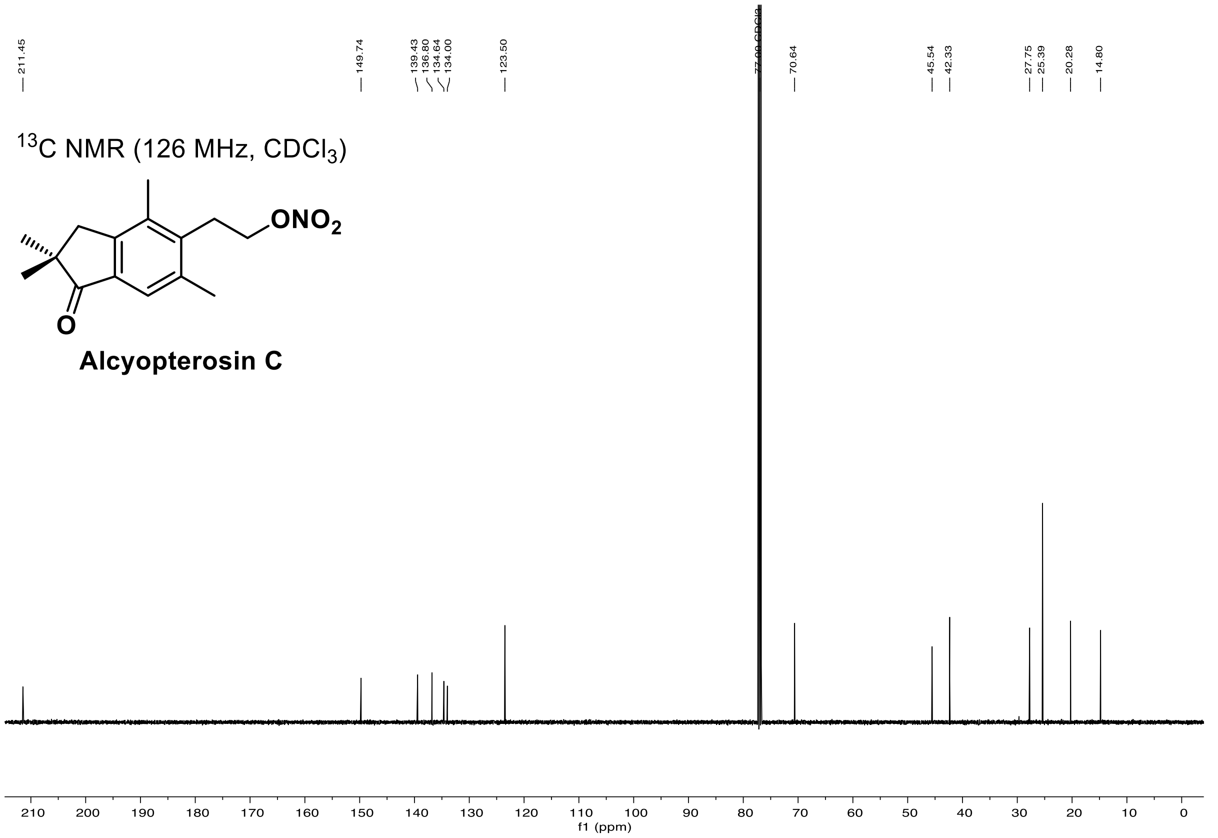

211.45
149.74
139.43
136.80
134.64
134.00
123.50
70.64
45.54
42.33
27.75
25.39
20.28
14.80
13C NMR (126 MHz, CDCl3)
ONO2
O
Alcyopterosin C
210 200 190 180 170 160 150 140 130 120 110 100 90 80 70 60 50 40 30 20 10 0
f1 (ppm)

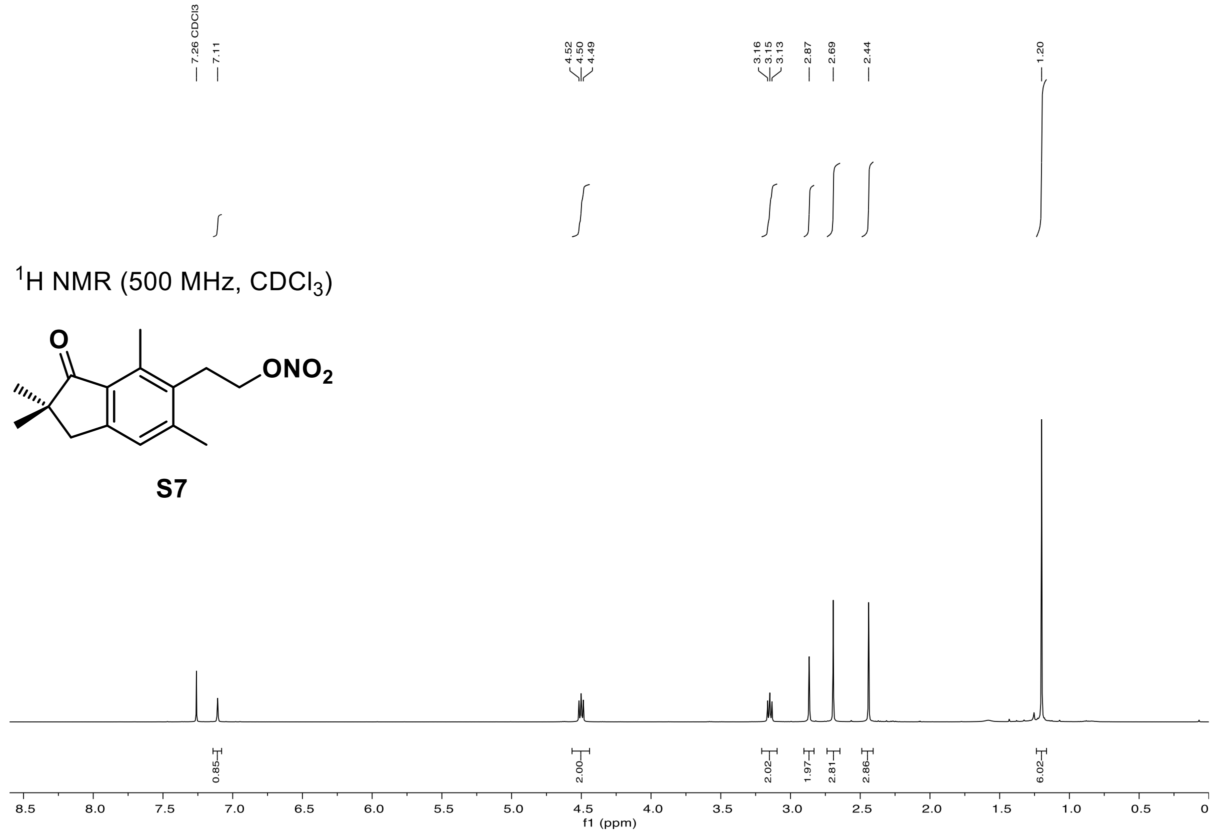
7.26 CDCl3
7.11
4.52
4.50
4.49
3.16
3.15
3.13
2.87
2.69
2.44
1.20
1H NMR (500 MHz, CDCl3)
ONO2
S7
0.85
2.00
2.02
1.97
2.81
2.86
6.02
8.5 8.0 7.5 7.0 6.5 6.0 5.5 5.0 4.5 4.0 3.5 3.0 2.5 2.0 1.5 1.0 0.5 0
f1 (ppm)

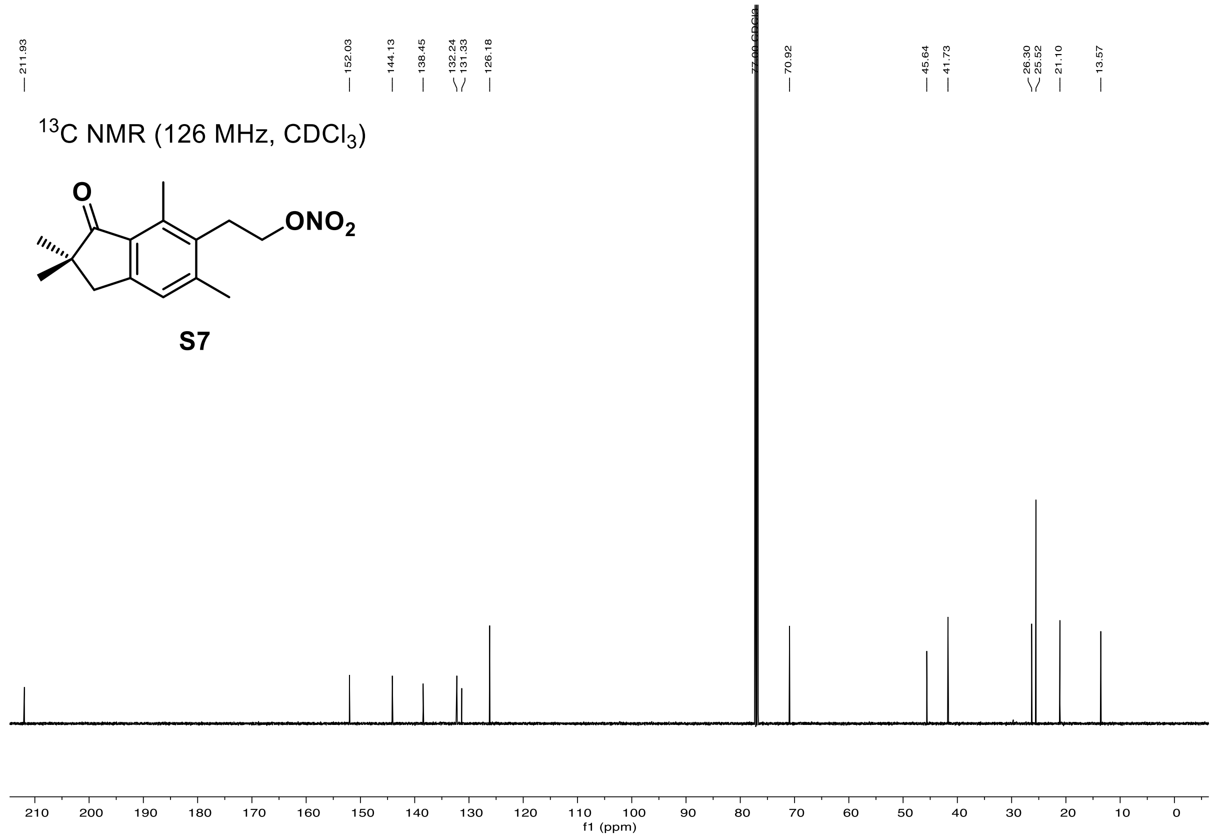
211.93
152.03
144.13
138.45
132.24
131.33
126.18
70.92
45.64
41.73
26.30
25.52
21.10
13.57
13C NMR (126 MHz, CDCl3)
ONO2
S7
210 200 190 180 170 160 150 140 130 120 110 100 90 80 70 60 50 40 30 20 10 0
f1 (ppm)

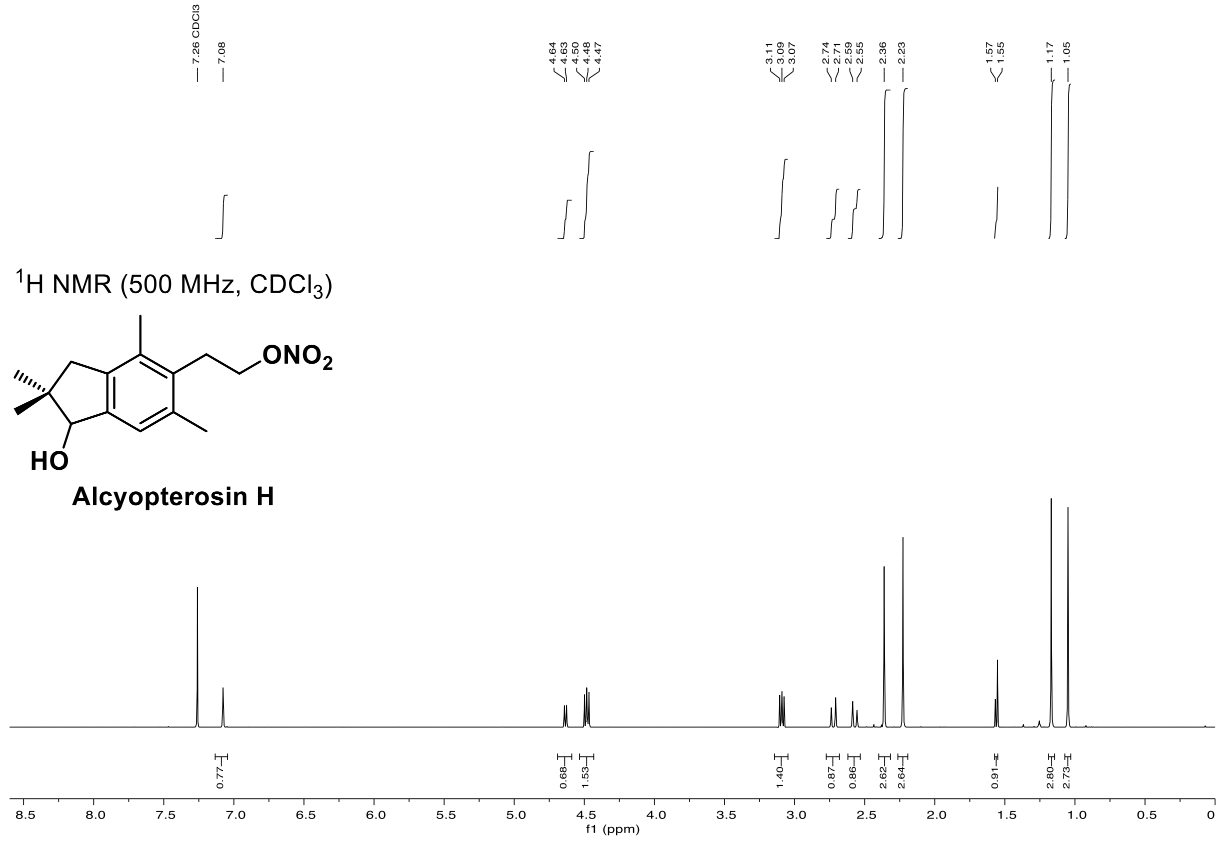

1H NMR (500 MHz, CDCl3)
ONO2
HO
Alcyopterosin H
7.26 CDCl3
7.08
4.64
4.63
4.50
4.48
4.47
3.11
3.09
3.07
2.74
2.71
2.59
2.55
2.36
2.23
1.57
1.55
1.17
1.05
0.77
0.68
1.53
1.40
0.87
0.86
2.62
2.64
0.91
2.80
2.73
f1 (ppm)

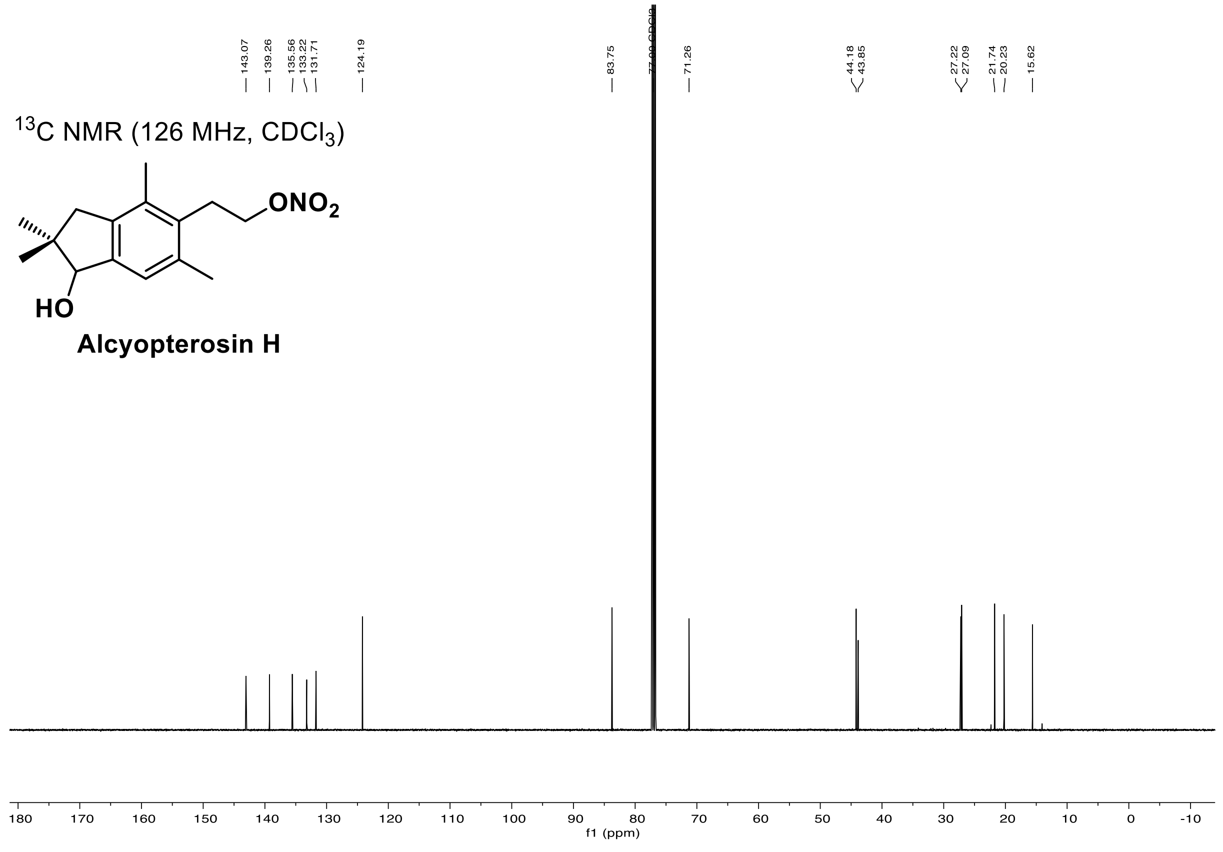

13C NMR (126 MHz, CDCl3)
ONO2
HO
Alcyopterosin H
143.07
139.26
135.56
133.22
131.71
124.19
83.75
71.26
44.18
43.85
27.22
27.09
21.74
20.23
15.62
f1 (ppm)

^{1}H NMR (500 MHz, $CDCl_3$)

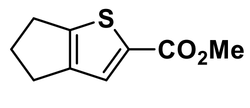

S8

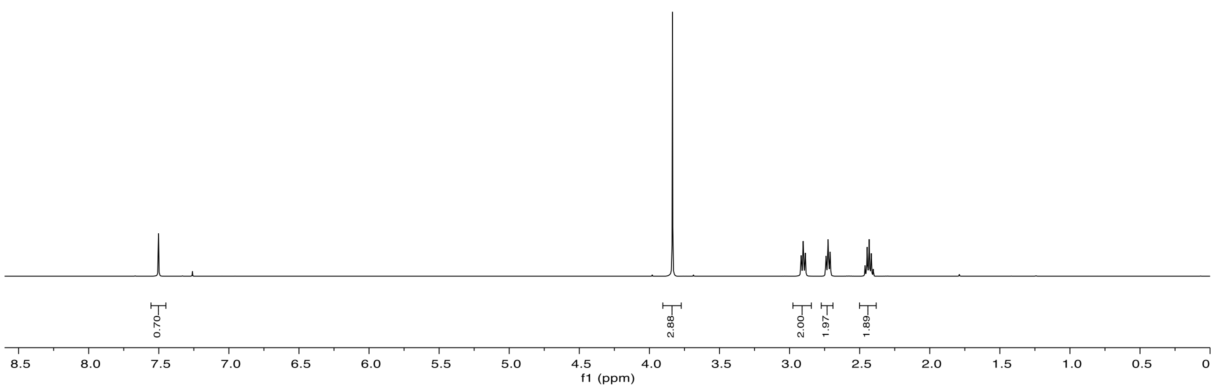

^{13}C NMR (126 MHz, $CDCl_3$)

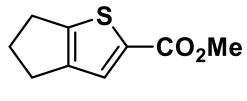

S8

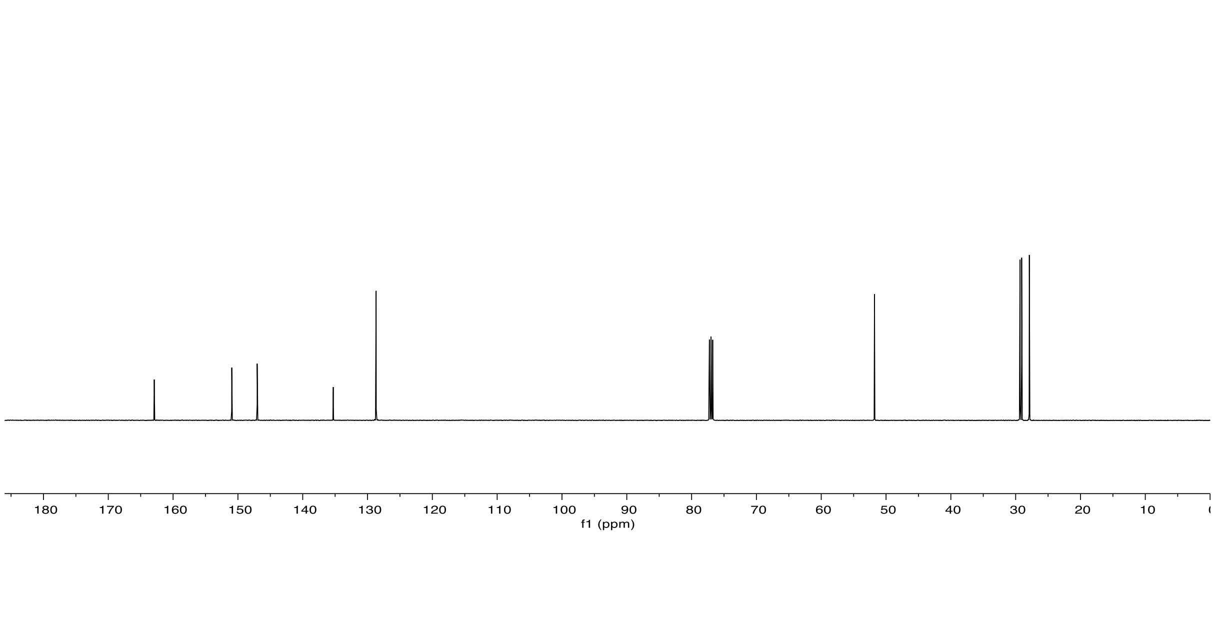

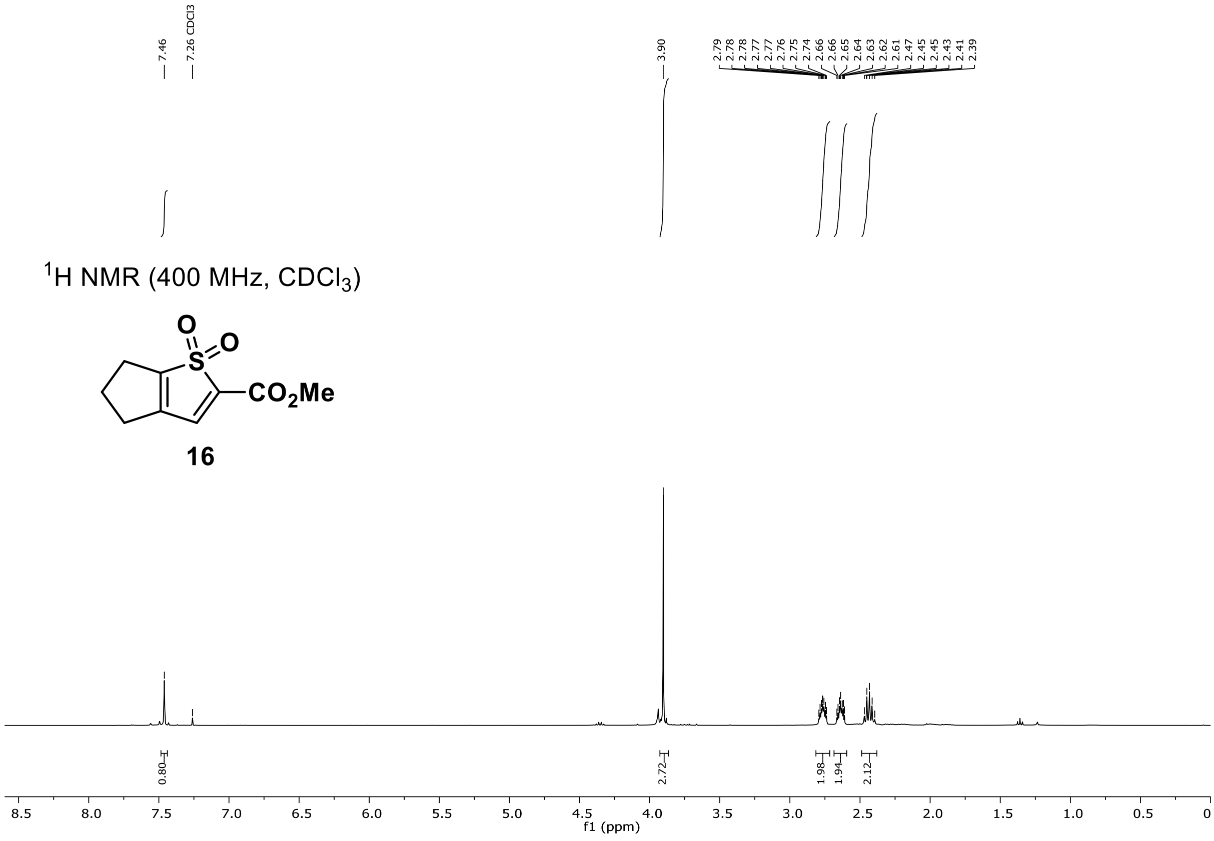
7.46
7.26 CDCl3
3.90
2.79 2.78 2.78 2.77 2.77 2.76 2.75 2.74 2.66 2.66 2.65 2.64 2.63 2.62 2.61 2.47 2.45 2.45 2.43 2.41 2.39
^{1}H NMR (400 MHz, $CDCl_3$)
CO_2Me
16
0.80
2.72
1.98
1.94
2.12
f1 (ppm)

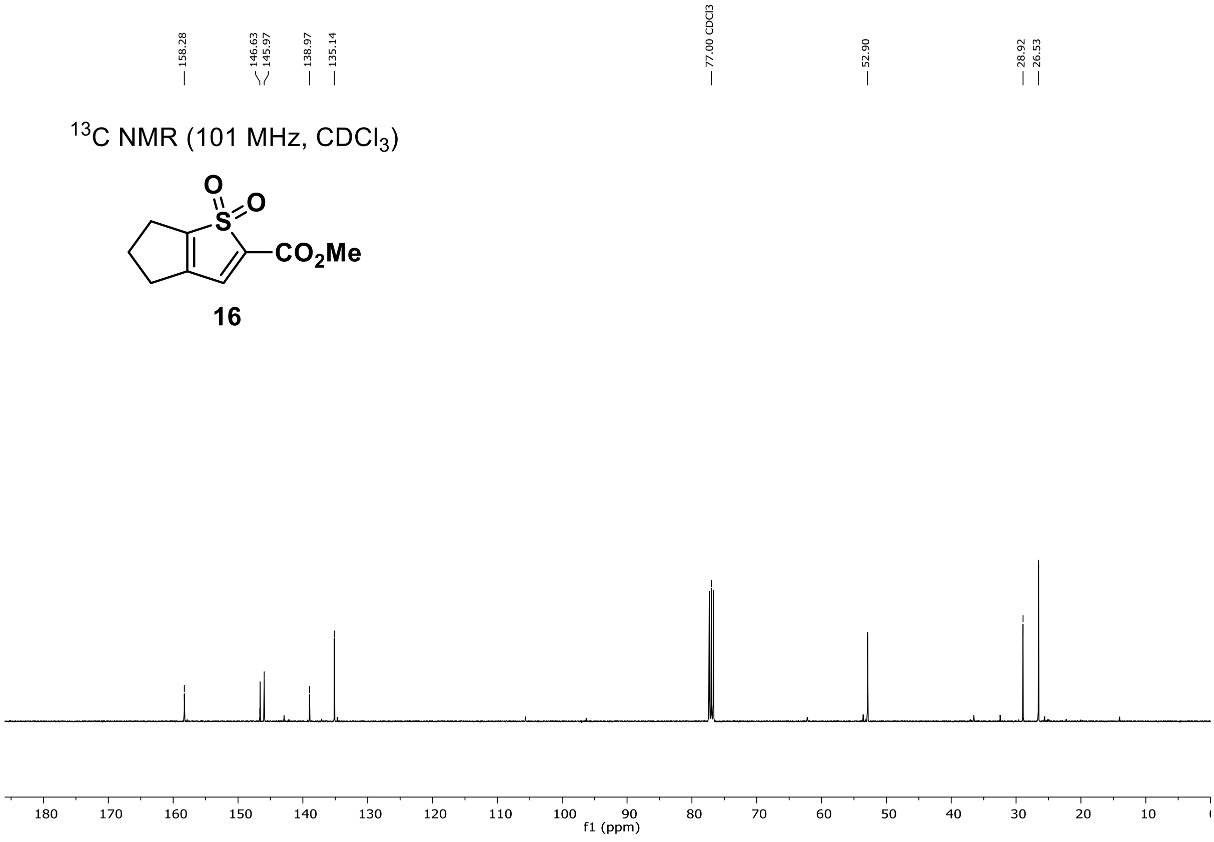
158.28
146.63
145.97
138.97
135.14
77.00 CDCl3
52.90
28.92
26.53
^{13}C NMR (101 MHz, $CDCl_3$)
CO_2Me
16
f1 (ppm)

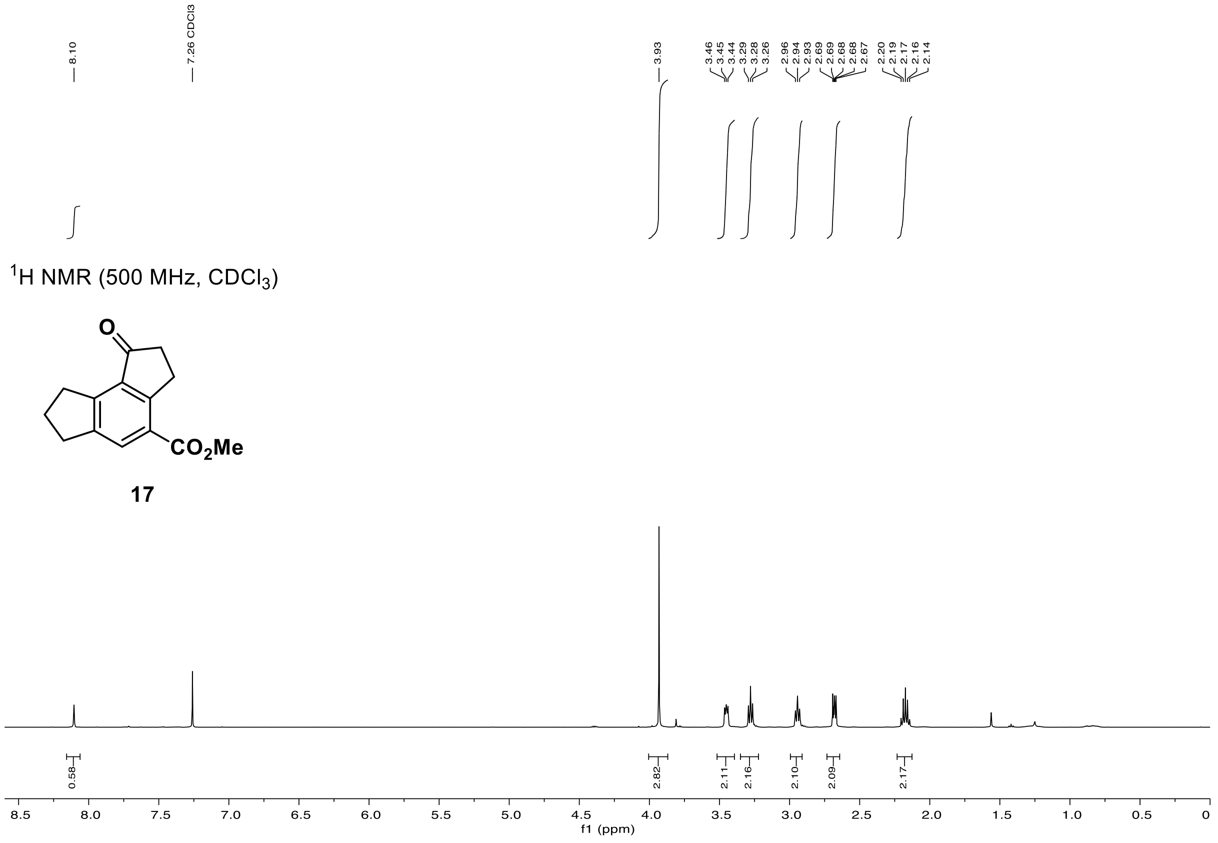
^{1}H NMR (500 MHz, $CDCl_3$)
CO2Me
17
8.10
7.26 CDCl3
3.93
3.46
3.45
3.44
3.29
3.28
3.26
2.96
2.94
2.93
2.69
2.68
2.68
2.67
2.20
2.19
2.17
2.16
2.14
0.58
2.82
2.11
2.16
2.10
2.09
2.17
8.5 8.0 7.5 7.0 6.5 6.0 5.5 5.0 4.5 4.0 3.5 3.0 2.5 2.0 1.5 1.0 0.5 0
f1 (ppm)

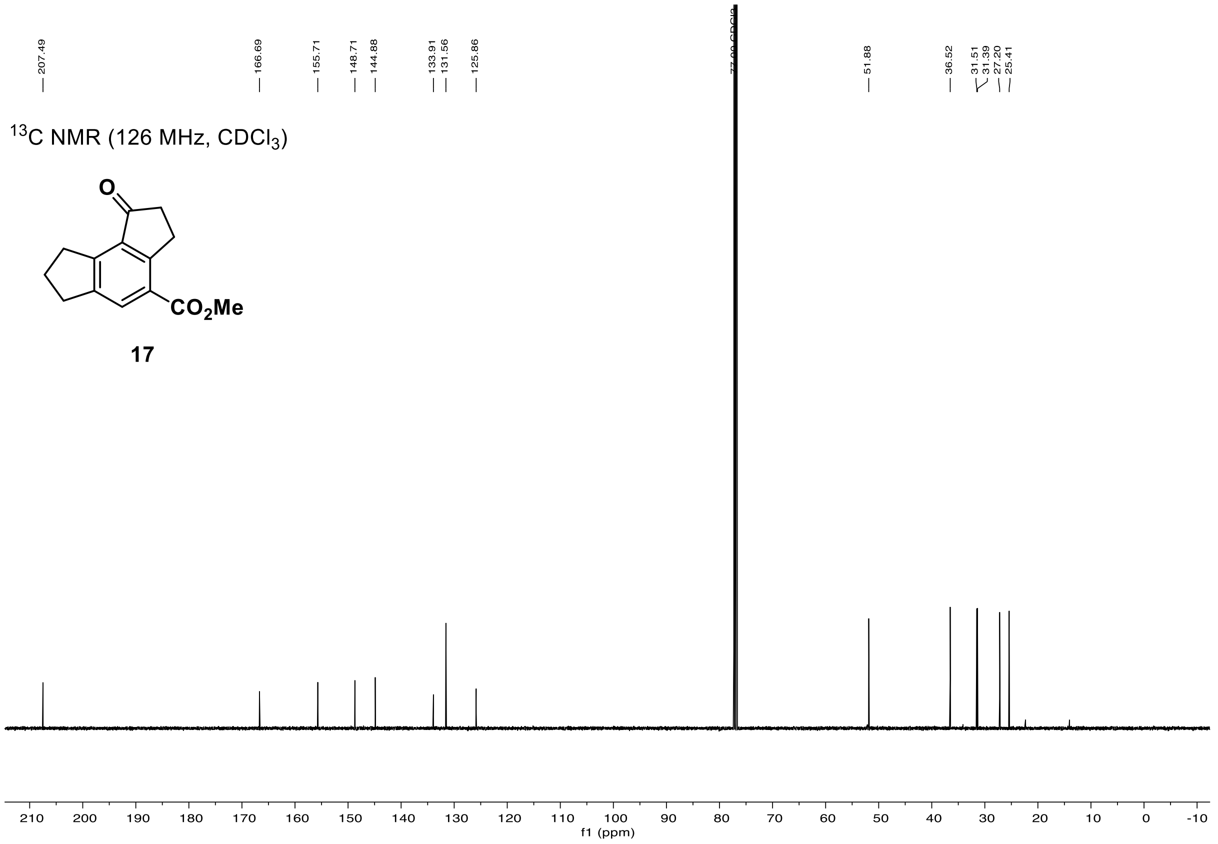
^{13}C NMR (126 MHz, $CDCl_3$)
CO2Me
17
207.49
166.69
155.71
148.71
144.88
133.91
131.56
125.86
51.88
36.52
31.51
31.39
27.20
25.41
210 200 190 180 170 160 150 140 130 120 110 100 90 80 70 60 50 40 30 20 10 0 -10
f1 (ppm)

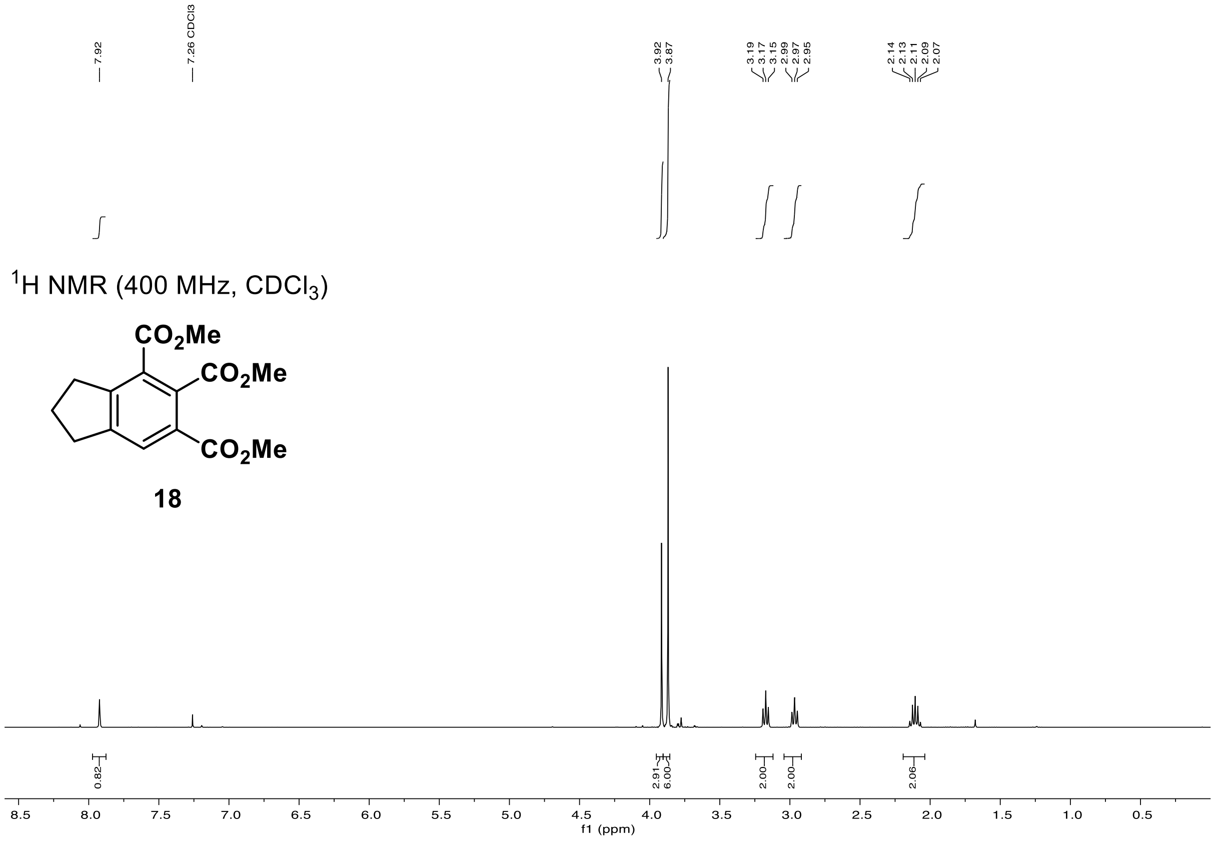
7.92
7.26 CDCl3
3.92
3.87
3.19
3.17
3.15
2.99
2.97
2.95
2.14
2.13
2.11
2.09
2.07
^{1}H NMR (400 MHz, CDCl$_3$)
CO_2Me
CO_2Me
CO_2Me
18
0.82
2.91
6.00
2.00
2.00
2.06
8.5
8.0
7.5
7.0
6.5
6.0
5.5
5.0
4.5
4.0
3.5
3.0
2.5
2.0
1.5
1.0
0.5
f1 (ppm)

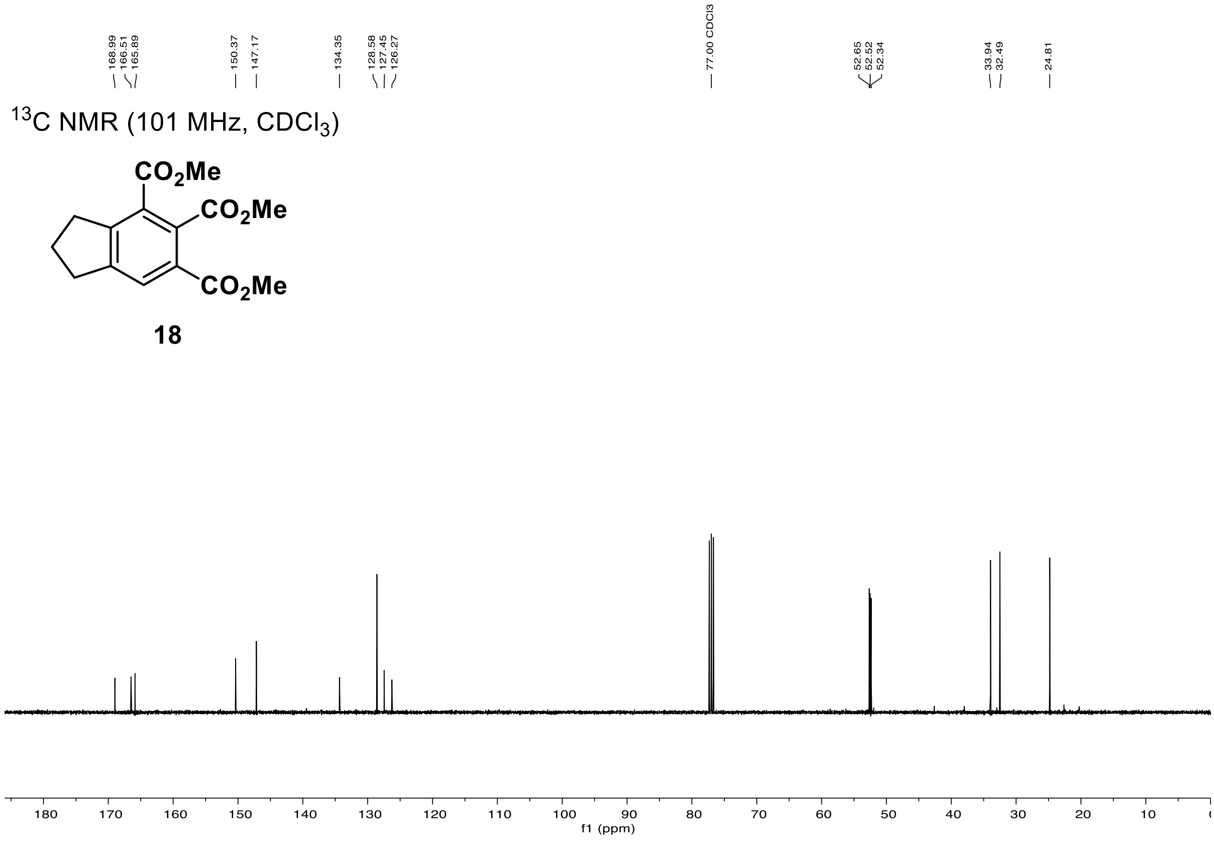
168.99
166.51
165.89
150.37
147.17
134.35
128.58
127.45
126.27
77.00 CDCl3
52.65
52.52
52.34
33.94
32.49
24.81
^{13}C NMR (101 MHz, CDCl$_3$)
CO_2Me
CO_2Me
CO_2Me
18
180
170
160
150
140
130
120
110
100
90
80
70
60
50
40
30
20
10
f1 (ppm)

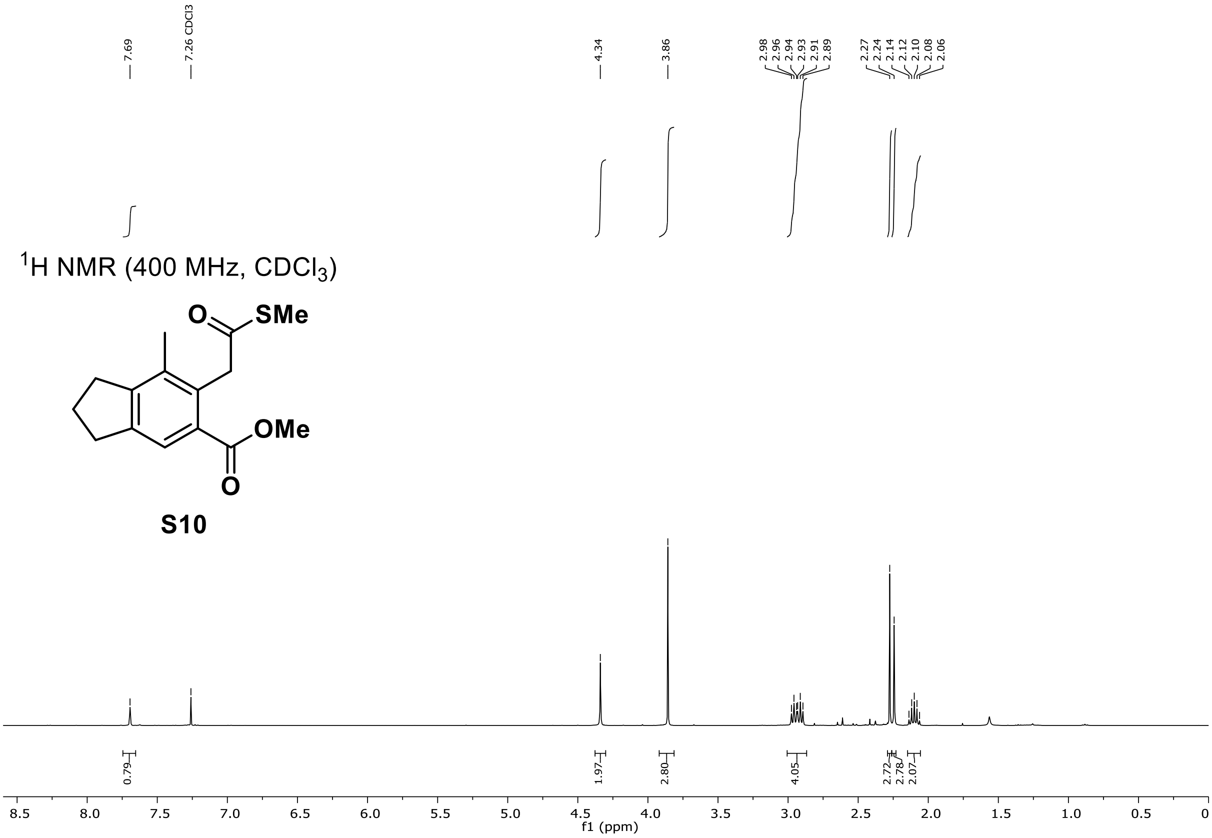

^{1}H NMR (400 MHz, $CDCl_3$)

S10

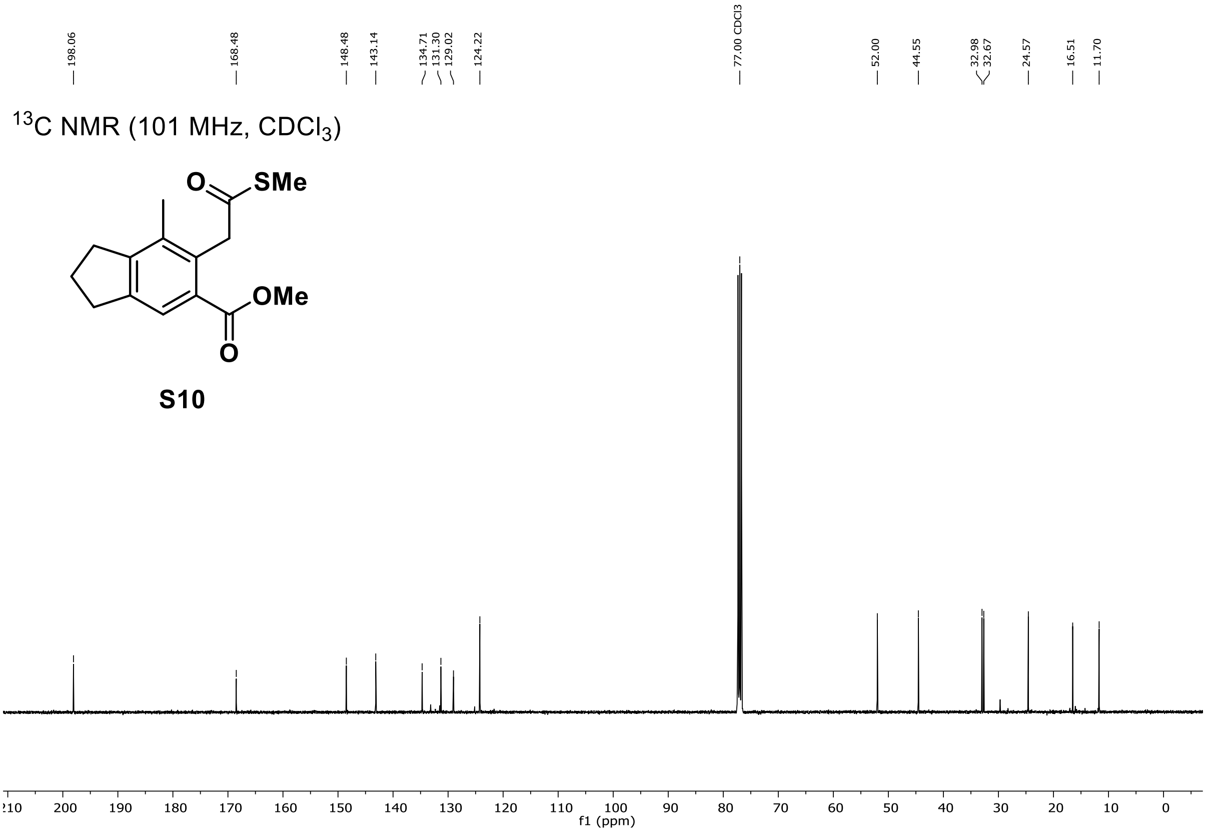

^{13}C NMR (101 MHz, $CDCl_3$)

S10

5. Computational details

All calculations were carried out using the ORCA suite of programs (version 4.2.1).[13] Optimizations and single-point energy calculations were carried out using "Tight" convergence criteria, corresponding to tolerances of 10^{-8} Hartrees for the SCF energy change, and 10^{-6} Hartrees for the optimization step. To speed up the calculations, the resolution-of-identity (RI) chain-of-spheres exchange (RIJCOSX) approximation was employed.[14] The integration grid "Grid5" was employed for energies, corresponding to a Lebedev-434 angular grid, and a radial integral accuracy (IntAcc) of 5.01. For calculations employing the RI approximation a "GridX6" was employed, corresponding to IntAcc = 4.34 and a Lebedev-194 angular grid. For calculations of triple-zeta basis set quality, correlation integrals used the def2-TZVPP/C auxiliary basis set.[15]

Geometries were initially obtained *via* autodE using standard settings, with GFN2-xTB for conformational sampling and PBE-D3BJ/def2-SVP for geometry optimization.[16] Dispersion corrections were included using Grimme's D3 empirical method with Becke-Johnson damping (D3BJ).[17] The obtained geometries were then optimized at the CPCM(Toluene)-M06-2X/def2-TZVPP level of theory.

The Domain-based Local Pair Natural Orbital coupled cluster method with singles, doubles and perturbative triples (DLPNO-CCSD(T)) was used as a final single point providing reliable electronic energies (*vide infra*) using the def2-TZVPP basis set.[18] DLPNO-CCSD(T) calculations were run using "NormalPNO" cut-offs, corresponding to TCutPairs = 10^{-5} Hartrees, TCutPNO = 3.33 10^{-7} and TCutMKN = 10^{-3}, where TCutPairs is the correlation threshold for inclusion of a given electron pair in the CCSD calculation, TCutPNO is the occupation threshold for each PNO (pair natural orbital) formed from an expansion of projected atomic orbitals (PAOs), and TCutMKN determines the threshold for the inclusion of each localized MO in the PNO expansion based on Mulliken population.[19]

Vibrational frequencies were computed at the optimization level of theory to confirm whether the structures correspond to minima or transition states. All intermediate structures were verified to be minima by the absence of imaginary frequencies upon calculation of the Hessian, unless stated otherwise.[1] Grimme's quasiRRHO approach was used to calculate free energies at 373.15 K.[20] A standard state correction from 1 atm to 1 M was applied by adding RTln(1/24.5) = 2.37 kcal mol^{-1} (T = 373.15 K) to the calculated free energy of each species. For calculating thermodynamic data the python-script *OTherm.py* was used with ω_0 = 100 cm^{-1} replacing harmonic oscillators with free-rotors below ω_0.[20-21]

The 2D surface (More O'Ferrall-Jencks plot) was generated in the space defined by the forming C1–C4 and C2–C5 bonds distances using a grid of 0.1 Å. The area around stationary points **TS3** and **TS4** were sampled with a smaller grid size of 0.05 Å (Figure S4). The used script for visualization of the PES using a cubic spline (*scipy.interp2d*) can be found in the accompanied .zip repository.

[1] Residual imaginary frequencies were inverted

Evaluation of level of theory

The selection of the density functional chosen for this study was based on the available literature, which was evidenced the reliability of M06-2X to model Diels–Alder reactions. For example, Brinck *et al.*[22] evaluated the dependence of transition state geometries on the choice of the optimization method, observing significant discrepancies between hybrid and pure density functionals. Their study identified ωB97X(D), B2PLYP(D) and M06-2X as the best functionals when compared to CCSD/6-31+G(d) geometries. Moreover, Houk *et al.* have also employed the M06-2X functional to investigate ambimodal reactivity,[23] demonstrating that while similar geometries are obtained between B3LYP(D3) and M062X, the former underestimate activation energies (see SI [24]).

To confirm that M06-2X correctly describes the geometry of the ambimodal transition state **TS3**, which is expected to have a larger dependence on the method,[22] we also compared our results to those obtained with PBE0 and B3LYP functionals. Considering the size of our system, we used SCS-MP2 as the reference method. (CPCM(Toluene)-SCS-MP2/def2-TZVPP).[25] Our results indicate that M06-2X provides the closest geometry to the one obtained with SCS-MP2, while the other two DFT functional have larger deviations, especially for the C3-C6 bond which would affect the product selectivity (Figure S1). Moreover, **IM1**, **IM2** and **TS1** geometries also show a good agreement with those obtained using SCS-MP2 (Table S1). The largest deviation of 0.13 Å was found for the secondary interaction C3-C6 in **IM1**.

M06-2X also provide similar trends when compared to DLPNO-CCSD(T) energies. However, significant differences are observed for **IM1** and **IM2**, even when larger basis sets are used (Table S2). These results motivated us to use DLPNO-CCSD(T)/def2-TZVPP single point energy corrections in all our results. Our method of choice, DLPNO-CCSD(T)/def2-TZVPP//M06-2X/def2-TZVPP therefore provides a good compromise between efficient geometry optimizations and accurate energies.

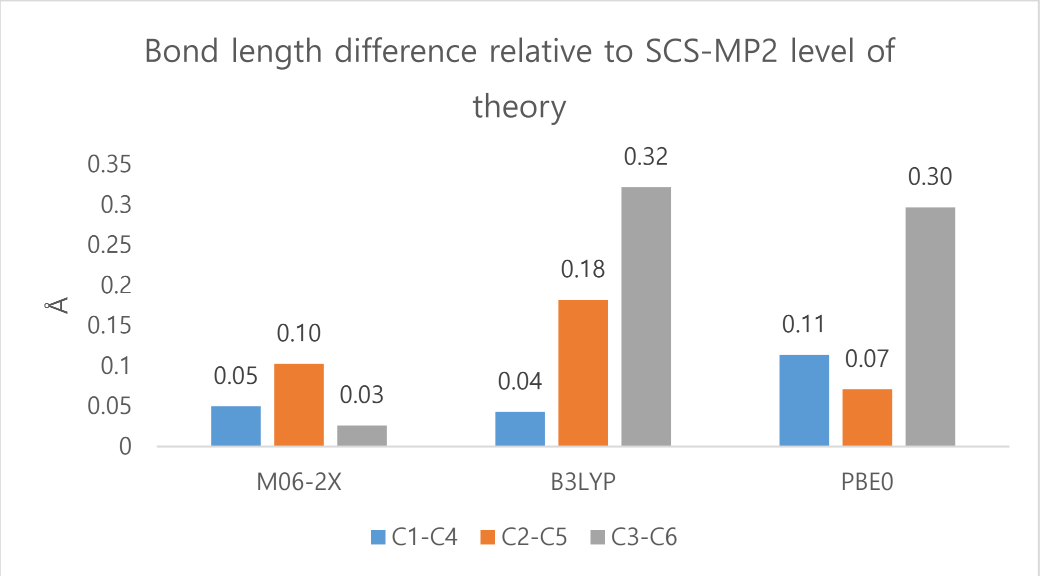

Figure S1. Bond length difference of **TS3** of tested functional at CPCM(Toluene)-[Functional]/def2-TZVPP level relative to CPCM(Toluene)-SCS-MP2/def2-TZVPP level of theory.

Table S1 Bond length difference of **IM1**, **IM2** and **TS1** of tested functional at CPCM(Toluene)-M06-2X/def2-TZVPP level relative to CPCM(Toluene)-SCS-MP2/def2-TZVPP level of theory.

Relative bond length in Å	IM1	IM2	TS1
**C1-C4**	0.01	0.00	−0.06
**C2-C5**	0.00	0.02	−0.03
**C3-C6**	−0.13	0.01	0.03

Table S2 Comparison of electronic energies relative to **5**+**16** in kcal mol^{-1} (see Table S4 for full data) at given level of theory. All calculations employ a CPCM(Toluene) solvent system.

Energies relative to **5**+**16** in kcal mol^{-1}	M06-2X/def2-TZVPP// M06-2X/def2-TZVPP	M06-2X/def2-QZVPP// M06-2X/def2-TZVPP	DLPNO-CCSD(T)/def2-TZVPP//M06-2X/def2-TZVPP	SCS-MP2/def2-TZVPP//SCS-MP2/def2-TZVPP
**TS1**	13.2	13.5	10.5	
**TS2**	24.5	24.9	26.0	
**TS3**	11.1	11.5	8.5	6.9
**TS4**	17.7	17.9	14.3	
**IM1**	−22.5	−22.5	−30.8	−28.7
**IM2**	−15.6	−14.3	−23.0	−20.0

Distortion-interaction and NBO analysis

Decomposition of the activation barrier for the reactions between **16** and different dienophiles (cyclopentenone, cyclopentene, DMAD, Butyene, Furan **5**) was performed employing the distortion-interaction analysis, which partitions the electronic energy in terms of distortion and interaction terms.[26]

This analysis reveals that all the systems undergoing a pericyclic pathway follow a similar trend, with the higher activation energies for DMAD and butyene arising from an increase in total distortion. For the ambimodal pathway, a favorable interaction energy (–21.2 kcal mol^{-1}) compensates the slight increase in distortion (29.7 kcal mol^{-1}), which results in an overall low activation energy (8.5 kcal mol^{-1}, Figure S2).

The origin of this stabilization can be further analyzed via second-order perturbation theory analysis of the Fock matrix using Natural Bond Orbital (NBO) analysis (Figure 4 main text and Table S3).[27]

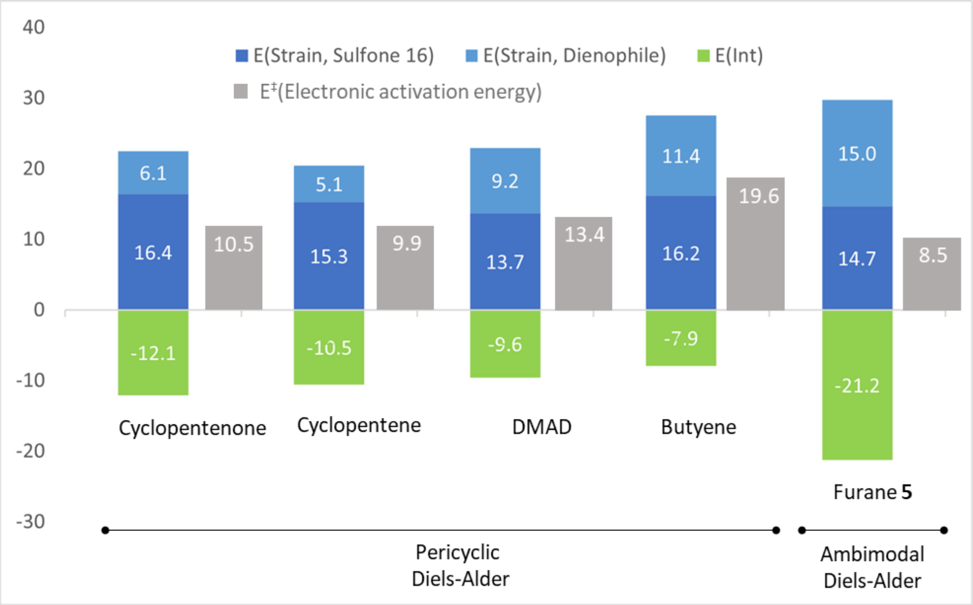

Figure S2. Energy components corresponding to distortion (blue), interaction (green) and total energy (kcal mol^{-1}) reactions undergoing pericyclic TSs (left) and ambimodal pathway (right). Calculations were carried out at the CPCM(Toluene)-DLPNO-CCSD(T)/def2-TZVPP//CPCM(Toluene)-M06-2X/def2-TZVPP level of theory. Full data provided in Table S5.

Table S3. Relevant second-order perturbation theory values of NBO analysis visualized in Figure 4 (main text). Calculations were carried out at the CPCM(Toluene)-M06-2X/def2-TZVPP level of theory. All values are given in kcal mol^{-1}.

Donor (L) NBO	Acceptor (NL) NBO	E(2)	E(NL)-E(L)	F(L,NL)
32. LP (2) O 8	97. BD*(1) C 1 – C 4	1.97	0.43	0.026
35. LP (2) O 16	97. BD*(1) C 1 – C 4	16.81	0.50	0.082
36. LP (1) C 15	97. BD*(1) C 1 – C 4	86.81	0.20	0.116
46. BD (1) O 8 – S 7	97. BD*(1) C 1 – C 4	1.10	1.02	0.0300
51. BD (1) C 1 – C 4	92. BD*(1) O 8 – S 2	3.06	0.79	0.044
51. BD (1) C 1 – C 4	95. BD*(1) S 7 – O 9	1.76	0.79	0.033
51. BD (1) C 1 – C 4	100. BD*(1) C 12 – H 13	2.58	0.84	0.042
51. BD (1) C 1 – C 4	101. BD*(1) C 12 – H 14	1.69	0.85	0.034
51. BD (1) C 1 – C 4	105. BD*(1) C 1 – C 12	1.10	0.84	0.027

51. BD (1) C 1 – C 4	112. BD*(2) C 5 – C 6	18.24	0.45	0.081
51. BD (1) C 1 – C 4	132. BD*(1) C 10 – H 11	3.67	0.84	0.050
54. BD (1) C 12 – H 13	97. BD*(1) C 1 – C 4	6.70	0.60	0.057
55. BD (1) C 12 – H 14	97. BD*(1) C 1 – C 4	1.13	0.61	0.023
59. BD (1) C 17 – C 15	97. BD*(1) C 1 – C 4	1.05	0.68	0.024
66. BD (2) C 5 – C 6	97. BD*(1) C 1 – C 4	17.53	0.38	0.073

Ambimodal selectivity

a) Goodman's *ValleyRidge.py* script

Goodman's ValleyRidge script[28] was used to determine the selectivity of the bifurcating PES. The Hesse-matrices of **TS4** and **TS3** as well as the coordinates of **IM1** and **IM2** were used as input files. Following output sequences was generated by *ValleyRidge.py* (the full file is available in the accompanied .xyz folder):

```
[…]

**** Analysis Completed ****
Major product is P2molfile.mol
Minor product is P1molfile.mol

mu1_ = -0.9872606032385755
mu2_ = 3.1239264437018717
lambda1_ = 118.42867384099547
lambda2_ = 215.06043224550746
|g_| = 1.76375948955816
WARNING: |g_| is large (|g_| > 1)
phi = 57.647271878305304

The algorith will now proceed to estimate the major and minor product
ratios

Product Ratio Calculation Completed:
Major Product : Minor Product ratio
99.9 : 0.1

***************************
```

Note: Major product resembles ***IM2****, Minor product resembles* ***IM1***

Note: A large |g_| limits the quantitatively predictability although the obtained result hints towards an extreme ratio ***IM1****:****IM2****.*

b) Houk's LFER between product ratio and forming bond length difference

Using published work of 15 ambimodal pericyclic reactions, Houk *et al.* derived a LFER between the bond length difference $\Delta d = \Delta(r_{C3\text{-}C6} - r_{C2\text{-}C5}) = -0.26$ Å and the product ratio **IM1**:**IM2 = IMC$_2$-C$_5$:IMC$_3$-C$_6$**. A $R^2 = 0.92$ shows good correlation between the asynchronicity of the ambimodal transition state arrangement but also indicates interplay of other effects, e.g. momenta distribution, determining dynamic selectivity.[29]

$$\ln\left(\frac{IMC_3-C_6}{IMC_2-C_5}\right) = -9.4(\Delta d) = 2.444 \quad \Rightarrow \frac{IMC_3-C_6}{IMC_2-C_5} = 11.5$$

Absolute energies and charge analysis

Table S4. Thermodynamic quantities of the investigated reaction. Thermodynamic calculations were carried out at the CPCM(Toluene)-M06-2X/def2-TZVPP level of theory. Free energy G was calculated using the electronic energy of CPCM(Toluene)-DLPNO-CCSD(T)/def2-TZVPP//CPCM(Toluene)-M06-2X/def2-TZVPP level of theory *via* $G = E_{el}(\text{DLPNO-CCSD(T)}) + [G(\text{M06-2X}) - E_{el}(\text{M06-2X})]$

		Electronic energy [Ha] M06-2X	Electronic energy DLPNO-CCSD(T) [Ha]	Enthalpy H M06-2X [Ha]	Free energy G [Ha]	ΔG rel.**16**+**5** [kcal mol^{-1}]
**Substrate**	**16**	−1048.0059	−1046.5345	−1047.8004	−1046.39799	-
	Furan **5**	−706.82918	−705.85626	−706.68773	−705.769315	-
	DMAD	−533.09395	−532.27562	−532.96052	−532.203118	-
	Cyclopentenone	−269.34156	−268.89607	−269.23419	−268.831175	-
**Amibmodal system**	**5**+**16**					0.0
	**TS3**	−1754.8175	−1752.3773	−1754.4698	−1752.12317	27.7
	**IM1**	−1754.8710	−1752.4399	−1754.5206	−1752.18225	−9.4
	**IM2**	−1754.8583	−1752.4275	−1754.5085	−1752.16844	−0.7
	**TS4**	−1754.8068	−1752.3681	−1754.4595	−1752.11236	34.5
	**TS2**	−1754.7961	−1752.3494	−1754.4491	−1752.09721	44.0
**-SO_2**	**IM1**		*Relative to IM1*			0.0
	TS(SO_2 Extrusion)	-1754.8458	-1752.4094	-1754.4979	-1752.1564	16.2
	SO_2 (aq)	-548.6292	-547.9845	-548.61661	-548.0044	−15.1
	Extruded product	-1206.2430	-1204.4516	-1205.9082	-1204.2019	
	TS(DMAD)	−1581.0737	−1315.4139	−1580.7345	−1578.551847	30.9
	TS(Cyclopentenone)	−1317.3265	−1578.7889	−1317.0127	−1315.183812	28.5

Table S5. Energetic data of distortion-interaction analysis**.** Calculations were carried out at the CPCM(Toluene)-DLPNO-CCSD(T)/def2-TZVPP//CPCM(Toluene)-M06-2X/def2-TZVPP level of theory.

	Cyclopentenone E[Ha]	Cyclopentene E[Ha]	DMAD E[Ha]	Butyene E[Ha]	Furan E[Ha]
**16, relaxed**	−1046.534533	−1046.534533	−1046.534533	−1046.534533	−1046.534533
**Dienophile, relaxed**	−268.8960676	−194.961286	−532.2756212	−155.6854358	−705.8562611
**16, unrelaxed**	−1046.508378	−1046.510113	−1046.512632	−1046.508719	−1046.511083
**Dienophile, unrelaxed**	−268.8863382	−194.9531819	−532.2609758	−155.6673478	−705.832321
**TS**	−1315.413932	−1241.480094	−1578.788857	−1202.188658	−1752.37726
			**Values in kcal mol^{-1}**		
**E(Strain, 16)**	16.4	15.3	13.7	16.2	14.7
**E(Strain, Dienophile)**	6.1	5.1	9.2	11.4	15.0
**E(Strain)**	22.5	20.4	22.9	27.5	29.7
**E(Int)**	−12.1	−10.5	−9.6	−7.9	−21.2

Table S6. Hirshfeld atomic charges (e^- units) for the different transition states computed at the CPCM(Toluene)-M06-2X/def2-TZVPP level of theory.

**16**		**Cyclopentenone**		**16**		**DMAD**		**16**		**Furan 5**	
**index**	**charge**	**index**	**H_{charge}**	**index**	**charge**	**index**	**H_{charge}**	**index**	**charge**	**index**	**H_{charge}**
**1**	0.95026	7	0.33743	5	-0.00178	1	-0.27271	1	0.97009	7	-0.29928
**2**	2.23454	8	0.10678	6	-0.04103	2	0.21296	2	-2.23276	8	0.22019
**3**	0.16653	9	0.45363	7	-0.03823	3	-0.02198	3	0.2436	9	0.31131
**4**	0.46287	10	0.52560	8	-0.03557	4	-0.02409	4	0.44166	10	-0.17153
**5**	0.38746	11	-0.57384	9	0.02124	19	0.22464	5	0.38957	11	0.46692
**6**	0.43385	12	0.58412	10	0.00303	20	-0.10894	6	0.43819	30	-0.26157
**13**	0.05351	27	-0.26815	11	-0.01667	21	0.01340	12	0.10091	31	-0.33423
**14**	0.16044	28	-0.25061	12	0.20358	22	-0.25857	13	0.0825	32	0.6589
**15**	0.39029	29	-0.23393	13	-0.10701	23	-0.11091	14	0.51418	33	-0.26608
**16**	-0.76989	30	-0.23388	14	0.01506	24	0.01067	15	-0.76811	34	-0.25906
**17**	0.48758	31	-0.24297	15	-0.27343	35	0.04930	16	0.49052	35	-0.23474
**18**	0.2642	32	-0.25243	16	0.44871	36	0.04859	17	0.26374	36	-0.22614
**19**	0.60946			17	-0.33342	37	0.05873	18	0.63406	37	0.74784
**20**	7.94707			18	-0.31760	38	0.05025	19	0.96992	38	-0.22911
**21**	-0.23982			25	0.04140	39	0.04423	20	-0.23873	39	-0.24332
**22**	-0.24244			26	0.05097	40	0.05576	21	-0.22411	40	-0.24988
**23**	-0.22285			27	0.03232			22	-0.22254		
**24**	-0.22858			28	0.04281			23	-0.20324		
**25**	-0.23328			29	0.05065			24	-0.21999		
**26**	-0.23298			30	0.05093			25	-0.22341		
**33**	-0.26038			31	0.08005			26	-0.24954		
**34**	-0.19483			32	0.04155			27	-0.19281		
**35**	-0.20772			33	0.05079			28	-0.20206		
**36**	-0.19796			34	0.06032			29	-0.19186		
**CT**	-0.04825		0.04825	**CT**	−0.02866		0.02866	**CT**	-0.36978		0.36978

Cartesian coordinates and investigated molecular properties

2-Methyl-5-(methylthio)furan

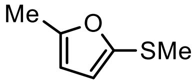

Electronic energy -706.829266057531
Number of imaginary frequencies: 0

C	2.89733421437862	1.76825630158146	0.09573069290974
S	2.59487877213890	0.26819385945847	-0.83955815848985
C	0.96255058767485	-0.20097216032051	-0.52706462560564
C	0.37671066614330	-1.42107776834153	-0.57339664758640
C	-1.03391388483447	-1.17297728072080	-0.46522839257117
C	-1.18736252969483	0.16593570209009	-0.34674969066015
C	-2.32865243562296	1.09082995327671	-0.16579649842725
O	0.03173314829492	0.77078059649034	-0.39761833248419
H	2.17410101828637	2.53686079273275	-0.15382532143877
H	3.88002816726683	2.08411174509201	-0.25354568883825
H	2.93540980099792	1.58280473797511	1.16339008077417
H	0.88416055792025	-2.36312763112009	-0.68794193654223
H	-1.82256231553427	-1.90387123542680	-0.46283487452084
H	-3.24586076110705	0.53246913106930	-0.00923295988126
H	-2.44408954493221	1.73437696999043	-1.03865569571016
H	-2.14942146137617	1.73304228617305	0.69683504907224

Methyl 5,6-dihydro-4H-cyclopenta[b]thiophene-2-carboxylate 1,1-dioxide ***16***

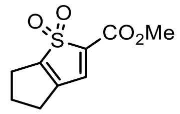

Electronic energy -1048.006218036763
Number of imaginary frequencies: 0

O	0.94800181508175	0.21852602789250	-2.26197057241464
S	0.64671377893598	0.87484527634424	-1.02590778240782
C	-1.04649624802583	0.69834361867235	-0.63419048791186
C	-1.19458970721391	-0.05864399742493	0.44569838414778
C	0.07014207596825	-0.54853961924868	1.03340715825378
C	1.14346034577031	-0.13642142606544	0.36100714387390
C	2.56770217417817	-0.41805828533088	0.61226708621830
O	3.31375799114025	0.11098289903387	-0.35110477156097
C	4.72381710772455	-0.09668291600706	-0.27092475515177
O	2.98615921490339	-1.04289543334601	1.54688489741720
C	-2.64814344037551	-0.28861599733475	0.74094208554500
C	-3.34471558272682	0.72979941357193	-0.20475844005144
C	-2.31060770055126	1.11867967561555	-1.29814272816914
O	1.07753017156432	2.22846980767438	-0.84405349842861
H	0.12902818281315	-1.17690731362380	1.91105345890184
H	4.94642679055098	-1.16114955850392	-0.25416910472838
H	5.12279101285260	0.37453124502732	0.62426072060517
H	5.13986826335168	0.36401218917304	-1.16027512375272
H	-2.89543475628354	-0.12346327930983	1.78774013069714
H	-2.91004334513684	-1.32050431763062	0.50008387433477
H	-3.59835888071349	1.62322366972091	0.36209837610073
H	-4.25961949269573	0.33390063054118	-0.63567800017669
H	-2.32991687244179	2.17869792973739	-1.54536862303826
H	-2.44359403867069	0.56120625082127	-2.22788134830329

Ambimodal transition state ***TS3***

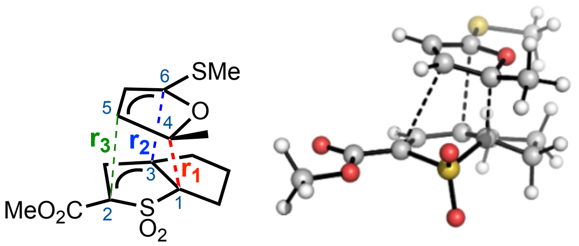

Electronic energy -1754.81744731546
Number of imaginary frequencies: 1
Lowest frequency: *-408 cm^{-1}*

O	0.37729136495095	-2.76615131359124	-1.44778681033142
S	0.74367338483125	-1.39219020291885	-1.22491783794708
C	-0.66310841163322	-0.55675347618409	-0.52362321459918
C	-2.07980432762202	-0.90678817265927	-0.92834188658780
C	-2.87736593367354	-0.68442551552073	0.37364532634931
C	-1.88058965963371	-0.94241673962602	1.52351970216891
C	-0.23865049273779	1.33731739465105	-1.00904260391946
C	1.06981709987709	1.52467044318599	-0.47457487948247
C	0.92457002312399	1.86202054204827	0.85485571401671
C	-0.44704858796023	1.97126781147231	1.06721755825944
O	-1.12309738993244	1.86834164107226	-0.07063622104126
C	-0.54325654089625	-0.71571425459654	0.87222720354126
C	0.76201681413412	-1.06362401236219	1.30120202836727
C	1.64784337671292	-1.24942003243976	0.28396944624679
C	3.07238137929154	-1.51800345576042	0.36180980850434
O	3.57517483434360	-1.76359866433066	-0.85240485061238
C	4.97183797163911	-2.01409387342461	-0.93807525698292
O	3.72945258899416	-1.51218232560060	1.37367020980253
O	1.34412374662259	-0.70162330469195	-2.33191029680810
H	-2.09116818854055	-1.95789871534621	-1.22525391493478
H	-2.46691337219835	-0.32516885023248	-1.76045399684741
H	-3.75338438060941	-1.32617724690386	0.43275857163947
H	-3.21920542517030	0.34603636792927	0.39613751165121
H	-2.05781573297544	-0.31390700478969	2.39903690321545
H	-1.94335896753066	-1.97973800456533	1.86097954051345
H	1.04925368821849	-1.17376172457542	2.33967464913666
H	5.53208494098509	-1.20891968952343	-0.46795798557412
H	5.19646019041299	-2.06309452669250	-1.99858605943490
H	5.21735041277831	-2.95786557009988	-0.45589816474970
H	1.68569456441001	1.96410863835339	1.60988068180875
S	-1.29578671142065	2.43571898024094	2.47817893143804
C	-0.67912568641433	1.58438388544138	-2.41758412893890
H	1.98425198405229	1.36494232786948	-1.01779246978237
H	-0.22957740423398	0.86290699990705	-3.09172282212362
H	-0.37828065049551	2.58737557646317	-2.71858255927779
H	-1.76273373066655	1.52548612567619	-2.47844686956722
C	-3.02918713990287	2.44615765111404	2.01884047860128
H	-3.45458774504059	1.45034966056998	2.06000269223131
H	-3.17324419944452	2.89822121320425	1.04387680558476
H	-3.49598768664555	3.06421241723667	2.78210706646592

*Methyl-(3aR,4S,7S,7aR)-4-methyl-7-(methylthio)-2,3,4,7-tetrahydro-1H-3a,7a-(epithioetheno)-4,7-epoxyindene-9-carboxylate 10,10-dioxide **IM2***

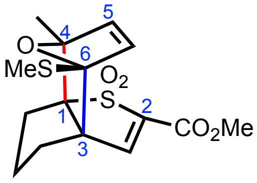

Electronic energy -1754.858389209102
Number of imaginary frequencies: 1
Lowest frequency: *-8 cm*$^{-1}$

C	0.51266865612052	0.65568875478689	-9.82305526509202
S	2.15410040634582	1.16948876921299	-9.30792156928951
C	2.72831282767774	-0.17803690720917	-8.28613555709021
C	4.14505663906893	-0.67405233511153	-8.54326540200968
C	4.13488089791394	-1.94495264148966	-8.17480910962031
C	2.70840376317848	-2.24490385083705	-7.70381599219940
O	1.96115421288117	-1.34179179105935	-8.52285857143980
C	2.45585081942801	-1.52102140049276	-6.33104371358781
C	2.58432309791913	-0.01572924110189	-6.71773503615984
C	1.28644036026913	0.66269950310264	-6.23201920881489
C	0.73934768297506	-0.28611713392099	-5.16348586960416
C	1.04549990043110	-1.66865763052530	-5.74669119138138
C	3.76890298110512	0.58216997347387	-6.00797531349921
C	4.40872857520271	-0.20032748499355	-5.15146878146262
C	5.52868864615627	0.04502684351414	-4.21929575354640
O	5.82978305743416	1.32887886388850	-4.07052920823278
C	6.86161535417067	1.58078749428230	-3.10943634215663
O	6.07724251113724	-0.84958933932365	-3.63685427302193
S	3.72619160222211	-1.83304421537764	-5.10114089201960
O	4.66297287307390	-2.80581637317315	-5.58503001966482
O	3.13013094524038	-2.05080213942411	-3.81563434988935
C	2.21065783647454	-3.65440627069526	-7.83722470016419
H	0.56381047384729	-0.24318322450357	-10.42622473871078
H	-0.16252967367949	0.51654890790633	-8.98548267452459
H	0.16093140671214	1.48658933912863	-10.43159500762417
H	4.96652303248035	-0.05585762541842	-8.87126679215265
H	4.95029386013931	-2.64677741287191	-8.13375408195634
H	1.47124940613662	1.67097522160262	-5.86392551241336
H	0.58575423498117	0.74040244178063	-7.06007984244449
H	0.36690775876886	-1.86665491057010	-6.57685335829702
H	0.96947954993900	-2.47815297145432	-5.02557882611537
H	4.03579147965766	1.62184140193440	-6.16197679246223
H	7.77697827298755	1.06906500138429	-3.39674687098727
H	7.00575163772080	2.65424127107933	-3.09845619204530
H	6.54828759202664	1.22442939394201	-2.13008510748384
H	1.14081703080424	-3.69931085623566	-7.64455654102650
H	2.73145045680700	-4.29915001090994	-7.13176852444093
H	2.39998698441940	-4.00912455585621	-8.84885589795321
H	-0.32044076409005	-0.13060456295435	-4.97304808514019
H	1.27483961391518	-0.15304529571000	-4.2226540342748

Methyl(4S,8aR)-8b-methyl-2-(methylthio)-3a,7,8,8b-tetrahydro-4,8a-epithioindeno[4,5-b]furan-4(6H)-carboxylate 9,9-dioxide ***IM1***

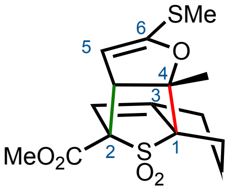

Electronic energy -1754.8710270300442
Number of imaginary frequencies: 1
Lowest frequency: *-14 cm^{-1}*

C	1.27443320806483	1.27779674883864	-9.38150997664772
S	2.74973565969113	0.59197214334788	-10.14753269988003
C	3.29617031537284	-0.46196980526696	-8.88861330945049
C	4.48785314675955	-0.56914522430469	-8.31784907881393
C	4.33369426392524	-1.43895467253290	-7.10877816198709
C	2.88054203286694	-2.00110641142648	-7.27723728218335
O	2.32617903847765	-1.27519463467225	-8.38650863574598
C	2.08923986439424	-1.53440887690316	-6.02693547882950
C	2.04368492789236	-0.02280952308089	-6.03202474709151
C	0.62069601882415	0.42793429688579	-5.98812433426280
C	-0.06050368695900	-0.74565913958155	-5.25603639760685
C	0.66077028654618	-2.00148856228092	-5.78529401580182
C	3.24960733419292	0.52428388457533	-5.91813314857640
C	4.29587700060476	-0.55612430327749	-5.82419382993692
C	5.65906030431650	-0.18760624336797	-5.32238211981270
O	5.63070970847446	0.91072012016119	-4.57961416883845
C	6.89306794495513	1.33941372208906	-4.06666807905840
O	6.65117459818141	-0.81564574687656	-5.56794997374045
S	3.36303810178730	-1.73983560188084	-4.74149481518355
O	3.96646879172389	-3.03952649511521	-4.74869301232830
O	2.97429363349521	-1.19686707013992	-3.47585500357849
C	2.79912269634188	-3.47650070885521	-7.59483409943818
H	1.53100266021663	1.79735496263034	-8.46234586045202
H	0.88321299070305	1.98876778597925	-10.10543375522089
H	0.53872753164873	0.50121364087032	-9.20112789438915
H	5.35968914889500	0.01323619025851	-8.56315416646994
H	5.07614075725955	-2.22326476214406	-6.98569685335819
H	0.49072324190479	1.39287057865439	-5.50389194534304
H	0.22549088572197	0.49498037436036	-7.00346224594179
H	0.24059619528601	-2.29828683790430	-6.74843478675851
H	0.59309223299400	-2.85514644312730	-5.11388921772306
H	3.48903685563862	1.57258674611265	-5.83194372638900
H	7.57749579885283	1.54188186242527	-4.88777591990464
H	6.69139944727095	2.24297694801508	-3.50307536313706
H	7.31470329253010	0.56986983781153	-3.42478925640921
H	1.76402473124572	-3.76248740505305	-7.77570823073629
H	3.20311878961113	-4.06922579991163	-6.77845491150196
H	3.37413469100512	-3.67426024761094	-8.49887050566912
H	-1.13220595284038	-0.77992491304662	-5.43608578769025
H	0.10940151212650	-0.65127041465467	-4.18421720411274

*Cope transition state **TS4** between **IM2** and **IM1***

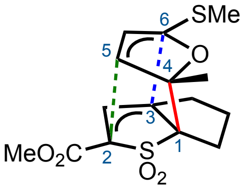

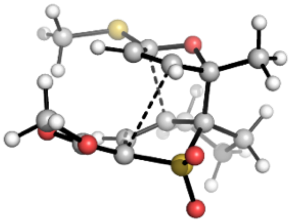

Electronic energy   -1754.806839148741
Number of imaginary frequencies: 1
Lowest frequency: *-242 cm^{-1}*

C	3.13179861399830	2.70398238346454	-8.20645433255151
S	1.83355113659111	1.51964762300746	-8.55416924531489
C	2.49999127538501	0.08973155622769	-7.89618253798386
C	3.90031859653093	-0.16689481589074	-7.69926325465700
C	3.98771742258505	-1.45268888253441	-7.34530799175592
C	2.58675747762461	-1.98846164029191	-7.24142530097450
O	1.85505096906593	-1.07592126690951	-8.08677154029598
C	2.00702394684279	-1.60493111126851	-5.77758403785533
C	1.60328880438140	-0.15545723448898	-5.78791855794099
C	0.10443984471771	-0.07331704233362	-5.80168888272577
C	-0.23909859426195	-1.26799210203816	-4.88361016362021
C	0.74133749043198	-2.36454375006009	-5.33807346489497
C	2.51001070790390	0.70832890192985	-5.19913073859502
C	3.68468113027588	0.11911675818918	-4.75517651477440
C	4.93125882920215	0.78151392155366	-5.01688601358330
O	6.01805719702185	-0.00094996283473	-5.00934001685560
C	7.16756141378779	0.58968523839448	-5.61052826706933
O	4.99016047949344	1.95259740943784	-5.34345095096353
S	3.32111465083281	-1.57422887230142	-4.52475420716662
O	4.40577922325842	-2.46957840094607	-4.81975529956207
O	2.66283229686430	-1.75908675608622	-3.26132817083687
C	2.38472133788082	-3.42072790050357	-7.66132708371506
H	3.46763415570990	2.62221234841517	-7.17350279199868
H	2.66611614885807	3.67306771500269	-8.36637594109127
H	3.95930665939133	2.58133050788997	-8.89762142624739
H	4.67993030386722	0.57162559902423	-7.77133782799034
H	4.86832658146848	-2.01651269477243	-7.09243422200901
H	-0.27546644802992	0.87775980293849	-5.43742747641091
H	-0.29001182481244	-0.25443251179488	-6.80582984847596
H	0.32061701473021	-2.89527374588666	-6.18895386271694
H	0.95531543092991	-3.09351340848026	-4.55927465036072
H	2.35285927277342	1.78040833259950	-5.18496206543707
H	7.94709040432599	-0.16227272224661	-5.57347469865450
H	7.46909612129758	1.48986815298796	-5.08147440300369
H	6.95133761259728	0.85277866649469	-6.64668123564626
H	1.33010106383677	-3.66795853177354	-7.73974559040911
H	2.85439898378549	-3.59254133204056	-8.62716434183101
H	2.84863358082905	-4.07227383804942	-6.92117289101050
H	-1.27774009340731	-1.58606795233180	-4.95404959814117
H	-0.03108921856547	-0.99987844169327	-3.84782055487227

*Stepwise transition state **TS2** in a (1,6)-Michael addition*

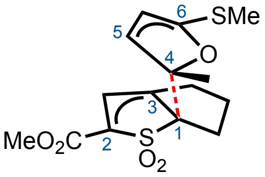

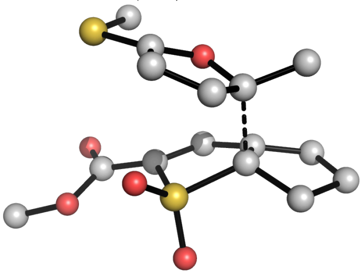

Electronic energy -1754.796102862568
Number of imaginary frequencies: 1
Lowest frequency: *-525 cm*$^{-1}$

C	2.10224524167607	3.53017297650543	-5.95165320737055
S	1.85641372371532	1.81273189633718	-5.50953918577062
C	2.45625799776207	1.69027863915996	-3.93357679247016
C	2.55802993870184	0.59576503317937	-3.07471497815884
C	3.46444839178666	0.98400894784058	-2.11157753558303
C	3.89326082326553	2.31434920711414	-2.38201778442715
O	3.15619488202726	2.71655431923650	-3.49780762550815
C	5.72705159961359	2.47661859411685	-3.18880636021233
C	5.83545313902701	3.70085990877314	-3.87585358910961
C	6.58122584521649	4.73213445476885	-3.07352956476703
C	7.03883326543538	3.95500963677996	-1.80801320930433
C	6.72049824363935	2.45648532835563	-2.05188920551971
C	5.58552573758685	3.62602511933662	-5.25535302591659
C	5.40755880149018	2.35678498799217	-5.75722717053515
C	5.26293898039410	1.96267124872656	-7.13643935048855
O	4.95522622563010	2.70452243629163	-8.04270011394115
O	5.54237750485480	0.66075392900833	-7.31481645116076
C	5.50994759108339	0.16747872179901	-8.64758487953382
S	5.86351480326020	1.23387092876571	-4.49620050770812
O	4.96120014509271	0.12562123742059	-4.37464139231932
O	7.26571278618162	0.91819871897808	-4.58470773484397
C	3.90927868485163	3.37837919022876	-1.32469838142493
H	3.13290321860835	3.82092654013566	-5.77919531277729
H	1.89085732552165	3.55632859863307	-7.01864381887067
H	1.41066631818394	4.17186584270790	-5.41615816872740
H	2.10678393667742	-0.36791565184737	-3.23526255188457
H	3.81696630278320	0.38973701349159	-1.28427773048880
H	5.98064549146972	5.60573617748752	-2.81595457543918
H	7.43659717097133	5.09307902005942	-3.64622199531426
H	6.50552694522426	4.31285455286722	-0.93109946349399
H	8.10002299621620	4.09690424499252	-1.62246204986088
H	7.60235100189711	1.91566052513798	-2.39873818038764
H	6.35163732834030	1.94439719953871	-1.16479876551450
H	5.61979335953654	4.48141684468430	-5.92030920637868
H	5.74291781619866	-0.89050954512580	-8.57645310490712
H	6.25373407220797	0.67284341676746	-9.26141918590978
H	4.52552708838884	0.30621092285017	-9.09068062350886
H	2.88853634418830	3.55091847723835	-0.98293515981152
H	4.29763631091042	4.30694113569001	-1.73759948054077
H	4.51123262038226	3.06521922397556	-0.47686258010910

*Stepwise intermediate **19** in a (1,6)-Michael addition*

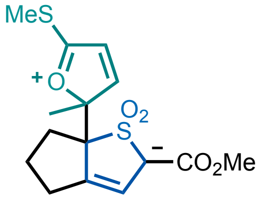

Electronic energy -1754.807970336281
Number of imaginary frequencies: 0

C	2.74357234429395	-0.86835357716163	-2.58598371099029
S	2.39631029890733	0.02026886695668	-1.06180001009881
C	3.02473975140198	1.53059347713830	-1.40385884893982
C	3.16280255542443	2.71516778061034	-0.59333265667110
C	3.84261796866615	3.58260712784168	-1.35974509996915
C	4.15270820977596	2.97980764777712	-2.69100040921573
O	3.51536822262900	1.68474230181291	-2.57589524577990
C	5.67809611585230	2.67186654334434	-2.86472196654822
C	6.48154989229079	3.89757535564862	-3.21742481891741
C	7.13347420071546	4.46225469829051	-1.98078232900080
C	6.91007628497377	3.37738173661657	-0.88848274179033
C	6.36321860113780	2.12848313752318	-1.61096925387016
C	6.69039566034231	4.04297975193654	-4.54050425837648
C	6.17615392896629	2.98605919330992	-5.37135390096211
C	5.78515233790393	3.05284620594480	-6.72395824033288
O	5.77837201633031	4.06084106175576	-7.41516767238114
O	5.37067910933085	1.85734933968093	-7.22461858758025
C	4.75844053388627	1.88778748141588	-8.50014833526085
S	5.91413671864542	1.63367038492310	-4.35963345109415
O	7.10242526067702	0.85240983412507	-4.10595038705675
O	4.74072805783379	0.86125920531912	-4.68936179871072
C	3.45867521155119	3.70155575752535	-3.84054322490464
H	2.50013266726564	-1.90363929628218	-2.36262450959727
H	2.11247250459274	-0.49102546845778	-3.38450779208384
H	3.79025651601006	-0.76175573099762	-2.85493637838407
H	2.79826133123392	2.82024033030439	0.41389457880369
H	4.14372630035361	4.58505177836330	-1.09520692202369
H	6.69257357614765	5.41147664154866	-1.66905197093810
H	8.19341205635332	4.64398012917176	-2.14941861762469
H	6.19569469619505	3.72727768000685	-0.14297021260424
H	7.82782126509421	3.14098009549656	-0.35470838693347
H	5.71394582672181	1.52050862546339	-0.97860597821408
H	7.18171352813402	1.49223995614489	-1.93947472672968
H	7.26997286325441	4.84979962465509	-4.97104014709605
H	4.48369585336058	0.86032896884636	-8.72397973078887
H	5.43970928911504	2.26508268767688	-9.26022066401445
H	3.86472408013936	2.51186745393780	-8.48633823592687
H	2.42572573672452	3.90165818376278	-3.55877181643841
H	3.97294446974760	4.63677399392852	-4.04210884342357
H	3.47444215801995	3.07337703409518	-4.72568669753055

*Transition state **TS1** of Cyclopentenone adding to **16***

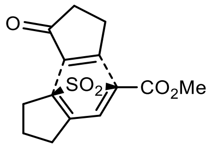

Electronic energy -1317.326477847076
Number of imaginary frequencies: 1
Lowest frequency: *-432 cm^{-1}*

O	0.94309718016449	-2.12133017936887	-1.96589017192753
S	0.89343042257213	-0.73879833526143	-1.58620141196147
C	-0.74643459145975	-0.38385234271942	-1.00309276274649
C	-2.04764075106217	-0.45748677016706	-1.73183734938938
C	-3.05170872457163	-0.65216222771489	-0.56489000640394
C	-2.23966596192211	-1.21554571160972	0.63084128856331
C	0.00523161029605	1.74271441003386	-0.70899345144713
C	1.24591572763027	1.63150448520462	-0.10092848991873
C	1.18967023451709	2.05652514812555	1.35067044990466
C	-0.30908094622476	2.10742649951841	1.66232467000666
C	-0.99097209216184	2.14225389995055	0.30556819639533
O	-2.14330520278142	2.43653730464936	0.09746287244499
C	-0.81928353629102	-0.87366748406589	0.28049895328522
C	0.42871354145620	-0.97361822281810	0.92467770484606
C	1.46729664779225	-0.57189120327351	0.10843044159625
C	2.89574035307671	-0.63822643782743	0.44655206666936
O	3.64785544700643	-0.60837568658419	-0.64918621733102
C	5.06123858865972	-0.57174535688974	-0.45158259450718
O	3.32165050861140	-0.66183473629632	1.56992229449670
O	1.43457733983924	0.20364947779085	-2.51557491116346
H	-2.02470937087840	-1.31688106076659	-2.40823303501823
H	-2.25319773391271	0.42911822882344	-2.32907262302843
H	-3.86769498455932	-1.31072925284415	-0.84971199081415
H	-3.46875089282287	0.31425093878976	-0.29565968367439
H	-2.55990366229565	-0.80248368705827	1.58599431743901
H	-2.32695249133004	-2.30210226920917	0.69374137209751
H	-0.16207283900794	1.88605041208675	-1.76499788757875
H	2.16746509241157	1.66857613561666	-0.66527708376704
H	1.74998261118828	1.39332096595888	2.00791901157279
H	1.63825252573559	3.04798127341251	1.43221428951800
H	-0.64175901087054	1.19969055437963	2.17278345456238
H	-0.62097730515942	2.95502456249683	2.26669474260407
H	0.58180678761756	-1.26675638638682	1.95385227639310
H	5.33367914269135	0.31516598415993	0.11663107684705
H	5.49816800225915	-0.53625939745108	-1.44321535501461
H	5.39009833378607	-1.46052353268496	0.08106554644944

*Transition state of Cyclopentene adding to **16***

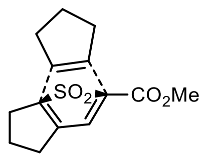

Electronic energy -1243.290066580104
Number of imaginary frequencies: 1
Lowest frequency: *-417 cm⁻¹*

O	3.46085106472434	1.16211703924792	-0.59304773356328
S	2.03344763625146	1.00219695455688	-0.64854556276796
C	1.28739691674252	2.55627705902859	-0.24908682617752
C	-0.71725484568266	1.62779355335635	-0.66330350518605
C	-0.67735620303105	0.38757970818907	-0.06078257684740
C	-1.56356051386052	0.35077287041120	1.15736283724408
C	-1.87378829630727	1.83546519352033	1.42750777498788
C	-1.67980794448097	2.53487925944101	0.06987140960511
C	1.46597722007969	3.86950716338976	-0.93940222705207
C	1.15946791121087	4.88000909233393	0.19845045321089
C	1.28641822161467	4.10745574209998	1.54079696996998
C	1.31389637036690	2.66595399629515	1.11949347340511
C	1.38916283008148	1.42884760276343	1.79603367109412
C	1.44433613103295	0.37113964573276	0.92228252325036
C	1.52530328844631	-1.05089625507048	1.26971269544582
O	1.82850401228246	-1.78640952427887	0.20129586853654
C	1.83367768279367	-3.20199566628788	0.37138061509757
O	1.29842881749965	-1.49758846592846	2.36330779547814
O	1.51747616107363	0.35007828413534	-1.81290059371821
H	-0.48645710926285	1.76420400935525	-1.71034893164877
H	-0.38096419693573	-0.50776511595792	-0.58785791052507
H	-1.10419096763061	-0.15160569120454	2.01036059252856
H	-2.47178114380195	-0.20531983586536	0.90901190117920
H	-1.16337081497537	2.23169076356054	2.15103071002001
H	-2.87032301322881	1.98571378753021	1.83543399502366
H	-2.61777908041562	2.56879965574549	-0.49029685542919
H	-1.32633824185170	3.56066325166786	0.16690071854720
H	2.49631224015923	3.95184931765032	-1.29491214246175
H	0.81206005225216	3.98902053937108	-1.80238052389864
H	0.14670405445191	5.25856990103881	0.08094194368902
H	1.83128914574440	5.73326715398583	0.15902820980593
H	0.46768800897425	4.33038304017918	2.22500755021277
H	2.21626604919683	4.35127447986249	2.05693241012126
H	1.32825029521895	1.29290530449348	2.86647979338242
H	2.66582559640865	-3.50303659168895	1.00351684051237
H	0.89838725064047	-3.53014098594873	0.81919499359578
H	1.94442741421767	-3.61865623671115	-0.62401935666793

*Transition state TS of DMAD (Dimethyl acetylenedicarboxylate) adding to **16***

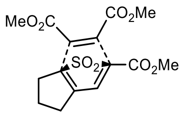

Electronic energy -1581.07365037717
Number of imaginary frequencies: 1
Lowest frequency: *-340 cm⁻¹*

O	2.29000261902469	0.66083020483534	-2.76783112068203
C	2.11495512197006	0.39067057598215	-1.61454937050807
C	0.94914045323928	0.81212483194905	-0.85299890907927
C	0.21929844644153	1.56079263906446	-0.21888473983965
C	-1.71764424501171	0.47121119645561	0.23299149378956
C	-2.76140771352670	1.22459342107543	0.99155008249963
C	-3.59741921114196	1.82009642349164	-0.16462431263996
C	-3.40244816174875	0.86599096340644	-1.36979345278066
C	-2.10934003684686	0.17657578692074	-1.04801247528703
C	-1.27544489664175	-0.75634733353252	-1.71897709213645
C	-0.25798996168458	-1.19796294144359	-0.91984393554776
C	0.78323738884042	-2.20791739615764	-1.21654459634720
O	1.21251444242666	-2.75701686754924	-0.08915670808448
C	2.33220986116590	-3.63665029676206	-0.17228226923895
O	1.20059890300957	-2.45261727127953	-2.31448219139667
S	-0.73429629562283	-0.88472042379803	0.79563239645933
O	-1.64805004489659	-1.91100893943341	1.20996923793085
O	0.32737865544038	-0.56071797772288	1.68874131773587
C	-0.00603297311074	2.77702464523126	0.55900696651587
O	0.90328446845784	2.85928845850442	1.51941251321918
C	0.81642790168466	4.00372421912412	2.37060711336192
O	-0.87880885099574	3.57546272530928	0.36270702749333
O	2.94642342913560	-0.31802500083400	-0.85779195133371
C	4.14274312003908	-0.75177404767508	-1.51186407485754
H	-2.36435509957486	1.97912868773502	1.66702328391512
H	-3.32869732352609	0.50500193916266	1.59027923189658
H	-3.18937284892594	2.79802402375087	-0.40813853126762
H	-4.64219601382790	1.93503081282465	0.11011166781560
H	-4.18624577875841	0.10514661947024	-1.40686329194930
H	-3.39471250168183	1.38026322076558	-2.32776374098917
H	-1.37268266317391	-1.04734294609590	-2.75521937231844
H	3.16541034387975	-3.12900939066923	-0.65268269378316
H	2.07477178965785	-4.52971560525980	-0.73634618399205
H	2.58555975837010	-3.88931785753366	0.85158256605437
H	0.93795807929325	4.91286089913240	1.78670931316411
H	-0.14413054968474	4.02040731145879	2.88048953683904
H	1.62521925127952	3.89690472478322	3.08441143240077
H	4.67803915455716	0.10323264967540	-1.91740231411690
H	3.89415850115904	-1.44568563957098	-2.31293889397952
H	4.73411548130951	-1.24140704479120	-0.74562495893551

*Transition state TS of 2-Butyene adding to **16***

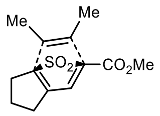

Electronic energy   -1203.935107700760
Number of imaginary frequencies: 1
Lowest frequency: *-421 cm*$^{-1}$

C	2.21991241161285	-1.99915570329975	-1.43452935884331
C	1.17593982025562	-1.17985867892738	-0.80465414897713
C	0.68324947776923	-0.19159229193584	-0.26201942760503
C	-1.18164176244661	-0.85155140044892	0.78696381291165
C	-1.86222614028934	0.14599632058465	1.67341573378750
C	-2.93978377482232	0.72680460831444	0.72578823817204
C	-3.21655379562909	-0.37083743026068	-0.33044829060485
C	-1.97771828758100	-1.21967836151975	-0.27338023748425
C	-1.51519134917400	-2.35355260055878	-0.96787429322344
C	-0.37755534635529	-2.87258221999552	-0.40073953658623
C	0.28214679796300	-4.12513787295089	-0.79546193880446
O	0.99154476971331	-4.62287364071350	0.21496397951287
C	1.75062644540353	-5.80084728252551	-0.05186570022018
O	0.21502554561284	-4.62022105930358	-1.88844694587289
S	-0.30576644897440	-2.30699250409265	1.30008908989368
O	-1.20688271915353	-3.09707543943952	2.09699610034261
O	0.99986631182827	-2.11501414453315	1.85059854115418
C	0.72619292041301	1.19995796866697	0.21378271072087
H	1.84750757659130	-2.57432134180550	-2.28054812438132
H	2.62797150143179	-2.69290524809730	-0.69618124805439
H	3.03161077720501	-1.35626686315138	-1.77434296882979
H	-2.30850102937913	-0.39343796029467	2.51440570978120
H	-1.19845101637594	0.90054771762756	2.08900875357786
H	-3.83567948228229	1.02175437233761	1.26559580532758
H	-2.54468543849284	1.61351767919925	0.23191023875704
H	-4.06865600844726	-0.98861593281147	-0.03782520785179
H	-3.42751475349187	0.03533130547461	-1.31757486813193
H	-1.94855062137884	-2.76190649659047	-1.86930026917771
H	1.09266113163554	-6.62175499364037	-0.32645624629969
H	2.45554854478723	-5.61347218358805	-0.85995770005099
H	2.27925505805930	-6.02694240639998	0.86758069793209
H	1.54504607865839	1.72650300263191	-0.27458652646665
H	0.90739018757007	1.21384899359471	1.28925901197894
H	-0.20185738223654	1.73376208845293	0.01535461361589

Cyclopentenone

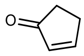

Electronic energy -269.341555145941
Number of imaginary frequencies: 0

O	2.08200032402370	-1.29840356996451	0.54239247351541
C	1.12739855712536	-0.60709256718795	0.28279757840355
C	1.12601255107895	0.85682060933351	0.08766253038041
C	-0.10852587930201	1.26818641361419	-0.19088585033021
C	-1.11928148924110	0.15120987812825	-0.21958828605854
C	-0.29917206979822	-1.10358320702492	0.09532749827716
H	2.01955912810719	1.45676746696406	0.16894207588842
H	-0.39013882407935	2.29484751384865	-0.38189850743190
H	-1.90500186826176	0.33205435136264	0.51425849325499
H	-1.60517059502031	0.09972827556703	-1.19432769819786
H	-0.31077523449202	-1.83776552572368	-0.70899902840226
H	-0.61701460014042	-1.61266963891727	1.00420872070084

Cyclopentene

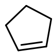

Electronic energy -195.30271243175
Number of imaginary frequencies: 0

C	-0.28391080991924	1.32151056734849	-0.08716193191933
C	0.96616558493181	0.94003559226674	0.13499849979860
C	1.07787488692027	-0.55688560983466	0.29164899202340
C	-0.28170543828289	-1.05999241505582	-0.23447774362457
C	-1.23380591707182	0.14879796591029	-0.12065517922392
H	-0.61195840600364	2.34723620932421	-0.18824122762833
H	1.81068204404644	1.60751346356629	0.24116845951327
H	1.22127191622190	-0.81443258647338	1.34511062287513
H	1.92090405630831	-0.98020377569627	-0.25456515561980
H	-0.17825773985298	-1.33306742513224	-1.28454357418672
H	-0.64157617916058	-1.93590171213889	0.30093589741680
H	-1.94393075455251	0.19990331369292	-0.94552787374541
H	-1.82174324358507	0.11559641222233	0.80121021432089

DMAD (Dimethyl acetylenedicarboxylate)

Electronic energy -533.093950828534
Number of imaginary frequencies: 0

O	-2.33627869373729	1.91213475327484	-0.70930620668476
C	-1.91962695219077	0.79783026201153	-0.57768152344362
C	-0.51117290528851	0.45069012142946	-0.65979148574820
C	0.65245380148261	0.18377865107058	-0.69123841896097
C	2.06098984329333	-0.17275033048854	-0.71053157999503
O	2.56050062598575	-0.12109684482581	0.51396711359289
C	3.93566029148565	-0.49310233472418	0.63766089000107
O	2.66136706209204	-0.47569102656789	-1.70047533971629
O	-2.65046306701060	-0.27776713160355	-0.32915428291110
C	-4.05730935813565	-0.04797752978387	-0.21427394484843
H	4.07116081415990	-1.51927814812128	0.30401239597776
H	4.17030260090718	-0.39813540684683	1.69162323147813
H	4.55757498613396	0.17106762987770	0.04271652152469
H	-4.50113737400452	-1.02335431966522	-0.05343994789117
H	-4.25763274461977	0.61014883349491	0.62812150630847
H	-4.43647893055330	0.40340282146815	-1.12779892868343

2-Butyne

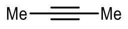

Electronic energy -155.963571135872
Number of imaginary frequencies: 0

C	-2.04769980301133	0.06265901973518	0.13093529283583
C	-0.59591001630911	0.01851754314206	-0.02948598118000
C	0.59565086131020	-0.01889091733076	-0.15986101585228
C	2.04760744638262	-0.06287488614607	-0.31866595737612
H	-2.42588715527144	1.06740852908589	-0.05276248566579
H	-2.53335674209911	-0.61633090232365	-0.56816028152954
H	-2.33274607280094	-0.22667968325886	1.14136516549209
H	2.31972201110112	-0.09327614205021	-1.37291599231968
H	2.51082412710407	0.81640518478826	0.12652703450304
H	2.46179534359392	-0.94684774564183	0.16422422109244

*Transition state **TS(SO_2 Extrusion)** of SO_2 extrusion after **IM1** formation*

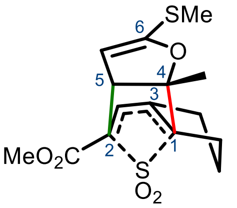

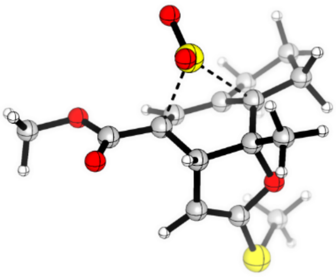

Electronic energy -1754.845814914299
Number of imaginary frequencies: 2
Lowest frequency: *-245 cm^{-1} (second small residual: -16 cm^{-1})*

C	-2.653227	0.629088	-0.721808
C	-1.347321	1.045943	-0.083027
C	-0.307095	1.663836	-1.068352
C	0.811490	0.647444	-1.031894
C	0.528763	-0.241343	-0.094115
S	1.501591	-1.477792	0.628883
C	1.310100	-1.075340	2.372927
O	-0.724350	-0.157316	0.429806
C	0.261113	2.976770	-0.522011
C	1.103541	3.696832	-1.511510
O	1.846923	4.651487	-0.959427
C	2.779730	5.277930	-1.839743
O	1.127660	3.425782	-2.683121
C	0.616958	3.011011	0.846373
C	-0.282453	2.431829	1.707706
C	-1.462427	1.890459	1.166865
S	-1.723948	3.886452	-0.268733
O	-1.661270	5.113668	0.468167
O	-2.440316	3.913343	-1.512033
C	-2.403002	1.517218	2.279020
C	-1.828030	2.244730	3.508397
C	-0.323899	2.394742	3.210149
H	-3.256604	0.072993	-0.005509
H	-2.438392	-0.020897	-1.569751
H	-3.211241	1.493331	-1.074873
H	-0.731516	1.831379	-2.056315
H	1.750451	0.748906	-1.548929
H	1.768791	-0.116079	2.595318
H	1.842438	-1.863144	2.901513
H	0.263391	-1.086444	2.659903
H	2.256589	5.772096	-2.654327
H	3.457674	4.531127	-2.248626
H	3.322809	5.996051	-1.236577
H	1.442829	3.615804	1.191332
H	-2.330777	0.428657	2.394875
H	-3.446026	1.747524	2.072899
H	-2.280275	3.231279	3.592044
H	-2.014839	1.703012	4.432288
H	0.218957	1.505219	3.544730
H	0.135776	3.259545	3.682484

Methyl 8b-methyl-2-(methylthio)-3a,7,8,8b-tetrahydro-6H-indeno[4,5-b]furan-4-carboxylate
Extruded product

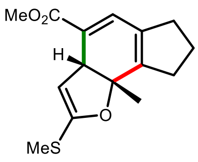

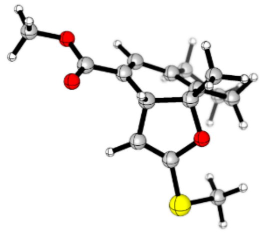

Electronic energy -1206.243004526657
Number of imaginary frequencies: 0

C	-0.63713111725694	-0.15880950431957	2.74803701420508
C	-0.66647686017654	-0.31994392682663	1.24542710887138
C	0.37372982203633	-1.31639319571506	0.67160791669243
C	-0.43053954958303	-1.94383257212752	-0.45437696226852
C	-1.70072008888877	-1.62189591860845	-0.25820500152584
S	-3.10933890471555	-1.95972458015060	-1.20029815513347
C	-3.92157140639972	-0.35629787841854	-1.23438486220838
O	-1.93692130379933	-0.90844099175971	0.88343964562379
C	1.61860790476026	-0.67355075429843	0.12413230508990
C	2.76811547287316	-1.58264015180225	-0.02376903973554
O	3.89830276440216	-0.98305789877335	-0.40710896186934
C	5.01273667922733	-1.85171503570928	-0.59313331402848
O	2.69725377090881	-2.77113091467336	0.17380332311212
C	1.61845726814576	0.57741204880536	-0.34799688496977
C	0.40227959431748	1.36878303062096	-0.26391449125616
C	-0.62235636564980	0.98448478203326	0.49720211356152
C	-1.75823087885869	1.96902490437287	0.45239412834383
C	-1.41991200530763	2.79803296116643	-0.80218844548990
C	0.10850811587385	2.66443825111563	-0.97727253138061
H	-1.46083803114738	0.47356874020691	3.07612265782230
H	0.30408099515537	0.29724886565737	3.05197953543371
H	-0.72995665620832	-1.13497634904226	3.22348509762417
H	0.64623337210553	-2.06115010679225	1.42252422532161
H	-0.02276980333665	-2.49708553974176	-1.28315406002250
H	-3.25428448662094	0.40061059299612	-1.63778464829509
H	-4.28021649354669	-0.08007511448769	-0.24823361019762
H	-4.76734338352015	-0.48674324523160	-1.90614823249416
H	5.23601013914447	-2.38504904340338	0.32849198212572
H	4.80020552893456	-2.57394270614486	-1.37913655620242
H	5.84381178156374	-1.21531171069759	-0.87572609498948
H	2.50303334633956	0.98729085975156	-0.81693297033543
H	-2.72750105403682	1.47815546445825	0.39107017322781
H	-1.76878879984149	2.58251225890082	1.35773491762488
H	-1.75598997343498	3.82956929949092	-0.73450509662605
H	-1.91221233253511	2.34519963451702	-1.66460856152514
H	0.64544615910883	3.49509093652449	-0.51320847655823
H	0.40618677996734	2.63014449810615	-2.02526521756815

SO_2 (aq)

Electronic energy -548.629228739794
Number of imaginary frequencies: 0

O	1.22498139539243	-0.24090001836912	0.00000000000000
S	-0.00003123863948	0.48195575731008	0.00000000000000
O	-1.22495015675295	-0.24105573894096	0.00000000000000

Benchmarking geometries of TS3

CPCM(Toluene)-SCS-MP2/def2-TZVPP-level of theory

O	0.40253075285234	-2.84425167352221	-1.55916183138443
S	0.72040726216416	-1.44637661616542	-1.35493054242986
C	-0.71900858395951	-0.66077905227191	-0.62399315467089
C	-2.12980779028791	-1.07502447579114	-1.01797958440024
C	-2.92510577708787	-0.86206005745266	0.29004444250142
C	-1.91705197860133	-1.15023941581406	1.42599103365808
C	-0.37623370238075	1.23148519033388	-0.95186574738010
C	0.96180497208863	1.42844586367667	-0.47175465876849
C	0.85558188346282	1.71170261343643	0.88457719176233
C	-0.50229008960422	1.75906650119954	1.18232676527965
O	-1.24561189711033	1.71341741399492	0.04315245169223
C	-0.58802561765299	-0.87879171867410	0.77536804376855
C	0.74189505092005	-1.15680700512706	1.19249590135666
C	1.63256184840010	-1.24822435955312	0.15783838199077
C	3.08610030968286	-1.38832820471824	0.30267845705594
O	3.66690260169740	-1.75263729492694	-0.85087421373085
C	5.09871860869097	-1.90430679575522	-0.78893289441205
O	3.68520271798248	-1.19703446381815	1.34249652118349
O	1.29437067465353	-0.73199628316268	-2.47136588691070
H	-2.10496448101482	-2.13428496728338	-1.28778797744582
H	-2.54240077972478	-0.52697150176996	-1.86183217199645
H	-3.80394323674821	-1.50318576954409	0.34116675959326
H	-3.25721645241660	0.17294613216264	0.34659406408246
H	-2.09858595286049	-0.54597362869905	2.31693334920801
H	-1.95548380862121	-2.20227862915876	1.72233772648440
H	1.04293508494268	-1.25611506807876	2.22813351562977
H	5.56161086048385	-0.96833855345410	-0.48790111491673
H	5.39938409690859	-2.17638998887300	-1.79394047050022
H	5.36132540514675	-2.68760822339387	-0.08299016131651
H	1.65207456540819	1.82698386615951	1.60263001601197
S	-1.28215972644554	2.09949815821288	2.69706060297720
C	-0.84519442905726	1.55120912402459	-2.34168810856692
H	1.85503143340236	1.35763842071348	-1.06818350130076
H	-0.29797516074047	0.94939577419794	-3.06236688651673
H	-0.65936356079399	2.60546933065254	-2.54678856356380
H	-1.91142186072639	1.36419292975772	-2.44161531619689
C	-2.74664365992494	2.99796852422008	2.11833651716367
H	-3.37776141736656	2.35644821541116	1.51189298979234
H	-2.45258375071981	3.88396147101604	1.56258630367527
H	-3.27960441504169	3.28817521783781	3.02130975154096

CPCM(Toluene)-B3LPYP/def2-TZVPP-level of theory

O	0.42550573475893	-2.79263431788415	-1.68521730440302
S	0.77579826272699	-1.41484201880183	-1.38734630792158
C	-0.68722994063103	-0.64232235065463	-0.61471743961583
C	-2.09630268945554	-1.03986165603811	-1.02095255912598
C	-2.89974342144559	-0.92065964082427	0.29841930799370
C	-1.87444848017821	-1.13286992387299	1.44391019361617
C	-0.37443954390332	1.30148110257847	-0.93124225243740
C	0.95306715523270	1.55391546955597	-0.46006242633124
C	0.86928957291762	1.95509610296942	0.85887162168826
C	-0.48678434467197	2.05233466016911	1.15876051435554
O	-1.23857738359171	1.81675349596600	0.07469115696337
C	-0.54501754900988	-0.91455990026169	0.77273719930301
C	0.76223518826524	-1.21348964275148	1.16975160547683
C	1.68235769722971	-1.30547820946428	0.14302755144376
C	3.10377349804849	-1.54014989945894	0.30294261050536
O	3.71290944110711	-1.83960097951246	-0.86322554302208
C	5.12576114688791	-2.08738471185694	-0.80216562458169
O	3.69310373363512	-1.47831238580502	1.36737610535677
O	1.35618766393853	-0.64860343136653	-2.47186095767327
H	-2.06143339325389	-2.07563065974299	-1.36831445554047
H	-2.52073093149776	-0.44854277776622	-1.82882264404681
H	-3.71751402475191	-1.63935440974479	0.33674397658779
H	-3.33985637969534	0.07316892821596	0.36779740626350
H	-2.05191987597239	-0.45922171715944	2.28665291245485
H	-1.92970188379002	-2.15032525780816	1.84064307893826
H	1.05308714735851	-1.39002718171134	2.19763641688081
H	5.65238591880332	-1.21063751468617	-0.42747129214588
H	5.42694544579760	-2.30310066244793	-1.82282114787253
H	5.33687476103587	-2.93794693633713	-0.15564012594528
H	1.67618890949389	2.15793723291626	1.54277183334626
S	-1.22009269515297	2.51521972755366	2.65350658666478
C	-0.84727784297947	1.62935705688893	-2.31887335807567
H	1.84269942343020	1.43405450139931	-1.05137011832615
H	-0.25912871714697	1.07542888404211	-3.04572390731690
H	-0.72207435525665	2.69691329552717	-2.50780422529217
H	-1.89930253835246	1.38496401701428	-2.44808426587756
C	-2.91922847685448	2.91878398648498	2.15098003537726
H	-3.43755201989135	2.03927584166654	1.78155431570664
H	-2.91813173709514	3.70787452550400	1.40323725934468
H	-3.40168247608963	3.27299835750526	3.05970226728387

CPCM(Toluene)-PBE0-D3BJ/def2-TZVPP-level of theory

O	0.40287945311974	-2.83486675088436	-1.58879441646645
S	0.73437345549163	-1.44978614889360	-1.34509458411484
C	-0.70929018684513	-0.68833210281314	-0.60870368209566
C	-2.11485248551905	-1.04909381839581	-1.00948896698656
C	-2.90938893976385	-0.82896809508747	0.29024436200822
C	-1.90652923295847	-1.04787253962253	1.44102421266177
C	-0.34349928935848	1.31231607568420	-0.96418746578426
C	0.98279134446793	1.47016380711497	-0.49410162069821
C	0.90899433825081	1.80261202781791	0.84624264600759
C	-0.43888548991432	1.92653050209346	1.14342283978635
O	-1.18610619539665	1.79725330914496	0.04191846441746
C	-0.57712129227244	-0.87317418702210	0.77770450075423
C	0.73879123632689	-1.13937972093094	1.19060801920442
C	1.63816535673421	-1.27793937994411	0.16332828071024
C	3.06759083652259	-1.44662773422719	0.31437247395568
O	3.66334829918090	-1.77433944843654	-0.83785680942303
C	5.07393380863312	-1.95241153972615	-0.77832893436879
O	3.66390825509834	-1.30706292127762	1.36143378428048
O	1.30447055233192	-0.72331959495190	-2.45167033931082
H	-2.11297442156183	-2.10324833066547	-1.30478917778013
H	-2.50887006785876	-0.48275258932956	-1.85162175439481
H	-3.77266700024387	-1.49046155841757	0.35773260912619
H	-3.28206548583693	0.19499711904846	0.30993396890802
H	-2.06851578627460	-0.36006680986822	2.27686357556356
H	-1.98470401728179	-2.06124885027487	1.84692513259010
H	1.04418131424029	-1.23358470169050	2.22595195649181
H	5.56267088998604	-1.03557866422386	-0.44779692440984
H	5.38236300001016	-2.20221809762381	-1.78982537653253
H	5.32972102586930	-2.76053839632858	-0.09287199957434
H	1.71923597538771	1.91704743443039	1.54823875894577
S	-1.17418337819072	2.32842160913367	2.64510537562006
C	-0.83043224511025	1.58269979454386	-2.34316821596987
H	1.86778396951411	1.33273139705116	-1.09093239272429
H	-0.25362867215249	0.99288743932711	-3.05223337053021
H	-0.70240910041510	2.64100954509287	-2.58028641391864
H	-1.88633038230002	1.33809490697369	-2.44469039511864
C	-2.83217184397548	2.79553701201221	2.12947674144739
H	-3.37561951746461	1.94267831566316	1.73087871247211
H	-2.79221657592059	3.60093712330743	1.39892752081295
H	-3.32274150455027	3.14695556219630	3.03610690443753

Benchmarking geometries of IM1 and IM2

IM1: CPCM(Toluene)-SCS-MP2/def2-TZVPP-level of theory

C	1.19666693157944	1.12597591658795	-9.95080234076371
S	2.75120000367621	0.34432050062149	-10.47075288452827
C	3.18695508875493	-0.43768147169259	-8.96998573402106
C	4.37988450737339	-0.45302456360453	-8.35544161019092
C	4.28695357905226	-1.31510470092840	-7.13221230576274
C	2.84342201649659	-1.91039532876460	-7.25984078173826
O	2.21061006969392	-1.19474033084623	-8.36809882014947
C	2.10218314669226	-1.49861484050058	-5.96999631145721
C	2.04071636912248	0.00818036136455	-5.92695601903196
C	0.61551832196696	0.44937595073432	-5.80810443108486
C	-0.01596087463253	-0.75284922177114	-5.07122239182497
C	0.68678893941051	-1.98966816054175	-5.67232523007124
C	3.25706417287428	0.57470000261074	-5.82777293799920
C	4.31350791163344	-0.49440438514712	-5.81133433213433
C	5.69396004610589	-0.12738115194101	-5.33488596155040
O	5.63244245857900	0.74242495686396	-4.32094912663401
C	6.91266820797202	1.12004314894887	-3.76739516093761
O	6.71759166914308	-0.57673878604297	-5.80019000850817
S	3.42943548355409	-1.73191734956713	-4.71960787019581
O	4.07258994462802	-3.02327961938231	-4.78102592258285
O	3.05160529151125	-1.22160935760328	-3.42559838453434
C	2.79953876318973	-3.38484044859531	-7.60709018459467
H	1.37557584586589	1.81115598056726	-9.12595698381488
H	0.83748046467907	1.68030254203334	-10.81531338082886
H	0.46647103744567	0.37284585703699	-9.67134671766444
H	5.25307018434025	0.09268275603518	-8.67285966076564
H	5.04083340188525	-2.09904300782534	-7.08298167445476
H	0.50119253182877	1.39435387716639	-5.27945201953406
H	0.17457852406342	0.54881743797150	-6.80391054862282
H	0.21708966952913	-2.26897369097812	-6.61770910305102
H	0.66251541414460	-2.85830379655444	-5.01482718251241
H	3.47682722337957	1.62347710367219	-5.69564067851385
H	7.51856275440303	1.60133622364762	-4.52996316388784
H	6.68231542267890	1.80929122100465	-2.96384604234599
H	7.42488211398977	0.24030218759778	-3.38835329340392
H	1.76947147148838	-3.69636699485855	-7.77725937448323
H	3.23137556699027	-3.98134729863629	-6.80678241384606
H	3.36824686218487	-3.55092399843891	-8.52226233814336
H	-1.09773152282828	-0.79724141727552	-5.18648677670681
H	0.21517898555408	-0.68338310296867	-4.00784789712368

IM2: CPCM(Toluene)-SCS-MP2/def2-TZVPP-level of theory

C	0.74761929431290	1.23418092094260	-9.54855974042862
S	2.54475624198686	1.17209541508925	-9.30819722286305
C	2.80851997368604	-0.23962099700751	-8.22758778160533
C	4.23341448862537	-0.75000390290905	-8.37714261694330
C	4.18261671757581	-2.04579538411491	-8.05165798655068
C	2.73266226576265	-2.33631739128709	-7.69012288897881
O	2.03209232736080	-1.40439400282698	-8.56309550927013
C	2.41497306521846	-1.65094328875605	-6.31271390588801
C	2.50725093708596	-0.13364175911720	-6.68409011199349
C	1.12714051536044	0.45281631665718	-6.32242671301913
C	0.60494454931912	-0.47969766992415	-5.22478233400495
C	0.99071478820750	-1.85835927860266	-5.77202044889781
C	3.62082928072347	0.49538843858832	-5.90849141533968
C	4.29132029907555	-0.28227316012820	-5.05082582595804
C	5.43482799410025	0.07936839047102	-4.17663742596554
O	5.80328619233202	1.35496366455805	-4.35121974037701
C	6.91142394623596	1.77479116244126	-3.52476662413299
O	5.95687393896673	-0.69567563595661	-3.40782105103655
S	3.64045464865403	-1.93598399920752	-4.99751780461332
O	4.63085193159039	-2.90357700829151	-5.40566234273401
O	2.97354895601746	-2.12933950387218	-3.73206186625060
C	2.25438199487029	-3.75089039175442	-7.88206736957801
H	0.35044035418074	0.22424994044819	-9.60619170945157
H	0.24641055907844	1.80455958472773	-8.77345128181319
H	0.60547502270410	1.72629964751433	-10.50857870231007
H	5.08755336889228	-0.13997620666010	-8.62975865710663
H	4.98953327045321	-2.75578119883356	-7.97798704312534
H	1.19043582080239	1.50085902639508	-6.02877446743403
H	0.46915560345252	0.37110702520302	-7.18432392765207
H	0.34336661511963	-2.10222169457628	-6.61527990483150
H	0.93130104764840	-2.65965056844871	-5.03844402581229
H	3.86281325325232	1.54211436158111	-6.05032255637753
H	7.78858218800529	1.17375323408168	-3.74698792754698
H	7.07979235538044	2.81359128288312	-3.78271686413188
H	6.65083370637872	1.67386481640247	-2.47501647773313
H	1.18550941369387	-3.83730220476796	-7.69838205242341
H	2.78691009885504	-4.40565658149620	-7.19214989805214
H	2.46463205177382	-4.07059806880806	-8.90188374875618
H	-0.46726709272997	-0.37620562387573	-5.06057230537032
H	1.11685401599019	-0.29237470696172	-4.28004472364162

2D PES scan

$r_{C1\text{-}C4}$ [Å]	$r_{C2\text{-}C5}$ [Å]	**electronic energy [E_h]**
1.50000000	2.50000000	-1753.40435822
1.50000000	2.60000000	-1753.40286959
1.50000000	2.70000000	-1753.40183641
1.50000000	2.80000000	-1753.40090120
1.50000000	2.90000000	-1753.39999273
1.50000000	3.00000000	-1753.43483329
1.50000000	3.10000000	-1753.43793146
1.50000000	3.20000000	-1753.44012874
1.50000000	3.30000000	-1753.44140297
1.50000000	3.40000000	-1753.44202609
1.60000000	2.50000000	-1753.40507921
1.60000000	2.60000000	-1753.41351806
1.60000000	2.70000000	-1753.42130540
1.60000000	2.80000000	-1753.42760854
1.60000000	2.90000000	-1753.43236465
1.60000000	3.00000000	-1753.43634531
1.60000000	3.10000000	-1753.43961486
1.60000000	3.20000000	-1753.44201859
1.60000000	3.30000000	-1753.44355004
1.60000000	3.40000000	-1753.44430946
1.70000000	2.50000000	-1753.40018121
1.70000000	2.60000000	-1753.40923218
1.70000000	2.70000000	-1753.41670938
1.70000000	2.80000000	-1753.42280041
1.70000000	2.90000000	-1753.42763240
1.70000000	3.00000000	-1753.43160120
1.70000000	3.10000000	-1753.43498852
1.70000000	3.20000000	-1753.43756233
1.70000000	3.30000000	-1753.43927984
1.70000000	3.40000000	-1753.44030181
1.80000000	2.50000000	-1753.39265818
1.80000000	2.60000000	-1753.40104003
1.80000000	2.70000000	-1753.40845286
1.80000000	2.80000000	-1753.41479479
1.80000000	2.90000000	-1753.41976245
1.80000000	3.00000000	-1753.42361627
1.80000000	3.10000000	-1753.42697285
1.80000000	3.20000000	-1753.42961542
1.80000000	3.30000000	-1753.43157831
1.80000000	3.40000000	-1753.43276630
1.90000000	2.50000000	-1753.38329161
1.90000000	2.60000000	-1753.39154043
1.90000000	2.70000000	-1753.39888020
1.90000000	2.80000000	-1753.40524003
1.90000000	2.90000000	-1753.41029612
1.90000000	3.00000000	-1753.41399086
1.90000000	3.10000000	-1753.41741018
1.90000000	3.20000000	-1753.42013946
1.90000000	3.30000000	-1753.42215152

1.90000000	3.40000000	-1753.42355235
2.00000000	2.50000000	-1753.38224327
2.00000000	2.60000000	-1753.38303540
2.00000000	2.70000000	-1753.38336979
2.00000000	2.80000000	-1753.38351540
2.00000000	2.90000000	-1753.38338803
2.00000000	3.00000000	-1753.38250873
2.00000000	3.10000000	-1753.38069395
2.00000000	3.20000000	-1753.37532029
2.00000000	3.30000000	-1753.37318662
2.00000000	3.40000000	-1753.37069179
1.50000000	2.40000000	-1753.40636262
1.50000000	2.30000000	-1753.40903753
1.50000000	2.20000000	-1753.41276558
1.50000000	2.10000000	-1753.41799000
1.50000000	2.00000000	-1753.42443642
1.50000000	1.90000000	-1753.43179621
1.50000000	1.80000000	-1753.43939592
1.50000000	1.70000000	-1753.44651407
1.50000000	1.60000000	-1753.45095288
1.50000000	1.50000000	-1753.44970755
1.60000000	2.40000000	-1753.40893183
1.60000000	2.30000000	-1753.41131713
1.60000000	2.20000000	-1753.41476004
1.60000000	2.10000000	-1753.41959915
1.60000000	2.00000000	-1753.42592864
1.60000000	1.90000000	-1753.43284109
1.60000000	1.80000000	-1753.44007803
1.60000000	1.70000000	-1753.44693716
1.60000000	1.60000000	-1753.45110010
1.60000000	1.50000000	-1753.44961640
1.70000000	2.40000000	-1753.40612542
1.70000000	2.30000000	-1753.40827452
1.70000000	2.20000000	-1753.41111306
1.70000000	2.10000000	-1753.41544019
1.70000000	2.00000000	-1753.42114787
1.70000000	1.90000000	-1753.42776768
1.70000000	1.80000000	-1753.43506313
1.70000000	1.70000000	-1753.44192467
1.70000000	1.60000000	-1753.44610073
1.70000000	1.50000000	-1753.44450758
1.80000000	2.40000000	-1753.40104676
1.80000000	2.30000000	-1753.40261528
1.80000000	2.20000000	-1753.40485050
1.80000000	2.10000000	-1753.40855490
1.80000000	2.00000000	-1753.41370751
1.80000000	1.90000000	-1753.41977909
1.80000000	1.80000000	-1753.42642588
1.80000000	1.70000000	-1753.43297176
1.80000000	1.60000000	-1753.43679450
1.80000000	1.50000000	-1753.43501958
1.90000000	2.40000000	-1753.39549689
1.90000000	2.30000000	-1753.39635939
1.90000000	2.20000000	-1753.39795952

1.90000000	2.10000000	-1753.40082064
1.90000000	2.00000000	-1753.40523190
1.90000000	1.90000000	-1753.41071952
1.90000000	1.80000000	-1753.41663713
1.90000000	1.70000000	-1753.42269896
1.90000000	1.60000000	-1753.42625502
1.90000000	1.50000000	-1753.42415576
2.00000000	2.40000000	-1753.38994504
2.00000000	2.30000000	-1753.38989869
2.00000000	2.20000000	-1753.39131023
2.00000000	2.10000000	-1753.39347870
2.00000000	2.00000000	-1753.39691295
2.00000000	1.90000000	-1753.40154958
2.00000000	1.80000000	-1753.40672621
2.00000000	1.70000000	-1753.41224785
2.00000000	1.60000000	-1753.41539403
2.00000000	1.50000000	-1753.41298841
2.10000000	3.40000000	-1753.39367106
2.10000000	3.30000000	-1753.39434729
2.10000000	3.20000000	-1753.39579123
2.10000000	3.10000000	-1753.39616410
2.10000000	3.00000000	-1753.39610113
2.10000000	2.90000000	-1753.39549006
2.10000000	2.80000000	-1753.39428464
2.10000000	2.70000000	-1753.39271954
2.10000000	2.60000000	-1753.39070290
2.10000000	2.50000000	-1753.38829601
2.10000000	2.40000000	-1753.38636523
2.10000000	2.30000000	-1753.38506780
2.10000000	2.20000000	-1753.38693691
2.10000000	2.10000000	-1753.38786900
2.10000000	2.00000000	-1753.38942978
2.10000000	1.90000000	-1753.39309043
2.10000000	1.80000000	-1753.39767753
2.10000000	1.70000000	-1753.40260001
2.10000000	1.60000000	-1753.40515322
2.10000000	1.50000000	-1753.40226800
2.20000000	3.40000000	-1753.39773798
2.20000000	3.30000000	-1753.39852999
2.20000000	3.20000000	-1753.39892975
2.20000000	3.10000000	-1753.39895905
2.20000000	3.00000000	-1753.39869349
2.20000000	2.90000000	-1753.39787254
2.20000000	2.80000000	-1753.39642249
2.20000000	2.70000000	-1753.39445722
2.20000000	2.60000000	-1753.39189210
2.20000000	2.50000000	-1753.38913170
2.20000000	2.40000000	-1753.38625444
2.20000000	2.30000000	-1753.38350457
2.20000000	2.20000000	-1753.38107492
2.20000000	2.10000000	-1753.38391351
2.20000000	2.00000000	-1753.38352432
2.20000000	1.90000000	-1753.38556748
2.20000000	1.80000000	-1753.38960083

2.20000000	1.70000000	-1753.39342680
2.20000000	1.60000000	-1753.39547999
2.20000000	1.50000000	-1753.39221015
2.30000000	3.40000000	-1753.40127500
2.30000000	3.30000000	-1753.40183904
2.30000000	3.20000000	-1753.40221317
2.30000000	3.10000000	-1753.40207891
2.30000000	3.00000000	-1753.40161514
2.30000000	2.90000000	-1753.40059058
2.30000000	2.80000000	-1753.39894417
2.30000000	2.70000000	-1753.39677100
2.30000000	2.60000000	-1753.39401830
2.30000000	2.50000000	-1753.39082525
2.30000000	2.40000000	-1753.38721329
2.30000000	2.30000000	-1753.38355129
2.30000000	2.20000000	-1753.38012925
2.30000000	2.10000000	-1753.38121512
2.30000000	2.00000000	-1753.37913586
2.30000000	1.90000000	-1753.37973795
2.30000000	1.80000000	-1753.38205961
2.30000000	1.70000000	-1753.38531605
2.30000000	1.60000000	-1753.38627728
2.30000000	1.50000000	-1753.38172005
1.50000000	1.40000000	-1753.43809979
1.50000000	1.30000000	-1753.40820070
1.60000000	1.40000000	-1753.43756372
1.60000000	1.30000000	-1753.40731751
1.70000000	1.40000000	-1753.43096805
1.70000000	1.30000000	-1753.40038271
1.80000000	1.40000000	-1753.42114409
1.80000000	1.30000000	-1753.39022909
1.90000000	1.40000000	-1753.40914332
1.90000000	1.30000000	-1753.37882957
2.00000000	1.40000000	-1753.39791199
2.00000000	1.30000000	-1753.36707367
2.10000000	1.40000000	-1753.38730543
2.10000000	1.30000000	-1753.35579027
2.20000000	1.40000000	-1753.37685801
2.20000000	1.30000000	-1753.34495058
2.30000000	1.40000000	-1753.36703437
2.30000000	1.30000000	-1753.33485919
2.40000000	3.40000000	-1753.39862705
2.40000000	3.30000000	-1753.39876758
2.40000000	3.20000000	-1753.40578646
2.40000000	3.10000000	-1753.40507718
2.40000000	3.00000000	-1753.40455623
2.40000000	2.90000000	-1753.40402617
2.40000000	2.80000000	-1753.40232479
2.40000000	2.70000000	-1753.39995573
2.40000000	2.60000000	-1753.39676451
2.40000000	2.50000000	-1753.39284418
2.40000000	2.40000000	-1753.38841653
2.40000000	2.30000000	-1753.38384353
2.40000000	2.20000000	-1753.37884209

2.40000000	2.10000000	-1753.37425288
2.40000000	2.00000000	-1753.37143634
2.40000000	1.90000000	-1753.37512327
2.40000000	1.80000000	-1753.37659492
2.40000000	1.70000000	-1753.37799141
2.40000000	1.60000000	-1753.37792831
2.40000000	1.50000000	-1753.37326059
2.40000000	1.40000000	-1753.37326000
2.50000000	3.40000000	-1753.40878578
2.50000000	3.30000000	-1753.40892064
2.50000000	3.20000000	-1753.40865808
2.50000000	3.10000000	-1753.40825988
2.50000000	3.00000000	-1753.40744519
2.50000000	2.90000000	-1753.40601017
2.50000000	2.80000000	-1753.40388505
2.50000000	2.70000000	-1753.40124350
2.50000000	2.60000000	-1753.39797994
2.50000000	2.50000000	-1753.39403499
2.50000000	2.40000000	-1753.38943598
2.50000000	2.30000000	-1753.38455553
2.50000000	2.20000000	-1753.37951424
2.50000000	2.10000000	-1753.37479005
2.50000000	2.00000000	-1753.37604301
2.50000000	1.90000000	-1753.37422825
2.50000000	1.80000000	-1753.37345010
2.50000000	1.70000000	-1753.37384099
2.50000000	1.60000000	-1753.37370388
2.50000000	1.50000000	-1753.36897209
2.50000000	1.40000000	-1753.35414925
2.50000000	1.30000000	-1753.32179836
2.60000000	3.40000000	-1753.41166882
2.60000000	3.30000000	-1753.41205628
2.60000000	3.20000000	-1753.41189306
2.60000000	3.10000000	-1753.41145738
2.60000000	3.00000000	-1753.41050927
2.60000000	2.90000000	-1753.40904940
2.60000000	2.80000000	-1753.40582070
2.60000000	2.70000000	-1753.40299030
2.60000000	2.60000000	-1753.39954044
2.60000000	2.50000000	-1753.39546213
2.60000000	2.40000000	-1753.39053581
2.60000000	2.30000000	-1753.38539893
2.60000000	2.20000000	-1753.37962983
2.60000000	2.10000000	-1753.37413243
2.60000000	2.00000000	-1753.37467284
2.60000000	1.90000000	-1753.37161674
2.60000000	1.80000000	-1753.36957866
2.60000000	1.70000000	-1753.36909295
2.60000000	1.60000000	-1753.36778316
2.60000000	1.50000000	-1753.36268660
2.60000000	1.40000000	-1753.34771664
2.60000000	1.30000000	-1753.31493719
2.70000000	3.40000000	-1753.41337277
2.70000000	3.30000000	-1753.41426673

2.70000000	3.20000000	-1753.41409790
2.70000000	3.10000000	-1753.41347822
2.70000000	3.00000000	-1753.41243534
2.70000000	2.90000000	-1753.41072948
2.70000000	2.80000000	-1753.40827923
2.70000000	2.70000000	-1753.40559393
2.70000000	2.60000000	-1753.40068932
2.70000000	2.50000000	-1753.39635211
2.70000000	2.40000000	-1753.39170133
2.70000000	2.30000000	-1753.38600190
2.70000000	2.20000000	-1753.37975063
2.70000000	2.10000000	-1753.37390677
2.70000000	2.00000000	-1753.37383153
2.70000000	1.90000000	-1753.36949066
2.70000000	1.80000000	-1753.36623088
2.70000000	1.70000000	-1753.36466156
2.70000000	1.60000000	-1753.36269520
2.70000000	1.50000000	-1753.35722661
2.70000000	1.40000000	-1753.34197028
2.70000000	1.30000000	-1753.30892983
2.80000000	3.40000000	-1753.41586901
2.80000000	3.30000000	-1753.41585450
2.80000000	3.20000000	-1753.41560836
2.80000000	3.10000000	-1753.41480808
2.80000000	3.00000000	-1753.41363654
2.80000000	2.90000000	-1753.41173006
2.80000000	2.80000000	-1753.40920666
2.80000000	2.70000000	-1753.40626254
2.80000000	2.60000000	-1753.40276064
2.80000000	2.50000000	-1753.39881904
2.80000000	2.40000000	-1753.39209109
2.80000000	2.30000000	-1753.38616780
2.80000000	2.20000000	-1753.38020026
2.80000000	2.10000000	-1753.37557377
2.80000000	2.00000000	-1753.37324457
2.80000000	1.90000000	-1753.36788339
2.80000000	1.80000000	-1753.36319640
2.80000000	1.70000000	-1753.36075430
2.80000000	1.60000000	-1753.35663659
2.80000000	1.50000000	-1753.34967618
2.80000000	1.40000000	-1753.33349325
2.80000000	1.30000000	-1753.29960070
2.90000000	3.40000000	-1753.41686013
2.90000000	3.30000000	-1753.41670083
2.90000000	3.20000000	-1753.41633959
2.90000000	3.10000000	-1753.41548757
2.90000000	3.00000000	-1753.41417428
2.90000000	2.90000000	-1753.41164078
2.90000000	2.80000000	-1753.40940401
2.90000000	2.70000000	-1753.40681674
2.90000000	2.60000000	-1753.40336588
2.90000000	2.50000000	-1753.39948801
2.90000000	2.40000000	-1753.39213246
2.90000000	2.30000000	-1753.38689317

2.90000000	2.20000000	-1753.38226475
2.90000000	2.10000000	-1753.37640291
2.90000000	2.00000000	-1753.37260739
2.90000000	1.90000000	-1753.36622220
2.90000000	1.80000000	-1753.36040409
2.90000000	1.70000000	-1753.35178800
2.90000000	1.60000000	-1753.35305576
2.90000000	1.50000000	-1753.34556753
2.90000000	1.40000000	-1753.32898314
2.90000000	1.30000000	-1753.29481243
3.00000000	3.40000000	-1753.41725272
3.00000000	3.30000000	-1753.41697706
3.00000000	3.20000000	-1753.41632488
3.00000000	3.10000000	-1753.41549912
3.00000000	3.00000000	-1753.41393395
3.00000000	2.90000000	-1753.41002911
3.00000000	2.80000000	-1753.40968171
3.00000000	2.70000000	-1753.40662716
3.00000000	2.60000000	-1753.40305496
3.00000000	2.50000000	-1753.40010272
3.00000000	2.40000000	-1753.39563674
3.00000000	2.30000000	-1753.39014063
3.00000000	2.20000000	-1753.38378327
3.00000000	2.10000000	-1753.37720902
3.00000000	2.00000000	-1753.37185758
3.00000000	1.90000000	-1753.36506113
3.00000000	1.80000000	-1753.35659257
3.00000000	1.70000000	-1753.35391591
3.00000000	1.60000000	-1753.34976565
3.00000000	1.50000000	-1753.34191080
3.00000000	1.40000000	-1753.32509304
3.00000000	1.30000000	-1753.29032333
3.20000000	3.40000000	-1753.41595209
3.20000000	3.30000000	-1753.41529231
3.20000000	3.20000000	-1753.40847057
3.20000000	3.10000000	-1753.40864408
3.20000000	3.00000000	-1753.40840341
3.20000000	2.90000000	-1753.40797094
3.20000000	2.80000000	-1753.40819135
3.20000000	2.70000000	-1753.41000728
3.20000000	2.60000000	-1753.40596303
3.20000000	2.50000000	-1753.40290250
3.20000000	2.40000000	-1753.39784445
3.20000000	2.30000000	-1753.39201299
3.20000000	2.20000000	-1753.38557187
3.20000000	2.10000000	-1753.37854743
3.20000000	2.00000000	-1753.37091225
3.20000000	1.90000000	-1753.36315147
3.20000000	1.80000000	-1753.35649924
3.20000000	1.70000000	-1753.35171798
3.20000000	1.60000000	-1753.34729911
3.20000000	1.50000000	-1753.33992680
3.20000000	1.40000000	-1753.33992600
1.65000000	1.30000000	-1753.40436600

1.65000000	1.40000000	-1753.43482200
1.65000000	1.50000000	-1753.44691800
1.65000000	1.60000000	-1753.44838200
1.65000000	1.70000000	-1753.44445400
1.65000000	1.80000000	-1753.43770800
1.65000000	1.90000000	-1753.43037100
1.65000000	2.00000000	-1753.42385900
1.65000000	2.10000000	-1753.41789900
1.65000000	2.20000000	-1753.41329900
1.65000000	2.30000000	-1753.40990900
1.65000000	2.40000000	-1753.40773600
1.65000000	2.50000000	-1753.40627800
1.65000000	2.60000000	-1753.40534500
1.65000000	2.70000000	-1753.40478900
1.65000000	2.80000000	-1753.40420200
1.65000000	2.90000000	-1753.42596000
1.65000000	3.00000000	-1753.43004400
1.65000000	3.10000000	-1753.43319600
1.65000000	3.20000000	-1753.43534500
1.65000000	3.30000000	-1753.43650100
1.65000000	3.40000000	-1753.43723700
1.55000000	1.30000000	-1753.40807400
1.55000000	1.40000000	-1753.43806800
1.55000000	1.50000000	-1753.44919000
1.55000000	1.60000000	-1753.44915600
1.55000000	1.70000000	-1753.44282200
1.55000000	1.80000000	-1753.43298400
1.55000000	1.90000000	-1753.42165000
1.55000000	2.00000000	-1753.40979100
1.55000000	2.10000000	-1753.39792100
1.55000000	2.20000000	-1753.38656300
1.55000000	2.30000000	-1753.377287
1.55000000	2.40000000	-1753.367722
1.55000000	2.50000000	-1753.359284
1.55000000	2.60000000	-1753.351762
1.55000000	2.70000000	-1753.345108
1.55000000	2.80000000	-1753.339044
1.55000000	2.90000000	-1753.334238
1.55000000	3.00000000	-1753.330501
1.55000000	3.10000000	-1753.388450
1.55000000	3.20000000	-1753.389242
1.55000000	3.30000000	-1753.387982
1.55000000	3.40000000	-1753.384398
1.40000000	1.30000000	-1753.395652
1.40000000	1.40000000	-1753.425115
1.40000000	1.50000000	-1753.435814
1.40000000	1.60000000	-1753.435499
1.40000000	1.70000000	-1753.428916
1.40000000	1.80000000	-1753.419025
1.40000000	1.90000000	-1753.407625
1.40000000	2.00000000	-1753.396061
1.40000000	2.10000000	-1753.385143
1.40000000	2.20000000	-1753.374057
1.40000000	2.30000000	-1753.363696

1.40000000	2.40000000	-1753.354338
1.40000000	2.50000000	-1753.345946
1.40000000	2.60000000	-1753.338406
1.40000000	2.70000000	-1753.331448
1.40000000	2.80000000	-1753.325458
1.40000000	2.90000000	-1753.320575
1.40000000	3.00000000	-1753.316323
1.40000000	3.10000000	-1753.377963
1.40000000	3.20000000	-1753.378786
1.40000000	3.30000000	-1753.377259
1.40000000	3.40000000	-1753.373418
1.30000000	3.50000000	-1753.392387
1.40000000	3.50000000	-1753.424586
1.50000000	3.50000000	-1753.437635
1.60000000	3.50000000	-1753.439370
1.70000000	3.50000000	-1753.434682
1.80000000	3.50000000	-1753.426755
1.90000000	3.50000000	-1753.417198
2.00000000	3.50000000	-1753.407265
2.10000000	3.50000000	-1753.397387
2.20000000	3.50000000	-1753.388200
2.30000000	3.50000000	-1753.379921
2.40000000	3.50000000	-1753.372631
2.50000000	3.50000000	-1753.402154
2.60000000	3.50000000	-1753.404810
2.70000000	3.50000000	-1753.406684
2.80000000	3.50000000	-1753.404504
2.90000000	3.50000000	-1753.411185
3.00000000	3.50000000	-1753.411255
1.30000000	3.60000000	-1753.381999
1.40000000	3.60000000	-1753.423950
1.50000000	3.60000000	-1753.437283
1.60000000	3.60000000	-1753.439239
1.70000000	3.60000000	-1753.434700
1.80000000	3.60000000	-1753.427014
1.90000000	3.60000000	-1753.417671
2.00000000	3.60000000	-1753.407846
2.10000000	3.60000000	-1753.398157
2.20000000	3.60000000	-1753.389070
2.30000000	3.60000000	-1753.380776
2.40000000	3.60000000	-1753.373523
2.50000000	3.60000000	-1753.367523
2.60000000	3.60000000	-1753.404362
2.70000000	3.60000000	-1753.406267
2.80000000	3.60000000	-1753.403951
2.90000000	3.60000000	-1753.411462
3.00000000	3.60000000	-1753.410823
3.10000000	3.60000000	-1753.410610
3.20000000	3.60000000	-1753.409297

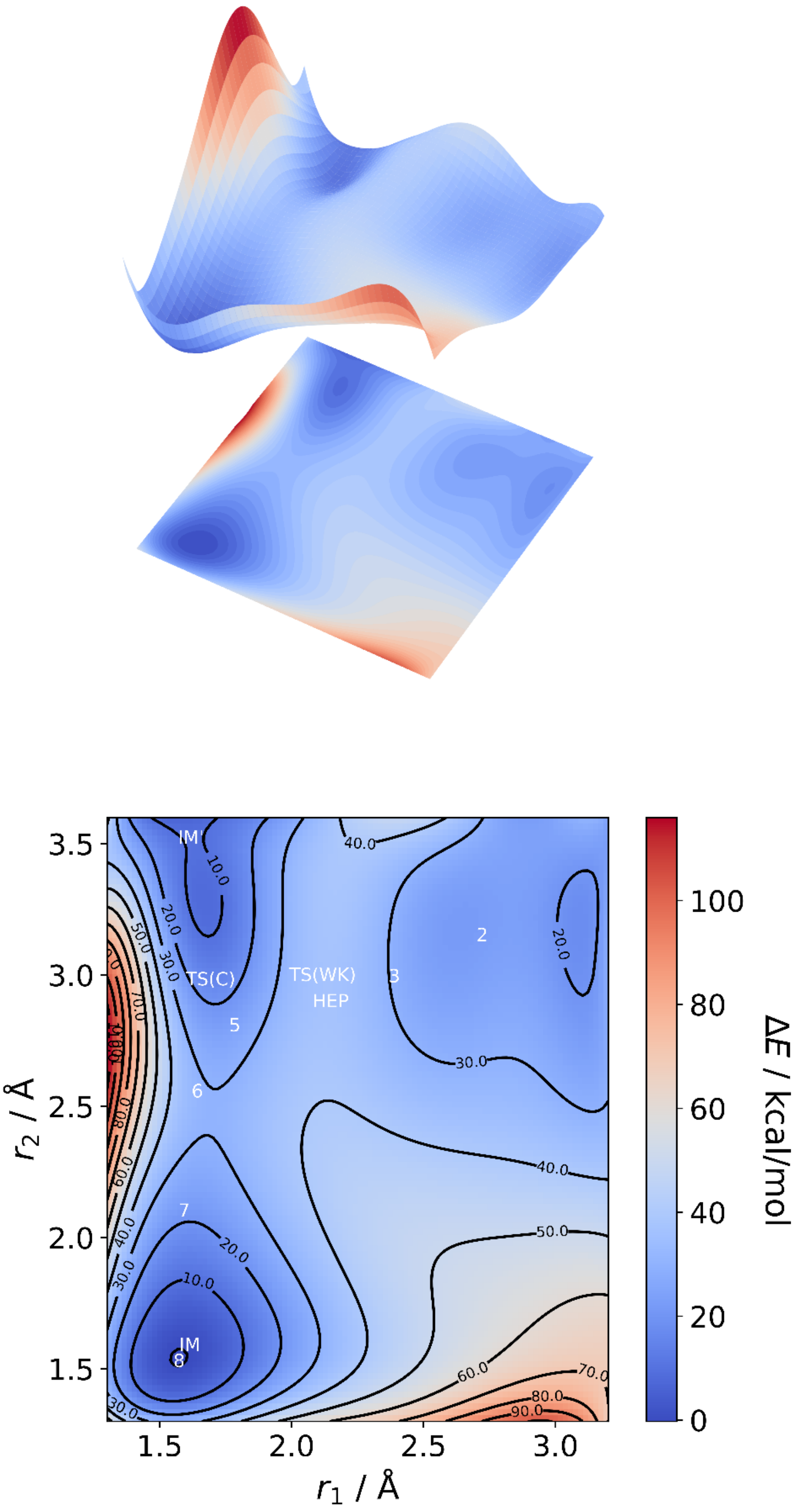

Figure S3. Above: Used raw interpolated PES using a cubic spline using *scipy.interp2d*. Below: Isosurface of the PES using a cubic spline using *scipy.interp2d*. Labelled onto the isosurface **TS3** = **TS(WK)**, **TS4** = **T(C)**, **IM2** = **IM'** and **IM1** = **IM**. Further, the points of a NEB between starting material **16** and **5** towards point **8** crossing the Highest Energy Point (HEP) near **TS3**.

5. References

1. Wang, X.; Barbosa, J.; Blomgren, P.; Bremer, M. C.; Chen, J.; Crawford, J. J.; Deng, W.; Dong, L.; Eigenbrot, C.; Gallion, S.; et al., Discovery of Potent and Selective Tricyclic Inhibitors of Bruton's Tyrosine Kinase with Improved Druglike Properties. *ACS Med. Chem. Lett.* **2017**, *8*, 608–613.2
2. Nenajdenko, V. G.; Moiseev, A. M.; Balenkova, E. S., A novel method for the oxidation of thiophenes. Synthesis of thiophene 1,1-dioxides containing electron-withdrawing substituents. *Russ. Chem. Bull.* **2004**, *53*, 2241–2247.
3. Sakamaki, S.; Kawanishi, E.; Nomura, S.; Ishikawa, T., Aryl-β-C-glucosidation using glucal boronate: application to the synthesis of tri-O-methylnorbergenin. *Tetrahedron* **2012**, *68*, 5744–5753.
4. Shaabani, A.; Mirzaei, P.; Naderi, S.; Lee, D. G., Green oxidations. The use of potassium permanganate supported on manganese dioxide. *Tetrahedron* **2004**, *60*, 11415–11420.
5. Zeng, Z.; Zhao, Y.; Zhang, Y., Divergent total syntheses of five illudalane sesquiterpenes and assignment of the absolute configuration. *Chem. Commun.* **2019**, *55*, 4250–4253.
6. Li, L.; Liu, Q.; Chen, J.; Huang, Y., Alcohol-Directed ortho-C–H Alkenylation. *Synlett* **2019**, *30*, 1366–1370.
7. Zhang Y.; Wu W.; Yang G.; Li Z.; Sun J.; Li J.; Li J., Chen S., *PCT Int. Appl.* **2019**, *WO 2019072235 A1*
8. Tanaka, R.; Nakano, Y.; Suzuki, D.; Urabe, H.; Sato, F., Selective Preparation of Benzyltitanium Compounds by the Metalative Reppe Reaction. Its Application to the First Synthesis of Alcyopterosin A. *J. Am. Chem. Soc.* **2002**, *124*, 9682–9683.
9. Welsch, T.; Tran, H.-A.; Witulski, B., Total Syntheses of the Marine Illudalanes Alcyopterosin I, L, M, N, and C. *Org. Lett.* **2010**, *12*, 5644–5647.
10. Tavakoli, A.; Dudley, G. B., Synthesis of 4,4-Dimethyl-1,6-heptadiyne and Alcyopterosin O. *Org. Lett.* **2020**, *22*, 8947–8951.
11. Hoang, T. T.; Birepinte, M.; Kramer, N. J.; Dudley, G. B., Six-Step Synthesis of Alcyopterosin A, a Bioactive Illudalane Sesquiterpene with a gem-Dimethylcyclopentane Ring. *Org. Lett.* **2016**, *18*, 3470–3473.
12. Palermo, J. A.; Rodríguez Brasco, M. F.; Spagnuolo, C.; Seldes, A. M., Illudalane Sesquiterpenoids from the Soft Coral Alcyonium paessleri: The First Natural Nitrate Esters. *J. Org. Chem.* **2000**, *65*, 4482–4486.
13. (a) Neese, F., The ORCA program system. *Wiley Interdiscip. Rev. Comput. Mol. Sci.* **2011**, *2*, 73-78; (b) Neese, F., Software update: the ORCA program system, version 4.0. *Wiley Interdiscip. Rev. Comput. Mol. Sci.* **2017**, *8*.

(14) Neese, F.; Wennmohs, F.; Hansen, A.; Becker, U., Efficient, approximate and parallel Hartree–Fock and hybrid DFT calculations. A 'chain-of-spheres' algorithm for the Hartree–Fock exchange. *Chem. Phys.* **2009**, *356*, 98-109.

(15) (a) Stoychev, G. L.; Auer, A. A.; Neese, F., Automatic Generation of Auxiliary Basis Sets. *J. Chem. Theory Comput.* **2017**, *13*, 554-562; (b) Eichkorn, K.; Weigend, F.; Treutler, O.; Ahlrichs, R., Auxiliary basis sets for main row atoms and transition metals and their use to approximate Coulomb potentials. *Theor. Chim. Acta* **1997**, *97*, 119-124; (c) Weigend, F., Accurate Coulomb-fitting basis sets for H to Rn. *Phys. Chem. Chem. Phys.* **2006**, *8*, 1057-1065; (d) Weigend, F., A fully direct RI-HF algorithm: Implementation, optimised auxiliary basis sets, demonstration of accuracy and efficiency. *Phys. Chem. Chem. Phys.* **2002**, *4*, 4285-4291.

(16) (a) Young, T. A.; Silcock, J. J.; Sterling, A. J.; Duarte, F., autodE: Automated Calculation of Reaction Energy Profiles- Application to Organic and Organometallic Reactions. *Angew. Chem. Int. Ed.* **2021**, *60*, 4266-4274; (b) Bannwarth, C.; Ehlert, S.; Grimme, S., GFN2-xTB-An Accurate and Broadly Parametrized Self-Consistent Tight-Binding Quantum Chemical Method with

Multipole Electrostatics and Density-Dependent Dispersion Contributions. *J. Chem. Theory Comput.* **2019**, *15*, 1652-1671.
(17) (a) Grimme, S.; Antony, J.; Ehrlich, S.; Krieg, H., A consistent and accurate ab initio parametrization of density functional dispersion correction (DFT-D) for the 94 elements H-Pu. *J. Chem. Phys.* **2010**, *132*, 154104; (b) Grimme, S.; Ehrlich, S.; Goerigk, L., Effect of the damping function in dispersion corrected density functional theory. *J. Comput. Chem.* **2011**, *32*, 1456-1465.
(18) Liakos, D. G.; Neese, F., Is It Possible To Obtain Coupled Cluster Quality Energies at near Density Functional Theory Cost? Domain-Based Local Pair Natural Orbital Coupled Cluster vs Modern Density Functional Theory. *J. Chem. Theory Comput.* **2015**, *11*, 4054-4063.
(19) Liakos, D. G.; Sparta, M.; Kesharwani, M. K.; Martin, J. M.; Neese, F., Exploring the Accuracy Limits of Local Pair Natural Orbital Coupled-Cluster Theory. *J. Chem. Theory Comput.* **2015**, *11*, 1525-1539.
(20) Grimme, S., Supramolecular binding thermodynamics by dispersion-corrected density functional theory. *Chem. Eur. J.* **2012**, *18*, 9955-9964.
(21) Young, T., duartegroup/otherm. **2020**.
(22) Linder, M.; Brinck, T., On the method-dependence of transition state asynchronicity in Diels-Alder reactions. *Phys. Chem. Chem. Phys.* **2013**, *15*, 5108-5114.
(23) (a) Black, K.; Liu, P.; Xu, L.; Doubleday, C.; Houk, K. N., Dynamics, transition states, and timing of bond formation in Diels-Alder reactions. *Proc. Natl. Acad. Sci. USA* **2012**, *109*, 12860-12865; (b) Yang, Z.; Yang, S.; Yu, P.; Li, Y.; Doubleday, C.; Park, J.; Patel, A.; Jeon, B. S.; Russell, W. K.; Liu, H. W.; Russell, D. H.; Houk, K. N., Influence of water and enzyme SpnF on the dynamics and energetics of the ambimodal [6+4]/[4+2] cycloaddition. *Proc. Natl. Acad. Sci. USA* **2018**, *115*, E848-E855.
(24) Zhang, H.; Novak, A. J. E.; Jamieson, C. S.; Xue, X. S.; Chen, S.; Trauner, D.; Houk, K. N., Computational Exploration of the Mechanism of Critical Steps in the Biomimetic Synthesis of Preuisolactone A, and Discovery of New Ambimodal (5 + 2)/(4 + 2) Cycloadditions. *J. Am. Chem. Soc.* **2021**, *143*, 6601-6608.
(25) Domingo, L. R.; Arnó, M.; Andrés, J., Influence of Reactant Polarity on the Course of the Inverse-Electron-Demand Diels−Alder Reaction. A DFT Study of Regio- and Stereoselectivity, Presence of Lewis Acid Catalyst, and Inclusion of Solvent Effects in the Reaction between Nitroethene and Substituted Ethenes. *J. Org. Chem.* **1999**, *64*, 5867-5875.
(26) Bickelhaupt, F. M.; Houk, K. N., Analyzing Reaction Rates with the Distortion/Interaction-Activation Strain Model. *Angew. Chem. Int. Ed.* **2017**, *56*, 10070-10086.
(27) E. D. Glendening, J., K. Badenhoop, A. E. Reed, J. E. Carpenter, J. A. Bohmann, C. M. Morales, P. Karafiloglou, C. R. Landis, and F. Weinhold, *NBO 7.0*. Theoretical Chemistry Institute, University of Wisconsin, Madison
(28) (a) Lee, S.; Goodman, J. M., VRAI-selectivity: calculation of selectivity beyond transition state theory. *Org. Biomol. Chem.* **2021**, *19*, 3940-3947; (b) Lee, S.; Goodman, J. M., Rapid Route-Finding for Bifurcating Organic Reactions. *J. Am. Chem. Soc.* **2020**, *142*, 9210-9219.
(29) Yang, Z.; Dong, X.; Yu, Y.; Yu, P.; Li, Y.; Jamieson, C.; Houk, K. N., Relationships between Product Ratios in Ambimodal Pericyclic Reactions and Bond Lengths in Transition Structures. *J. Am. Chem. Soc.* **2018**, *140*, 3061-3067.
